# Impact of osmotic stress on the phosphorylation and subcellular location of *Listeria monocytogenes* stressosome proteins

**DOI:** 10.1038/s41598-020-77738-z

**Published:** 2020-11-30

**Authors:** Charlotte Dessaux, Duarte N. Guerreiro, M. Graciela Pucciarelli, Conor P. O’Byrne, Francisco García-del Portillo

**Affiliations:** 1 Laboratory of Intracellular Bacterial Pathogens, National Centre for Biotechnology (CNB)-CSIC, Darwin 3, 28049 Madrid, Spain; 2 Bacterial Stress Response Group, Microbiology, School of Natural Sciences, National University of Ireland Galway, Galway, H91 TK33 Ireland; 3 Department of Molecular Biology, Centre of Molecular Biology ‘Severo Ochoa’ (CBMSO)-CSIC, Universidad Autónoma de Madrid, 28049 Madrid, Spain

**Keywords:** Microbiology, Bacteria

## Abstract

*Listeria monocytogenes* responds to environmental stress using a supra-macromolecular complex, the stressosome, to activate the stress sigma factor SigB. The stressosome structure, inferred from *in vitro*-assembled complexes, consists of the core proteins RsbR (here renamed RsbR1) and RsbS and, the kinase RsbT. The active complex is proposed to be tethered to the membrane and to support RsbR1/RsbS phosphorylation by RsbT and the subsequent release of RsbT following signal perception. Here, we show in actively-growing cells that *L. monocytogenes* RsbR1 and RsbS localize mostly in the cytosol in a fully phosphorylated state regardless of osmotic stress. RsbT however distributes between cytosolic and membrane-associated pools. The kinase activity of RsbT on RsbR1/RsbS and its requirement for maximal SigB activation in response to osmotic stress were demonstrated in vivo. Cytosolic RsbR1 interacts with RsbT, while this interaction diminishes at the membrane when RsbR1 paralogues (RsbR2, RsbR3 and RsbL) are present. Altogether, the data support a model in which phosphorylated RsbR1/RsbS may sustain basal SigB activity in unstressed cells, probably assuring a rapid increase in such activity in response to stress. Our findings also suggest that in vivo the active RsbR1-RsbS-RsbT complex forms only transiently and that membrane-associated RsbR1 paralogues could modulate its assembly.

## Introduction

**Figure 1:**
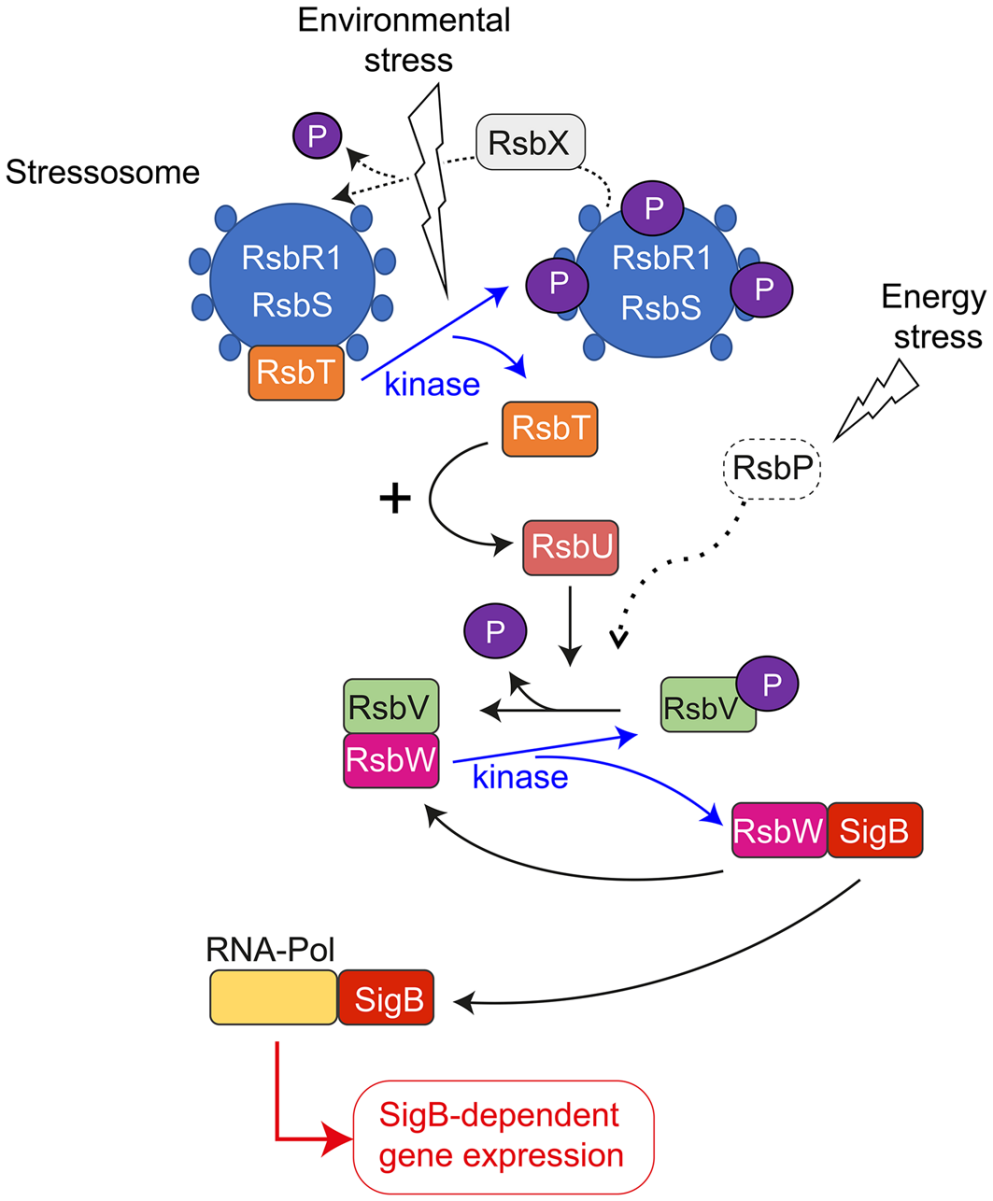
Model of the regulatory signalling cascade that controls SigB activity. The model highlights the role of RsbV phosphorylation in controlling SigB activity, through its modulation of the levels of free RsbW, the anti-sigma factor that sequesters SigB. RsbT is released from the stressosome following activation of its kinase activity in response to stress signals and this promotes its association with RsbU, which in turn promotes the dephosphorylation of RsbV and subsequent release of SigB from RsbW. The scheme is based mostly in data obtained in *B. subtilis*, including the alternative stimulatory pathway that responds to energy stress and involves RsbP, a protein that is absent in *L. monocytogenes*. The putative role of RsbX as phosphatase reverting the stimulatory state of the stressosome (with RsbT released from the complex) is also depicted. RNA-Pol, RNA polymerase. See text for further details.

Stress response is central for all organisms to adapt and survive in adverse environmental conditions. Gram-positive bacteria like *Bacillus subtilis* and *Listeria monocytogenes* use an alternative sigma factor, named SigB (σ^B^), that binds to the RNA polymerase core to drive the stress response by altering expression of up to 300 genes^1–5^. SigB activity is controlled by an anti-sigma factor, RsbW^6^. Bacteria adjust the amount of RsbW capable of sequestering SigB using the anti-anti-sigma factor RsbV. RsbW can associate preferentially with RsbV when the latter is dephosphorylated through the action of RsbU, a specific phosphatase. The RsbV:RsbV ~ P ratio therefore determines the amount of free SigB that is available to associate with the core RNA polymerase and, as consequence, the intensity of the transcriptional output response^7,8^. In *B. subtilis*, RsbV phosphorylation levels can be modulated by either RsbU or RsbP phosphatases depending on the type of signal perceived whether it is environmental or energy stress, respectively^9–11^. *Listeria monocytogenes* lacks the RsbP-like phosphatase and its RsbQ cofactor found in *B. subtilis*, which indicates that, in this bacterium, the cascades responding to environmental and energy stresses occur in a single RsbU-dependent pathway^12^ (Fig. 1).

The integration of environmental stress signals was first proposed in *B. subtilis* and, later confirmed in *L. monocytogenes*, to rely on a supra-macromolecular complex named the stressosome. This complex was assembled in vitro using purified recombinant proteins^13–16^. These experiments established the key structural roles of two core proteins: RsbS and RsbR, this latter known as RsbRA in *B. subtilis* and, proposed here to be renamed RsbR1 for the *L. monocytogenes* paralogue. These two core proteins are hypothesised to be phosphorylated by the kinase RsbT following the detection of a stress sensory input. Recent in vitro studies performed with purified *L. monocytogenes* proteins showed that the RsbR1-RsbS-RsbT complex, as visualized by negative-stain electron microscopy, assembles as a more homogeneous particle than the RsbR1-RsbS complex^16^. The atomic structure of the RsbR1-RsbS-RsbT complex, as determined by cryogenic electron microscopy, reveals an icosahedron-like architecture with sensory domains protruding from the surface (turrets), produced by 20 RsbR1-dimers interacting with 10 RsbS dimers and 10 RsbT dimers^16^. The widely accepted model in both *B. subtilis* and *L. monocytogenes* involves assembly of RsbR1 and RsbS in a mono- and unphosphorylated stage, respectively^16,17^. The current model predicts that stress signal perception stimulates the kinase activity of RsbT to phosphorylate the second site in RsbR1 and the single site present in RsbS^18–22^. These phosphorylation events cause the release of RsbT from the stressosome complex and allow its interaction with RsbU, leading to the dephosphorylation of RsbV ~ P, sequestration of RsbW and thereby SigB activation^10,23,24^. Most of these steps have been inferred from in vitro studies based on recombinant proteins lacking kinase or phosphatase activities. For example, in one study in *B. subtilis* an RsbT variant lacking its kinase activity was unable to form a complex together with RsbRA and RsbS^25^.

Recent studies in *L. monocytogenes* have also uncovered the importance of the subcellular location of the stressosome to properly integrate the external stress signal. An N-terminomics analysis in *L. monocytogenes* identified a membrane spanning miniprotein, Prli42, which was proposed to tether the stressosome complex to the membrane in response to oxidative stress by interacting with RsbR1^15^. Interestingly, immunoprecipitation assays shown in that study indicated that Prli42 also interacts with three RsbR1 paralogue proteins, Lmo0161, Lmo0799 and Lmo1642, with a fourth paralogue, Lmo1842, not detected in these assays. Lmo0799 was recently renamed as RsbL^26^ to reflect its involvement in light sensing. We propose here to rename Lmo0161, Lmo1642 and Lmo1842 as RsbR2, RsbR3 and RsbR4, respectively. RsbR2, RsbR3, RbsR4 and RsbL are orthologues of the *B. subtilis* proteins RsbRB, RsbRC, RsbRD, and YtvA, respectively. Although not specifically discussed in the study of Impens et al. in *L. monocytogenes*^15^, the immunoprecipitation of RsbR1 paralogues with the miniprotein Prli42 opens the possibility of heterogeneous stressosome complexes associating with the plasma membrane. Even though the core stressosome of *L. monocytogenes* clearly comprises RsbR1, RsbS and RsbT, it is not yet clear how the in vivo structure of the stressosome varies over time and space, particularly in relation to the relative abundance of the RsbR1 paralogues.

Despite the information obtained from structural analyses and the protein–protein interactions characterized in vitro within the RsbR1-RsbS-RsbT complex, there remains much to learn about the dynamics of these interactions and how they are modulated by live cells in response to stress. Here, we have addressed in actively growing *L. monocytogenes* cells the subcellular location of the main stressosome components, their interactions and the phosphorylation status in the absence/presence of osmotic stress. Unexpectedly, some of these proteins were detected mostly in the cytosol and with phosphorylation states that remained unaltered in response to hyperosmolarity. Our findings also suggest that some of the RsbR1 paralogues may negatively modulate the functioning of the stressosome in vivo.

## Results

### RsbR1 and RsbS are mainly cytosolic in *L. monocytogenes* live cells regardless of osmotic stress

**Figure 2:**
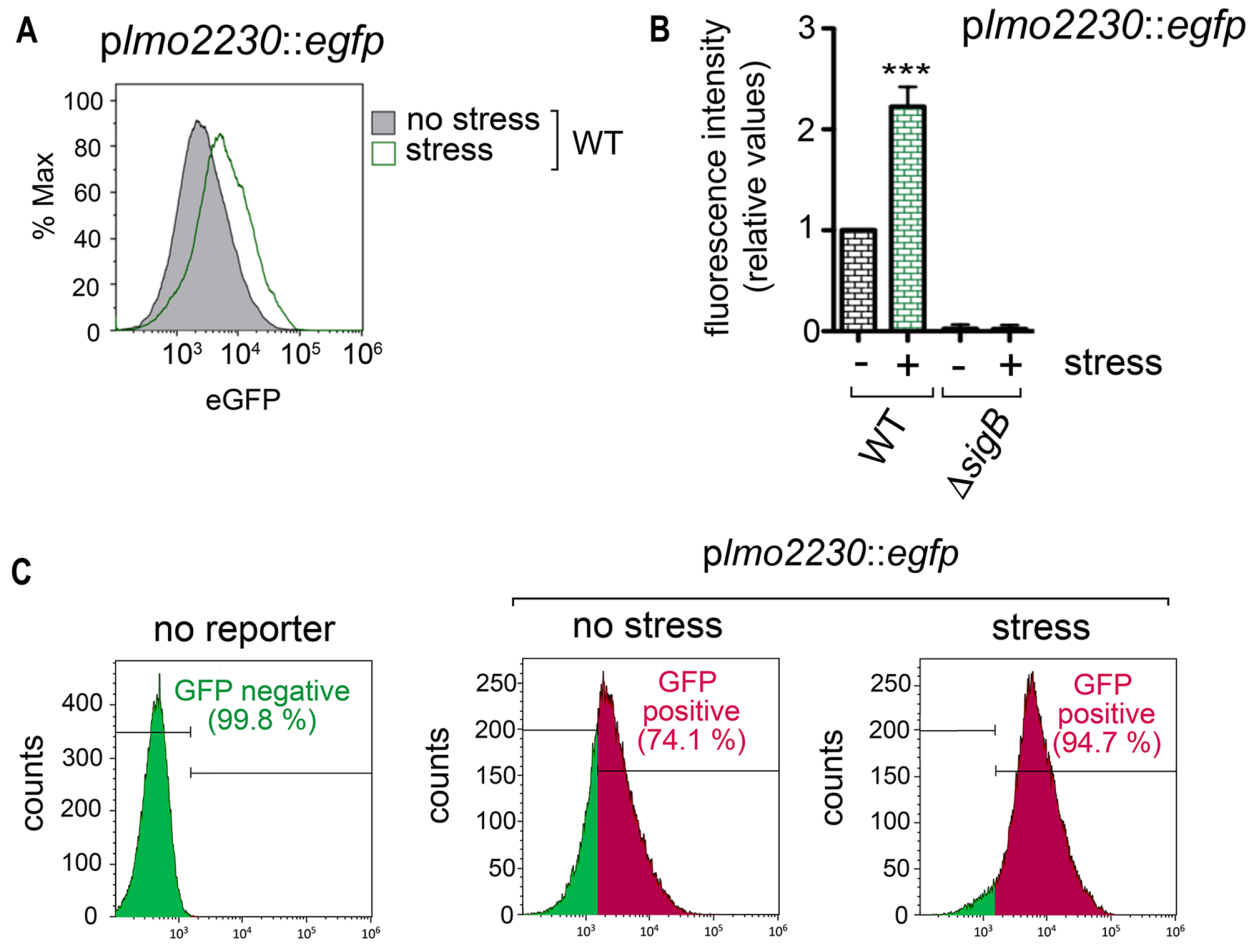
Monitoring of the *L. monocytogenes* response to osmotic stress with the P_*lmo2230*_::*egfp* reporter fusion. (**A**) SigB activity measured as expression of the reporter fusion P_*lmo2230*_::*egfp*, engineered from the SigB-regulated gene *lmo2230*. Reporter expression was measured after 30 min incubation in BHI medium containing 0.5 M NaCl. Shown are data representative of a total of three biological replicates. (**B**) Mean fluorescence intensity quantified in wild-type and D*sigB* strains in unstress and stress conditions. ***, *P* < 0.0005 (one-way ANOVA with Bonferroni’s multiple comparisons test). (**C**) Quantification of the proportion of the bacterial population positive for eGFP levels in the indicated conditions.

Recent studies performed in *L. monocytogenes* show that RsbR (hereinafter renamed as RsbR1) and RsbS are core proteins of the stressosome complex, which can be tethered to the membrane by the miniprotein Prli42 for the response to oxidative stress^15,16^. However, the distribution of these core proteins or the kinase RsbT between cytosol and membrane has been addressed exclusively for RsbR1^15^. To assess the relative levels of the stressosome components at each subcellular location in vivo, we prepared cytosol and membrane fractions of *L. monocytogenes* wild-type strain EGD-e growing exponentially in the nutrient-rich medium BHI. A culture of bacteria exposed for 30 min to BHI supplemented with 0.5 M NaCl was run in parallel to determine the effect of osmotic stress on the location or levels of these proteins. Exposure to this salt concentration of 0.5 M NaCl did not alter *L. monocytogenes* viability or morphology (Supplementary Fig. S1). By monitoring expression of a highly SigB-dependent gene such as *lmo2230* using *a* P_*lmo2230*_::*egfp* fluorescent reporter fusion, we confirmed that the 30 min exposure to 0.5 M NaCl is sufficient to activate SigB (Fig. 2A,B)^27,28^. Importantly, we also observed a noticeable basal activity of SigB in unstressed cells using this reporter. Thus, the cytometry assays revealed that ~ 75% of the bacterial population displayed a positive signal for eGFP above the background that increased to ~ 95% in the population exposed to salt stress (Fig. 2C). These findings are consistent with a previous study reporting basal SigB activity in unstressed *L. monocytogenes* cells^29^.

**Figure 3:**
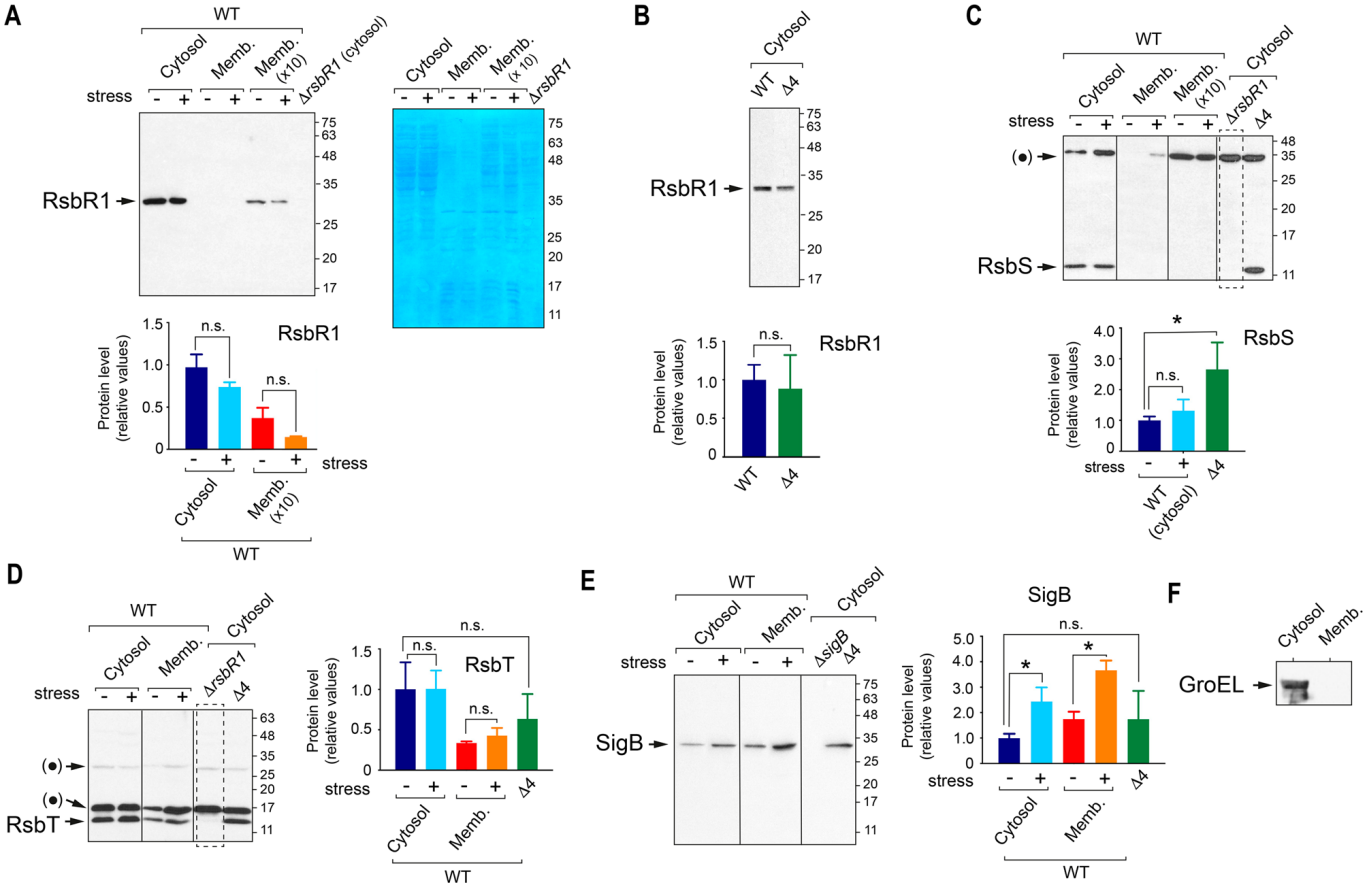
The stressosome proteins RsbR1, RsbS and RsbT abound in the cytosol of *L. monocytogenes* strain EGD-e, regardless of osmotic stress. Distribution of RsbR1 (**A**,**B**), RsbS (**C**), RsbT (**D**), and SigB (**E**) in cytosol and membrane fractions of *L. monocytogenes* wild type strain EGD-e and derivative strains including a polar Δ*rsbR1* mutant, not expressing RsbR1, RsbS and RsbT; a Δ4 mutant lacking function of all four RsbR1 paralogues known (see text for details); and a Δ*sigB* mutant. Cytosol and membrane extracts were prepared from unstressed and stressed (exposed 30 min to 0.5 M NaCl) bacteria. Panel A includes a a membrane corresponding to the anti-RsbR1 western blot that was subsequently stained with Coomassie dye. This procedure was used for every blot to verify the proper adjustment of the samples. Quantification of the signals obtained in the blots from three independent biological replicates and for each of the stressosome proteins, is also shown as mean values and standard deviation. *, *P* < 0.05; n.s. not significant (one-way ANOVA with Tukey’s multiple comparisons test). (**F**) Control immunoblot with anti-GroEL antibodies discarding cross-contamination between cytosolic and membrane fractions. Bands indicated as ( • ) in panels (**C**,**D**) correspond to unspecific protein recognition. Molecular weight markers are indicated at the right side of each image. Vertical lines within blots indicate separate parts that were grouped from different positions of the same blot. Dashed rectangles highlight the gel lanes corresponding to negative control strains. See Supplementary Fig. S6 for images of full blots.

The immunoblot analyses to detect RsbR1, RsbS and RsbT in the subcellular fractions were carried out using affinity-purified antibodies. An important control included in these experiments was a polar Δ*rsbR1* mutant known to have affected the expression of the downstream genes *rsbS* and *rsbT*^30^. These assays showed that most of the RsbR1 and RsbS were in cytosol, regardless of presence or absence of osmotic stress (Fig. 3A–C). Although we consistently failed to detect RsbS by immunoblot in membrane fractions (Fig. 3C), RsbR1 was however visualized in highly concentrated (tenfold) membrane fractions (Fig. 3A). Control immunoblots against purified RsbR1 and RsbS showed that the antibodies have equal affinity (Supplementary Fig. S2). Unlike RsbS, the kinase RsbT was however easily detected in both the cytosolic and membrane fractions (Fig. 3D). No alterations in the amount of RsbT present in cytosol and membrane were observed in response to osmotic stress.

Similar to RsbT, we unexpectedly detected SigB in both cytosolic and membrane fractions (Fig. 3E). The possibility of cross-contamination between subcellular fractions was discarded as immunoblot assays showed that the abundant chaperone protein GroEL was present exclusively in the cytosol (Fig. 3F). Unlike the three stressosome proteins tested, SigB protein levels clearly increased in response to osmotic stress in both the cytosol and membrane fractions (Fig. 3E). Such increased SigB levels agree with the positive autoregulation that SigB exerts over the *rsbV-rsbW-sigB-rsbX* operon in stressed bacteria^31^. The unexpected membrane localisation of SigB led us to test whether it was facilitated by transertion of SigB-regulated proteins. To investigate this, we inhibited protein synthesis concomitantly with the increase in osmolarity. Under these conditions, lower levels of membrane-associated SigB were detected relative to the untreated control samples (Supplementary Fig. S3). This finding suggests that some protein(s) upregulated by SigB upon stress could favour membrane localisation of this sigma factor.

Lastly, we sought to determine the distribution of RsbR1, RsbS and RsbT in the absence of the RsbR1 paralogues. To do this, we analysed a *L. monocytogenes* mutant that lacks the RsbR1 paralogues Lmo0161, Lmo1642 and Lmo1842—here renamed as RsbR2, RsbR3 and RsbR4, respectively- and bears an additional loss-of-function mutation (C56A) in the fourth paralogue RsbL^26^. For simplicity, this quadruple mutant (with the genotype Δr*sbR2*, Δ*rsbR3*, Δ*rsbR4*, *rsbL-C56A*) is described hereinafter as “Δ4”. The lack of functional RsbR1 paralogues resulted in increased RsbS levels in the cytosol, an effect not observed for the other proteins tested, RsbR1, RsbT or SigB (Fig. 3A–E). Taken together, these data pointed to a predominant localisation of *L. monocytogenes* stressosome proteins to the cytosol, irrespective of the presence or absence of osmotic stress and increased amounts of RsbS in the cytosol of bacteria having no functional RsbR1 paralogues.

### *Listeria monocytogenes* RsbR1 and RsbS are predominantly phosphorylated regardless of stress

The current models of stressosome activation in *B. subtilis* and *L. monocytogenes* involve phosphorylation of RsbRA (RsbR1) and RsbS by the kinase RsbT concomitant with the exposure to stress^16,17^. *L. monocytogenes* RsbR1 has two conserved phosphorylatable threonine residues (T175, T209) with one of them, T175, being phosphorylated in basal non-stressed conditions^32^ and reported to be required to respond to acidic stress^33^. RsbS has one conserved phosphorylatable serine residue (S56), proposed to be phosphorylated only upon stress^15,18,22^. Despite *L. monocytogenes* having the conserved phosphorylatable residues in RsbR1 and RsbS, as in the respective *B. subtilis* orthologues, their phosphorylation dynamics in vivo remains unknown. Only a phosphoproteomic study, performed in unstressed *L. monocytogenes* cells, reported the identification of a RsbR1 peptide containing the phosphorylated T175 ~ P residue^32^. However, no further studies on the kinase involved or the changes occurring under stress, were reported.

To determine in vivo the phosphorylation dynamics of the *L. monocytogenes* stressosome proteins, we used two types of mutants: one harbouring a predicted kinase-inactive RsbT-N49A variant and, the other expressing a RsbR1-T175A variant, therefore not phosphorylatable at residue 175. To differentiate the different phosphorylated isoforms, we exploited the Phos-Tag system based on phosphate-Zn^2+^ ion sequestration that results in distinct electrophoretic mobility for the distinct isoforms (see “Methods”). To avoid any putative undesirable phosphorylation event due to stress during sample processing (for example SigB activation by centrifugation is known to occur^34^) the bacterial cultures were in all assays heat-inactivated at the time of collection and azide added before the first centrifugation step. In other controls, bacteria were fixed with paraformaldehyde before cell disruption. No changes in the phosphorylation patterns were observed when applying this additional control (Supplementary Fig. 4).

**Figure 4:**
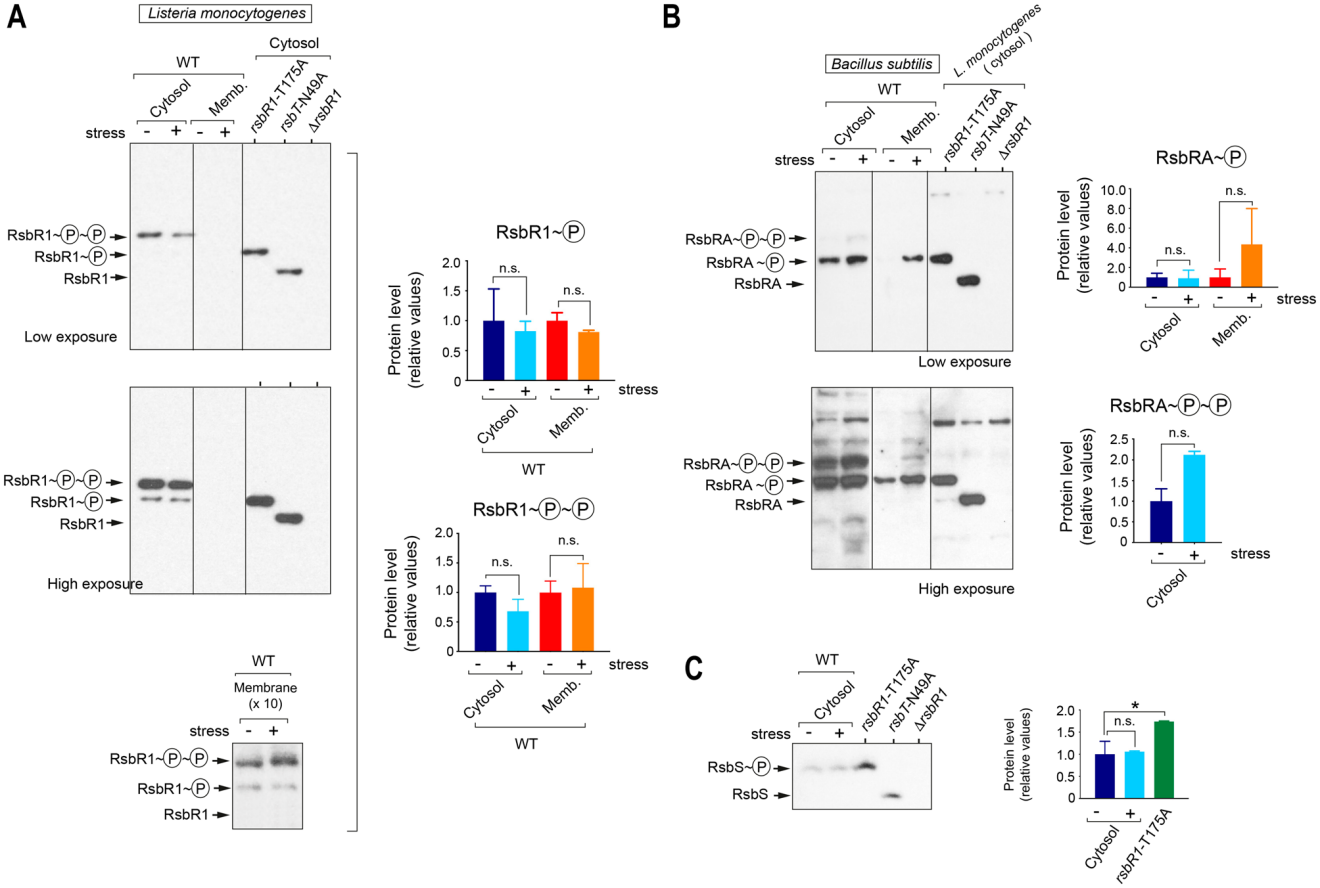
RsbR1 is phosphorylated at large extent by *L. monocytogenes*, regardless of osmotic stress. (**A**) Distinct phosphorylated forms of RsbR1 identified by the Phos-Tag system in *L. monocytogenes* EGD-e subcellular extracts –cytosol/membrane- and in response to 0.5 M NaCl osmolarity stress. Control samples from strains with the phosphorylatable site T175 mutated (T175A) or with a non-functional RsbT kinase (N49A variant), are included. Note the predominance of the doubly-phosphorylated RsbR1 form in the cytosol and the no detection of membrane-bound RsbR1 with this number of bacteria (5 × 10^7^). Two different exposition times of the western blot are shown. Phosphorylated RsbR1 was detected in membrane fractions of more concentrated membrane samples (10×), corresponding to 5 × 10^8^ bacteria. Note the similar pattern obtained in these membrane fractions compared to the cytosolic fractions. (**B**) Phosphorylation pattern of *B. subtilis* RsbRA exposed to the 0.5 M NaCl osmolarity stress. Two different exposition times are shown. (**C**) Phosphorylation pattern of *L. monocytogenes* RsbS in absence or presence of stress. Strains lacking a functional RsbT kinase (N49A) and the polar Δ*rsbR1* mutant were included as controls. Quantification of the signals obtained in three independent biological replicates is shown as mean and standard deviation of a total of three biological replicates. *P < 0.05; n.s.: not significant (one-way ANOVA with Tukey’s multiple comparisons test). Vertical lines within blots indicate separate parts that were grouped from different positions of the same blot. See Supplementary Fig. S6 for images of full blots.

Subcellular fractions were then prepared from *L. monocytogenes* cells growing exponentially in BHI medium in the absence or presence of 0.5 M NaCl. The Phos-Tag method revealed that most RsbR1 present in the cytosol is doubly phosphorylated, with only ~ 5% of the total accounting for the mono-phosphorylated isoform (Fig. 4A). Surprisingly, densitometry of the bands detected in the Western blots showed that the osmotic stress has no effect on the phosphorylation pattern of cytosolic RsbR1, with no switch of the mono-phosphorylated pool to the doubly phosphorylated one in stressed cells (Fig. 4A). Conservation of the phosphorylation pattern following osmotic stress also occurred for membrane-associated RsbR1, detected in this location when using more concentrated membrane samples (10×) (Fig. 4A). This membrane RsbR1 pool was estimated in ~ 5% of the amount detected in the cytosol. Importantly, membrane-associated RsbR1 was also predominantly in the double-phosphorylated form (Fig. 4A). Consistent with the high level of phosphorylation found for RsbR1, even in the basal unstressed condition, the RsbR1-T175A variant was detected in the mono-phosphorylated state, presumably phosphorylated at residue T209 (Fig. 4A). In the kinase-inactive RsbT-N49A mutant, the entire RsbR1 pool was detected unphosphorylated (Fig. 4A), providing, to our knowledge, the first in vivo evidence for the kinase activity of *L. monocytogenes* RsbT over its substrate RsbR1.

We next evaluated whether the high phosphorylation rate observed in *L. monocytogenes* RsbR1 was also noticeable in its *B. subtilis* paralogue RsbRA. We prepared cytosolic and membrane fractions of *B. subtilis* growing exponentially in LB medium and the absence/presence of 0.5 M NaCl. These fractions were analysed for the presence of RsbRA using the Phos-Tag system. Unlike the case in *L. monocytogenes*, the predominant isoform detected for *B. subtilis* RsbRA was the mono-phosphorylated, in both the cytosol and the membrane fractions (Fig. 4B). In contrast to the most accepted model of stressosome function, no double-phosphorylated RsbRA was detected in the *B. subtilis* membrane, even when cells were subjected to osmotic stress conditions (Fig. 4B). The data obtained with *B. subtilis*, in which the monophosphorylated form predominated, provided an additional control for the lack of phosphorylation events taking place after bacteria were collected for sample processing. The Phos-Tag system also showed that *L. monocytogenes* RsbS is fully (mono)-phosphorylated, irrespective of stress. This evidence was reached after comparing the electrophoretic mobility of RsbS in the wild-type versus the *rsbT*-N49A kinase-inactive mutant (Fig. 4C). The levels of RsbS were also higher in the *rsbR1-*T175A mutant respect wild type bacteria (Fig. 4C). Altogether, these data confirmed a high basal kinase activity of *L. monocytogenes* RsbT in both the cytosol and the membrane over its substrates RsbR1 and RsbS, irrespective of the prevailing osmotic stress conditions.

### RsbR1 phosphorylation and RsbR1 paralogues act antagonistically in SigB activation

**Figure 5:**
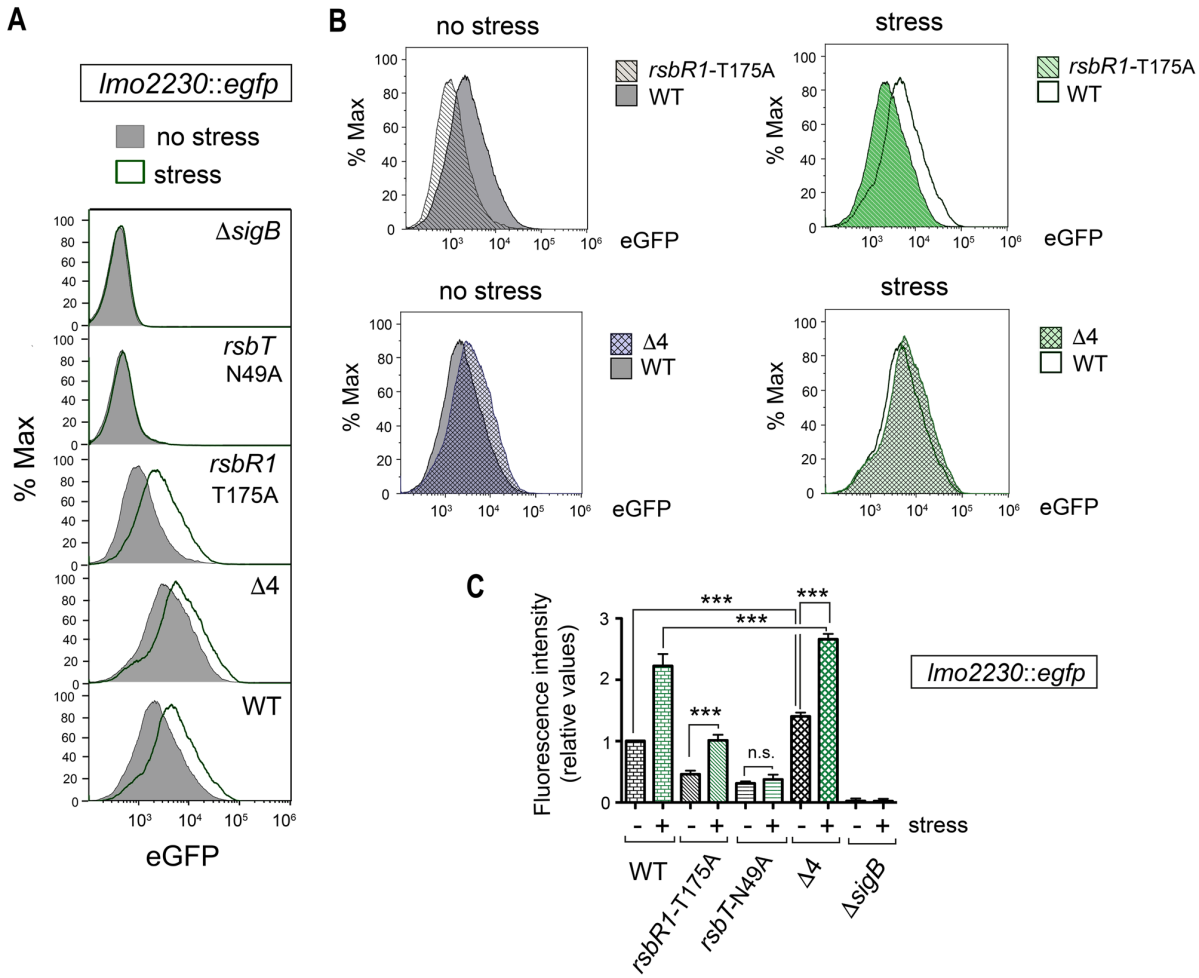
The kinase activity of RsbT is essential for SigB activation with RsbR1 paralogues antagonising such activity in unstressed and stressed *L. monocytogenes*. (**A**) Expression of the P_*lmo2230*_*::egfp* reporter fusion as proxy of SigB activity in the indicated strains in no stress and stress (30 min, 0.5 M NaCl) conditions. Shown is the fluorescence intensity registered in the bacterial population by flow cytometry. (**B**) Side-to-side comparison of SigB activity in wild type compared to the mutant lacking the first phosphorylatable threonine residue, T175, and the Δ4 mutant lacking functional RsbR1 paralogues. Data shown in panels (**A**,**B**) are representative of a total of six samples (3 biological replicates with two technical replicates each). (**C**) Mean fluorescence values of the expression data depicted in panel A. Data shown are the mean and standard deviation of a total of six samples, corresponding to three biological replicates with two technical replicates each. ***, *P* ≤ 0.005; n. s., not significant (one-way ANOVA with Bonferroni’s multiple comparisons test).

Knowing the distinct phosphorylation pattern of RsbR1 in wild-type *L. monocytogenes* compared to those of the *rsbR1*-T175A and *rsbT*-N49A mutant strains (Fig. 4), we next sought to determine whether SigB activity correlates with the phosphorylation status of the stressosome proteins. To assess this, we introduced the P_*lmo2230*_::*egfp* reporter fusion in a series of isogenic wild-type, Δ*sigB*, *rsbR1*-T175A, *rsbT*-N49A and Δ4 mutant strains, this latter strain lacking functional RsbR1 paralogues^29^. Cytometry assays allowed us to monitor SigB activity at the population level (Fig. 5A,B). The kinase-inactive *rsbT*-N49A mutant exhibited residual SigB activity above the Δ*sigB* mutant but had lost responsiveness to the osmotic stress (Fig. 5A, C). In contrast, the *rsbR1*-T175A mutant was able to respond to salt stress with increased SigB activity, although not reaching the level detected in wild-type bacteria (Fig. 5B,C). This result highlighted the need for RsbT-mediated phosphorylation to transduce osmotic stress signals but suggested that RsbR1-T175 was partially dispensable for signal transduction, in contrast to previous observations reported for the response to acid stress^33^.

Interestingly, unlike the case of the *rsbR1*-T175A and *rsbT*-N49A mutants, we detected slightly increased SigB activity in the Δ4 mutant compared to wild type bacteria in the absence or presence of stress (Fig. 5B,C), changes that were statistically significant when measuring mean fluorescence intensity of the bacterial populations (Fig. 5C). These observations suggested that RsbR1 has the capacity to form a functional stressosome independently of the RsbR1 paralogues and further suggested that the RsbR1 paralogues might attenuate the downstream signalling that leads to SigB activation.

### Distinct interactions in the cytosol and membrane involving *L. monocytogenes* stressosome proteins

**Figure 6:**
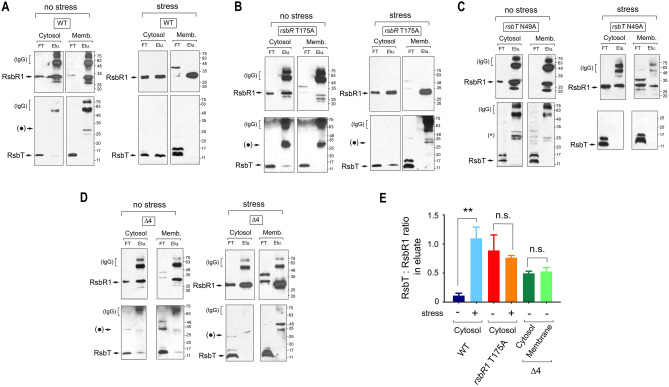
Analysis of *L. monocytogenes* RsbR1-RsbT interaction and action of RsbR1 paralogues in unstressed and stressed cells. Levels of interacting RsbR1 and RsbT detected in fractions of cytosol and membrane (25-fold concentrated versus cytosol) prepared from unstressed and stressed (30 min, 0.5 M NaCl) cells after pull-down using anti-RsbR1 antibodies coupled to magnetic beads. Strains analysed: (**A**) wild-type; (**B**) *rsbR1*-T715A; (**C**) *rsbT*-N49A, and (**D**) the Δ4 mutant lacking functional RsbR1 paralogues. The blots shown correspond to a representative experiment of a total of three biological replicates. Bands indicated as ( • ) correspond to unspecific protein recognition. Molecular weight markers are indicated at the right side of each image. (**E**) Quantification of the RsbT:RsbR1 in eluate samples, which provides an estimation of the amount of RsbT:RsbR1 complex present in the sample. Note that RsbT signal in eluate was only evident in cytosol samples (unstressed, stressed) and in cytosol/membrane eluates of the Δ4 mutant. Data shown are mean and standard deviation of a total of three biological replicates. **, *P* < 0.005, n.s. = not significant (one-way ANOVA with Tukey’s multiple comparisons test). FT, flow-trough; Elu., eluate. See Supplementary Fig. S7 for images of full blots.

In contrast to current accepted models, the above data suggested that *L. monocytogenes* RsbR1 is mostly cytosolic and fully phosphorylated in actively growing cells not subjected to stress (Figs. 3, 4). We then sought to identify whether RsbR1 could have distinct interactions with RsbT in the cytosol and/or the membrane. RsbR1-RsbT interaction was examined by immunoprecipitation assays in cytosol and membrane fractions prepared from four different strains: wild type, *rsbR1*-T175A, *rsbT*-N49A and Δ4. Using anti-RsbR1 antibodies coupled to magnetic beads, it was possible to co-immunoprecipitate RsbT from the cytosol but not the membrane of wild-type bacteria (Fig. 6A). The cytosolic RsbR1-RsbT interaction was detected in the *rsbR1*-T175A mutant (Fig. 6B) but not in the kinase-inactive *rsbT*-N49A mutant (Fig. 6C). Interestingly, the lack of functional RsbR1 paralogues in the Δ4 mutant resulted in the detection of a stable RsbR1-RsbT interaction in the membrane extract that was not visualized in the corresponding extract of wild-type bacteria (Fig. 6A,D). This result opened the possibility of RsbR1 paralogues associated to the membrane and competing with RsbR1 for the interaction with RsbT. This competition could modulate negatively the assembly of functional stressosome complexes and, consequently regulate the strength of the response.

The RsbR1-RsbT interaction was also analysed in bacteria responding to osmotic stress. In stressed cells, the amounts of RsbR1/RsbT complexes present in the cytosol increased significantly in wild-type bacteria (Fig. 6A,E). Notably, the exposure to stress led to loss of RsbR1-RsbT complexes in the cytosol and membrane fractions of the Δ4 mutant lacking functional paralogues (Fig. 6D). This result supported the idea of RsbR1 paralogues attenuating the stress response by probably reducing the number of active RsbR1-RsbS-RsbT stressosomes, from which RsbT would be rapidly released in the presence of the stress signal.

The idea of RsbR1 paralogues associated with the membrane was further tested by a proteomics approach in unstressed bacteria. We reasoned that in the absence of stress, in which a reduced but basal SigB activation is still detected (Fig. 2B), the competition of RsbR1 paralogues with RsbR1 for forming an active stressosome complex could be more evident at the membrane level. The immunoprecipitated material obtained using anti-RsbR1 antibodies from cytosol and membrane fractions of unstressed wild-type bacteria was processed for high resolution liquid chromatography-tandem mass spectrometry. The proteomic analysis, performed in three independent biological replicates, revealed the presence in the membrane of the RsbR1 paralogues RsbR2, RsbR3 and RsbL (Table 1). These results were in concordance with previous reports showing immunoprecipitation of these three RsbR1 paralogues, as well as RsbS, by pulling-down Prli42^15^. Importantly, none of these RsbR1 paralogues were pulled down from the cytosol of wild type bacteria (Table 1) despite the much larger amount of RsbR1 detected in the cytosol by immunoblot assays (see Fig. 3A). These findings supported a stable interaction of RsbR1 with RsbS and the RsbR1 paralogues only in association to the membrane. Globally, these data allowed us to conclude the presence in actively growing *L. monocytogenes* cells of membrane-associated protein heterocomplexes in which RsbR1 paralogues could modulate the formation and/or stability of the RsbR1-RsbS-RsbT complex characterized in in vitro studies.

**Table 1:** *L. monocytogenes* stressosome proteins interacting with RsbR1 detected in cytosol and membrane fractions by high resolution MS.

Stressosome-related protein	Mass (Da)	Wild type^a^						Δ*rsbR1*^b^			
		Cytosol			Membrane			Cytosol		Membrane	
		Unique peptides (coverage, %)			Unique peptides (coverage, %)			Unique peptides (coverage, %)		Unique peptides (coverage, %)	
		Exp. 1	Exp. 2	Exp. 3	Exp. 1	Exp. 2	Exp. 3	Exp. 1	Exp. 2	Exp. 1	Exp. 2
RsbR1 (Lmo0889)	31,620	19 (74.0)	19 (74.0)	27 (78.0)	13 (55.0)	11 (44.0)	25 (74.0)	2 (10.0)	1 (4.3)	1 (4.0)	1 (4.3)
RsbS (Lmo0890)	12,596	–	–	–	–	–	3 (20.0)	–	–	–	–
RsbT (Lmo0891)	14,727	–	–	–	–	–	–	–	–	–	–
RsbR2 (Lmo0161)	31,737	–	–	–	1 (2.9)	2 (8.3)	12 (49.2)	1 (5.1)	–	–	–
RsbL (Lmo0799)	28,816	–	–	–	6 (33.0)	3 (16.0)	11 (56.1)	1 (3.6)	1 (5.5)	–	–
RsbR3 (Lmo1642)	30,414	–	–	–	–	–	11 (50.2)	–	–	–	–
RsbR4 (Lmo1842)	31,516	–	–	–	–	–	–	–	–	–	–

Complete lists of proteins identified in each of the three independent experiments performed are included in Supplementary information.

– not detected.

^a^Examples of fragmentation spectra of high confidence for each of these proteins are shown in Supplementary Fig. S8.

^b^These peptides were detected with low quality (see example in Supplementary Fig. S9).

## Discussion

In this study, we have examined in live *L. monocytogenes* cells the subcellular location and interaction dynamics of the core stressosome proteins RsbR1 and RsbS and the kinase RsbT. These three proteins have been extensively studied in vitro mainly in structural analyses in which purified proteins were combined and assembled into highly ordered macromolecular complexes^15,16^. To date, it is widely accepted that the RsbR1-RsbS-RsbT complex that adopts an icosahedral structure is the entity perceiving the external stress signal in live *L. monocytogenes* cells^16,17^. Integration of stress signals is thought to trigger phosphorylation at a second site (T209) in mono-phosphorylated RsbR1 (T175 ~ P) and the single phosphorylatable residue present in RsbS (S56), events that could occur preferentially at the membrane level due to the tethering role assigned to the Prli42 miniprotein^16^. Our in vivo findings in live *L. monocytogenes* cells, however, differ somewhat from this view since we were unable to detect such stable RsbR1-RsbS-RsbT complex associated with the membrane. Instead, we identified a minimal membrane-associated RsbR1-RsbS complex interacting with at least three RsbR1 paralogues, RsbR2, RsbR3 and RsbL; and, in which RsbT was not detected (Table 1). The absence of RsbT from this membrane-associated complex is consistent with the high phosphorylation level detected for RsbR1 (Fig. 4A), the lack of RsbT in RsbR1-immuno-precipitates prepared from membrane fractions (Fig. 6A) and, as reported by others^15^, the absence of RsbT in anti-Prli42 immuno-precipitates. Nonetheless, our immunoblot assays identified a RsbR1-RsbT interaction in the cytosol that was not reflected in the proteomic approach. RsbT is a small protein (~ 15 kDa), which limits the detection of tryptic peptides. It is also possible that only a small fraction of the immuno-precipitated cytosolic RsbR1 may establish a stable interaction with RsbT. This low abundance of interacting RsbT is another factor that might have limited its identification in membrane fractions by proteomic approaches.

On the other hand, the failure to detect a stable RsbR1-RsbT interaction in the *L. monocytogenes* membrane agrees with a previous report in *B. subtilis* based on immunoprecipitation assays using anti-RsbRA antibodies. This study, performed in total cell extracts, showed the predominance of RsbRA-RbsS complexes lacking RsbT^35^. Overall, our in vivo findings and those collected in *B. subtilis* support a short half-life for the RsbR1-RsbS-RsbT complex in live cells, in contrast to the stable complexes that are assembled in vitro with purified proteins. The transient nature of this stressosome could also explain why, in cells exposed to osmotic stress, the majority of complexes containing RsbR1 and RsbT are located in the cytosol (Fig. 6A). This finding opens the possibility of “cytosolic” stressosome complexes assembled with fully phosphorylated RsbR1/RsbS and stable RsbR1-RsbT interactions (Fig. 6A).

**Figure 7:**
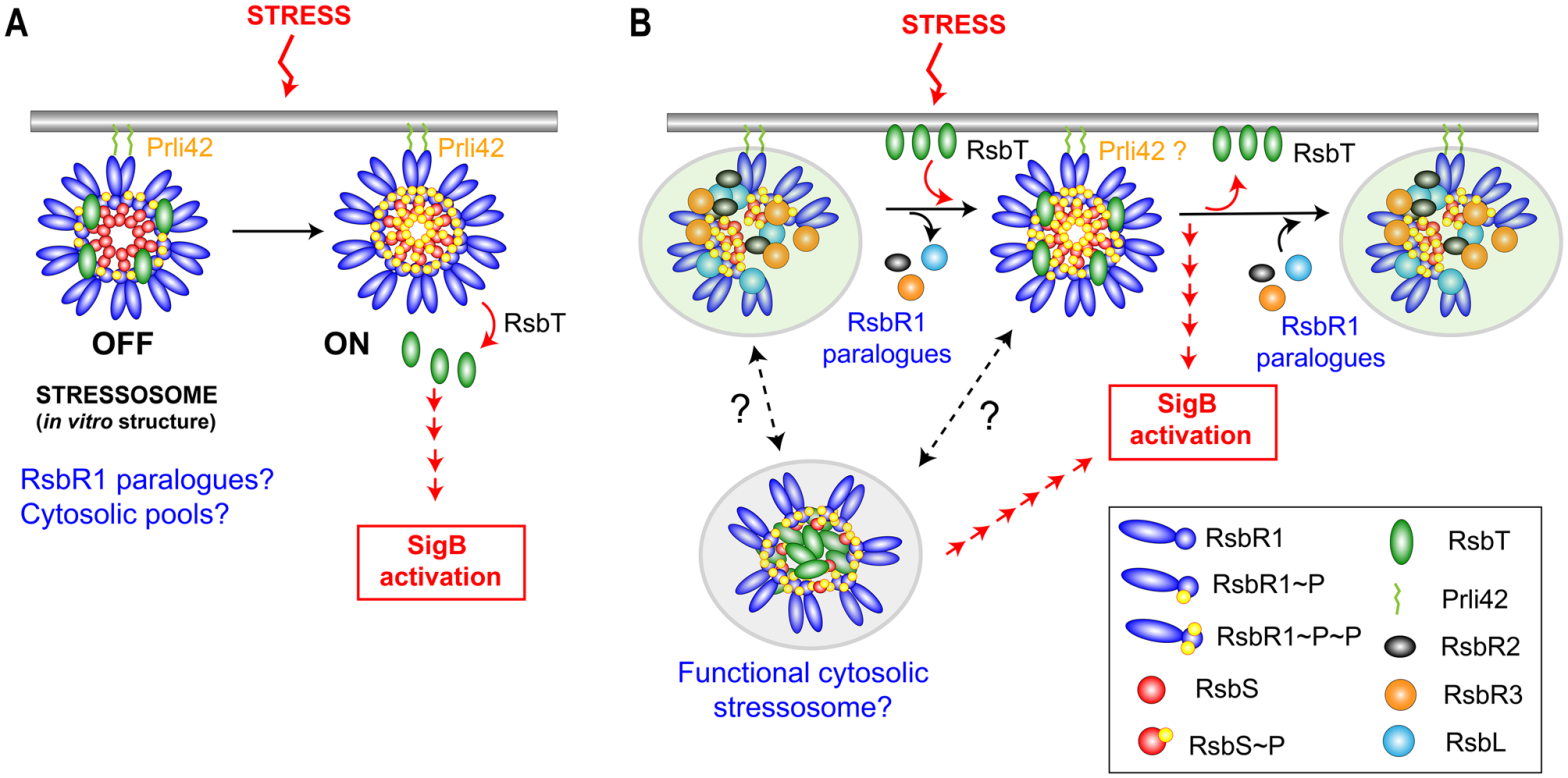
Tentative model depicting the dynamics of interaction among the stressosome proteins analysed in this study. (**A**) Model proposed for stress response based on crystallographic data obtained in vitro with purified proteins in *L. monocytogenes*^16^; (**B**) Model integrating the observations obtained in vivo from *L. monocytogenes* about the distribution and interaction of distinct stressosome proteins. Note the putative negative role assigned to RsbR1 paralogues to prevent the formation of a stable RsbR1-RsbS-RsbT complex, which could be only transiently formed upon stress. The association of RsbT with the membrane is depicted as a direct interaction, although it could be indirect since it lacks defined hydrophobic domains. The model also highlights the presence of a large pools of cytosolic RsbR1, RsbS and RsbT, with the possibility of functional stressosomes in this location capable of activating SigB. See text for details.

Moreover, our data showed that, in osmotic stress conditions and the absence of RsbR1 paralogues, the RsbR1-RsbT interaction becomes undetectable probably due to the exacerbated release of RsbT and consistent with the slightly higher SigB activity displayed by the Δ4 mutant (Fig. 5). Based on these observations, we propose a model that allows for distinct stressosome configurations, all sharing the presence of fully phosphorylated RsbR1 and RsbS but with differences in other components such as RsbR1 paralogues, the kinase RsbT or, the association (or not) with a miniprotein (Fig. 7). This tentative model establishes a putative negative role of at least three of the four known RsbR1 paralogues in modulating the in vivo assembly of the membrane-associated stressosome but, based on our proteomic data, with probably no structural or regulatory role in the assembly of the hypothetical cytosolic stressosome containing phosphorylated RsbR1/RsbS. Except for RsbL, which is involved in the response to blue light^26^, no other functions were previously known for the other RsbR1 paralogues, RsbR2 and RsbR3^26,36–39^.

Our study also provides insights into the distribution of stressosome proteins in subcellular compartments. The vast majority of RsbR1 and RsbS was detected in the cytosol and no changes in these protein pools were observed under stress. This may indicate that, in the nutrient-rich medium used in laboratory conditions, *L. monocytogenes* produces an excess of stressosome proteins compared to what it is needed to cope with at least the type of stress tested, hyperosmolarity. It would be of interest in the future to monitor whether a proportion of these cytosolic pools mobilize to the membrane in response to other stresses or during the interaction of *L. monocytogenes* with the mammalian host or, as above postulated, contribute to the assembly of functional stressosomes in this subcellular location. These are questions that we aim to address in future work.

One of the most puzzling observations of our study was the predominance of highly phosphorylated forms of RsbR1 and RsbS, even in actively growing *L. monocytogenes* not subjected to osmotic stress. Moreover, the assays performed with control mutant strains harbouring a mutation in the first phosphorylatable site of RsbR1 (T175) or a kinase-inactive RsbT variant, demonstrated that most RsbR1 molecules are doubly phosphorylated and that RsbS is also fully phosphorylated, irrespective of exposure to osmotic stress. Importantly, although we observed that RsbT-mediated phosphorylation is absolutely required for SigB activation by osmotic stress, our findings in live *L. monocytogenes* contradict the view of phosphorylation events occurring on RsbR1 and RsbS concomitantly to stress perception. Interestingly, the kinase-inactive RsbT-N49A variant loses the capacity to interact with RsbR1 regardless of stress and is unable to activate SigB (Figs. 5, 6). So, the release of RsbT from the stressosome seems not to be per se sufficient to activate SigB. As described for RsbT in *Vibrio brasiliensis*^40^, the possibility that *L. monocytogenes* RsbT has autophosphorylation activity cannot be discarded, which could be a requisite to activate RsbU and, as a result, the SigB cascade. Future studies should certainly test this tempting hypothesis.

It is also relevant to note that phosphorylation at the T175 residue of *L. monocytogenes* RsbR1 is not absolutely required to respond to stress, to interact with RsbT in the cytosol (Fig. 6B), or to become phosphorylated at the second phosphorylatable site, the T209 residue (Fig. 4A). Nonetheless, the lack of RsbR1 phosphorylation at T175 decreases the intensity of the SigB response, suggesting that this phosphorylation might stabilize at a larger extent the functional RsbR1-RsbS stressosome once RsbT is released. It is important to highlight that, unlike what we observe in *L. monocytogenes*, lack of phosphorylation at the first phosphorylatable residue of *B. subtilis* RsbRA (T171) impairs the stress response^18^ and that a recent study claimed phosphorylation of T175 as relevant for *L. monocytogenes* to cope with acid stress^33^. Interestingly, the loss of RsbT kinase activity in *L. monocytogenes* renders RsbR1 less stable in no stress and stress conditions (Supplementary Fig. S5). This lower amount of RsbR1 can also affect the capacity of the cell to activate SigB.

An additional difference observed between *L. monocytogenes* RsbR1 and *B. subtilis* RsbRA was the phosphorylation state in basal conditions. Most of the former existed as doubly-phosphorylated isoform whereas the second most common one was the mono-phosphorylated. We are still uncertain about the basis of this difference. However, the fact that we did not detect a predominance of doubly-phosphorylated RsbRA in *B. subtilis*, even in stress conditions, allowed us to discard potential artefacts during sample processing linked to unspecific increased phosphorylation. In this context, it is noteworthy that the study of Eymann et al. in *B. subtilis* concluded that phosphorylation of the RsbRA T205 residue could contribute as a feedback mechanism to limit SigB activation, together with the phosphatase activity of RsbX^22^. As an alternative interpretation, the possibility that the stressosome could be tuned down in *L. monocytogenes* in basal conditions by an excess of RsbR1 phosphorylated at T209 should therefore not be discarded. Nonetheless, T209 (T205 in *B. subtilis*) phosphorylation has been proposed to be linked to extreme stress in order to limit SigB signalling^16,22^, a condition that we think is unlikely to be present in actively growing *L. monocytogenes* cells.

As we propose in the model depicted in Fig. 7, stimulation of the downstream signalling cascade that activates *L. monocytogenes* SigB might be modulated by cytosolic and membrane-associated complexes responding to distinct environmental stimuli. The dynamics of assembly and disassembly of these stressosome complexes could be fine-tuned by RsbR1 paralogues, especially at the membrane, making these paralogues a new regulatory element controlling stressosome function.

## Methods

### Bacterial strains, plasmids and primers

*Listeria monocytogenes* EGD-e, *E. coli* and *B. subtilis* BG214 strains and plasmids and the primers used for this study are listed in supplementary information, Tables S1 and S2, respectively.

### Construction of genetically modified *L. monocytogenes*

The genes *rsbR1* and *rsbT* were mutated to incorporate Thr-to-Ala in the codon 175 and Asn-to-Ala in the codon 49, respectively. The codon 175 ACA (Thr) in *rsbR1* was changed to GCG (Ala) and the codon 49 AAT (Asn) in *rsbT* was changed to GCT (Ala). In both cases silent mutations were added the two adjunct codons in order to discern mutant from WT codons by PCR during mutagenesis (*rsbR1* WT -ATTGACACAGAAAGA- sequence mutated to *rsbR1* T175A -ATAGATGCGGAGCGT- and *rsbT* WT -GCTAGAAATATTTTC- to *rsbT* N49A -GCACGTGCTATCTTT-). The mutagenic sequences, each with a total length of 612 bp, including EcoRI and BamHI restriction sites at each end, were artificially synthetized in the vectors pEX-A128::*rsbR1* (T175A) and pEX-K168::*rsbT* (N49A) (Eurofins Genomics). The mutagenic sequences were subsequently cloned into shuttle vector pMAD, originating pMAD::*rsbR1* (T175A) and pMAD::*rsbT* (N49A). *L. monocytogenes* electrocompetent cells were created as previously described^41^. The electrocompetent *L. monocytogenes* WT was separately transformed with pMAD::*rsbR1* (T175A) and pMAD::*rsbT* (N49A). The *L. monocytogenes rsbL* (C56A) mutant strain was transformed with pMAD::∆*lmo1842*. Electroporated cells were plated in BHI supplemented with erythromycin (Ery). Chromosomal integration and subsequent excision were achieved through a two-step recombination as previously described^38^. cPCR with the primers *rsbR1*_upflank_F paired with either *rsbR1* (T175A)_R and *rsbT* (N49A)_R were used to identify the chromosomal mutation in *rsbR1* and *rsbT*, respectively, while the deletion of *lmo1842* was verified with primers *lmo1842*_flank_F and *lmo1842*_flank_R. To construct the *L. monocytogenes* EGD-e quadruple-mutant (*rsbL* (C56A); ∆*lmo1842*; ∆*lmo1642*; ∆*lmo0161*), the double mutant *L. monocytogenes* EGD-e (*rsbL* (C56A); ∆*lmo1842*) was sequentially mutated using the mutagenic vectors pMAD::∆*lmo1642* and pMAD::∆*lmo0161*. The primers pair *lmo1642_flank*_F; *lmo1642_*flank_R and *lmo0161*_flank_F; *lmo0161*_flank_R, were used to verify the chromosomal deletion of each respective gene. The SigB-eGFP reporter vector pKSV7-P_*lmo2230*_::*egfp* was transformed in each mutant strain as previously described^29^. The chromosomal integration of the SigB-eGFP reporter occurred upstream of the original *lmo2230* promoter region by homologous recombination resulting in a duplication of the promoter region. Integration was verified by PCR (using primers *eGFP-lmo2230*-F integration and *eGFP-lmo2230*-R integration). All genomic DNA from each mutant strain were whole genome-sequenced to confirm the mutation and identify secondary mutations.

### Hyperosmotic shock growth conditions

*Listeria monocytogenes* EGD-e and *B. subtilis* BG214 strains were cultured in brain heart infusion, BHI (Becton Dickinson, BD) and Luria–Bertani, LB, broth, respectively, with continuous shaking at 37 °C until reaching mid-exponential phase (OD_600nm_ = 0.4) (no stress). Salt (0.5 M NaCl in the form of solid salt crystals) was added to the BHI medium for 30 min (osmotic stress). Around 3 × 10^10^ bacteria (50 mL culture) were spun down by centrifugation (10,000 × *g*, 18 min, 4 °C) and washed twice in phosphate buffer saline (PBS), pH 7.4. The pellet was kept at − 80 °C until being subjected to subcellular fractionation.

The same method was performed in experiments involving addition of 100 μg/mL chloramphenicol to inhibit protein synthesis in no stress and hyperosmotic conditions.

### Subcellular fractionation and Western blot analysis of stressosome machinery-related proteins in *L. monocytogenes*

Fractions containing cytosolic and membrane proteins from exponential phase growing bacteria were obtained as described previously^42^. The pellet of bacteria was washed in 10 mL of TS buffer (10 mM Tris HCl pH 6.9, 10 mM MgCl_2_, 0.5 M sucrose) and centrifuged at 10,000 × *g*, 18 min, 4 °C. Then, the pellet was resuspended in a lysis solution: TS buffer containing 60 μg/mL mutanolysin from *Streptomyces globisporus* (ATCC 21553, Sigma-Aldrich), 250 μg/mL RNAse A, protease inhibitor cocktail and incubated during 5 h at 37 °C with slow-rotation agitation. Protoplasts were recovered by centrifugation at 15,000 × *g*, 10 min, 4 °C. The supernatant corresponding to the cell wall fraction was discarded and the pellet containing the protoplasts was washed with 1 mL of PBS, pH 7.4 and centrifuged at 10,000 × *g*, 18 min, 4 °C. The protoplasts were resuspended into 400 μL of PBS, pH 7.4, 1 μg/mL DNAse A, protease inhibitor cocktail and they were lysed by low power sonication. Unbroken cells were removed by centrifugation at 20,000 × *g*, 10 min, 4 °C and the supernatant was subjected to ultracentrifugation at 100,000 × *g*, 1 h, 4 °C to separate cytosol and membrane fractions. The pellet containing membrane proteins was washed with PBS, pH 7.4 by centrifugation at 100,000 × *g*, 1 h, 4 °C. The pellet was resuspended into 400 μL of PBS, pH 7.4 to get a membrane extract containing the same number of bacteria as in the cytosolic fraction. To obtain tenfold (10 ×) concentrated membrane extracts, subcellular fractionation was performed from twice as many *L. monocytogenes* cells and the pellet containing membrane proteins was resuspended into 80 μL of PBS, pH 7.4. A volume of 10 µL of cytosolic and membrane fractions was analyzed by SDS-PAGE and Western-blot using affinity purified polyclonal rabbit antibodies against stressosome machinery-related proteins (RsbR1, RsbT, RsbS) and SigB. Protein levels detected by western blot from three independent experiments were quantified with ImageJ and statistical analysis was undertaken by one-way ANOVA (Tukey’s multiple comparison test).

### Identification of phosphorylated isoforms of RsbR1 and RsbS with Phos-Tag system based on phosphate-Zn^2+^ ion sequestration

*Listeria monocytogenes* and *B. subtilis* cultures were boiled for 20 s at 100 °C to stop the metabolism before harvesting the cells by centrifugation. The cells were washed twice with a killing buffer containing 20 mM Tris pH 7.5, 5 mM MgCl_2_, 20 mM NaN_3_ (10,000 × *g*, 18 min, 4 °C)^22^. The pellet was subjected to subcellular fractionation as previously described. An additional control of sample preparation consisted in fixing *L. monocytogenes* cells with 4% (w/v) paraformaldehyde before subcellular fractionation.

Phos-tag electrophoresis based on phosphate-Zn^2+^ ion sequestration (SuperSep Phos-tag precast gels) was used to separate the phosphorylated isoforms of RsbR1 and RsbS. The Phos-tag polyacrylamide gel contains zinc ions that selectively bind to the phosphate ions and the complex is stable at neutral pH. The migration speed of phosphorylated proteins decreases due to the binding of the metallic ion and phosphorylated/non-phosphorylated proteins are separated as different bands.

### Purification of His-tagged RsbR1 protein by immobilized metal affinity purification (IMAC)

His-tagged RsbR1 was expressed in *E. coli* BL21 (DE3) Gold competent cells (Novagen). Bacteria were grown overnight at 16 °C in LB broth (100 mL) and protein expression was induced during 4 h with 1 mM IPTG after reaching an OD_600_ = 0.2. Bacteria were then spun down (10,000 × *g*, 18 min, 4 °C) and washed with PBS, pH 7.4. Cells were resuspended in 4 mL of binding buffer [50 mM NaH_2_PO_4_, 200 mM NaCl, 5 mM imidazole, 3 μg/mL DNAse A, 100 mM Phenylmethylsulfonylfluoride (PMSF), pH 7.0] and lysed by sonication. The bacterial lysate was separated from cell debris by high-speed centrifugation (13,000 × *g*, 30 min, 4 °C).

Immobilized metal affinity chromatography (IMAC) resin (TALON) was pre-equilibrated by three washes with binding buffer (700 × g, 2 min, RT). The bacterial lysate was incubated with the resin by slow-rotation agitation during 4 h at 4 °C to allow binding of the His-tagged protein to the resin. Incubation was done at 4 °C to prevent proteolysis. The flow-through was collected by centrifugation (700 × *g*, 5 min, 4 °C) and the resin was washed three times with 10 mL of binding buffer (700 × g, 5 min, 4 °C). The purified His-tagged protein was eluted with an imidazole gradient (from 50 to 500 mM). The fractions were analyzed by Coomassie and those containing the purified His-tagged protein (eluted from 150 to 250 mM imidazole) were concentrated to reach a final volume of 500 μL (Millipore Amicon).

The concentrated eluate fraction was dialyzed overnight against 1 L of coupling buffer (100 mM MOPS buffer, pH 7.5) at 4 °C to remove imidazole and incubated with derivatized cross-linked agarose gel bead support (Affigel 10, BioRad) at 4 °C for 4 h with slow-rotation agitation. The purified protein RsbR1 coupling to the resin was cross-linked by adding 100 μL of quenching buffer (1 M ethanolamine pH 8.0) at 4 °C during 1 h. The coupling mixture was washed with 10 mL of binding buffer (700 × g, 5 min, 4 °C) and transferred to a column (TALON, 2 mL gravity columns). The column was washed with PBS, pH 7.4 and was stored at 4 °C in 2 mL of PBS pH 7.4, 0.2% (w/v) sodium azide.

### Antibody purification by antigen-specific affinity

Polyclonal rabbit crude serum anti-RsbR1 (1.5 mL) (Charles River, France) was inactivated at 56 °C for 30 min to destroy complement factors. The serum was spun down (13,000 × *g*, 10 min, 4 °C) and the supernatant was applied three times at 4 °C to the column coupled to purified His-tagged RsbR1 protein. A gauge needle was fitted to slow rate at which the column flows. The column was washed with 10 mL of PBS, pH 7.4 and affinity purified antibodies were eluted with 10 mL of elution buffer (100 mM glycine, pH 2.4). The pH of the elution fractions was immediately neutralized with 1 M Tris pH 8.0 and the fractions concentrated to reach a final volume of 500 μL corresponding to a concentration of antibodies of ~ 1 mg/mL. The purified anti-RsbR1 antibody was stored at − 20 °C in 20% glycerol.

### Purification of the stressosome from cell extracts by pull-down experiments

Wild-type, *rsbR1*-T175A, *rsbT*-N49A, Δ4 and Δ*rsbR1* strains were grown to exponential phase (OD_600nm_ = 0.4) in 300 mL of BHI medium (1.8 × 10^11^ bacteria). The cells were washed three times with PBS, pH 7.4 by centrifugation at 10,000 × *g*, 18 min, 4 °C. Bacteria were disrupted using a French press (three passes) in 10 mL of a buffer containing 50 mM Tris HCl, pH 7.0, 60 μg/mL DNAse A, 2 mM PMSF. Cell debris were removed by centrifugation at 5000 × *g*, 5 min, 4 °C and the supernatant was subjected to ultracentrifugation at 145,000 × *g*, 1 h, 4 °C. The supernatant constituting the cytosolic fraction (10 mL) was kept at − 20 °C and the pellet containing the membrane proteins was incubated with 400 μL of PBS, pH 7.4, 0.1% Triton X-100 during 30 min at 4 °C with slow-rotation agitation. The sample was subjected to ultracentrifugation at 100,000 × *g*, 1 h, 4 °C and supernatant containing membrane proteins and complexes (400 μL) was kept at − 20 °C (25 × concentrated membrane extract compared to the cytosolic fraction).

To immuno-precipitate the stressosome complex, 1.5 mg (50 μL) of Dynabeads protein G (Invitrogen) were incubated with 120 μg of affinity purified rabbit polyclonal anti-RsbR1 antibodies during 15 min at room temperature with slow-rotation agitation. Then, the antibodies were crosslinked to the magnetic beads with 3 mM BS^3^ [bis(sulfosuccinimidyl)suberate] (Thermo Fisher) during 30 min at room temperature. The tube was placed on the magnet to remove the supernatant containing unbound antibodies and crosslinker, and the beads were washed three times with the washing buffer: PBS, pH 7.4, with 0.02% (v/v) Tween 20.

The cytosolic (10 mL) and the 25-fold (25 ×) concentrated membrane (400 μL) fractions were incubated separately with the beads at 4 °C during 5 h with slow-rotation agitation. The supernatant constituting the flow-through was kept for further analysis and the beads were washed three times with the washing buffer. Finally, the beads were incubated with 40 μL of elution buffer (100 mM glycine, 150 mM NaCl, pH 2.4) during 5 min. The supernatant constituting the eluate was transferred to a clean tube and the pH was adjusted by adding 10 μL of 1 M Tris, pH 7.5 to preserve the integrity of the proteins. The pull-down procedure resulted in the obtention of eluates from the cytosolic and membrane extracts 250 × and 10 × concentrated, respectively, compared to their respective flow-through sample. The eluate and flow through samples from the cytosolic and membrane fractions were analyzed by SDS-PAGE and Western blot using affinity purified polyclonal rabbit anti-RsbR1 and anti-RsbT antibodies.

The eluates were also analyzed by Liquid Chromatography-Tandem Mass Spectrometry (LC–MS/MS). The samples were cleaned and concentrated using a concentrator SDS-PAGE gel and one third of each individual lane was in-gel digested using trypsin and reducing conditions. Resulted peptides were dried in a speed vacuum and analyzed by LC–MS, using an Eksigent 1D Plus nanochromatographer and a Sciex 5600 TripleTOF mass spectrophotometer. The gradient time used for peptide identification was 100 min. MS and MS/MS spectra were used to launch a database search using Mascot against the UNIPROT *L. monocytogenes* EGD-e database (entry NC 003210.1).

### Flow cytometry analysis of eGFP-expressing cells

Bacterial strains were grown to OD_600nm_ = 0.4 (no stress) in BHI or BHI supplemented with 0.5 M NaCl during 30 min (stress). Around 3 × 10^8^ bacteria were spun down by centrifugation (10,000 × *g*, 18 min, 4 °C) and washed twice in PBS, pH 7.4. The cells were fixed with 4% (w/v) paraformaldehyde for 15 min at room temperature. Fixed cells were harvested by centrifugation and resuspended in 500 µL of filtered PBS, pH 7.4. Quantification of single cell fluorescence was achieved by flow cytometry with Beckman Coulter GALLIOS Analyzer with 488 nm blue laser excitation and 50,000 events recorded for each sample. The data collected were processed with Kaluza software to plot side and forward scatter values, the percentage of eGFP-positive cells and the mean of fluorescence values. Statistical analysis has been carried out by one-way ANOVA (Bonferroni’s multiple comparison test).

### Statistical analyses and densitometry

Statistical significance was analyzed with GraphPad Prism v8.4.3 software (GraphPad Inc.) using one-way analysis of variance (ANOVA) with Tukey’s or Bonferroni’s multiple comparison tests. A *P* value ≤ 0.05 was considered significant. Densitometry on bands obtained in immunoblot assays was performed using ImageJ, available from the National Institute of Health, USA.

## Supplementary information

Supplementary Information.
