# Supplementary material for: Impact of osmotic stress on the phosphorylation and subcellular location of Listeria monocytogenes stressosome proteins
Source: Sci Rep. 2020 Nov 30;10:20837. doi: 10.1038/s41598-020-77738-z (PMC7705745; doi:10.1038/s41598-020-77738-z)
Supplement: Supplementary file 1 — Supplementary Information. [file 41598_2020_77738_MOESM1_ESM.zip › Dessaux-et-al-Supp-Information/Figures-S1-to-S9-Tables-S1-S2.pdf]

Supplementary information

Impact of osmotic stress on the phosphorylation and subcellular location of *Listeria monocytogenes* stressosome proteins

Charlotte Dessaux, Duarte N. Guerreiro, M. Graciela Pucciarelli, Conor P. O'Byrne, & Francisco García-del Portillo

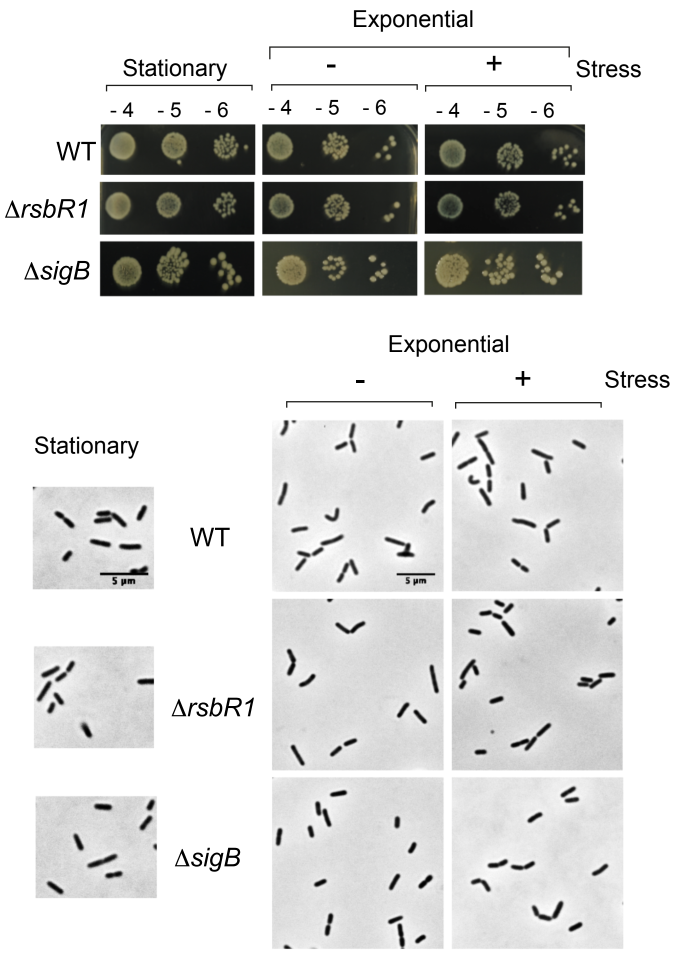

Figure S1. Osmotic stress during growth in BHI medium does not affect *L. monocytogenes* viability or morphology. The EGD-e wild-type strain (WT), Δ*sigB* and Δ*rsbR1* were grown in BHI at 37°C until reaching OD_{600nm} = 0.4 [no stress, (-)] or after the addition of 0.5 M NaCl during 30 min [stress, (+)]. Stationary phase bacteria used to start the cultures were also analysed. Bacterial survival was monitored on BHI-agar plates (A) and the cell morphology with phase-contrast microscopy (B).

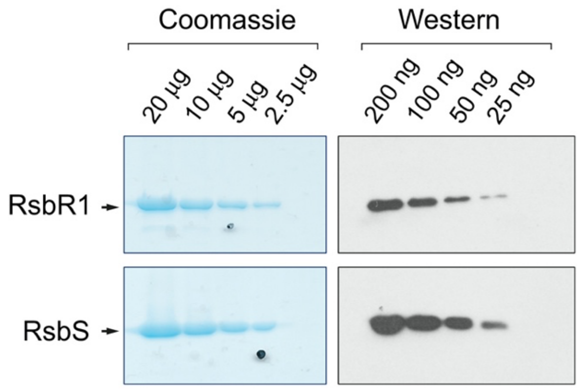

Figure S2. Affinity of the antibodies that were used in this study to recognize RsbR1 and RsbS. Shown in a immunoblot against the indicated amounts of purified protein and equal dilution (1:2,000) of the respective sera.

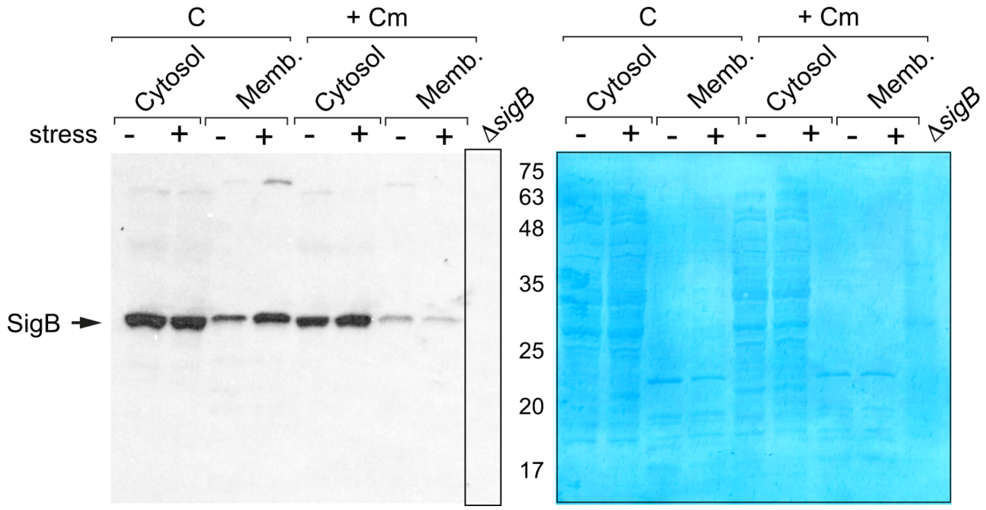

Figure S3. Inhibition of protein synthesis concomitant to osmotic stress decreased the amount of membrane-associated SigB. *L. monocytogenes* wild type strain EGD-e was grown in BHI medium and exposed to 0.5 M NaCl (stress) for 30 min at OD_{600} = 0.4. At that time, parallel cultures of unstressed and stressed were treated with 100 µg/mL chloramphenicol (Cm) to inhibit protein synthesis. Note the decreased in membrane-associated SigB in stressed cells treated with Cm, despite equivalent total protein content evidenced by Coomasssie staining of the membrane used in Western blot (right). Molecular weight markers are indicated. The Δ*sigB* mutant was included as western blot negative control.

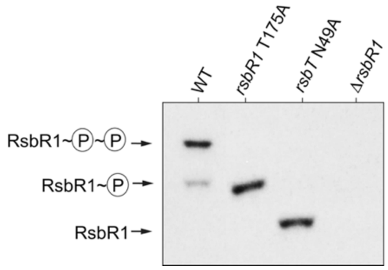

Figure S4. Fixation of the bacteria prior to subcellular fractionation does not alter the phosphorylation pattern of RsbR1 in *L. monocytogenes* wild type strain EGD-e (WT). The phosphorylation pattern was analysed with the Phos-Tag system as described in Methods. Control strains rsbR1 T175A and rsbT N49A were included for comparison. Note that the most abundant RsbR1 form is double phosphorylated, discarding artefactual phosphorylation during sample preparation.

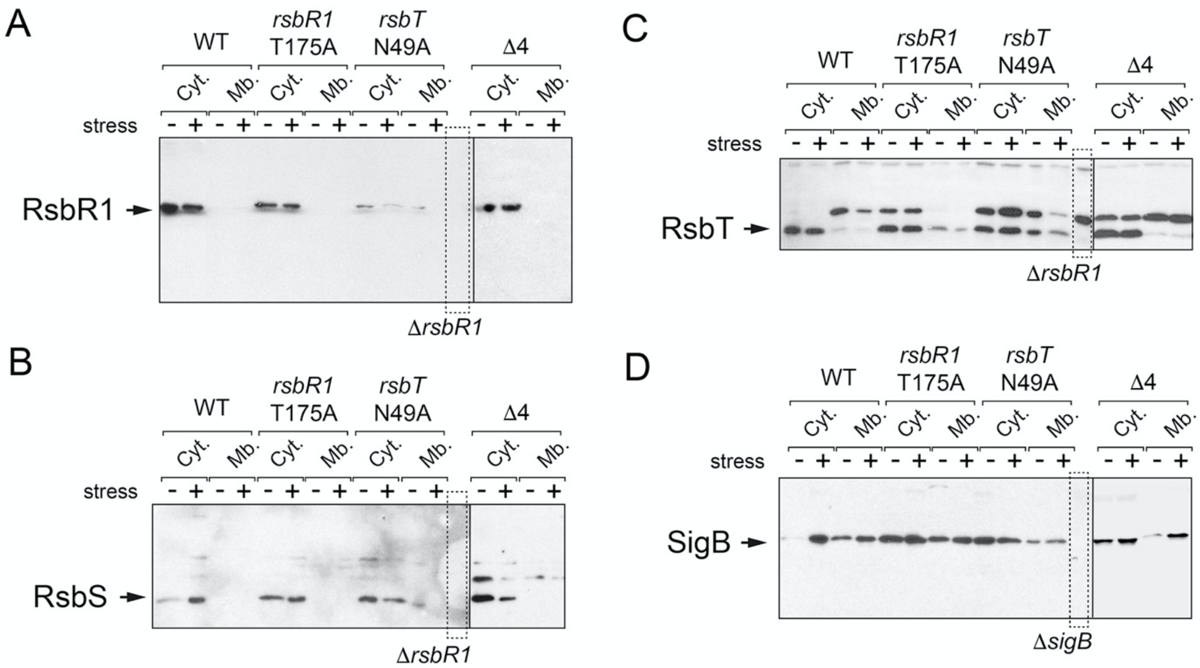

Figure S5. Levels of stressosome proteins RsbR1 (A), RsbS (B), RsbT (C) and the sigma factor SigB (D) in *L. monocytogenes* mutants with phosphorylation defects and in response to osmotic stress. Shown are western blot data representative from a total of three biological replicates and obtained from cytosolic and membrane fractions of the indicated mutants. Main alterations observed included the decrease of RsbR1 levels in the kinase-defective mutant *rsbT* N49A and the no increment of SigB levels in response to stress in the same *rsbT* N49A mutant. Vertical lines indicate parts of different blots that were grouped to assemble the figures. See Supplementary Fig. S7 for images of full blots. Dashed rectangles highlight the gel lanes corresponding to negative control strains.

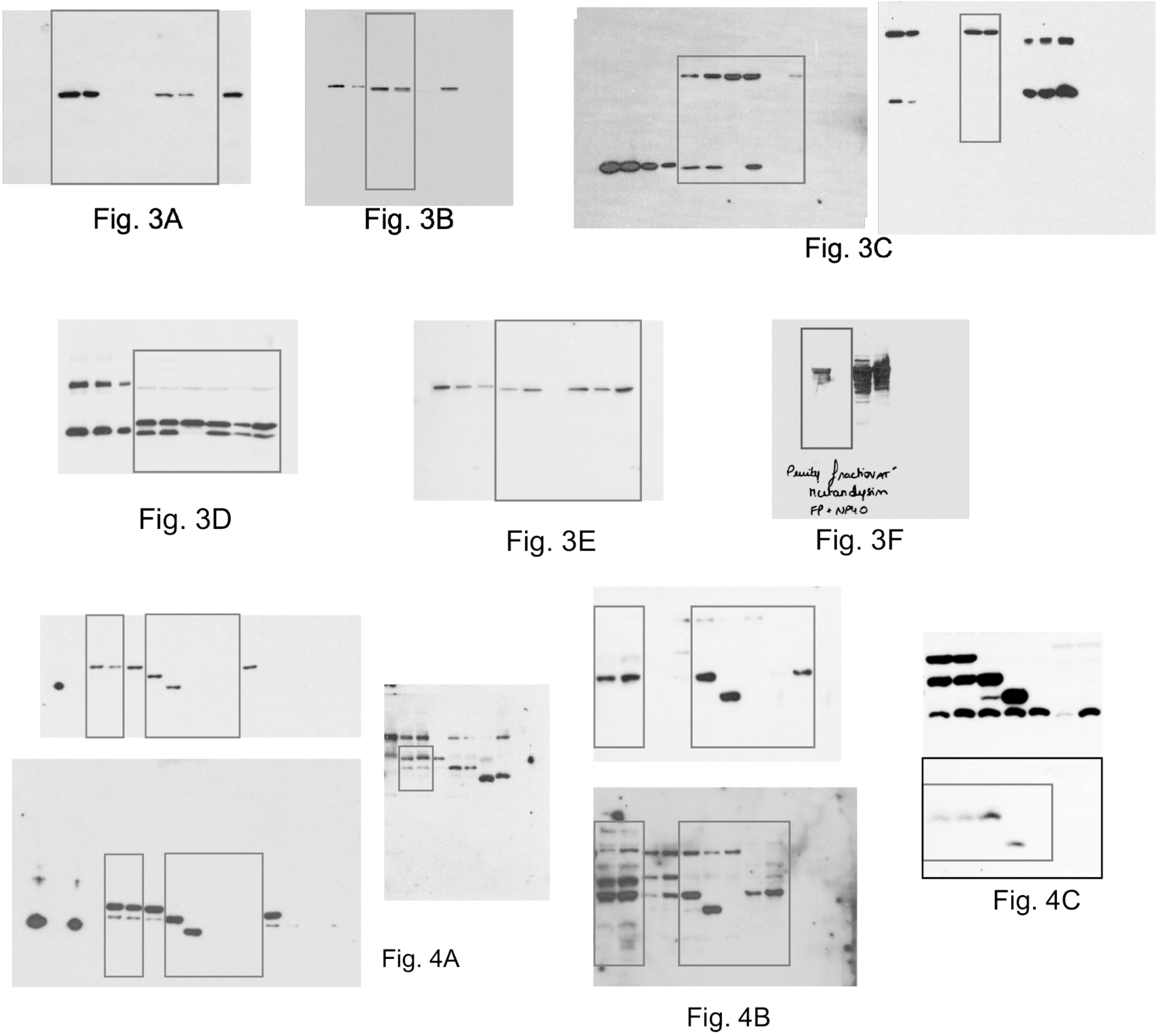

Figure S6. Full blots corresponding to the Figures 3 and 4 shown in the manuscript. Red boxes indicate the cropped regions for each figure. In figure 4C, the edges of the membrane in which anti-RsbS was used for the immunoblot are marked in black.

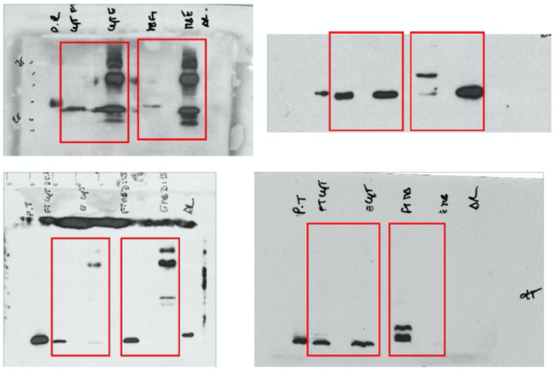

Fig. 6A

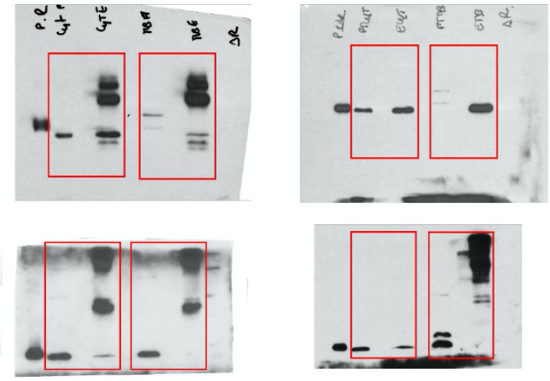

Fig. 6B

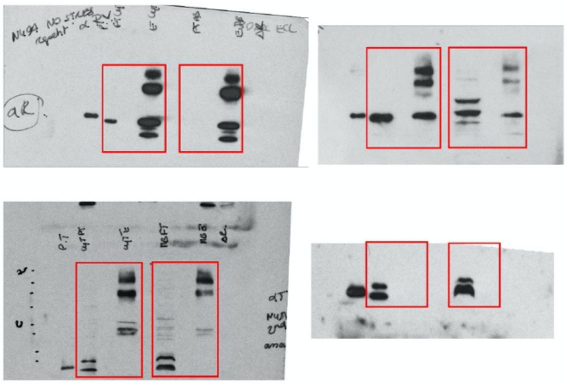

Fig. 6C

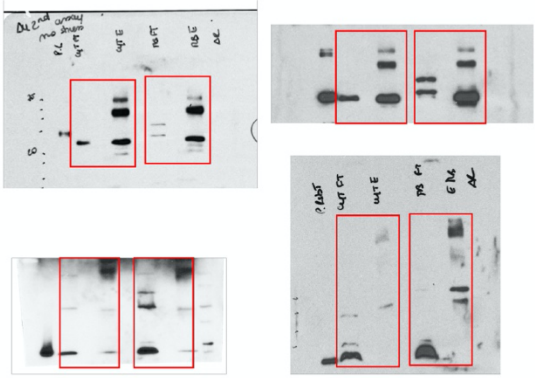

Fig. 6D

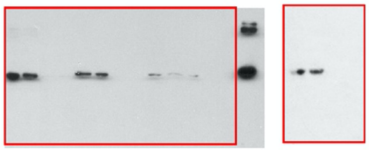

Fig. S5-A

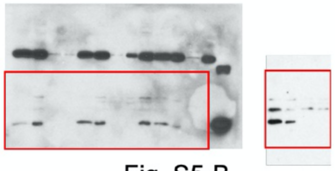

Fig. S5-B

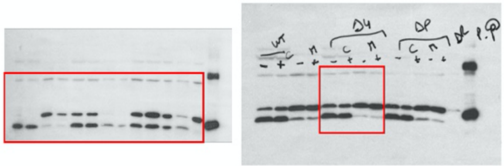

Fig. S5-C

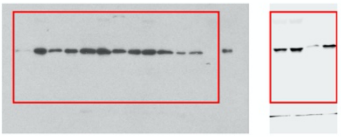

Fig. S5-D

Figure S7. Full blots corresponding to Figure 6 and Supplementary Figure S5. Red boxes indicate the cropped regions for each figure. In Figure S5, the figures of panels A, B, C, and D, were assembled with parts from distinct blots, as shown. These parts are separated by vertical lines in the respective images shown in Supplementary Fig. S5.

RsbR1 – tryptic peptide: **FNEKLDDFAEK**

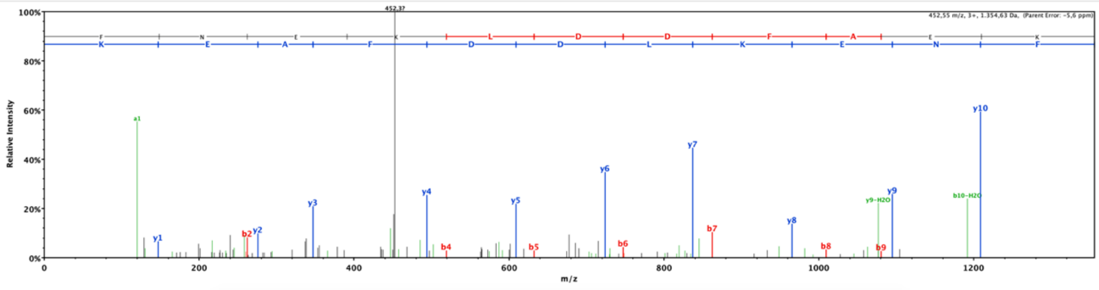

Lmo0161 – tryptic peptide: **LIEEISTPVISITDK**

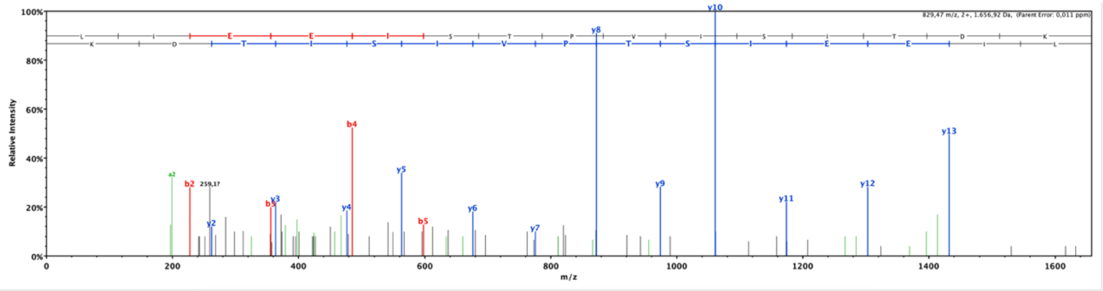

Lmo0799 – tryptic peptide: **DVTTEHDYQLELEK**

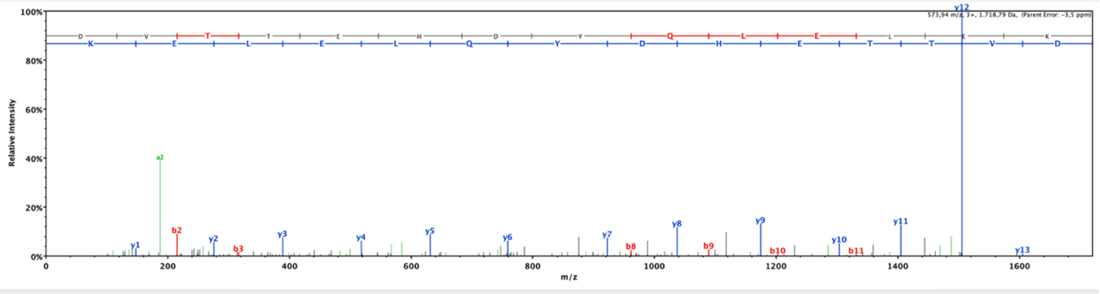

Lmo1642 – tryptic peptide: **LSSGEEEVSIR**

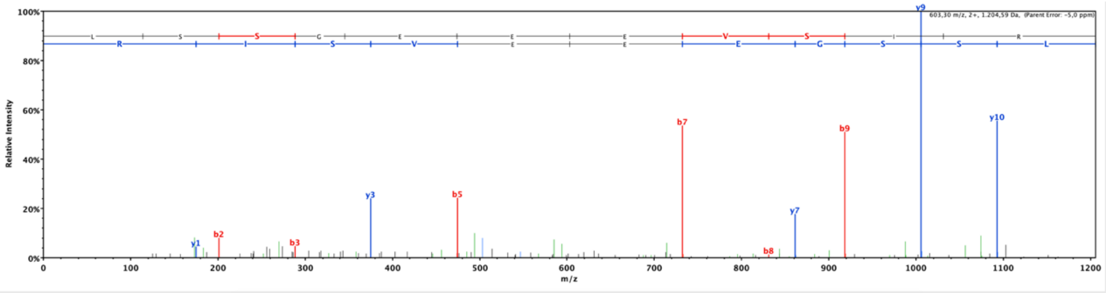

Figure S8. Examples of peptide fragmentation spectra of high confidence identified for distinct *L. monocytogenes* stressosome proteins after immunoprecipitation with anti-RsbR1 antibodies in extracts of wild type bacteria EGD-e.

RbsR1 – tryptic peptide SALQELSAPLLPIFEK

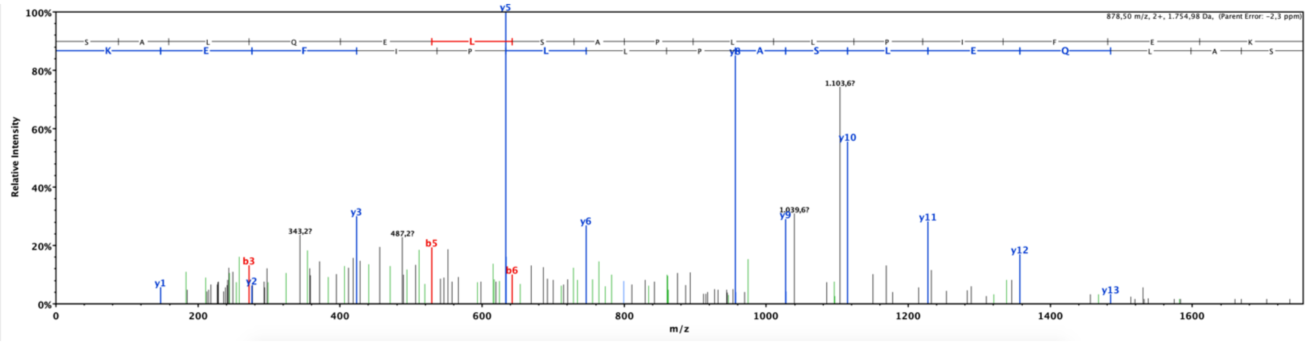

Figure S9. Example of a peptide fragmentation spectrum of low confidence identified for *L. monocytogenes* RsbR1 after immuno-precipitation with anti-RsbR1 antibodies in extracts of Δ*rsbR1* mutant. This identification does not pass the quality threshold.

TABLE S1. Bacterial strains and plasmids used in this study

Bacterial strains	Reference/ source
Escherichia coli	
DH-5α	Lab stock
BL21 (DE3)	Novagen
Listeria monocytogenes	
EGD-e	K. Boor
EGD-e Δ*sigB*	[1]
EGD-e Δ*rsbR1*	[2]
EGD-e *rsbL*-(C56A)	[3]
EGD-e *rsbL*-(C56A) Δ*lmo1842*	This study
EGD-e *rsbL*-(C56A) Δ*lmo1842* Δ*lmo1642*	This study
EGD-e *rsbL*-(C56A) Δ*lmo1842* Δ*lmo1642* Δ*lmo0161*	This study
EGD-e *rsbR* (T175A)	This study
EGD-e *rsbT* (N49A)	This study
EGD-e WT/pKSV7-$P_{lmo2230}$:: *eGFP*	[4]
EGD-e Δ*sigB*/pKSV7-$P_{lmo2230}$:: *eGFP*	[1]
EGD-e *lmo0799* (C56A); Δ*lmo1842*; Δ*lmo1642*; Δ*lmo0161*/pKSV7-$P_{lmo2230}$::*eGFP*	This study
EGD-e *rsbR* (T175A) / pKSV7-$P_{lmo2230}$::*eGFP*	This study
EGD-e *rsbT* (N49A) / pKSV7-$P_{lmo2230}$:: *eGFP*	This study
Bacillus subtilis	
BG214 *(trpCE metA5 amyE1 rsbV37 xre1 xkdA1 attSPβ attICEBs1)*	J.C. Alonso
**Plasmids**	**Reference/ source**
pKSV7-$P_{lmo2230}$::*eGFP*	[4]
pMAD	[5]
pEX-A128::*rsbR1* (T175A)	Eurofins Genomics
pEX-K168::*rsbT* (N49A)	Eurofins Genomics
pMAD::*rsbR1* (T175A)	This study
pMAD::*rsbT* (N49A)	This study

TABLE S2. Oligonucleotides used in this study

Sequence (5'-3')	Target
GAAAGATAGCACGTGCTAATTCA	*rsbT* (N49A)_R
GGCACGCTCCGCATCTATC	*rsbR* (T175A)_R
CACCATGTATAAAGATTTTGCAAACTTCATCCG	*rsbR* _upflank_F
ATAAAGGAGGCCAAACATATGG	*lmo1842*_flank_F
GATGGGAAAGAAGCGAGAAC	*lmo1842*_flank_R
GCAGCAGAGGAAATCATCAAC	*lmo1642*_flank_F
GTCCTTAATTACTCGGCCATC	*lmo1642*_flank_R
AGTGCTGTGCGCTTCTTC	*lmo0161*_flank_F
ACGGTTGCTGATTTACTTCC	*lmo0161*_flank_R

References

1. Guerreiro, D. N. et al. Mild stress conditions during laboratory culture promote the proliferation of mutations that negatively affect Sigma B activity in Listeria monocytogenes. J Bacteriol, doi:10.1128/JB.00751-19 (2020).
2. D’Donoghue, B. A molecular genetic investigation into stress sensing in the food-borne pathogen Listeria monocytogenes: roles for RsbR and its paralogues, National University of Ireland, (2016).
3. O'Donoghue, B. et al. Blue-Light Inhibition of Listeria monocytogenes Growth Is Mediated by Reactive Oxygen Species and Is Influenced by sigmaB and the Blue-Light Sensor Lmo0799. Appl Environ Microbiol 82, 4017-4027, doi:10.1128/AEM.00685-16 (2016).
4. Utratna, M., Cosgrave, E., Baustian, C., Ceredig, R. & O'Byrne, C. Development and optimization of an EGFP-based reporter for measuring the general stress response in Listeria monocytogenes. Bioeng Bugs 3, 93-103, doi:10.4161/bbug.19476 (2012).
5. Arnaud, M., Chastanet, A. & Debarbouille, M. New vector for efficient allelic replacement in naturally nontransformable, low-GC-content, gram-positive bacteria. Appl Environ Microbiol 70, 6887-6891, doi:10.1128/AEM.70.11.6887-6891.2004 (2004).
