# Supplementary material for: Impact of osmotic stress on the phosphorylation and subcellular location of Listeria monocytogenes stressosome proteins
Source: Sci Rep. 2020 Nov 30;10:20837. doi: 10.1038/s41598-020-77738-z (PMC7705745; doi:10.1038/s41598-020-77738-z)
Supplement: Supplementary file 1 — Supplementary Information. [file 41598_2020_77738_MOESM1_ESM.zip › Dessaux-et-al-Supp-Information/MS-Proteomics-3-assays/WT-cytosol-assay-exp3.pdf]

MATRIX SCIENCE Mascot Search Results

**User**	:	
**Email**	:	
**Search title**	:	20181004_Charlotte_CNB_Cyt_WT
**MS data file**	:	20181004_Charlotte_CNB_Cyt_WT.mgf
**Database**	:	UKBsp_p169963_TD p169963_TD_20180702 (5856 sequences; 1791414 residues)
**Timestamp**	:	5 Oct 2018 at 14:57:11 GMT
**Enzyme**	:	Trypsin
**Fixed modifications**	:	Carbamidomethyl (C)
**Variable modifications**	:	Acetyl (Protein N-term),Oxidation (M)
**Mass values**	:	Monoisotopic
**Protein Mass**	:	Unrestricted
**Peptide Mass Tolerance**	:	± 25 ppm
**Fragment Mass Tolerance**	:	± 0.1 Da
**Max Missed Cleavages**	:	1
**Instrument type**	:	ESI-QUAD-TOF
**Number of queries**	:	21840

Protein hits :

tr|Q8Y8K9	RsbR protein OS=Listeria monocytogenes serovar 1/2a (strain ATCC BAA-679 / EGD-e) OX=169963 GN=RsbR PE=4 SV=1
sp|P02769	Serum albumin (Laboratory-Cont) OS=Bos taurus GN=ALB PE=1 SV=4
sp|P04264	Keratin, type II cytoskeletal 1 (Contact-Cont) OS=Homo sapiens GN=KRT1 PE=1 SV=6
sp|P13645	Keratin, type I cytoskeletal 10 (Contact-Cont) OS=Homo sapiens GN=KRT10 PE=1 SV=6
sp|Q9AGE6	60 kDa chaperonin OS=Listeria monocytogenes serovar 1/2a (strain ATCC BAA-679 / EGD-e) OX=169963 GN=groL PE=3 SV
sp|P35527	Keratin, type I cytoskeletal 9 (Contact-Cont) OS=Homo sapiens GN=KRT9 PE=1 SV=3
sp|P35908	Keratin, type II cytoskeletal 2 epidermal (Contact-Cont) OS=Homo sapiens GN=KRT2 PE=1 SV=2
sp|P00761	Trypsin (Laboratory-Cont) OS=Sus scrofa PE=1 SV=1
sp|P0DJM2	Chaperone protein DnaK OS=Listeria monocytogenes serovar 1/2a (strain ATCC BAA-679 / EGD-e) OX=169963 GN=dnaK PE
sp|Q8Y422	Elongation factor Tu OS=Listeria monocytogenes serovar 1/2a (strain ATCC BAA-679 / EGD-e) OX=169963 GN=tuf PE=1
tr|Q8Y6W1	Pyruvate kinase OS=Listeria monocytogenes serovar 1/2a (strain ATCC BAA-679 / EGD-e) OX=169963 GN=pykA PE=3 SV=1
sp|Q927L9	50S ribosomal protein L5 OS=Listeria monocytogenes serovar 1/2a (strain ATCC BAA-679 / EGD-e) OX=169963 GN=rplE
tr|Q8Y863	Dihydrolipoamide acetyltransferase component of pyruvate dehydrogenase complex OS=Listeria monocytogenes serovar
sp|Q8Y6M6	30S ribosomal protein S2 OS=Listeria monocytogenes serovar 1/2a (strain ATCC BAA-679 / EGD-e) OX=169963 GN=rpsB
tr|Q8Y498	FbaA protein OS=Listeria monocytogenes serovar 1/2a (strain ATCC BAA-679 / EGD-e) OX=169963 GN=fbaA PE=3 SV=1
sp|Q8Y440	50S ribosomal protein L3 OS=Listeria monocytogenes serovar 1/2a (strain ATCC BAA-679 / EGD-e) OX=169963 GN=rplC
tr|Q8Y865	PdhA protein OS=Listeria monocytogenes serovar 1/2a (strain ATCC BAA-679 / EGD-e) OX=169963 GN=pdhA PE=4 SV=1
sp|Q8Y6M7	Elongation factor Ts OS=Listeria monocytogenes serovar 1/2a (strain ATCC BAA-679 / EGD-e) OX=169963 GN=tsf PE=3
sp|P66548	30S ribosomal protein S3 OS=Listeria monocytogenes serovar 1/2a (strain ATCC BAA-679 / EGD-e) OX=169963 GN=rpsC
tr|Q8Y7B0	6-phosphogluconate dehydrogenase, decarboxylating OS=Listeria monocytogenes serovar 1/2a (strain ATCC BAA-679 /
sp|Q8Y822	GMP synthase [glutamine-hydrolyzing] OS=Listeria monocytogenes serovar 1/2a (strain ATCC BAA-679 / EGD-e) OX=169
tr|Q8Y4I1	Glyceraldehyde-3-phosphate dehydrogenase OS=Listeria monocytogenes serovar 1/2a (strain ATCC BAA-679 / EGD-e) OX
tr|Q8YAN9	Lmo0078 protein OS=Listeria monocytogenes serovar 1/2a (strain ATCC BAA-679 / EGD-e) OX=169963 GN=lmo0078 PE=3 S
sp|Q8Y446	30S ribosomal protein S5 OS=Listeria monocytogenes serovar 1/2a (strain ATCC BAA-679 / EGD-e) OX=169963 GN=rpsE
sp|Q8Y4B3	Uracil phosphoribosyltransferase OS=Listeria monocytogenes serovar 1/2a (strain ATCC BAA-679 / EGD-e) OX=169963
sp|Q8Y5G2	Pyridoxal 5'-phosphate synthase subunit PdxS OS=Listeria monocytogenes serovar 1/2a (strain ATCC BAA-679 / EGD-e
tr|Q8Y862	Dihydrolipoyl dehydrogenase OS=Listeria monocytogenes serovar 1/2a (strain ATCC BAA-679 / EGD-e) OX=169963 GN=Pd
sp|Q8YAA4	50S ribosomal protein L1 OS=Listeria monocytogenes serovar 1/2a (strain ATCC BAA-679 / EGD-e) OX=169963 GN=rplA
sp|Q8Y421	Elongation factor G OS=Listeria monocytogenes serovar 1/2a (strain ATCC BAA-679 / EGD-e) OX=169963 GN=fusA PE=3
sp|Q8Y915	Glutamine--fructose-6-phosphate aminotransferase [isomerizing] OS=Listeria monocytogenes serovar 1/2a (strain AT
sp|Q8Y6W0	ATP-dependent 6-phosphofructokinase OS=Listeria monocytogenes serovar 1/2a (strain ATCC BAA-679 / EGD-e) OX=1699
sp|O31149	Phosphoenolpyruvate-protein phosphotransferase OS=Listeria monocytogenes serovar 1/2a (strain ATCC BAA-679 / EGD
sp|Q8Y6D2	Glutamyl-tRNA(Gln) amidotransferase subunit A OS=Listeria monocytogenes serovar 1/2a (strain ATCC BAA-679 / EGD-
sp|O77727	Keratin, type I cytoskeletal 15 (Contact-Cont) OS=Ovis aries GN=KRT15 PE=2 SV=1
tr|Q8Y4M2	Lmo2415 protein OS=Listeria monocytogenes serovar 1/2a (strain ATCC BAA-679 / EGD-e) OX=169963 GN=lmo2415 PE=4 S
sp|P66042	50S ribosomal protein L10 OS=Listeria monocytogenes serovar 1/2a (strain ATCC BAA-679 / EGD-e) OX=169963 GN=rplJ
tr|Q8Y6P3	Aconitate hydratase OS=Listeria monocytogenes serovar 1/2a (strain ATCC BAA-679 / EGD-e) OX=169963 GN=citB PE=3
sp|P66330	30S ribosomal protein S10 OS=Listeria monocytogenes serovar 1/2a (strain ATCC BAA-679 / EGD-e) OX=169963 GN=rpsJ
tr|Q8Y6T3	Catabolite control protein A OS=Listeria monocytogenes serovar 1/2a (strain ATCC BAA-679 / EGD-e) OX=169963 GN=c
tr|Q926Y9	Inosine-5'-monophosphate dehydrogenase OS=Listeria monocytogenes serovar 1/2a (strain ATCC BAA-679 / EGD-e) OX=1
sp|P64074	Enolase OS=Listeria monocytogenes serovar 1/2a (strain ATCC BAA-679 / EGD-e) OX=169963 GN=eno PE=1 SV=1
sp|Q8Y4R7	Glucose-6-phosphate isomerase OS=Listeria monocytogenes serovar 1/2a (strain ATCC BAA-679 / EGD-e) OX=169963 GN=
sp|P61055	50S ribosomal protein L4 OS=Listeria monocytogenes serovar 1/2a (strain ATCC BAA-679 / EGD-e) OX=169963 GN=rplD
tr|Q8Y8D5	Enoyl-[acyl-carrier-protein] reductase [NADH] OS=Listeria monocytogenes serovar 1/2a (strain ATCC BAA-679 / EGD-
sp|Q8Y495	CTP synthase OS=Listeria monocytogenes serovar 1/2a (strain ATCC BAA-679 / EGD-e) OX=169963 GN=pyrG PE=3 SV=1
tr|Q8Y6Y3	MreB protein OS=Listeria monocytogenes serovar 1/2a (strain ATCC BAA-679 / EGD-e) OX=169963 GN=mreB PE=4 SV=1
sp|P66054	50S ribosomal protein L11 OS=Listeria monocytogenes serovar 1/2a (strain ATCC BAA-679 / EGD-e) OX=169963 GN=rplK
sp|Q8Y444	50S ribosomal protein L6 OS=Listeria monocytogenes serovar 1/2a (strain ATCC BAA-679 / EGD-e) OX=169963 GN=rplF
sp|Q8Y766	4-hydroxy-tetrahydrodipicolinate synthase OS=Listeria monocytogenes serovar 1/2a (strain ATCC BAA-679 / EGD-e) O
sp|Q8Y7J7	GTP-sensing transcriptional pleiotropic repressor CodY OS=Listeria monocytogenes serovar 1/2a (strain ATCC BAA-6
sp|Q8YA96	DNA-directed RNA polymerase subunit beta' OS=Listeria monocytogenes serovar 1/2a (strain ATCC BAA-679 / EGD-e) O
sp|Q8YAA3	50S ribosomal protein L7/L12 OS=Listeria monocytogenes serovar 1/2a (strain ATCC BAA-679 / EGD-e) OX=169963 GN=r
tr|Q8Y864	PdhB protein OS=Listeria monocytogenes serovar 1/2a (strain ATCC BAA-679 / EGD-e) OX=169963 GN=PdhB PE=4 SV=1
sp|P33380	L-lactate dehydrogenase 1 OS=Listeria monocytogenes serovar 1/2a (strain ATCC BAA-679 / EGD-e) OX=169963 GN=ldh1
tr|Q8Y690	FabG protein OS=Listeria monocytogenes serovar 1/2a (strain ATCC BAA-679 / EGD-e) OX=169963 GN=fabG PE=1 SV=1
sp|Q8YAF2	Methionine--tRNA ligase OS=Listeria monocytogenes serovar 1/2a (strain ATCC BAA-679 / EGD-e) OX=169963 GN=metG P
sp|Q8Y6T6	30S ribosomal protein S4 OS=Listeria monocytogenes serovar 1/2a (strain ATCC BAA-679 / EGD-e) OX=169963 GN=rpsD
tr|Q8Y6L1	1,4-dihydroxy-2-naphthoyl-CoA synthase OS=Listeria monocytogenes serovar 1/2a (strain ATCC BAA-679 / EGD-e) OX=1
tr|Q8Y730	Lmo1493 protein OS=Listeria monocytogenes serovar 1/2a (strain ATCC BAA-679 / EGD-e) OX=169963 GN=lmo1493 PE=3 S
sp|Q9RLT9	DNA-directed RNA polymerase subunit beta OS=Listeria monocytogenes serovar 1/2a (strain ATCC BAA-679 / EGD-e) OX
tr|Q8Y3P3	Lmo2792 protein OS=Listeria monocytogenes serovar 1/2a (strain ATCC BAA-679 / EGD-e) OX=169963 GN=lmo2792 PE=4 S
tr|Q8Y7I0	Glutamine synthetase OS=Listeria monocytogenes serovar 1/2a (strain ATCC BAA-679 / EGD-e) OX=169963 GN=glnA PE=3
sp|P02662	Alpha-S1-casein (Laboratory-Cont) OS=Bos taurus GN=CSN1S1 PE=1 SV=2
sp|P66611	30S ribosomal protein S7 OS=Listeria monocytogenes serovar 1/2a (strain ATCC BAA-679 / EGD-e) OX=169963 GN=rpsG
tr|Q8Y8T0	Lmo0814 protein OS=Listeria monocytogenes serovar 1/2a (strain ATCC BAA-679 / EGD-e) OX=169963 GN=lmo0814 PE=4 S
sp|O53083	50S ribosomal protein L19 OS=Listeria monocytogenes serovar 1/2a (strain ATCC BAA-679 / EGD-e) OX=169963 GN=rplS
sp|Q9RQI6	ATP-dependent Clp protease proteolytic subunit OS=Listeria monocytogenes serovar 1/2a (strain ATCC BAA-679 / EGD
tr|Q8YAW1	Beta sliding clamp OS=Listeria monocytogenes serovar 1/2a (strain ATCC BAA-679 / EGD-e) OX=169963 GN=dnaN PE=3 S
sp|P66699	DNA-directed RNA polymerase subunit alpha OS=Listeria monocytogenes serovar 1/2a (strain ATCC BAA-679 / EGD-e) O
sp|P0DJP0	Protein RecA OS=Listeria monocytogenes serovar 1/2a (strain ATCC BAA-679 / EGD-e) OX=169963 GN=recA PE=3 SV=1
sp|Q8Y634	Putative pyruvate, phosphate dikinase regulatory protein 2 OS=Listeria monocytogenes serovar 1/2a (strain ATCC B
sp|P66383	30S ribosomal protein S13 OS=Listeria monocytogenes serovar 1/2a (strain ATCC BAA-679 / EGD-e) OX=169963 GN=rpsM
sp|P02666	Beta-casein (Laboratory-Cont) OS=Bos taurus GN=CSN2 PE=1 SV=2
tr|Q7AP52	Lmo2196 protein OS=Listeria monocytogenes serovar 1/2a (strain ATCC BAA-679 / EGD-e) OX=169963 GN=lmo2196 PE=4 S
sp|Q8Y5E6	Phosphoglucosamine mutase OS=Listeria monocytogenes serovar 1/2a (strain ATCC BAA-679 / EGD-e) OX=169963 GN=glmM
sp|Q8Y450	50S ribosomal protein L17 OS=Listeria monocytogenes serovar 1/2a (strain ATCC BAA-679 / EGD-e) OX=169963 GN=rplQ
tr|Q8Y5B3	Ribonucleoside-diphosphate reductase subunit beta OS=Listeria monocytogenes serovar 1/2a (strain ATCC BAA-679 /
sp|P66352	30S ribosomal protein S11 OS=Listeria monocytogenes serovar 1/2a (strain ATCC BAA-679 / EGD-e) OX=169963 GN=rpsK
tr|Q8Y7F9	Transcription termination/antitermination protein NusA OS=Listeria monocytogenes serovar 1/2a (strain ATCC BAA-6
sp|Q8Y4I3	Triosephosphate isomerase 1 OS=Listeria monocytogenes serovar 1/2a (strain ATCC BAA-679 / EGD-e) OX=169963 GN=tp
sp|Q8Y4C0	ATP synthase subunit alpha 2 OS=Listeria monocytogenes serovar 1/2a (strain ATCC BAA-679 / EGD-e) OX=169963 GN=a
tr|Q8Y6Q0	Aldehyde-alcohol dehydrogenase OS=Listeria monocytogenes serovar 1/2a (strain ATCC BAA-679 / EGD-e) OX=169963 GN
sp|Q48793	Ribose-phosphate pyrophosphokinase 1 OS=Listeria monocytogenes serovar 1/2a (strain ATCC BAA-679 / EGD-e) OX=169
sp|Q8Y573	3-oxoacyl-[acyl-carrier-protein] synthase 3 OS=Listeria monocytogenes serovar 1/2a (strain ATCC BAA-679 / EGD-e)
sp|Q8Y6Y9	50S ribosomal protein L21 OS=Listeria monocytogenes serovar 1/2a (strain ATCC BAA-679 / EGD-e) OX=169963 GN=rplU
sp|Q8Y441	50S ribosomal protein L23 OS=Listeria monocytogenes serovar 1/2a (strain ATCC BAA-679 / EGD-e) OX=169963 GN=rplW
sp|Q8Y493	Arginine--tRNA ligase OS=Listeria monocytogenes serovar 1/2a (strain ATCC BAA-679 / EGD-e) OX=169963 GN=argS PE=

sp|Q927L7	50S ribosomal protein L14 OS=Listeria monocytogenes serovar 1/2a (strain ATCC BAA-679 / EGD-e) OX=169963 GN=rplN
sp|Q8Y754	Glycine--tRNA ligase beta subunit OS=Listeria monocytogenes serovar 1/2a (strain ATCC BAA-679 / EGD-e) OX=169963
sp|Q927L2	50S ribosomal protein L22 OS=Listeria monocytogenes serovar 1/2a (strain ATCC BAA-679 / EGD-e) OX=169963 GN=rplV
sp|Q8Y709	Aspartate--tRNA ligase OS=Listeria monocytogenes serovar 1/2a (strain ATCC BAA-679 / EGD-e) OX=169963 GN=aspS PE
tr|Q8Y851	Lmo1067 protein OS=Listeria monocytogenes serovar 1/2a (strain ATCC BAA-679 / EGD-e) OX=169963 GN=lmo1067 PE=4 S
sp|Q8Y7K9	ATP-dependent Clp protease ATP-binding subunit ClpX OS=Listeria monocytogenes serovar 1/2a (strain ATCC BAA-679
tr|Q8Y4M3	Lmo2414 protein OS=Listeria monocytogenes serovar 1/2a (strain ATCC BAA-679 / EGD-e) OX=169963 GN=lmo2414 PE=4 S
sp|Q8Y7N6	Ribonuclease PH OS=Listeria monocytogenes serovar 1/2a (strain ATCC BAA-679 / EGD-e) OX=169963 GN=rph PE=3 SV=1
tr|Q8Y4U6	FruA protein OS=Listeria monocytogenes serovar 1/2a (strain ATCC BAA-679 / EGD-e) OX=169963 GN=fruA PE=4 SV=1
sp|Q8Y459	30S ribosomal protein S9 OS=Listeria monocytogenes serovar 1/2a (strain ATCC BAA-679 / EGD-e) OX=169963 GN=rpsI
sp|P65927	Uridylate kinase OS=Listeria monocytogenes serovar 1/2a (strain ATCC BAA-679 / EGD-e) OX=169963 GN=pyrH PE=3 SV=
tr|Q8YAB6	Endopeptidase Clp ATP-binding chain C OS=Listeria monocytogenes serovar 1/2a (strain ATCC BAA-679 / EGD-e) OX=16
tr|Q8YAC3	Cysteine synthase OS=Listeria monocytogenes serovar 1/2a (strain ATCC BAA-679 / EGD-e) OX=169963 GN=cysK PE=3 SV
sp|Q8Y6X9	Valine--tRNA ligase OS=Listeria monocytogenes serovar 1/2a (strain ATCC BAA-679 / EGD-e) OX=169963 GN=valS PE=3
tr|Q8YAJ0	Lmo0135 protein OS=Listeria monocytogenes serovar 1/2a (strain ATCC BAA-679 / EGD-e) OX=169963 GN=lmo0135 PE=1 S
sp|P66372	30S ribosomal protein S12 OS=Listeria monocytogenes serovar 1/2a (strain ATCC BAA-679 / EGD-e) OX=169963 GN=rpsL
tr|Q8Y786	Pyruvate formate-lyase OS=Listeria monocytogenes serovar 1/2a (strain ATCC BAA-679 / EGD-e) OX=169963 GN=pflB PE
sp|Q8Y6M4	Leucine--tRNA ligase OS=Listeria monocytogenes serovar 1/2a (strain ATCC BAA-679 / EGD-e) OX=169963 GN=leuS PE=3
sp|Q8YAD3	50S ribosomal protein L25 OS=Listeria monocytogenes serovar 1/2a (strain ATCC BAA-679 / EGD-e) OX=169963 GN=rplY
tr|Q8Y6T2	3-deoxy-D-arabino-heptulosonate 7-phosphate synthase OS=Listeria monocytogenes serovar 1/2a (strain ATCC BAA-679
sp|Q8Y458	50S ribosomal protein L13 OS=Listeria monocytogenes serovar 1/2a (strain ATCC BAA-679 / EGD-e) OX=169963 GN=rplM
sp|Q8Y6M0	S-adenosylmethionine synthase OS=Listeria monocytogenes serovar 1/2a (strain ATCC BAA-679 / EGD-e) OX=169963 GN=
tr|Q8Y9H0	Lmo0558 protein OS=Listeria monocytogenes serovar 1/2a (strain ATCC BAA-679 / EGD-e) OX=169963 GN=lmo0558 PE=4 S
sp|Q8Y7Q1	Phenylalanine--tRNA ligase beta subunit OS=Listeria monocytogenes serovar 1/2a (strain ATCC BAA-679 / EGD-e) OX=
tr|Q8Y7E4	Lmo1339 protein OS=Listeria monocytogenes serovar 1/2a (strain ATCC BAA-679 / EGD-e) OX=169963 GN=lmo1339 PE=4 S
sp|Q8Y443	50S ribosomal protein L24 OS=Listeria monocytogenes serovar 1/2a (strain ATCC BAA-679 / EGD-e) OX=169963 GN=rplX
tr|Q8Y685	Lmo1812 protein OS=Listeria monocytogenes serovar 1/2a (strain ATCC BAA-679 / EGD-e) OX=169963 GN=lmo1812 PE=4 S
tr|Q8YAV6	DNA gyrase subunit A OS=Listeria monocytogenes serovar 1/2a (strain ATCC BAA-679 / EGD-e) OX=169963 GN=gyrA PE=3
tr|Q8Y7B2	Dihydrolipoamide acetyltransferase component of pyruvate dehydrogenase complex OS=Listeria monocytogenes serovar
sp|Q9NSB2	Keratin, type II cuticular Hb4 (Contact-Cont) OS=Homo sapiens GN=KRT84 PE=2 SV=2
tr|Q8Y6W5	Isocitrate dehydrogenase [NADP] OS=Listeria monocytogenes serovar 1/2a (strain ATCC BAA-679 / EGD-e) OX=169963 G
tr|Q8Y5M5	Cell division protein FtsZ OS=Listeria monocytogenes serovar 1/2a (strain ATCC BAA-679 / EGD-e) OX=169963 GN=fts
tr|Q8Y4G8	Lmo2475 protein OS=Listeria monocytogenes serovar 1/2a (strain ATCC BAA-679 / EGD-e) OX=169963 GN=lmo2475 PE=3 S
sp|P02663	Alpha-S2-casein (Laboratory-Cont) OS=Bos taurus GN=CSN1S2 PE=1 SV=2
tr|Q8Y6J6	Lmo1688 protein OS=Listeria monocytogenes serovar 1/2a (strain ATCC BAA-679 / EGD-e) OX=169963 GN=lmo1688 PE=4 S
sp|Q8Y4C4	UDP-N-acetylglucosamine 1-carboxyvinyltransferase 1 OS=Listeria monocytogenes serovar 1/2a (strain ATCC BAA-679
sp|P0DJM1	Chaperone protein DnaJ OS=Listeria monocytogenes serovar 1/2a (strain ATCC BAA-679 / EGD-e) OX=169963 GN=dnaJ PE
tr|Q8Y770	Lmo1431 protein OS=Listeria monocytogenes serovar 1/2a (strain ATCC BAA-679 / EGD-e) OX=169963 GN=lmo1431 PE=4 S
sp|Q8Y699	30S ribosomal protein S16 OS=Listeria monocytogenes serovar 1/2a (strain ATCC BAA-679 / EGD-e) OX=169963 GN=rpsP
sp|Q8Y5W9	Glycerol-3-phosphate dehydrogenase [NAD(P)+] OS=Listeria monocytogenes serovar 1/2a (strain ATCC BAA-679 / EGD-e
tr|Q8Y436	Lmo2637 protein OS=Listeria monocytogenes serovar 1/2a (strain ATCC BAA-679 / EGD-e) OX=169963 GN=lmo2637 PE=4 S
sp|Q8Y688	Phosphate acyltransferase OS=Listeria monocytogenes serovar 1/2a (strain ATCC BAA-679 / EGD-e) OX=169963 GN=plsX
tr|Q8Y5V7	ResD protein OS=Listeria monocytogenes serovar 1/2a (strain ATCC BAA-679 / EGD-e) OX=169963 GN=resD PE=4 SV=1
tr|Q8Y782	Lmo1414 protein OS=Listeria monocytogenes serovar 1/2a (strain ATCC BAA-679 / EGD-e) OX=169963 GN=lmo1414 PE=3 S
tr|Q8Y4U2	Pseudouridine-5'-phosphate glycosidase OS=Listeria monocytogenes serovar 1/2a (strain ATCC BAA-679 / EGD-e) OX=1
tr|Q8Y701	Lmo1529 protein OS=Listeria monocytogenes serovar 1/2a (strain ATCC BAA-679 / EGD-e) OX=169963 GN=lmo1529 PE=4 S
sp|P66401	30S ribosomal protein S14 type Z OS=Listeria monocytogenes serovar 1/2a (strain ATCC BAA-679 / EGD-e) OX=169963
sp|Q8Y8K6	Serine-protein kinase RsbW OS=Listeria monocytogenes serovar 1/2a (strain ATCC BAA-679 / EGD-e) OX=169963 GN=rsb
sp|Q8Y6X8	Glutamate-1-semialdehyde 2,1-aminomutase 1 OS=Listeria monocytogenes serovar 1/2a (strain ATCC BAA-679 / EGD-e)
sp|Q8Y445	50S ribosomal protein L18 OS=Listeria monocytogenes serovar 1/2a (strain ATCC BAA-679 / EGD-e) OX=169963 GN=rplR
sp|Q8Y7C5	Bifunctional protein FolD OS=Listeria monocytogenes serovar 1/2a (strain ATCC BAA-679 / EGD-e) OX=169963 GN=folD
sp|P60384	Redox-sensing transcriptional repressor Rex OS=Listeria monocytogenes serovar 1/2a (strain ATCC BAA-679 / EGD-e)
sp|Q8Y6U0	Probable tRNA sulfurtransferase OS=Listeria monocytogenes serovar 1/2a (strain ATCC BAA-679 / EGD-e) OX=169963 G
sp|Q8Y447	50S ribosomal protein L15 OS=Listeria monocytogenes serovar 1/2a (strain ATCC BAA-679 / EGD-e) OX=169963 GN=rplO
sp|P66484	30S ribosomal protein S19 OS=Listeria monocytogenes serovar 1/2a (strain ATCC BAA-679 / EGD-e) OX=169963 GN=rpsS
tr|Q8Y6B8	Adenylosuccinate lyase OS=Listeria monocytogenes serovar 1/2a (strain ATCC BAA-679 / EGD-e) OX=169963 GN=purB PE
sp|Q8Y3T8	Probable transaldolase 1 OS=Listeria monocytogenes serovar 1/2a (strain ATCC BAA-679 / EGD-e) OX=169963 GN=tal1
sp|P66623	30S ribosomal protein S8 OS=Listeria monocytogenes serovar 1/2a (strain ATCC BAA-679 / EGD-e) OX=169963 GN=rpsH
sp|Q8Y8C0	Peptide chain release factor 3 OS=Listeria monocytogenes serovar 1/2a (strain ATCC BAA-679 / EGD-e) OX=169963 GN
tr|Q8Y764	Aspartate-semialdehyde dehydrogenase OS=Listeria monocytogenes serovar 1/2a (strain ATCC BAA-679 / EGD-e) OX=169
sp|P28764	Superoxide dismutase [Mn] OS=Listeria monocytogenes serovar 1/2a (strain ATCC BAA-679 / EGD-e) OX=169963 GN=sodA
sp|P58695	Asparagine--tRNA ligase OS=Listeria monocytogenes serovar 1/2a (strain ATCC BAA-679 / EGD-e) OX=169963 GN=asnS P
tr|Q8Y8V1	Lmo0791 protein OS=Listeria monocytogenes serovar 1/2a (strain ATCC BAA-679 / EGD-e) OX=169963 GN=lmo0791 PE=4 S
tr|Q8Y7B3	Lmo1373 protein OS=Listeria monocytogenes serovar 1/2a (strain ATCC BAA-679 / EGD-e) OX=169963 GN=lmo1373 PE=4 S
tr|Q8Y835	dTDP-glucose 4,6-dehydratase OS=Listeria monocytogenes serovar 1/2a (strain ATCC BAA-679 / EGD-e) OX=169963 GN=l
tr|Q8Y846	Pyruvate carboxylase OS=Listeria monocytogenes serovar 1/2a (strain ATCC BAA-679 / EGD-e) OX=169963 GN=pycA PE=4
sp|Q9AGE7	10 kDa chaperonin OS=Listeria monocytogenes serovar 1/2a (strain ATCC BAA-679 / EGD-e) OX=169963 GN=groS PE=3 SV
tr|Q8Y7C7	Acetyl-CoA carboxylase subunit (Biotin carboxylase subunit) OS=Listeria monocytogenes serovar 1/2a (strain ATCC
sp|P0DJP1	30S ribosomal protein S21 OS=Listeria monocytogenes serovar 1/2a (strain ATCC BAA-679 / EGD-e) OX=169963 GN=rpsU
sp|Q8Y722	Alanine--tRNA ligase OS=Listeria monocytogenes serovar 1/2a (strain ATCC BAA-679 / EGD-e) OX=169963 GN=alaS PE=3
tr|Q8Y791	Lmo1401 protein OS=Listeria monocytogenes serovar 1/2a (strain ATCC BAA-679 / EGD-e) OX=169963 GN=lmo1401 PE=4 S
tr|Q8Y7J1	DNA topoisomerase 4 subunit B OS=Listeria monocytogenes serovar 1/2a (strain ATCC BAA-679 / EGD-e) OX=169963 GN=
sp|Q8Y6V0	Acetate kinase 1 OS=Listeria monocytogenes serovar 1/2a (strain ATCC BAA-679 / EGD-e) OX=169963 GN=ackA1 PE=3 SV
tr|Q929C7	Lmo2248 protein OS=Listeria monocytogenes serovar 1/2a (strain ATCC BAA-679 / EGD-e) OX=169963 GN=lmo2248 PE=4 S
sp|Q8Y5X5	Chorismate synthase OS=Listeria monocytogenes serovar 1/2a (strain ATCC BAA-679 / EGD-e) OX=169963 GN=aroC PE=3
tr|Q8Y838	Lmo1080 protein OS=Listeria monocytogenes serovar 1/2a (strain ATCC BAA-679 / EGD-e) OX=169963 GN=lmo1080 PE=4 S
tr|Q8Y781	Lmo1415 protein OS=Listeria monocytogenes serovar 1/2a (strain ATCC BAA-679 / EGD-e) OX=169963 GN=lmo1415 PE=4 S
tr|Q8Y5Q0	AlsS protein OS=Listeria monocytogenes serovar 1/2a (strain ATCC BAA-679 / EGD-e) OX=169963 GN=alsS PE=3 SV=1
sp|P13128	Listeriolysin O OS=Listeria monocytogenes serovar 1/2a (strain ATCC BAA-679 / EGD-e) OX=169963 GN=hly PE=1 SV=1
sp|Q8Y624	Formate--tetrahydrofolate ligase OS=Listeria monocytogenes serovar 1/2a (strain ATCC BAA-679 / EGD-e) OX=169963
sp|Q8Y9C1	FMN-dependent NADH-azoreductase 1 OS=Listeria monocytogenes serovar 1/2a (strain ATCC BAA-679 / EGD-e) OX=169963
sp|Q8Y723	Putative pre-16S rRNA nuclease OS=Listeria monocytogenes serovar 1/2a (strain ATCC BAA-679 / EGD-e) OX=169963 GN
tr|Q8Y9D8	Lmo0592 protein OS=Listeria monocytogenes serovar 1/2a (strain ATCC BAA-679 / EGD-e) OX=169963 GN=lmo0592 PE=4 S
sp|Q8YAR1	Adenylosuccinate synthetase OS=Listeria monocytogenes serovar 1/2a (strain ATCC BAA-679 / EGD-e) OX=169963 GN=pu
tr|Q8Y5F0	Lmo2114 protein OS=Listeria monocytogenes serovar 1/2a (strain ATCC BAA-679 / EGD-e) OX=169963 GN=lmo2114 PE=4 S
tr|Q8Y3T6	Lmo2745 protein OS=Listeria monocytogenes serovar 1/2a (strain ATCC BAA-679 / EGD-e) OX=169963 GN=lmo2745 PE=4 S
tr|Q8Y765	Aspartokinase OS=Listeria monocytogenes serovar 1/2a (strain ATCC BAA-679 / EGD-e) OX=169963 GN=lmo1436 PE=3 SV=
tr|Q8Y5P2	Lmo2014 protein OS=Listeria monocytogenes serovar 1/2a (strain ATCC BAA-679 / EGD-e) OX=169963 GN=lmo2014 PE=4 S
tr|Q8YAV7	DNA gyrase subunit B OS=Listeria monocytogenes serovar 1/2a (strain ATCC BAA-679 / EGD-e) OX=169963 GN=gyrB PE=3
tr|Q8Y616	Metal-dependent carboxypeptidase OS=Listeria monocytogenes serovar 1/2a (strain ATCC BAA-679 / EGD-e) OX=169963
DECOY tr|Q8Y9L1	Lmo0516 protein OS=Listeria monocytogenes serovar 1/2a (strain ATCC BAA-679 / EGD-e) OX=169963 GN=lmo0516 PE=4 S
tr|Q8Y993	Lmo0640 protein OS=Listeria monocytogenes serovar 1/2a (strain ATCC BAA-679 / EGD-e) OX=169963 GN=lmo0640 PE=4 S
DECOY tr|Q8Y8I5	Lmo0917 protein OS=Listeria monocytogenes serovar 1/2a (strain ATCC BAA-679 / EGD-e) OX=169963 GN=lmo0917 PE=3 S
tr|Q928M6	Lmo2411 protein OS=Listeria monocytogenes serovar 1/2a (strain ATCC BAA-679 / EGD-e) OX=169963 GN=lmo2411 PE=4 S
DECOY tr|Q8Y5Y6	PflA protein OS=Listeria monocytogenes serovar 1/2a (strain ATCC BAA-679 / EGD-e) OX=169963 GN=pflA PE=4 SV=1
sp|Q8Y5G1	Pyridoxal 5'-phosphate synthase subunit PdxT OS=Listeria monocytogenes serovar 1/2a (strain ATCC BAA-679 / EGD-e
DECOY tr|Q8Y859	Lmo1059 protein OS=Listeria monocytogenes serovar 1/2a (strain ATCC BAA-679 / EGD-e) OX=169963 GN=lmo1059 PE=4 S
DECOY tr|Q8Y3V9	Lmo2722 protein OS=Listeria monocytogenes serovar 1/2a (strain ATCC BAA-679 / EGD-e) OX=169963 GN=lmo2722 PE=4 S
DECOY sp|P00921	Carbonic anhydrase 2 (MW-Marker) OS=Bos taurus GN=CA2 PE=1 SV=3
tr|Q8Y4S2	Transmembrane protein OS=Listeria monocytogenes serovar 1/2a (strain ATCC BAA-679 / EGD-e) OX=169963 GN=lmo2360
tr|Q8Y3P4	Partition protein ParB homolg OS=Listeria monocytogenes serovar 1/2a (strain ATCC BAA-679 / EGD-e) OX=169963 GN=
tr|Q8Y4C5	Mbl protein OS=Listeria monocytogenes serovar 1/2a (strain ATCC BAA-679 / EGD-e) OX=169963 GN=mbl PE=4 SV=1
tr|Q7AP59	Lmo1601 protein OS=Listeria monocytogenes serovar 1/2a (strain ATCC BAA-679 / EGD-e) OX=169963 GN=lmo1601 PE=4 S
tr|Q8Y9F6	Lmo0572 protein OS=Listeria monocytogenes serovar 1/2a (strain ATCC BAA-679 / EGD-e) OX=169963 GN=lmo0572 PE=4 S
tr|Q8YAV5	Cardiolipin synthase OS=Listeria monocytogenes serovar 1/2a (strain ATCC BAA-679 / EGD-e) OX=169963 GN=lmo0008 P
DECOY sp|Q8Y789	DNA mismatch repair protein MutS OS=Listeria monocytogenes serovar 1/2a (strain ATCC BAA-679 / EGD-e) OX=169963
sp|Q8Y4I2	Phosphoglycerate kinase OS=Listeria monocytogenes serovar 1/2a (strain ATCC BAA-679 / EGD-e) OX=169963 GN=pgk PE
tr|Q8Y7L4	Lmo1263 protein OS=Listeria monocytogenes serovar 1/2a (strain ATCC BAA-679 / EGD-e) OX=169963 GN=lmo1263 PE=4 S
tr|Q8Y6V1	Universal stress protein OS=Listeria monocytogenes serovar 1/2a (strain ATCC BAA-679 / EGD-e) OX=169963 GN=lmo15

sp|Q8Y6T4	Tyrosine--tRNA ligase OS=Listeria monocytogenes serovar 1/2a (strain ATCC BAA-679 / EGD-e) OX=169963 GN=tyrS PE=
sp|Q8Y832	Ribitol-5-phosphate cytidylyltransferase OS=Listeria monocytogenes serovar 1/2a (strain ATCC BAA-679 / EGD-e) OX
sp|Q8Y757	Probable manganese-dependent inorganic pyrophosphatase OS=Listeria monocytogenes serovar 1/2a (strain ATCC BAA-6
tr|Q8YAM0	Lmo0098 protein OS=Listeria monocytogenes serovar 1/2a (strain ATCC BAA-679 / EGD-e) OX=169963 GN=lmo0098 PE=4 S
sp|Q8Y4F5	UvrABC system protein B OS=Listeria monocytogenes serovar 1/2a (strain ATCC BAA-679 / EGD-e) OX=169963 GN=uvrB P
sp|Q8YAC7	Bifunctional protein TilS/HprT OS=Listeria monocytogenes serovar 1/2a (strain ATCC BAA-679 / EGD-e) OX=169963 GN
tr|Q8Y6P6	Lmo1638 protein OS=Listeria monocytogenes serovar 1/2a (strain ATCC BAA-679 / EGD-e) OX=169963 GN=lmo1638 PE=1 S
sp|Q8Y5W8	GTPase Der OS=Listeria monocytogenes serovar 1/2a (strain ATCC BAA-679 / EGD-e) OX=169963 GN=der PE=3 SV=1
tr|Q8Y5K9	Lmo2050 protein OS=Listeria monocytogenes serovar 1/2a (strain ATCC BAA-679 / EGD-e) OX=169963 GN=lmo2050 PE=4 S
sp|Q8Y3L0	Phosphoserine aminotransferase OS=Listeria monocytogenes serovar 1/2a (strain ATCC BAA-679 / EGD-e) OX=169963 GN
tr|Q8Y9K6	Lmo0521 protein OS=Listeria monocytogenes serovar 1/2a (strain ATCC BAA-679 / EGD-e) OX=169963 GN=lmo0521 PE=3 S
tr|Q8Y8E3	Lmo0961 protein OS=Listeria monocytogenes serovar 1/2a (strain ATCC BAA-679 / EGD-e) OX=169963 GN=lmo0961 PE=4 S
DECOY_tr|Q8Y587	Lmo2182 protein OS=Listeria monocytogenes serovar 1/2a (strain ATCC BAA-679 / EGD-e) OX=169963 GN=lmo2182 PE=4 S
sp|Q8Y5F1	Putative heme-dependent peroxidase lmo2113 OS=Listeria monocytogenes serovar 1/2a (strain ATCC BAA-679 / EGD-e)
tr|Q8Y840	UTP--glucose-1-phosphate uridylyltransferase OS=Listeria monocytogenes serovar 1/2a (strain ATCC BAA-679 / EGD-e
tr|Q8Y4A4	Homoserine dehydrogenase OS=Listeria monocytogenes serovar 1/2a (strain ATCC BAA-679 / EGD-e) OX=169963 GN=hom P
tr|Q8YAA2	Lmo0252 protein OS=Listeria monocytogenes serovar 1/2a (strain ATCC BAA-679 / EGD-e) OX=169963 GN=lmo0252 PE=4 S
tr|Q8Y971	ThiD protein OS=Listeria monocytogenes serovar 1/2a (strain ATCC BAA-679 / EGD-e) OX=169963 GN=thiD PE=4 SV=1

Select Summary Report

Format As: Select Summary (protein hits) Help

Significance threshold p< 0.05 Max. number of hits AUTO

Standard scoring ○ MudPIT scoring ◉ Ions score or expect cut-off 17 Show sub-sets 0

Show pop-ups ◉ Suppress pop-ups ○ Require bold red ☑

Preferred taxonomy: All entries

Re-Search ◉ All queries ○ Unassigned ○ Below homology threshold ○ Below identity threshold

1. tr|Q8Y8K9 **Mass:** 31657 **Score:** 6430 **Matches:** 163(161) **Sequences:** 27(27) **emPAI:** 52.48

RsbR protein OS=Listeria monocytogenes serovar 1/2a (strain ATCC BAA-679 / EGD-e) OX=169963 GN=RsbR PE=4 SV=1

Query	Observed	Mr(expt)	Mr(calc)	ppm	Miss	Score	Expect	Rank	Unique	Peptide
413	379.729	757.443	757.445	-1.51	0	56	1e-05	1	U	R.ALALTNR.E 414 415 417
1140	419.203	836.392	836.392	0.29	0	45	7.9e-05	1	U	K.LDDFAEK.V 1138 1139 1141 1142 1143 1146
1745	440.227	878.439	878.439	0.19	0	53	2.3e-05	1	U	R.DEDLFLK.R 1738 1739 1743 1744 1746 1747 1748 1749 1750 1751
1796	441.727	881.440	881.440	0.30	0	47	6.3e-05	1	U	K.DFANFIR.T 1790 1791 1792 1793 1794 1795 1797 1798
3682	518.275	1034.535	1034.540	-4.34	1	56	9.1e-06	1	U	R.DEDLFLKR.E 3680 3683 3684
6413	622.827	1243.640	1243.641	-0.72	0	90	2.9e-09	1	U	K.QSDQLINDIAK.E 6402 6403 6410 6411 6412
6904	641.818	1281.622	1281.624	-1.77	0	78	4.6e-08	1	U	K.VVTAYADTWEK.T 6903
6958	643.352	1284.690	1284.690	-0.35	0	60	2.8e-06	1	U	R.VFGLLVYTAMR.D 6955 6956 6957 6959 6960 6961 6962 6965 6968
7468	440.885	1319.634	1319.633	0.85	1	74	9.5e-08	1	U	-.MYKDFANFIR.T 7467 7469
7524	662.433	1322.852	1322.854	-1.22	0	103	5.5e-11	1	U	K.LIIENLLIGVVK.N 7521 7522 7523 7525 7526 7527 7528 7529 7530 7531 7532 7533 7
7542	441.959	1322.856	1322.854	1.48	0	(58)	1.6e-06	1	U	K.LIIENLLIGVVK.N
2102	452.554	1354.639	1354.640	-1.02	1	76	7.2e-08	1	U	K.FNEKLDDFAEK.V 7936 7937
7934	678.327	1354.639	1354.640	-0.81	1	(62)	1.9e-06	1	U	K.FNEKLDDFAEK.V 7935
8026	681.829	1361.643	1361.644	-0.75	1	(56)	4.4e-06	1	U	-.MYKDFANFIR.T 8027
8561	472.210	1413.607	1413.605	1.62	0	84	4.1e-09	1	U	R.EEKPEDDAYYR.F 8557 8558 8562
8643	711.323	1420.632	1420.633	-0.90	0	56	4.2e-06	1	U	R.FETWLSSMYNK.V 8642
9632	508.337	1521.988	1521.986	1.69	1	69	1.2e-07	1	U	R.AKLIIENLLIGVVK.N 9630 9633
9915	772.916	1543.818	1543.828	-6.61	0	(78)	5.1e-08	1	U	K.ISVMPLIGTIDTER.A
10089	780.920	1559.825	1559.823	1.60	0	115	9.4e-12	1	U	K.ISVMPLIGTIDTER.A 10087 10088 10090 10091 10092 10093 10094 10095 10096
10209	524.243	1569.709	1569.706	1.75	1	73	5.8e-08	1	U	K.REEKPEDDAYYR.F 10208 10210
10513	532.007	1592.999	1592.998	0.87	1	88	1.7e-09	1	U	K.LIIENLLIGVVKNR.S
11157	820.850	1639.686	1639.686	-0.16	0	66	2.4e-07	1	U	K.ADLLNDWMNEMEK.Q 11155 11156
11158	547.570	1639.690	1639.686	2.28	0	(57)	2e-06	1	U	K.ADLLNDWMNEMEK.Q
12752	875.946	1749.878	1749.878	-0.19	0	93	1.7e-09	1	U	K.EFVDLIVSNVTENGSK.F 12751
12753	584.300	1749.878	1749.878	-0.05	0	(86)	8.5e-09	1	U	K.EFVDLIVSNVTENGSK.F 12754 12755 12756 12757 12759 12763
12812	878.498	1754.981	1754.982	-0.28	0	(89)	2.4e-09	1	U	K.SALQELSAPLLPIFEK.I 12803 12805 12806 12807 12809 12810 12813 12814 12819 12
12817	586.002	1754.983	1754.982	0.93	0	108	2.3e-11	1	U	K.SALQELSAPLLPIFEK.I 12808 12815 12816 12818 12822 12824 12825 12826
12880	587.323	1758.948	1758.955	-4.03	1	76	6.6e-08	1	U	K.ISVMPLIGTIDTERAK.L
15293	661.964	1982.871	1982.871	-0.42	1	19	0.013	1	U	R.TNKADLLNDWMNEMEK.Q
16113	1073.566	2145.118	2145.118	0.14	1	(45)	8.9e-05	1	U	R.VFGLLVYTAMRDEDLFLK.R
16117	716.047	2145.120	2145.118	1.04	1	50	2.8e-05	1	U	R.VFGLLVYTAMRDEDLFLK.R 16112 16115 16116
17776	804.798	2411.372	2411.368	1.84	1	93	4.8e-10	1	U	K.TVSIQKSALQELSAPLLPIFEK.I
20266	940.451	2818.331	2818.322	3.09	1	(35)	0.00051	1	U	K.EAMYEETSKEFVDLIVSNVTENGSK.F
20314	945.780	2834.317	2834.317	0.17	1	104	6.7e-11	1	U	K.EAMYEETSKEFVDLIVSNVTENGSK.F 20313
20316	709.587	2834.320	2834.317	1.12	1	(40)	0.00019	1	U	K.EAMYEETSKEFVDLIVSNVTENGSK.F
14701	956.117	2865.328	2865.316	4.12	1	38	0.00026	1	U	K.ADLLNDWMNEMEKQSDQLINDIAK.E
21038	804.436	3213.715	3213.707	2.69	0	123	5.5e-13	1	U	R.SEVVLIDITGVPVVDTMVAHHIIQASEAVR.L
21039	1072.246	3213.717	3213.707	3.25	0	(67)	2.2e-07	1	U	R.SEVVLIDITGVPVVDTMVAHHIIQASEAVR.L

2. sp|P02769 **Mass:** 71244 **Score:** 3725 **Matches:** 97(96) **Sequences:** 42(42) **emPAI:** 11.07

Serum albumin (Laboratory-Cont) OS=Bos taurus GN=ALB PE=1 SV=4

Query	Observed	Mr(expt)	Mr(calc)	ppm	Miss	Score	Expect	Rank	Unique	Peptide
410	379.716	757.416	757.416	1.07	0	46	0.00011	1	U	K.GACLLPK.I 409
682	395.238	788.462	788.464	-2.54	0	49	4.2e-05	1	U	K.LVTDLTK.V 681 683 684 685
1848	443.710	885.405	885.408	-3.53	0	57	3.2e-06	1	U	K.DDSPDLPK.L 1849
2039	449.745	897.475	897.474	0.37	0	35	0.00077	1	U	R.LCVLHEK.T
2346	461.747	921.480	921.481	-0.56	0	47	5.7e-05	1	U	K.AEFVEVTK.L 2344 2345
2397	464.250	926.485	926.486	-0.78	0	36	0.00055	1	U	K.YLYEIAR.R 2394 2398 2399
2967	487.735	973.455	973.451	4.26	0	31	0.0012	1	U	K.DLGEEHFK.G 2966
3284	501.794	1001.574	1001.576	-1.78	0	32	0.0024	1	U	K.LVVSTQTALA.- 3283
3443	507.814	1013.613	1013.612	0.86	0	64	5.8e-07	1	U	K.QTALVELLK.H
4191	536.758	1071.501	1071.502	-0.96	0	56	4.5e-06	1	U	K.SHCIAEVEK.D
4644	554.262	1106.509	1106.507	2.07	0	67	4.8e-07	1	U	K.EACFAVEGPK.L
5057	569.750	1137.486	1137.491	-3.72	0	59	1.3e-06	1	U	K.CCTESLVNR.R

5112	571.861	1141.707	1141.707	-0.44	1	76	2.8e-08	1	U	K.KQTALVELLK.H 5111
5374	582.319	1162.623	1162.623	-0.31	0	75	1.1e-07	1	U	K.LVNELTEFAK.T 5368 5369 5371 5372 5373 5375 5376 5377 5378
6477	417.212	1248.615	1248.614	0.62	1	50	3.3e-05	1	U	R.FKDLGEEHFK.G
6926	642.358	1282.701	1282.703	-1.77	0	66	6.4e-07	1	U	R.HPEYAVSVLLR.L 6927
7038	646.304	1290.594	1290.595	-0.93	0	31	0.0015	1	U	K.ECCDKPLLEK.S
7040	431.207	1290.599	1290.595	3.09	0	(27)	0.004	1	U	K.ECCDKPLLEK.S
7260	653.361	1304.707	1304.709	-1.20	0	72	2e-07	1	U	K.HLVDEPQNLIK.Q 7253 7254 7257 7258 7261 7262
8577	708.346	1414.678	1414.680	-1.67	0	85	8.3e-09	1	U	K.TVMENFVAFVDK.C 8578 8579
8623	710.349	1418.682	1418.686	-2.76	0	(19)	0.033	1	U	K.SLHTLFGDELCK.V
8624	473.902	1418.685	1418.686	-0.64	0	69	3.7e-07	1	U	K.SLHTLFGDELCK.V
8831	480.609	1438.805	1438.804	0.60	1	92	1.4e-09	1	U	R.RHPEYAVSVLLR.L 8830
8875	722.325	1442.635	1442.635	0.48	0	72	6.9e-08	1	U	K.YICDNQDTISSK.L 8874
9066	488.535	1462.583	1462.582	0.63	0	74	4e-08	1	U	K.TCVADESHAGCEK.S
9225	740.402	1478.790	1478.788	0.96	0	125	8.6e-13	1	U	K.LGEYGFQNALIVR.Y 9223 9224 9226
9375	747.762	1493.509	1493.511	-1.31	0	72	5.8e-08	1	U	R.ETYGDMADCCEK.Q
9460	751.811	1501.607	1501.606	0.60	0	82	6.4e-09	1	U	K.EYEATLEECCAK.D
9549	756.424	1510.833	1510.836	-1.36	0	90	2e-09	1	U	K.VPQVSTPTLVEVSR.S 9548
9791	511.599	1531.774	1531.774	0.33	1	69	3.6e-07	1	U	K.LKECCDKPLLEK.S
10160	784.375	1566.736	1566.735	0.41	0	94	7.9e-10	1	U	K.DAFLGSFLYEYSR.R 10157 10158 10159 10161 10162 10163 10165
10305	526.261	1575.762	1575.760	1.29	0	83	1.4e-08	1	U	K.LKPDPNTLCDEFK.A 10304
11153	547.317	1638.929	1638.930	-0.87	1	109	1.7e-11	1	U	R.KVPQVSTPTLVEVSR.S 11145 11149 11150
12648	580.948	1739.822	1739.822	-0.01	0	64	1e-06	1	U	R.MPCTEDYLSLILNR.L
12707	874.356	1746.697	1746.698	-0.39	0	86	2.8e-09	1	U	K.YNGVFQECCQAEDK.G
14320	470.986	1879.913	1879.914	-0.26	0	71	2.3e-07	1	U	R.RPCFSALTPDETYVPK.A
14321	940.964	1879.914	1879.914	0.14	0	(38)	0.00041	1	U	R.RPCFSALTPDETYVPK.A
14322	627.645	1879.915	1879.914	0.40	0	(57)	5.7e-06	1	U	R.RPCFSALTPDETYVPK.A
14597	634.628	1900.862	1900.863	-0.18	1	48	2e-05	1	U	R.NECFLSHKDDSPDLPK.L
14652	636.643	1906.908	1906.914	-2.95	0	86	5.8e-09	1	U	K.LFTFHADICTLPDTEK.Q
14867	643.271	1926.791	1926.791	-0.26	1	118	1.6e-12	1	U	K.CCAADDKEACFAVEGPK.L 14866
15106	978.483	1954.952	1954.952	-0.23	0	75	8.2e-08	1	U	K.DAIPENLPPLTADFAEDK.D
15528	505.749	2018.965	2018.962	1.79	1	87	5e-09	1	U	K.LKPDPNTLCDEFKADEK.K
16723	749.988	2246.941	2246.935	2.61	1	35	0.00035	1	U	K.ECCHGDLLECADDRADLAK.Y
18135	820.066	2457.175	2457.173	0.73	1	106	5.3e-11	1	U	K.DAIPENLPPLTADFAEDKDVCK.N 18134
11467	831.423	2491.248	2491.257	-3.57	0	73	3.5e-07	1	U	K.GLVLIAFSQYLQQCPFDEHVK.L

3. sp|P04264 **Mass:** 66170 **Score:** 1906 **Matches:** 45(44) **Sequences:** 29(29) **emPAI:** 5.37
Keratin, type II cytoskeletal 1 (Contact-Cont) OS=Homo sapiens GN=KRT1 PE=1 SV=6

Query	Observed	Mr(expt)	Mr(calc)	ppm	Miss	Score	Expect	Rank	Unique	Peptide
35	352.695	703.375	703.375	-0.61	0	28	0.01	1	U	R.LDSELK.N
795	402.703	803.392	803.402	-13.66	0	27	0.0071	1	U	R.SEIDNVK.K
1084	416.750	831.485	831.481	4.58	0	32	0.0018	1	U	K.SISISVAR.G
1689	437.752	873.490	873.492	-2.04	0	49	7.2e-05	1	U	R.SLVNLGGSK.S 1690
2955	487.269	972.524	972.524	0.39	0	67	7.6e-07	1		K.IEISELNR.V 2953 2954
3444	508.223	1014.431	1014.433	-1.60	0	48	1.8e-05	1	U	K.DVDGAYMTK.V
3648	517.262	1032.510	1032.509	1.38	0	61	7.5e-06	1	U	R.TLLEGEESR.M 3647 3650
4104	533.265	1064.515	1064.514	1.01	0	59	5.5e-06	1	U	K.AQYEDIAQK.S 4103
4114	356.177	1065.510	1065.509	1.33	1	28	0.0069	1		K.YEDEINKR.T
4883	563.274	1124.534	1124.535	-1.08	0	69	4.1e-07	1	U	K.AEAESLYQSK.Y
5291	579.260	1156.506	1156.507	-1.01	0	41	0.00011	1	U	R.DYQELMNTK.L
6685	633.320	1264.626	1264.630	-2.99	0	75	1.1e-07	1	U	R.TNAENEFVTIK.K
6851	639.358	1276.702	1276.703	-0.31	0	64	8e-07	1	U	K.LALDLEIATYR.T
7187	434.906	1301.696	1301.694	1.30	1	42	0.00028	1	U	R.NSKIEISELNR.V
7189	651.860	1301.706	1301.708	-1.75	0	66	1.2e-06	1	U	R.SLDLDSIIAEVK.A 7188 7190
7656	666.761	1331.507	1331.512	-4.07	0	64	3.8e-07	1	U	K.NMQDMVEDYR.N 7657
7760	670.836	1339.657	1339.662	-3.69	1	91	2.7e-09	1	U	K.SKAEAESLYQSK.Y
7971	679.351	1356.687	1356.688	-1.11	0	97	7.3e-10	1	U	K.LNDLEDALQQAK.E 7966 7970 7972
8244	692.350	1382.685	1382.683	1.09	0	91	2.2e-09	1	U	K.SLNNQFASFIDK.V 8243
8355	465.250	1392.727	1392.725	1.73	1	91	3e-09	1	U	R.TNAENEFVTIKK.D
9185	738.378	1474.741	1474.742	-0.16	0	72	2.1e-07	1	U	K.WELLQQVDTSTR.T
9187	738.394	1474.774	1474.778	-2.45	0	88	4.7e-09	1		R.FLEQQNQVLQTK.W 9188 9189
10574	800.419	1598.824	1598.826	-1.59	1	97	6e-10	1	U	K.NKLNDLEDALQQAK.E
11092	546.962	1637.864	1637.853	6.70	1	49	2.9e-05	1	U	K.SLNNQFASFIDKVR.F
12387	858.929	1715.843	1715.844	-0.60	0	121	2.7e-12	1	U	K.QISNLQQSISDAEQR.G
13020	883.376	1764.738	1764.727	6.07	0	126	2.7e-13	1	U	R.FSSCGGGGGSFGAGGGFGSR.S
15366	665.332	1992.974	1992.969	2.20	0	89	3.3e-09	1	U	R.THNLEPYFESFINNLR.R 15365
17580	795.325	2382.952	2382.945	3.04	0	108	1.6e-11	1	U	R.GGGGGGYGSGGSSYGSGGGSYGSGGGGGGGR.G
18676	861.058	2580.152	2580.154	-1.05	0	136	2.9e-14	1	U	R.MSGECAPNVSVSVSTSHTTISGGGSR.G

4. sp|P13645 **Mass:** 59020 **Score:** 1738 **Matches:** 36(36) **Sequences:** 25(25) **emPAI:** 4.97
Keratin, type I cytoskeletal 10 (Contact-Cont) OS=Homo sapiens GN=KRT10 PE=1 SV=6

Query	Observed	Mr(expt)	Mr(calc)	ppm	Miss	Score	Expect	Rank	Unique	Peptide
34	352.694	703.373	703.375	-2.68	0	30	0.0057	1	U	K.TIDDLK.N
822	404.203	806.392	806.392	-0.26	0	50	4.2e-05	1		R.LAADDFR.L 820 821 823
847	405.223	808.432	808.433	-0.79	0	41	0.0002	1		R.LASYLDK.V
1340	424.229	846.444	846.445	-0.34	0	44	0.00023	1	U	K.SEITELR.R 1339
3636	516.303	1030.591	1030.591	0.14	0	72	2.4e-07	1		R.VLDELTLTK.A 3635 3637
4088	532.799	1063.584	1063.603	-17.26	1	33	0.0018	1	U	R.LASYLDKVR.A
4631	553.766	1105.517	1105.519	-1.53	0	77	6.5e-08	1	U	K.VTMQNLNDR.L
4674	555.257	1108.499	1108.483	15.0	0	33	0.001	1	U	K.DAEAWFNEK.S
5394	583.295	1164.575	1164.577	-2.44	0	75	3.1e-07	1	U	R.LENEIQTYR.S 5395
5830	601.311	1200.607	1200.610	-2.10	0	101	2.7e-10	1		R.QSVEADINGLR.R
6647	631.802	1261.590	1261.590	0.42	0	49	3.1e-05	1	U	R.SLLEGEGSSGGGGR.G
7980	453.245	1356.712	1356.711	0.53	1	52	2.4e-05	1		R.QSVEADINGLRR.V
8060	683.321	1364.628	1364.632	-3.00	0	61	1.7e-06	1	U	R.SQYEQLAEQNR.K

8220	691.327	1380.640	1380.641	-0.75	0	83	1.1e-08	1	U	R.ALEESNYELEGK.I 8219
8311	695.843	1389.672	1389.674	-1.15	0	90	2.9e-09	1	U	K.QSLEASLAETEGR.Y
8765	717.881	1433.748	1433.763	-10.35	1	58	4.6e-06	1	U	K.IRLENEIQTYR.S
9364	498.582	1492.725	1492.727	-1.15	1	62	2e-06	1	U	R.SQYEQLAEQNRK.D
12235	854.393	1706.771	1706.765	3.30	0	158	3.5e-16	1	U	K.GSLGGGFSSGGFSGGSFSR.G
15387	998.990	1995.965	1995.964	0.78	0	108	3.6e-11	1	U	K.ELTTEIDNNIEQISSYK.S
15820	694.993	2081.958	2081.958	0.11	0	60	2e-06	1	U	R.AETECQNTEYQQLLDIK.I
15970	704.685	2111.034	2111.034	-0.12	0	88	4.6e-09	1	U	K.ADLEMQIESLTEELAYLK.K 15969
16503	738.038	2211.091	2211.091	0.09	1	(26)	0.0032	1	U	K.SKELTTEIDNNIEQISSYK.S
16505	1106.553	2211.092	2211.091	0.55	1	109	1.6e-11	1	U	K.SKELTTEIDNNIEQISSYK.S
16682	747.384	2239.130	2239.129	0.17	1	105	8.7e-11	1	U	K.ADLEMQIESLTEELAYLKK.N 9370
20505	968.799	2903.376	2903.375	0.30	0	96	5e-10	1	U	R.NVSTGDVNVEMNAAPGVDLTQLLNNMR.S
15611	1018.218	3051.633	3051.620	4.42	1	109	1.7e-11	1	U	K.TIDDLKNQILNLTTDNANILLQIDNAR.L

---

5. sp|Q9AGE6 **Mass:** 57332 **Score:** 1708 **Matches:** 32(32) **Sequences:** 25(25) **emPAI:** 5.29

60 kDa chaperonin OS=Listeria monocytogenes serovar 1/2a (strain ATCC BAA-679 / EGD-e) OX=169963 GN=groL PE=3 SV=1

Query	Observed	Mr(expt)	Mr(calc)	ppm	Miss	Score	Expect	Rank	Unique	Peptide
17	351.216	700.417	700.412	7.06	0	32	0.0031	1	U	R.NVVLEK.K 18
179	360.177	718.339	718.340	-1.61	0	40	0.0003	1	U	K.APGFGDR.R 178
880	407.262	812.510	812.512	-2.96	0	56	5.2e-06	1	U	K.LAGGVAVVK.V
1041	415.226	828.438	828.434	5.18	0	24	0.0072	1	U	R.SLEEPVR.Q
1081	416.741	831.468	831.470	-2.79	0	48	6e-05	1	U	K.LVSEVASK.T
2491	467.771	933.528	933.528	-0.55	0	67	4.9e-07	1	U	R.GTFNVVAVK.A 2490
2667	474.743	947.471	947.475	-3.83	0	33	0.0018	1	U	K.LIAEAMER.V
3478	509.762	1017.509	1017.509	0.18	0	65	1.2e-06	1	U	R.IEDALNSTR.A
3482	509.775	1017.536	1017.534	1.67	0	77	8.1e-08	1	U	K.VGAATETELK.E
5255	577.816	1153.618	1153.620	-1.93	0	58	4e-06	1	U	K.NVTAGANPVGVR.R
6101	609.820	1217.625	1217.625	-0.54	0	78	8.5e-08	1	U	K.TATVDQLGTANK.V 6102 6103
7203	435.234	1302.679	1302.678	1.06	1	113	1.6e-11	1	U	K.VGAATETELKER.K
8011	680.850	1359.685	1359.688	-2.30	0	97	7.9e-10	1	U	R.VGNDGVITIEESK.G
9023	730.310	1458.605	1458.593	7.82	0	61	8e-07	1	U	R.AQMEETTSEFDR.E
9793	766.918	1531.822	1531.825	-1.52	0	76	7.9e-08	1	U	K.FGSPLITNDGVTIAK.E
11022	819.373	1636.731	1636.729	1.02	0	67	3.1e-07	1	U	K.EIELEDPFENMGAK.L
12006	564.974	1691.899	1691.895	1.93	0	106	5.1e-11	1	U	R.QIAHNAGLEGSVIVER.L
12381	572.919	1715.734	1715.731	1.82	1	83	4.7e-09	1	U	R.AQMEETTSEFDREK.L
13259	895.444	1788.874	1788.874	0.10	0	137	7.8e-14	1	U	K.ESIAQVAAISSGDEEVGK.L 13258
13667	610.618	1828.831	1828.830	0.61	0	(36)	0.00027	1	U	K.GFATELDVVEGMQFDR.G
13668	915.424	1828.833	1828.830	1.74	0	90	1.1e-09	1	U	K.GFATELDVVEGMQFDR.G
13724	918.434	1834.854	1834.854	-0.47	0	132	1.8e-13	1	U	K.DDTTIVEGAGDSTQISAR.V
15521	673.698	2018.072	2018.068	1.82	0	107	4.2e-11	1	U	R.AAVEEGIVAGGGTALVSIYNK.V
17424	788.076	2361.205	2361.202	1.11	1	100	2.5e-10	1	U	K.VVVTKDDTTIVEGAGDSTQISAR.V
18474	841.089	2520.245	2520.238	2.69	0	94	9.6e-10	1	U	K.TNDVAGDGTTTATVLAQAMIQEGLK.N
18581	853.461	2557.362	2557.356	2.24	0	48	2.7e-05	1	U	K.AMLEDIAILTGGQVITEDLGLELK.T

---

6. sp|P35527 **Mass:** 62255 **Score:** 1303 **Matches:** 26(25) **Sequences:** 21(21) **emPAI:** 3.15

Keratin, type I cytoskeletal 9 (Contact-Cont) OS=Homo sapiens GN=KRT9 PE=1 SV=3

Query	Observed	Mr(expt)	Mr(calc)	ppm	Miss	Score	Expect	Rank	Unique	Peptide
334	373.214	744.413	744.413	0.10	0	27	0.012	1	U	K.EVTQLR.H
847	405.223	808.432	808.433	-0.79	0	41	0.0002	1		R.LASYLDK.V
2226	457.210	912.406	912.401	5.73	0	46	7e-05	1	U	R.MTLDDFR.I 2224
4014	530.785	1059.555	1059.556	-0.81	0	65	1.2e-06	1	U	K.TLLDIDNTR.M
4335	541.750	1081.485	1081.486	-0.97	0	18	0.027	1	U	K.FEMEQNLR.Q
5299	579.298	1156.581	1156.584	-2.20	0	91	2.4e-09	1	U	R.QGVDADINGLR.Q 5298 5300
5912	603.804	1205.593	1205.596	-3.01	0	58	6.6e-06	1	U	R.QVLDNLTMEK.S
6246	616.801	1231.587	1231.591	-2.69	0	130	1.6e-13	1	U	R.SGGGGGGGLGSGGSIR.S
6305	618.267	1234.519	1234.521	-2.05	0	90	1e-09	1	U	R.FSSSSGYGGGSSR.V
7412	658.339	1314.663	1314.678	-11.56	0	46	5.8e-05	1	U	K.DQIVDLTVGNNK.T
7513	441.895	1322.664	1322.665	-0.65	1	82	2.3e-08	1	U	R.IKFEMEQNLR.Q 7512
13279	896.369	1790.724	1790.720	1.88	0	97	1.8e-10	1	U	R.GGSGGSYGGGGSGGGYGGGSGSR.G
13758	613.331	1836.970	1836.958	6.73	0	88	2.3e-09	1	U	R.HGVQELEIELQSQLSK.K
14167	934.466	1866.917	1866.915	1.08	1	29	0.01	1	U	K.TLNDMRQEYEQLIAK.N 14168
16456	735.010	2202.007	2202.007	0.12	0	23	0.0062	1	U	K.SDLEMQYETLQEELMALK.K
17306	777.709	2330.104	2330.102	0.95	1	79	2.7e-08	1	U	K.SDLEMQYETLQEELMALKK.N
18432	837.385	2509.132	2509.124	2.94	0	128	1.8e-13	1	U	K.EIETYHNLLEGGQEDFESSGAGK.I
19478	902.393	2704.156	2704.154	0.81	0	58	1.6e-06	1	U	R.GGGGSFGYSYGGGSGGGFSASSLGGGFGGGSR.G
20502	968.144	2901.409	2901.403	1.88	1	129	3.1e-13	1	U	K.NYSPYYNTIDDLKDQIVDLTVGNNK.T
16146	1075.106	3222.297	3222.274	7.05	0	51	1.5e-05	1	U	R.GGSGGSHGGGSGFGGESGGSYGGGEEASGSGGGYGGGSGK.S
21096	816.884	3263.507	3263.507	0.03	0	114	4.3e-12	1	U	K.DIENQYETQITQIEHEVSSSGQEVQSSAK.E

---

7. sp|P35908 **Mass:** 65678 **Score:** 1245 **Matches:** 36(35) **Sequences:** 27(27) **emPAI:** 4.67

Keratin, type II cytoskeletal 2 epidermal (Contact-Cont) OS=Homo sapiens GN=KRT2 PE=1 SV=2

Query	Observed	Mr(expt)	Mr(calc)	ppm	Miss	Score	Expect	Rank	Unique	Peptide
1020	414.219	826.423	826.423	0.23	0	40	0.00024	1		K.FASFIDK.V
1074	416.249	830.483	830.486	-3.36	0	42	0.00025	1	U	R.SLVGLGGTK.S
2955	487.269	972.524	972.524	0.39	0	67	7.6e-07	1		K.IEISELNR.V 2953 2954
3327	503.236	1004.457	1004.460	-2.47	0	46	4.8e-05	1	U	K.LLEGEECR.M
3705	519.266	1036.518	1036.519	-1.31	0	37	0.00066	1	U	R.YLDGLTAER.T 3706
3766	521.281	1040.547	1040.550	-3.47	0	43	0.00012	1	U	K.VDPEIQNVK.A
4114	356.177	1065.510	1065.509	1.33	1	28	0.0069	1		K.YEDEINKR.T
4358	542.755	1083.496	1083.491	4.91	0	52	9.7e-06	1	U	K.DVDNAYMIK.V
4649	554.274	1106.533	1106.536	-2.68	0	42	0.0002	1	U	K.AQYEEIAQR.S 4648
4964	566.261	1130.508	1130.503	4.60	0	53	6.4e-06	1	U	R.STSSFSCLSR.H
5264	578.276	1154.538	1154.528	8.45	0	40	0.00039	1	U	R.DYQELMNVK.L 5263

5735	597.311	1192.608	1192.609	-0.58	0	61	4.5e-06	1	U	K.YEELQVTVGR.H
5786	599.276	1196.538	1196.542	-3.31	0	48	4.8e-05	1	U	K.GGSISGGGYGSGGGK.H
5952	604.810	1207.606	1207.608	-1.93	0	78	7.7e-08	1	U	R.TAAENDFVTLK.K
6561	627.807	1253.599	1253.600	-0.96	0	68	7.5e-07	1	U	R.GFSSGSAVVSGGSR.R
7463	440.867	1319.580	1319.576	3.38	0	36	0.00026	1	U	R.HGGGGGGFGGGGFGSR.S
7629	665.326	1328.637	1328.632	3.65	0	46	0.00018	1	U	K.NVQDAIADAEQR.G
7634	665.365	1328.716	1328.719	-2.13	0	76	8.4e-08	1	U	R.NLDLDSIIAEVK.A 7637
7703	668.846	1335.677	1335.703	-19.96	1	36	0.00069	1	U	R.TAAENDFVTLKK.D
8121	686.360	1370.705	1370.704	0.97	0	73	1.2e-07	1	U	K.LNDLEEALQQAK.E
8320	464.565	1390.675	1390.673	1.28	1	63	1.8e-06	1	U	R.SKEEAEALYHSK.Y
9041	730.904	1459.793	1459.792	0.65	0	89	5.5e-09	1	U	K.VDLLNQEIEFLK.V 9040
9187	738.394	1474.774	1474.778	-2.45	0	88	4.7e-09	1		R.FLEQQNQVLQTK.W 9188 9189
10687	807.431	1612.848	1612.842	3.64	1	84	8.6e-09	1	U	R.NKLNDLEEALQQAK.E
16915	758.017	2271.029	2271.032	-1.40	1	28	0.0014	1	U	R.TSQNSELNNMQDLVEDYKK.K
17679	800.348	2398.022	2398.011	4.59	0	54	4e-06	1	U	R.GGGFGGGSSFGGGSGFSGGGFGGGGFGGGR.F
20761	1022.168	3063.484	3063.482	0.57	0	78	2.1e-08	1	U	K.VLYDAEISQIHQSVTDTNVILSMDNSR.N

8. sp|P00761 **Mass:** 25078 **Score:** 1215 **Matches:** 39(38) **Sequences:** 3(3) **emPAI:** 0.65
Trypsin (Laboratory-Cont) OS=Sus scrofa PE=1 SV=1

Query	Observed	Mr(expt)	Mr(calc)	ppm	Miss	Score	Expect	Rank	Unique	Peptide
1268	421.758	841.501	841.502	-1.03	0	61	2e-06	1	U	R.VATVSLPR.S 1251 1253 1254 1255 1256 1259 1260 1262 1264 1265 1266 1267 1269 12
3834	523.283	1044.551	1044.556	-4.85	0	63	2e-06	1	U	K.LSSPATLNSR.V 3832 3833 3835 3840 3841 3842 3845 3846
13301	598.628	1792.861	1792.857	2.50	0	32	0.0018	1	U	K.VCNYVNWIQQTIAAN.-

9. sp|P0DJM2 **Mass:** 66104 **Score:** 1083 **Matches:** 24(23) **Sequences:** 18(18) **emPAI:** 2.16
Chaperone protein DnaK OS=Listeria monocytogenes serovar 1/2a (strain ATCC BAA-679 / EGD-e) OX=169963 GN=dnaK PE=3 SV=1

Query	Observed	Mr(expt)	Mr(calc)	ppm	Miss	Score	Expect	Rank	Unique	Peptide
1528	431.235	860.456	860.460	-4.94	0	30	0.0072	1	U	R.NTTIPTSK.S
1855	443.753	885.491	885.492	-1.44	0	50	3.3e-05	1	U	K.IAGLEVER.I
2864	483.770	965.525	965.529	-4.51	0	43	0.0001	1	U	K.IIPNPEGAR.T
3242	499.796	997.578	997.581	-2.56	0	43	6.7e-05	1	U	R.IPAVQETIK.K
5974	605.839	1209.663	1209.665	-1.55	0	57	4.2e-06	1	U	K.IIDYLVAEFK.K
6162	612.839	1223.664	1223.666	-1.60	0	47	4.3e-05	1	U	R.FQLADIPPAPR.G 6159 6160 6161
7636	665.369	1328.723	1328.730	-5.09	0	94	1.4e-09	1	U	R.AAITNPNTISSIK.R
8041	682.345	1362.676	1362.678	-1.20	0	79	3.5e-08	1	U	R.NNADQLVFTVDK.T
2187	455.241	1362.703	1362.678	18.0	0	(18)	0.19	1	U	R.NNADQLVFTVDK.T
9296	495.948	1484.822	1484.831	-6.25	1	39	0.00036	1	U	R.AAITNPNTISSIKR.H
10133	782.910	1563.806	1563.805	1.07	0	(44)	0.00014	1	U	K.AVITVPAYFNDAQR.Q
3800	522.277	1563.808	1563.805	2.01	0	74	3.2e-07	1	U	K.AVITVPAYFNDAQR.Q
10239	524.938	1571.791	1571.794	-1.91	1	86	1e-08	1	U	R.AKFDELTHDLVER.T
10241	393.957	1571.799	1571.794	2.81	1	(83)	1.9e-08	1	U	R.AKFDELTHDLVER.T
10266	787.424	1572.834	1572.836	-1.34	0	50	3.9e-05	1	U	K.TESLNEIVQNLSVK.L
10502	531.907	1592.699	1592.699	0.21	1	116	2.3e-12	1	U	K.MVQDAEANAEEDKK.N
14327	941.004	1879.992	1879.993	-0.34	0	72	1.8e-07	1	U	K.DYSPQEISAIILQYLK.S
15580	1016.018	2030.021	2030.028	-3.70	0	113	1.1e-11	1	U	K.DANLSASDIDQVILVGGSTR.I
16025	710.057	2127.148	2127.142	2.54	0	78	3.4e-08	1	U	K.IIGIDLGTTNSAVAVLEGGEAK.I
18226	824.439	2470.296	2470.303	-2.91	1	67	2.6e-07	1	U	R.QALKDANLSASDIDQVILVGGSTR.I
20552	976.482	2926.425	2926.424	0.36	0	82	1.1e-08	1	U	K.SQTFSTAADNQPAVDIHVLQGERPMAK.D

10. sp|Q8Y422 **Mass:** 43429 **Score:** 1055 **Matches:** 23(23) **Sequences:** 14(14) **emPAI:** 2.89
Elongation factor Tu OS=Listeria monocytogenes serovar 1/2a (strain ATCC BAA-679 / EGD-e) OX=169963 GN=tuf PE=1 SV=1

Query	Observed	Mr(expt)	Mr(calc)	ppm	Miss	Score	Expect	Rank	Unique	Peptide
2373	462.754	923.493	923.496	-3.89	0	33	0.0017	1	U	K.AETYVLTK.E 2372
4975	566.322	1130.630	1130.630	0.30	0	74	1.6e-07	1	U	R.TVGAGVVSNISK.- 4976
5220	576.807	1151.600	1151.601	-0.85	0	61	2.6e-06	1	U	K.VVVTGVEMFR.K
7205	652.395	1302.775	1302.776	-1.04	0	63	4.9e-07	1	U	K.TTLTAAITTVLAK.K
12194	852.452	1702.889	1702.889	0.06	0	103	1.7e-10	1	U	K.LLDYAEAGDNIGALLR.G 12193
12877	587.303	1758.886	1758.889	-1.26	1	91	2.7e-09	1	U	K.VGDEVEVIGIEEESKK.V 12875 12878
13137	593.295	1776.862	1776.864	-1.04	0	83	1.7e-08	1	U	R.GITISTAHVEYQTDSR.H
13335	449.744	1794.946	1794.949	-1.42	0	22	0.021	1	U	R.SKPHVNIGTIGHVDHGK.T
15156	982.498	1962.982	1962.983	-0.39	0	93	1.5e-09	1	U	R.DLLTEYEFPGDDIPVIK.G 15155 15157
15183	984.939	1967.864	1967.850	7.37	0	131	8.7e-14	1	U	K.GYADAQAYDQIDGAPEER.E
15363	665.319	1992.935	1992.935	-0.02	0	31	0.0014	1	U	K.IDELMEAVDSYIPTPER.D
16531	739.679	2216.014	2216.013	0.39	0	65	5e-07	1	U	R.DTDKPFMMPVEDVFSITGR.G 16532
16533	1109.017	2216.020	2216.013	3.27	0	(43)	0.00011	1	U	R.DTDKPFMMPVEDVFSITGR.G
16905	757.335	2268.984	2268.980	1.86	0	81	8.2e-09	1	U	K.CDMVDDEELLELVEMEIR.D
19966	921.775	2762.302	2762.304	-0.48	0	118	2e-12	1	U	K.NMITGAAQMDGAILVVSAADGPMPQTR.E

11. tr|Q8Y6W1 **Mass:** 62673 **Score:** 924 **Matches:** 22(22) **Sequences:** 21(21) **emPAI:** 3.11
Pyruvate kinase OS=Listeria monocytogenes serovar 1/2a (strain ATCC BAA-679 / EGD-e) OX=169963 GN=pykA PE=3 SV=1

Query	Observed	Mr(expt)	Mr(calc)	ppm	Miss	Score	Expect	Rank	Unique	Peptide
268	367.208	732.402	732.402	-0.32	0	23	0.025	1	U	K.DATSLVK.D
745	400.747	799.480	799.480	0.02	0	35	0.00098	1	U	K.IQLIGEK.V
1221	420.269	838.523	838.528	-5.59	0	28	0.0016	1	U	K.VLNPGVLK.N
1526	431.235	860.455	860.460	-5.67	0	43	0.00036	1	U	K.ESLASGVAK.Q
3145	495.275	988.536	988.544	-8.03	0	50	6.2e-05	1	U	R.ATDVLEITK.I
3265	500.795	999.576	999.596	-20.10	0	58	4e-06	1	U	K.QVAILLDTK.G
3594	514.802	1027.590	1027.591	-1.43	0	54	1.2e-05	1	U	K.AEEGGILIVK.T
4082	532.767	1063.519	1063.522	-3.24	0	58	4.1e-06	1	U	R.VSMTPVEGTK.E
4971	566.296	1130.578	1130.582	-3.65	0	48	4.3e-05	1	U	R.TEEVLVAQDK.F
5366	582.308	1162.601	1162.598	2.46	0	69	5.3e-07	1	U	K.LEFQTGDVVR.V
8327	696.368	1390.722	1390.734	-8.92	1	63	2.1e-06	1	U	K.TTDKEILPAFEK.S

8330	464.585	1390.733	1390.734	-1.32	1	(21)	0.032	1	U	K.TTDKEILPAFEK.S
3690	518.308	1551.901	1551.898	1.93	1	31	0.0022	1	U	K.QVAILLDTKGPEIR.T
13062	885.467	1768.920	1768.915	2.88	0	128	4.5e-13	1	U	R.FGLEQGIDFIAASFVR.R
14149	933.959	1865.904	1865.908	-2.38	0	95	8.7e-10	1	U	R.LATPVSNTDEMFDLAVK.E
14197	624.339	1869.996	1870.005	-4.92	1	83	8.6e-09	1	U	K.SNAEALEKAEEGGILIVK.T
14324	940.980	1879.945	1879.986	-22.27	0	52	2.1e-05	1	U	K.NLNVQTIVAATQSGHTAR.M
14795	641.000	1919.977	1919.981	-1.79	0	68	5.8e-07	1	U	K.LGKPVITATQMLDSMQR.N
16075	535.510	2138.011	2138.006	2.09	0	65	8.2e-07	1	U	K.LHENTDMTEAIGQAVGHTAK.N
10264	787.416	2359.226	2359.230	-2.04	0	51	7e-05	1	U	K.QGDLIIITAGVPVTESGTTNVMK.I
19304	896.115	2685.322	2685.328	-2.18	0	85	5.9e-09	1	U	K.IENQEGVDNIDEILQVSQGLMVAR.G
20386	953.823	2858.448	2858.452	-1.40	0	80	1.9e-08	1	U	K.IVCTIGPASESVDTLVQLIEAGMNVAR.L

12. sp|Q927L9 **Mass:** 19983 **Score:** 692 **Matches:** 18(18) **Sequences:** 11(11) **emPAI:** 8.91
50S ribosomal protein L5 OS=Listeria monocytogenes serovar 1/2a (strain ATCC BAA-679 / EGD-e) OX=169963 GN=rplE PE=3 SV=1

Query	Observed	Mr(expt)	Mr(calc)	ppm	Miss	Score	Expect	Rank	Unique	Peptide
471	382.705	763.396	763.398	-1.92	0	40	0.0004	1	U	K.NSIAGFR.L
1755	440.237	878.459	878.461	-2.28	0	34	0.0018	1	U	R.GNYTLGVR.E
1833	442.782	883.549	883.549	-0.20	0	49	1.6e-05	1	U	K.LVTVSLPR.V 1834
2646	474.213	946.411	946.411	0.08	0	55	3.1e-06	1	U	R.MYDFLDK.L 2647
3295	502.277	1002.539	1002.542	-2.49	0	33	0.0025	1	U	K.EIVPALMSK.F 3296
5181	575.313	1148.611	1148.611	0.29	0	32	0.0017	1	U	R.GMDVVIVTTAK.S
7821	672.810	1343.606	1343.607	-0.24	0	57	2.4e-06	1	U	K.FNYDSVMEVPK.I 7822
8881	722.393	1442.772	1442.773	-0.86	0	107	7.1e-11	1	U	K.IVINTGVGDATANAK.V 8880
8883	481.932	1442.774	1442.773	0.42	0	(93)	1.5e-09	1	U	K.IVINTGVGDATANAK.V
13626	912.457	1822.899	1822.899	-0.18	0	56	7e-06	1	U	R.EQLIFPEIDYDQVSK.V
13627	608.642	1822.905	1822.899	3.08	0	(47)	5.4e-05	1	U	R.EQLIFPEIDYDQVSK.V
15938	702.334	2103.979	2103.978	0.29	0	70	3.3e-07	1	U	K.SDEESHELLTQLGMPFQK.-
16585	556.827	2223.280	2223.273	3.15	0	84	3.8e-09	1	U	K.VLDSAVEELALITGQKPVITK.A

13. tr|Q8Y863 **Mass:** 58159 **Score:** 578 **Matches:** 13(13) **Sequences:** 13(13) **emPAI:** 1.57
Dihydrolipoamide acetyltransferase component of pyruvate dehydrogenase complex OS=Listeria monocytogenes serovar 1/2a (strain ATCC BAA-679 / EGD-e)

Query	Observed	Mr(expt)	Mr(calc)	ppm	Miss	Score	Expect	Rank	Unique	Peptide
298	370.703	739.392	739.390	2.08	0	22	0.013	1	U	K.GIFEFK.L
322	372.241	742.468	742.470	-2.72	0	48	5.3e-05	1	U	K.ALVATLR.D
4594	551.302	1100.589	1100.583	6.11	0	60	3.8e-06	1	U	K.GVNIAEVAGSGK.N
8567	707.876	1413.738	1413.742	-3.37	0	41	0.00026	1	U	R.LLNDPELLLMEV.-
8629	710.836	1419.657	1419.663	-3.92	0	52	1.7e-05	1	U	K.QPVASSDAYPETR.E
9558	504.945	1511.814	1511.813	0.99	1	18	0.039	1	U	K.KDPNGLVIAMPSVR.K
9778	766.362	1530.710	1530.716	-3.77	0	82	9.7e-09	1	U	K.AESTESTPAPAQASGK.G
10623	535.955	1604.842	1604.841	0.68	0	85	1e-08	1	U	K.LPDIGEGIHEGEIVK.W
11271	825.423	1648.831	1648.831	0.18	0	48	7e-05	1	U	K.SVFQISDEINELAGK.A
12710	583.289	1746.845	1746.854	-4.73	1	90	3.5e-09	1	U	K.AAGKQPVASSDAYPETR.E
15480	670.691	2009.051	2009.058	-3.34	0	103	9.5e-11	1	U	K.DGEIVAAPVLALSLSFDHR.V
16295	1092.046	2182.078	2182.068	4.30	0	54	1.3e-05	1	U	R.DFPVLNTTLDDATEELVYK.H
18373	831.398	2491.172	2491.171	0.09	0	79	2.3e-08	1	U	K.ADIDAFLNGEQPAAATTTAQTEEK.A

14. sp|Q8Y6M6 **Mass:** 28414 **Score:** 552 **Matches:** 15(15) **Sequences:** 11(11) **emPAI:** 4.06
30S ribosomal protein S2 OS=Listeria monocytogenes serovar 1/2a (strain ATCC BAA-679 / EGD-e) OX=169963 GN=rpsB PE=3 SV=1

Query	Observed	Mr(expt)	Mr(calc)	ppm	Miss	Score	Expect	Rank	Unique	Peptide
247	365.215	728.416	728.418	-2.30	0	49	4.2e-05	1	U	R.IAVAEAR.K
1027	414.716	827.417	827.418	-1.30	0	26	0.0033	1	U	K.YIFTER.N
5121	572.759	1143.502	1143.502	0.49	0	47	2.2e-05	1	U	K.VDEAFNFMR.E
5515	588.834	1175.653	1175.655	-1.29	0	49	3.9e-05	1	U	R.NGIYIIDLQK.T
6772	424.873	1271.596	1271.597	-0.88	1	31	0.0014	1	U	K.KVDEAFNFMR.E
7367	656.866	1311.718	1311.719	-0.76	0	62	1.1e-06	1	U	K.GLPDALFIVDPR.K 7365 7366
7906	676.822	1351.629	1351.633	-2.59	0	64	8.9e-07	1	U	R.MEADGTFEVLPK.K 7905
8426	467.909	1400.704	1400.701	2.30	1	66	8.1e-07	1	U	K.QAQESVRDEAIR.S
9968	775.906	1549.798	1549.799	-0.26	0	75	1.1e-07	1	U	R.EVASDNGTILFVGTK.K 9969
10645	804.421	1606.828	1606.836	-4.41	0	54	1.4e-05	1	U	R.WLGGTLTNFETIQK.R
4800	560.306	1677.897	1677.894	2.21	1	61	5.8e-06	1	U	R.EVASDNGTILFVGTKK.Q

15. tr|Q8Y498 **Mass:** 30219 **Score:** 523 **Matches:** 11(11) **Sequences:** 8(8) **emPAI:** 2.04
FbaA protein OS=Listeria monocytogenes serovar 1/2a (strain ATCC BAA-679 / EGD-e) OX=169963 GN=fbaA PE=3 SV=1

Query	Observed	Mr(expt)	Mr(calc)	ppm	Miss	Score	Expect	Rank	Unique	Peptide
4332	541.334	1080.653	1080.654	-0.80	0	57	1.9e-06	1	U	K.VIGPGVDAIIK.T
5189	575.786	1149.557	1149.559	-1.31	0	68	6.1e-07	1	U	K.MTEGLVEDLK.I 5190
5970	605.373	1208.732	1208.749	-14.27	1	53	5.3e-06	1	U	R.KVIGPGVDAIIK.T
5987	606.351	1210.687	1210.692	-4.46	0	49	2.4e-05	1	U	K.APVILGVSEGAAK.Y 5988
13491	905.987	1809.959	1809.984	-13.57	1	99	2.9e-10	1	U	K.AAEAEKAPVILGVSEGAAK.Y
13492	604.335	1809.983	1809.984	-0.33	1	(79)	2e-08	1	U	K.AAEAEKAPVILGVSEGAAK.Y
16570	741.395	2221.164	2221.153	4.89	0	32	0.0013	1	U	K.YAVGQFNINNLEWTQAILK.A
18317	828.112	2481.314	2481.323	-3.36	0	60	1.8e-06	1	U	K.EISELTGAPLVLHGGSGIPEHQIK.K
20876	781.883	3123.504	3123.497	2.33	0	82	1.2e-08	1	U	K.EANIDALAAALGSVHGPYHGEPVLGFDEMK.E

16. sp|Q8Y440 **Mass:** 22811 **Score:** 498 **Matches:** 7(7) **Sequences:** 4(4) **emPAI:** 1.08
50S ribosomal protein L3 OS=Listeria monocytogenes serovar 1/2a (strain ATCC BAA-679 / EGD-e) OX=169963 GN=rplC PE=3 SV=1

Query	Observed	Mr(expt)	Mr(calc)	ppm	Miss	Score	Expect	Rank	Unique	Peptide
6563	418.884	1253.630	1253.630	-0.18	0	58	4.9e-06	1	U	R.RPGSMGPVAPNR.V
11944	844.945	1687.875	1687.881	-3.83	0	111	2.1e-11	1	U	R.MGGEQITIQNLEIVK.V
12589	868.441	1734.868	1734.868	0.10	0	107	7.7e-11	1	U	K.VDVFAEGDIIDATGVSK.G
5301	579.300	1734.877	1734.868	5.65	0	(58)	1.3e-05	1	U	K.VDVFAEGDIIDATGVSK.G
20920	1048.560	3142.658	3142.658	-0.22	0	108	1.6e-11	1	U	K.VGMTQVFTENGELIPVTVIEAAQNVVLQK.K

20922	786.674	3142.667	3142.658	2.76	0	(82)	6e-09	1	U	K.VGMTQVFTENGELIPVTVIEAAQNVVLQK.K 20921

---

17. tr|Q8Y865 **Mass:** 41241 **Score:** 458 **Matches:** 11(11) **Sequences:** 8(8) **emPAI:** 1.26
PdhA protein OS=Listeria monocytogenes serovar 1/2a (strain ATCC BAA-679 / EGD-e) OX=169963 GN=pdhA PE=4 SV=1

Query	Observed	Mr(expt)	Mr(calc)	ppm	Miss	Score	Expect	Rank	Unique	Peptide
299	370.709	739.403	739.402	1.36	0	37	0.0003	1	U	K.AFLFSR.G
3109	494.249	986.483	986.503	-20.19	0	21	0.018	1	U	K.ENAVIDQAK.E
3856	523.771	1045.527	1045.540	-13.21	0	73	2.5e-07	1	U	K.QSAAETLAQK.A
4085	532.785	1063.556	1063.555	0.56	0	58	6.9e-06	1	U	K.EQLAIYEAK.E
8013	680.876	1359.738	1359.740	-1.67	0	94	1.5e-09	1	U	K.QFELVQILNEK.G 8012
12480	862.927	1723.840	1723.845	-2.78	0	95	8.9e-10	1	U	R.AVAGEGPTLIETMTYR.Y 12479
12482	575.624	1723.850	1723.845	2.78	0	(24)	0.013	1	U	R.AVAGEGPTLIETMTYR.Y
17404	786.750	2357.228	2357.230	-0.88	0	70	2.2e-07	1	U	K.AVAAGIPGVQVDGMDPLAVYAVTK.F
18094	818.077	2451.210	2451.207	1.11	0	55	7.6e-06	1	U	R.LGFYAPTAGQEASQLASHYALEK.H

---

18. sp|Q8Y6M7 **Mass:** 32675 **Score:** 437 **Matches:** 10(10) **Sequences:** 10(10) **emPAI:** 2.62
Elongation factor Ts OS=Listeria monocytogenes serovar 1/2a (strain ATCC BAA-679 / EGD-e) OX=169963 GN=tsf PE=3 SV=1

Query	Observed	Mr(expt)	Mr(calc)	ppm	Miss	Score	Expect	Rank	Unique	Peptide
2067	451.259	900.503	900.503	-0.07	0	57	7.3e-06	1	U	K.IGENISLR.R
3354	504.255	1006.496	1006.497	-0.68	0	45	0.00013	1	U	R.FEVGEGIEK.K
6338	619.285	1236.556	1236.554	1.13	0	59	3.1e-06	1	U	K.ALVETEGDMEK.A
7982	453.541	1357.602	1357.600	1.83	0	42	7.6e-05	1	U	R.EDVSSEEVAHEK.E
9184	738.375	1474.735	1474.742	-4.18	0	58	1.3e-05	1	U	K.NDNFQQLVDALAK.Q
9595	506.580	1516.720	1516.719	0.29	0	68	5.2e-07	1	U	R.VASEGMTHVISNEK.H
11609	834.436	1666.857	1666.845	7.00	0	51	1.6e-05	1	U	K.YLSEISLEDQPFVK.N
12551	866.489	1730.964	1730.967	-1.79	0	65	5e-07	1	U	R.IGVLTLLEGTTDTTVAK.D
15807	694.380	2080.119	2080.116	1.16	0	82	8.5e-09	1	U	K.EVLTQQALNEGKPANIVEK.M
16173	719.016	2154.025	2154.015	4.69	0	53	1.2e-05	1	U	K.TEMPNGQTVQDYITEAITK.I

---

19. sp|P66548 **Mass:** 24527 **Score:** 436 **Matches:** 13(13) **Sequences:** 9(9) **emPAI:** 3.64
30S ribosomal protein S3 OS=Listeria monocytogenes serovar 1/2a (strain ATCC BAA-679 / EGD-e) OX=169963 GN=rpsC PE=3 SV=1

Query	Observed	Mr(expt)	Mr(calc)	ppm	Miss	Score	Expect	Rank	Unique	Peptide
525	386.719	771.424	771.424	-0.47	0	64	1.4e-06	1	U	R.LGGADIAR.A 527
1103	417.719	833.423	833.424	-1.41	0	67	9e-07	1	U	R.LSDASVSR.V 1102
1845	443.261	884.507	884.508	-1.05	0	24	0.0075	1	U	K.LVAENIAR.Q
2276	459.236	916.457	916.461	-5.05	0	66	9.5e-07	1	U	K.GGSEVEALR.K
2793	480.259	958.504	958.508	-5.01	0	46	9.7e-05	1	U	K.NLNELTQK.R 2792
3836	523.284	1044.554	1044.556	-2.61	1	38	0.00066	1	U	K.GGSEVEALRK.N
4092	532.825	1063.635	1063.639	-3.27	0	31	0.00082	1	U	R.VHINIVEIK.R
8344	697.326	1392.638	1392.631	4.95	0	99	2e-10	1	U	K.DYADFLHEDLR.I
12290	570.624	1708.851	1708.853	-1.25	0	52	2.2e-05	1	U	R.AEHYSEGTVPLHTLR.A
12291	428.221	1708.856	1708.853	1.51	0	(24)	0.011	1	U	R.AEHYSEGTVPLHTLR.A

---

20. tr|Q8Y7B0 **Mass:** 52497 **Score:** 421 **Matches:** 9(9) **Sequences:** 7(7) **emPAI:** 0.75
6-phosphogluconate dehydrogenase, decarboxylating OS=Listeria monocytogenes serovar 1/2a (strain ATCC BAA-679 / EGD-e) OX=169963 GN=lmo1376 PE=3 S\

Query	Observed	Mr(expt)	Mr(calc)	ppm	Miss	Score	Expect	Rank	Unique	Peptide
3604	515.282	1028.549	1028.561	-11.79	0	60	3.5e-06	1	U	R.NLALNIESR.G
4122	534.253	1066.492	1066.493	-1.43	0	58	3.4e-06	1	U	K.ITDAYNQDK.N
4843	561.813	1121.611	1121.612	-0.77	0	49	4.5e-05	1	U	K.NLLLDPYFK.D
8713	714.896	1427.778	1427.773	3.10	0	93	1.9e-09	1	U	R.SEVLSANLIQAQR.D 8712
9736	764.392	1526.769	1526.773	-2.24	0	69	3.6e-07	1	U	R.VYASTVLTGPSNYR.F
14564	633.354	1897.041	1897.041	0.13	1	42	9.8e-05	1	U	K.VKDEETGKPIVDVILDK.A
14565	475.271	1897.055	1897.041	7.49	1	(35)	0.00037	1	U	K.VKDEETGKPIVDVILDK.A
15950	703.035	2106.084	2106.088	-2.03	0	77	6.1e-08	1	U	K.LVPTYSLEEFVESLEVPR.R

---

21. sp|Q8Y822 **Mass:** 58363 **Score:** 421 **Matches:** 11(11) **Sequences:** 11(11) **emPAI:** 1.21
GMP synthase [glutamine-hydrolyzing] OS=Listeria monocytogenes serovar 1/2a (strain ATCC BAA-679 / EGD-e) OX=169963 GN=guaA PE=3 SV=1

Query	Observed	Mr(expt)	Mr(calc)	ppm	Miss	Score	Expect	Rank	Unique	Peptide
3472	509.279	1016.544	1016.539	4.94	0	43	0.00021	1	U	R.VLGEITEEK.L
3798	522.271	1042.526	1042.529	-2.87	0	50	2.2e-05	1	U	K.LAGVSDPEQK.R
5326	580.306	1158.598	1158.603	-4.71	0	35	0.0023	1	U	R.HSVYGNELLK.N
5669	594.354	1186.694	1186.696	-2.06	0	29	0.002	1	U	K.LIEPLNTLFK.D
5751	398.880	1193.618	1193.615	2.50	0	45	0.00014	1	U	R.IVNEVDHVNR.V
7424	659.313	1316.611	1316.603	5.87	0	28	0.0084	1	U	K.SCPIAGIADEER.S
4384	543.316	1626.926	1626.919	3.91	1	94	2.2e-09	1	U	R.VLGEITEEKLEIVR.D
11924	562.985	1685.932	1685.935	-1.98	1	100	2.4e-10	1	U	K.LIEPLNTLFKDEVR.A
14873	643.341	1927.000	1926.999	0.75	0	50	3.1e-05	1	U	K.AIGDQLTCIFVDHGLLR.K
15147	655.058	1962.153	1962.151	0.78	0	83	4.6e-09	1	U	K.VLLALSGGVDSSVVGVLIHK.A
17912	809.724	2426.150	2426.146	1.62	1	37	0.00038	1	U	R.KGEADQVMETLQGEFNMNIIK.V

---

22. tr|Q8Y4I1 **Mass:** 36435 **Score:** 413 **Matches:** 10(10) **Sequences:** 10(10) **emPAI:** 2.17
Glyceraldehyde-3-phosphate dehydrogenase OS=Listeria monocytogenes serovar 1/2a (strain ATCC BAA-679 / EGD-e) OX=169963 GN=gap PE=3 SV=1

Query	Observed	Mr(expt)	Mr(calc)	ppm	Miss	Score	Expect	Rank	Unique	Peptide
937	410.230	818.446	818.440	7.91	0	22	0.034	1	U	K.VGINGFGR.I
3753	520.821	1039.627	1039.628	-0.79	0	43	5.8e-05	1	U	K.AIGEVLPTLK.G
5811	600.346	1198.678	1198.692	-11.53	0	59	3.7e-06	1	U	K.VLTVGDQQLVK.T
5890	602.813	1203.611	1203.617	-4.97	0	57	6.6e-06	1	U	K.VVISAPATGDMK.T
8852	721.377	1440.739	1440.757	-12.61	0	65	9.1e-07	1	U	R.AAAENIIPNTTGAAK.A
9722	509.238	1524.692	1524.689	2.19	0	42	7.4e-05	1	U	R.FDGEVEVHDGFFK.V
10321	789.367	1576.719	1576.708	7.05	0	75	5.3e-08	1	U	K.GMTFGSLFDETQTK.V
13049	590.007	1767.000	1767.003	-1.48	0	60	1.4e-06	1	U	R.VPVPTGSLTELVTVLDK.K

14546	632.707	1895.100	1895.098	1.15	1	77	1.8e-08	1	U	R.VPVPTGSLTELVTVLDKK.V
15631	681.032	2040.075	2040.074	0.72	0	53	1.3e-05	1	U	R.IQNVEGIEVVAINDLTDAK.M

---

23. tr|Q8YAN9 **Mass:** 33944 **Score:** 408 **Matches:** 10(9) **Sequences:** 8(7) **emPAI:** 1.38
Lmo0078 protein OS=Listeria monocytogenes serovar 1/2a (strain ATCC BAA-679 / EGD-e) OX=169963 GN=lmo0078 PE=3 SV=1

Query	Observed	Mr(expt)	Mr(calc)	ppm	Miss	Score	Expect	Rank	Unique	Peptide
582	390.207	778.399	778.386	16.9	0	18	0.063	1	U	K.IGEDFAK.L
2051	450.284	898.553	898.560	-8.31	0	43	6.6e-05	1	U	K.TLGIIGLGR.I
3892	525.288	1048.562	1048.567	-4.37	0	44	0.00013	1	U	K.SSAFLINAAR.G 3893
4892	563.326	1124.637	1124.644	-6.63	0	63	5.3e-07	1	U	R.GPVVEEAALIK.A
6732	634.882	1267.750	1267.750	-0.00	0	72	6.6e-08	1	U	K.IAIANVEAVLAGK.A 6731
12421	574.287	1719.839	1719.840	-0.56	0	91	2.5e-09	1	U	R.AAAFGMNIIYSGHQPK.E
15045	650.356	1948.045	1948.038	3.87	0	55	7.1e-06	1	U	K.LDNVVLTPHIGNATVETR.A
15792	693.032	2076.075	2076.078	-1.53	0	93	1.1e-09	1	U	K.ALETGVIAGAALDVFEFEPK.I

---

24. sp|Q8Y446 **Mass:** 17445 **Score:** 394 **Matches:** 11(11) **Sequences:** 9(9) **emPAI:** 7.54
30S ribosomal protein S5 OS=Listeria monocytogenes serovar 1/2a (strain ATCC BAA-679 / EGD-e) OX=169963 GN=rpsE PE=3 SV=1

Query	Observed	Mr(expt)	Mr(calc)	ppm	Miss	Score	Expect	Rank	Unique	Peptide
19	351.218	700.422	700.423	-1.58	0	43	0.00031	1	U	R.VVTINR.V
173	359.209	716.404	716.407	-4.02	0	54	2.3e-05	1	U	R.ATIDGIK.Q
432	380.710	759.406	759.401	5.69	0	39	0.0013	1	U	K.TVEELLG.-
3237	499.766	997.517	997.519	-2.68	0	46	4.9e-05	1	U	K.AQEVPDAIR.K
3877	524.806	1047.597	1047.596	0.29	0	51	2.8e-05	1	U	R.FTALVVVGDK.N
4904	376.214	1125.620	1125.614	5.33	1	65	7.1e-07	1	U	K.AQEVPDAIRK.A 4903
7219	652.833	1303.652	1303.655	-2.32	0	79	4.2e-08	1	U	K.SLGSNTPINMVR.A
7991	679.879	1357.744	1357.745	-0.68	0	86	8e-09	1	U	R.AVLELAGVADVSSK.S 7990
11409	828.422	1654.829	1654.816	7.77	1	43	0.00013	1	U	M.PEQIDGNKLDLEER.V

---

25. sp|Q8Y4B3 **Mass:** 23044 **Score:** 393 **Matches:** 9(9) **Sequences:** 8(8) **emPAI:** 3.25
Uracil phosphoribosyltransferase OS=Listeria monocytogenes serovar 1/2a (strain ATCC BAA-679 / EGD-e) OX=169963 GN=upp PE=3 SV=1

Query	Observed	Mr(expt)	Mr(calc)	ppm	Miss	Score	Expect	Rank	Unique	Peptide
1763	440.803	879.591	879.591	-0.01	0	46	2.5e-05	1	U	K.LGIVPILR.A
4782	559.797	1117.579	1117.580	-1.24	0	100	3.6e-10	1	U	R.AGLGMQDGILK.L
7713	669.333	1336.652	1336.652	-0.05	0	40	0.00035	1	U	K.FMCLVAAPEGVK.A
10479	796.391	1590.768	1590.757	7.01	0	42	0.00018	1	U	R.DHDTLEPVEYFVK.L
14176	934.969	1867.924	1867.924	0.34	0	54	1.6e-05	1	U	R.ELVDEVATLMAYEITR.D 14178
15824	695.017	2082.030	2082.027	1.29	0	66	6e-07	1	U	K.ALQDAHPDVEIYVAGLDEK.L
16275	1089.531	2177.047	2177.041	2.82	0	61	2e-06	1	U	R.DMELEDIQVETPLQTTAK.T
18202	823.093	2466.258	2466.257	0.18	0	92	1.2e-09	1	U	R.LFIVVDPMLATGGSAIMAIDCLK.K

---

26. sp|Q8Y5G2 **Mass:** 31814 **Score:** 385 **Matches:** 11(10) **Sequences:** 10(9) **emPAI:** 2.27
Pyridoxal 5'-phosphate synthase subunit PdxS OS=Listeria monocytogenes serovar 1/2a (strain ATCC BAA-679 / EGD-e) OX=169963 GN=pdxS PE=3 SV=1

Query	Observed	Mr(expt)	Mr(calc)	ppm	Miss	Score	Expect	Rank	Unique	Peptide
537	387.212	772.409	772.408	1.75	0	31	0.0021	1	U	R.DIGEALR.R
1547	432.229	862.444	862.447	-3.37	0	45	0.00017	1	U	R.IVEEVMK.A
2452	466.266	930.518	930.521	-2.64	0	21	0.038	1	U	K.AVSIPVMAK.A
2473	467.248	932.481	932.475	6.60	0	19	0.065	1	U	R.IGEGAAMLR.T
7140	649.832	1297.650	1297.663	-9.51	0	18	0.052	1	U	K.GEPGTGNIVEAVR.H
8913	723.865	1445.715	1445.718	-2.44	0	83	1.5e-08	1	U	K.GGVIMDVVNAEQAK.I 8914
9740	509.942	1526.803	1526.805	-1.52	1	74	1.1e-07	1	U	R.TKGEPGTGNIVEAVR.H
9922	773.400	1544.785	1544.787	-1.16	0	97	7e-10	1	U	K.IAEEAGAVAVMALER.V
10792	812.892	1623.769	1623.748	12.6	0	73	1.7e-07	1	U	R.QIAGMTDDELMVAAK.N
20760	1022.166	3063.475	3063.463	3.89	0	41	9.1e-05	1	U	R.VLEAMGVDYIDESEVLTPADDEFHLLK.S

---

27. tr|Q8Y862 **Mass:** 49571 **Score:** 383 **Matches:** 9(9) **Sequences:** 9(9) **emPAI:** 1.15
Dihydrolipoyl dehydrogenase OS=Listeria monocytogenes serovar 1/2a (strain ATCC BAA-679 / EGD-e) OX=169963 GN=PdhD PE=3 SV=1

Query	Observed	Mr(expt)	Mr(calc)	ppm	Miss	Score	Expect	Rank	Unique	Peptide
25	351.722	701.430	701.432	-3.88	0	33	0.0019	1	U	K.VTIIEK.E
2943	486.771	971.528	971.529	-1.08	0	57	8e-06	1	U	K.IAAEAIAGEK.S
6803	637.838	1273.662	1273.667	-3.50	0	46	9.9e-05	1	U	R.ALSLDAPEGFVR.L
9298	743.428	1484.841	1484.845	-2.62	0	131	1.8e-13	1	U	R.VLSSTGALALTEVPK.K
10812	542.956	1625.848	1625.837	6.35	0	57	6.5e-06	1	U	R.RPNTDEIGLEQAGVK.V
10836	815.449	1628.883	1628.889	-3.19	0	33	0.0016	1	U	R.DTIVIGAGPGGYVAAIR.A
15474	670.649	2008.925	2008.920	2.69	0	37	0.00037	1	U	K.VEMLEGEAFFVDDHSLR.V
16138	1074.572	2147.129	2147.174	-21.03	0	57	7e-06	1	U	R.SNVSNIFAIGDIVPGVPLAHK.A
16749	751.360	2251.059	2251.058	0.60	1	68	2.7e-07	1	U	K.NKVEMLEGEAFFVDDHSLR.V

---

28. sp|Q8YAA4 **Mass:** 24517 **Score:** 332 **Matches:** 10(9) **Sequences:** 8(7) **emPAI:** 2.30
50S ribosomal protein L1 OS=Listeria monocytogenes serovar 1/2a (strain ATCC BAA-679 / EGD-e) OX=169963 GN=rplA PE=3 SV=1

Query	Observed	Mr(expt)	Mr(calc)	ppm	Miss	Score	Expect	Rank	Unique	Peptide
286	369.196	736.376	736.376	1.28	0	37	0.0008	1	U	K.VSFDAAK.L 285
570	389.219	776.423	776.418	5.99	0	20	0.09	1	U	K.LVENFR.T
2268	458.759	915.504	915.503	1.27	0	43	0.00025	1	U	R.TVNDVLQK.A
3424	506.793	1011.571	1011.571	-0.43	0	33	0.0005	1	U	R.GAVVLPNGTGK.T
4137	534.761	1067.508	1067.517	-8.56	0	72	1.6e-07	1	U	K.TGTVTMDVTK.A
7500	661.844	1321.673	1321.677	-2.79	0	57	7.3e-06	1	U	K.VYTAEEAVELAK.K
7692	667.867	1333.719	1333.724	-3.98	0	53	1.8e-05	1	U	K.NLSVTTTFGPGIK.V
14580	634.285	1899.834	1899.837	-1.68	0	(54)	3.6e-06	1	U	K.EAEAAGADYVGESEFVEK.I
14581	950.925	1899.836	1899.837	-0.96	0	88	1.8e-09	1	U	K.EAEAAGADYVGESEFVEK.I

---

29. sp|Q8Y421 **Mass:** 76973 **Score:** 323 **Matches:** 11(11) **Sequences:** 11(11) **emPAI:** 0.83

Elongation factor G OS=Listeria monocytogenes serovar 1/2a (strain ATCC BAA-679 / EGD-e) OX=169963 GN=fusA PE=3 SV=1

Query	Observed	Mr(expt)	Mr(calc)	ppm	Miss	Score	Expect	Rank	Unique	Peptide
1636	435.247	868.479	868.479	-2.20	0	25	0.0041	1	U	R.ILFYTGR.I
2083	451.763	901.512	901.512	-0.33	0	40	0.00042	1	U	K.SIAEEIIK.A
3172	496.761	991.508	991.509	-0.86	0	56	1.7e-05	1	U	R.QATTYGVPR.V
3932	527.257	1052.500	1052.496	3.36	0	24	0.01	1	U	K.VMTDPYVGR.L
4259	539.264	1076.514	1076.514	-0.07	0	34	0.001	1	U	K.LAEEDPTFR.A
8757	717.356	1432.698	1432.695	2.21	0	50	2.7e-05	1	U	R.VEANVGDPQVSYR.E
10471	795.911	1589.808	1589.820	-7.74	0	45	0.00012	1	U	K.GFEFENAIVGGVVPR.E
11751	559.957	1676.850	1676.852	-1.37	0	89	4.6e-09	1	U	K.IGADFLYSVGTLHER.L
13415	602.294	1803.861	1803.864	-1.75	0	49	1.9e-05	1	U	R.VYSGTLNSGSYVQNSTK.G
13629	608.648	1822.923	1822.919	2.40	0	37	0.00053	1	U	R.AFVPLANMFGYATHLR.S
20497	967.192	2898.554	2898.538	5.71	0	41	8.8e-05	1	U	R.EYIPAVQAGLEGALDNGVLAGYPLIDIK.A

30. sp|Q8Y915 **Mass:** 66125 **Score:** 316 **Matches:** 12(11) **Sequences:** 10(9) **emPAI:** 0.78

Glutamine--fructose-6-phosphate aminotransferase [isomerizing] OS=Listeria monocytogenes serovar 1/2a (strain ATCC BAA-679 / EGD-e) OX=169963 GN=gl

Query	Observed	Mr(expt)	Mr(calc)	ppm	Miss	Score	Expect	Rank	Unique	Peptide
977	412.724	823.434	823.434	0.37	0	36	0.00079	1	U	R.NAFFLGR.N
2921	486.263	970.511	970.520	-8.44	0	20	0.081	1	U	R.GNVNEVLAR.G
2926	486.290	970.566	970.570	-3.61	0	34	0.0017	1	U	K.GILLEGLEK.L
5295	579.288	1156.562	1156.572	-9.14	0	33	0.0011	1	U	K.EIDEQPAVTR.K 5296
6643	631.336	1260.658	1260.656	1.65	0	72	2.5e-07	1	U	K.TIIDEILSSDR.I
9314	744.402	1486.789	1486.799	-6.54	0	53	3.8e-05	1	U	R.TLTLTNVPGSTLDR.E 9315
11937	563.292	1686.854	1686.858	-1.98	0	50	3.2e-05	1	U	K.EVIEHIAGEYLATSR.N
12287	855.414	1708.814	1708.816	-1.15	0	75	1.1e-07	1	U	K.DGFTLETLEGEEITR.A
13430	903.456	1804.897	1804.884	7.28	0	63	2.8e-06	1	U	K.IIQAYQDEAGEINVDK.T
16388	733.718	2198.133	2198.133	-0.16	0	33	0.0017	1	U	R.IADLASLVPSDAFGTTGIGHTR.W

31. sp|Q8Y6W0 **Mass:** 34399 **Score:** 311 **Matches:** 7(7) **Sequences:** 7(7) **emPAI:** 1.35

ATP-dependent 6-phosphofructokinase OS=Listeria monocytogenes serovar 1/2a (strain ATCC BAA-679 / EGD-e) OX=169963 GN=pfkA PE=3 SV=1

Query	Observed	Mr(expt)	Mr(calc)	ppm	Miss	Score	Expect	Rank	Unique	Peptide
2915	486.250	970.486	970.487	-1.77	0	42	8.1e-05	1	U	R.GGTFLYSAR.Y
4328	541.288	1080.562	1080.564	-1.72	0	23	0.037	1	U	R.TFIIEVMGR.D
6781	636.856	1271.697	1271.697	-0.02	0	42	0.00027	1	U	R.IVENDISEILK.E
8518	706.333	1410.650	1410.667	-11.50	0	69	5.5e-07	1	U	R.YPEFATEEGQLK.G
8796	479.613	1435.817	1435.815	1.85	1	100	1.8e-10	1	U	R.KLELGSVGDLLHR.G
12546	866.434	1730.854	1730.862	-4.69	0	88	4.7e-09	1	U	R.IAILTSGGDAPGMNAATR.A
6736	635.322	1902.944	1902.951	-3.50	0	44	0.00027	1	U	K.HSIIVVAEGVMSGNEFAK.Q

32. sp|O31149 **Mass:** 63286 **Score:** 307 **Matches:** 7(7) **Sequences:** 7(7) **emPAI:** 0.60

Phosphoenolpyruvate-protein phosphotransferase OS=Listeria monocytogenes serovar 1/2a (strain ATCC BAA-679 / EGD-e) OX=169963 GN=ptsI PE=3 SV=2

Query	Observed	Mr(expt)	Mr(calc)	ppm	Miss	Score	Expect	Rank	Unique	Peptide
919	409.717	817.419	817.418	1.41	0	54	2e-05	1	U	R.TLDIGGDK.T
3703	519.259	1036.504	1036.519	-14.33	0	47	5.5e-05	1	U	R.FESALEVSR.T
4393	543.807	1085.600	1085.608	-7.72	0	52	1.9e-05	1	U	K.GIAASDGIAIAK.A
7133	649.813	1297.612	1297.612	-0.09	0	64	6.5e-07	1	U	R.LCFANEELFR.T
7663	666.847	1331.679	1331.682	-2.10	0	74	2.1e-07	1	U	K.STAEEVVELVEK.Y
9875	770.391	1538.767	1538.787	-12.61	0	49	7.7e-05	1	U	K.AYLLVEPDLSYEK.T
12338	856.947	1711.880	1711.893	-7.53	0	71	3e-07	1	U	R.VSYLYQPYNPSILR.L

33. sp|Q8Y6D2 **Mass:** 52434 **Score:** 306 **Matches:** 6(6) **Sequences:** 6(6) **emPAI:** 0.62

Glutamyl-tRNA(Gln) amidotransferase subunit A OS=Listeria monocytogenes serovar 1/2a (strain ATCC BAA-679 / EGD-e) OX=169963 GN=gatA PE=3 SV=1

Query	Observed	Mr(expt)	Mr(calc)	ppm	Miss	Score	Expect	Rank	Unique	Peptide
1489	429.254	856.494	856.502	-9.26	0	36	0.0011	1	U	K.QAVLDALK.T
3014	489.784	977.554	977.555	-0.30	0	49	3.3e-05	1	U	K.VGSFITLNK.E
4338	541.771	1081.528	1081.529	-0.90	0	85	9.8e-09	1	U	R.FSDSLTGDIK.G
9095	733.871	1465.727	1465.730	-2.35	0	104	1.2e-10	1	U	R.SPNATTLEELYTK.T
13630	912.472	1822.930	1822.935	-3.11	0	32	0.0017	1	U	K.ILENFDPIYDATVVSK.L
14275	939.518	1877.022	1877.030	-4.13	0	84	6.5e-09	1	U	R.FGLIAFASSLDQIGPITK.N

34. sp|O77727 **Mass:** 48740 **Score:** 305 **Matches:** 10(9) **Sequences:** 5(4) **emPAI:** 0.41

Keratin, type I cytoskeletal 15 (Contact-Cont) OS=Ovis aries GN=KRT15 PE=2 SV=1

Query	Observed	Mr(expt)	Mr(calc)	ppm	Miss	Score	Expect	Rank	Unique	Peptide
822	404.203	806.392	806.392	-0.26	0	50	4.2e-05	1		R.LAADDFR.L 820 821 823
1090	417.218	832.421	832.429	-9.66	0	18	0.071	1	U	K.SEITDLR.R
3636	516.303	1030.591	1030.591	0.14	0	72	2.4e-07	1		R.VLDELTLTK.T 3635 3637
5830	601.311	1200.607	1200.610	-2.10	0	101	2.7e-10	1		R.QSVEADINGLR.R
7980	453.245	1356.712	1356.711	0.53	1	52	2.4e-05	1		R.QSVEADINGLRR.V

35. tr|Q8Y4M2 **Mass:** 29193 **Score:** 289 **Matches:** 6(6) **Sequences:** 6(6) **emPAI:** 1.37

Lmo2415 protein OS=Listeria monocytogenes serovar 1/2a (strain ATCC BAA-679 / EGD-e) OX=169963 GN=lmo2415 PE=4 SV=1

Query	Observed	Mr(expt)	Mr(calc)	ppm	Miss	Score	Expect	Rank	Unique	Peptide
743	400.712	799.409	799.408	1.73	0	52	8.2e-06	1	U	K.EGGPELAK.R
5813	600.779	1199.543	1199.546	-2.41	0	64	6.9e-07	1	U	R.YLNEGFSGGEK.K
9009	728.903	1455.792	1455.801	-6.27	0	60	3.3e-06	1	U	R.NEILQLLMIEPK.L
12578	578.957	1733.849	1733.841	4.90	1	18	0.053	1	U	R.REEGDEIPVMQFIR.K
13515	907.494	1812.974	1812.972	1.29	0	91	1.4e-09	1	U	K.LAILDEIDSGLDIDALK.V
18505	844.091	2529.251	2529.257	-2.60	0	83	1.2e-08	1	U	R.AGLFLAMQYPSEISGVTNAEFIR.A

36. sp|P66042 **Mass:** 17739 **Score:** 282 **Matches:** 8(8) **Sequences:** 7(7) **emPAI:** 4.14
50S ribosomal protein L10 OS=Listeria monocytogenes serovar 1/2a (strain ATCC BAA-679 / EGD-e) OX=169963 GN=rplJ PE=3 SV=1

Query	Observed	Mr(expt)	Mr(calc)	ppm	Miss	Score	Expect	Rank	Unique	Peptide
944	410.730	819.445	819.449	-4.77	0	23	0.027	1	U	K.ILNDFAK.D
1035	414.750	827.486	827.486	-1.06	0	54	1e-05	1	U	K.ALATLPSR.E
1884	444.754	887.493	887.496	-3.25	0	54	1.7e-05	1	U	K.VASLEEIK.A
4983	378.209	1131.606	1131.614	-6.35	1	30	0.0041	1	U	K.QSAVEEIKTK.L
5655	593.824	1185.633	1185.635	-2.26	0	58	5.7e-06	1	U	R.GLNVGEITDLR.K
7405	438.919	1313.735	1313.730	3.82	1	64	1.3e-06	1	U	R.GLNVGEITDLRK.Q
8225	691.370	1380.726	1380.725	0.99	0	85	1.6e-08	1	U	K.LSASASTVIVDYR.G 8224

37. tr|Q8Y6P3 **Mass:** 98473 **Score:** 267 **Matches:** 8(8) **Sequences:** 8(8) **emPAI:** 0.41
Aconitate hydratase OS=Listeria monocytogenes serovar 1/2a (strain ATCC BAA-679 / EGD-e) OX=169963 GN=citB PE=3 SV=1

Query	Observed	Mr(expt)	Mr(calc)	ppm	Miss	Score	Expect	Rank	Unique	Peptide
161	358.224	714.434	714.439	-6.66	0	25	0.016	1	U	K.VTQVLR.E
2412	464.794	927.573	927.575	-2.00	0	41	0.00017	1	U	R.VLLESVLR.Q
3304	502.290	1002.565	1002.575	-9.64	0	59	5.1e-06	1	U	K.AGLLPYLEK.L
8221	691.334	1380.653	1380.652	0.50	0	35	0.0009	1	U	R.EDGSSITFDALAR.F
9480	752.399	1502.784	1502.798	-9.46	0	53	1.7e-05	1	U	K.ALVEETVTPELFR.E
11685	836.961	1671.907	1671.920	-7.73	0	45	7.9e-05	1	U	K.LLGALPNGATATDFALK.V
13086	591.631	1771.870	1771.874	-2.38	0	82	1.6e-08	1	U	K.FGDSVTTDHISPAGAIGK.D
15491	671.718	2012.132	2012.131	0.62	0	45	4.1e-05	1	U	R.VILQDFTGVPAVVDLASLR.K

38. sp|P66330 **Mass:** 11674 **Score:** 267 **Matches:** 8(8) **Sequences:** 5(5) **emPAI:** 4.83
30S ribosomal protein S10 OS=Listeria monocytogenes serovar 1/2a (strain ATCC BAA-679 / EGD-e) OX=169963 GN=rpsJ PE=3 SV=1

Query	Observed	Mr(expt)	Mr(calc)	ppm	Miss	Score	Expect	Rank	Unique	Peptide
1424	426.250	850.485	850.491	-6.81	0	43	7.9e-05	1	U	K.SIYTVLR.A 1423
2091	452.242	902.469	902.471	-2.28	0	61	3.6e-06	1	U	R.ILDQSAEK.I 2090
7142	649.863	1297.712	1297.713	-1.06	0	59	2.3e-06	1	U	R.LDLPSGVDIEIK.L
8534	706.403	1410.792	1410.797	-3.87	1	36	0.00049	1	U	R.LDLPSGVDIEIKL.-
8829	720.390	1438.766	1438.767	-0.85	0	84	7.4e-09	1	U	R.SGASVSGPIPLPTEK.S 8828

39. tr|Q8Y6T3 **Mass:** 36960 **Score:** 267 **Matches:** 7(7) **Sequences:** 7(7) **emPAI:** 1.22
Catabolite control protein A OS=Listeria monocytogenes serovar 1/2a (strain ATCC BAA-679 / EGD-e) OX=169963 GN=ccpA PE=4 SV=1

Query	Observed	Mr(expt)	Mr(calc)	ppm	Miss	Score	Expect	Rank	Unique	Peptide
5595	590.785	1179.556	1179.555	0.32	0	42	0.00019	1	U	R.EANVSMATVSR.V
7115	648.814	1295.614	1295.618	-3.39	0	30	0.0022	1	U	K.QVDGIIYMGER.I
8346	697.337	1392.659	1392.652	4.60	0	69	7.6e-07	1	U	R.ISEQLQEEFDR.S
8362	465.269	1392.786	1392.784	1.90	0	71	1e-07	1	U	R.VVNGNPNVKPVTR.K
10846	815.931	1629.848	1629.847	0.08	0	77	7e-08	1	U	K.QIAFVSGSLNEPVNR.E
17003	760.416	2278.226	2278.221	2.35	0	26	0.0034	1	U	R.TTTVGVIIPDISNVFYAELAR.G
18739	864.153	2589.437	2589.438	-0.33	0	44	3.8e-05	1	U	K.KPNAVVVADDELAIGILNAALDAGIK.V

40. tr|Q926Y9 **Mass:** 52637 **Score:** 251 **Matches:** 11(11) **Sequences:** 10(10) **emPAI:** 1.23
Inosine-5'-monophosphate dehydrogenase OS=Listeria monocytogenes serovar 1/2a (strain ATCC BAA-679 / EGD-e) OX=169963 GN=guaB PE=1 SV=1

Query	Observed	Mr(expt)	Mr(calc)	ppm	Miss	Score	Expect	Rank	Unique	Peptide
592	391.203	780.392	780.402	-13.01	0	22	0.018	1	U	K.YSGDIVK.A
949	411.213	820.411	820.408	3.43	0	26	0.025	1	U	R.EEAAFVR.M
1846	443.275	884.536	884.544	-9.17	0	45	0.00013	1	U	K.LVGILTNR.D
1868	444.265	886.515	886.512	3.04	0	33	0.0017	1	U	K.TIIADGGIK.Y
5418	584.350	1166.685	1166.691	-5.37	0	68	2.8e-07	1	U	K.LPLVDEAGILK.G
5441	585.332	1168.649	1168.645	3.18	0	34	0.00064	1	U	R.ISGVPIVNNEK.E
7793	671.875	1341.735	1341.750	-11.65	0	40	0.00035	1	U	K.ENLVTAPVGTTLK.Q
9587	758.916	1515.817	1515.818	-0.94	0	32	0.0019	1	U	K.EGLTFDDVLLVPAK.S
20677	755.125	3016.472	3016.464	2.49	0	18	0.025	1	U	R.SESGVIIDPFYLTPDHQVFAAEHLMGK.Y
15688	1028.154	3081.441	3081.438	0.97	0	55	4.5e-06	1	U	K.ALAAGGNAVMLGSMLAGTDESPGETEIFQGR.Q
9891	771.375	3081.469	3081.438	10.1	0	(37)	0.0011	1	U	K.ALAAGGNAVMLGSMLAGTDESPGETEIFQGR.Q

41. sp|P64074 **Mass:** 46444 **Score:** 249 **Matches:** 5(4) **Sequences:** 5(4) **emPAI:** 0.44
Enolase OS=Listeria monocytogenes serovar 1/2a (strain ATCC BAA-679 / EGD-e) OX=169963 GN=eno PE=1 SV=1

Query	Observed	Mr(expt)	Mr(calc)	ppm	Miss	Score	Expect	Rank	Unique	Peptide
2413	464.795	927.576	927.575	0.45	0	30	0.002	1	U	K.GIANSILIK.V
7169	650.834	1299.653	1299.667	-10.48	0	80	2.6e-08	1	U	K.AVENVNDIIADK.I
9806	767.397	1532.779	1532.809	-19.61	0	21	0.074	1	U	K.IIGFDVTDQIGIDK.A
11222	823.409	1644.804	1644.811	-4.12	0	93	1.2e-09	1	U	K.GLNTGVGDEGGFAPNLK.S
11274	825.441	1648.868	1648.867	0.54	0	90	4.8e-09	1	U	R.VQLVGDDLFVTNTTK.L

42. sp|Q8Y4R7 **Mass:** 49866 **Score:** 248 **Matches:** 7(7) **Sequences:** 7(7) **emPAI:** 0.81
Glucose-6-phosphate isomerase OS=Listeria monocytogenes serovar 1/2a (strain ATCC BAA-679 / EGD-e) OX=169963 GN=pgi PE=3 SV=1

Query	Observed	Mr(expt)	Mr(calc)	ppm	Miss	Score	Expect	Rank	Unique	Peptide
2838	482.277	962.540	962.544	-3.37	0	30	0.0027	1	U	R.NLFETVIK.V
5507	588.812	1175.609	1175.607	1.71	0	31	0.0034	1	U	R.ELDYLEPAVK.A
6509	625.810	1249.605	1249.630	-20.42	0	18	0.057	1	U	K.SGTTTEPAIAFR.V
6562	627.814	1253.613	1253.615	-2.05	0	39	0.00045	1	U	K.NNIAYQYAAAR.N
16236	1085.494	2168.973	2168.986	-6.06	0	116	6.7e-12	1	U	K.TLSDNEGYETFVVPDDVGGR.F
17757	803.743	2408.207	2408.201	2.48	0	47	4e-05	1	U	K.AVAISGYLNGVNPFDQPGVEAYK.A
18927	876.085	2625.232	2625.246	-5.23	0	68	3.1e-07	1	U	K.GIYPSSANFSTDLHSIGQYIQDGR.R

43. sp|P61055 **Mass:** 22590 **Score:** 244 **Matches:** 9(9) **Sequences:** 7(7) **emPAI:** 2.64
50S ribosomal protein L4 OS=Listeria monocytogenes serovar 1/2a (strain ATCC BAA-679 / EGD-e) OX=169963 GN=rplD PE=3 SV=1

Query	Observed	Mr(expt)	Mr(calc)	ppm	Miss	Score	Expect	Rank	Unique	Peptide
991	413.226	824.438	824.443	-6.52	0	47	0.00021	1	U	K.EFAAFLK.N
3803	522.282	1042.549	1042.556	-7.12	0	25	0.027	1	U	R.GGGVVFGPTPR.S
5303	579.321	1156.628	1156.634	-5.01	1	46	8.6e-05	1	U	K.AAVEKVEEVLA.-
8432	701.403	1400.791	1400.792	-0.04	0	57	3.4e-06	1	U	K.LVVLEGLTFDAPK.T 8431
12838	878.960	1755.906	1755.937	-17.49	0	73	1.5e-07	1	U	K.ALIVVAGESENVELSAR.N 12839
15808	694.396	2080.166	2080.178	-5.98	0	49	1.1e-05	1	U	R.NLQGITVIPAESISVLEVAK.H
11182	821.401	2461.182	2461.161	8.72	0	48	9.2e-05	1	U	K.QDGTNAGEITLNDTVFGIEPNEK.V

44. tr|Q8Y8D5 **Mass:** 28391 **Score:** 238 **Matches:** 6(5) **Sequences:** 5(4) **emPAI:** 0.80
Enoyl-[acyl-carrier-protein] reductase [NADH] OS=Listeria monocytogenes serovar 1/2a (strain ATCC BAA-679 / EGD-e) OX=169963 GN=lmo0970 PE=3 SV=1

Query	Observed	Mr(expt)	Mr(calc)	ppm	Miss	Score	Expect	Rank	Unique	Peptide
1866	444.261	886.507	886.502	4.75	0	25	0.026	1	U	R.SIAWAIAR.S
4562	550.280	1098.546	1098.535	10.4	0	56	7.6e-06	1	U	K.LVFTYADDR.A
7098	647.843	1293.672	1293.675	-2.53	0	17	0.074	1	U	R.YLAMDLGAIGVR.V
10369	791.394	1580.774	1580.768	3.43	0	105	1.1e-10	1	U	R.GVSGFSDSISLVEER.A
16647	745.693	2234.059	2234.049	4.22	0	68	3.7e-07	1	U	R.ATQAEEVGDTAYYLFSNLSR.G
16649	1118.047	2234.079	2234.049	13.4	0	(49)	2.8e-05	1	U	R.ATQAEEVGDTAYYLFSNLSR.G

45. sp|Q8Y495 **Mass:** 59701 **Score:** 237 **Matches:** 11(11) **Sequences:** 9(9) **emPAI:** 0.89
CTP synthase OS=Listeria monocytogenes serovar 1/2a (strain ATCC BAA-679 / EGD-e) OX=169963 GN=pyrG PE=3 SV=1

Query	Observed	Mr(expt)	Mr(calc)	ppm	Miss	Score	Expect	Rank	Unique	Peptide
945	410.731	819.448	819.449	-1.05	0	48	7e-05	1	U	K.DFVGAALK.N
1320	423.244	844.473	844.477	-4.32	0	38	0.00053	1	U	K.GITAASLGR.L
2145	453.754	905.493	905.497	-4.68	0	39	0.00047	1	U	K.IAAIEYAR.V
2988	488.775	975.536	975.539	-3.47	0	22	0.019	1	U	R.FIDINLNK.Y
3021	490.264	978.514	978.521	-6.64	0	30	0.0081	1	U	K.IALFCDIK.A
7318	654.903	1307.791	1307.793	-0.83	0	38	0.00015	1	U	R.SLGIQPNIIVVR.T 7317
13351	899.473	1796.931	1796.931	0.34	0	80	2.7e-08	1	U	K.YVSLQDAYLSVAEALR.H 13350
16529	739.399	2215.175	2215.189	-6.46	0	24	0.0063	1	U	K.GDVGAENVLYIHTTLIPYIK.A
20988	795.911	3179.615	3179.610	1.65	0	17	0.027	1	U	R.NVLGLEGAHSAEIEPETNHNIIDLLPEQK.N

46. tr|Q8Y6Y3 **Mass:** 35595 **Score:** 228 **Matches:** 9(9) **Sequences:** 9(9) **emPAI:** 1.89
MreB protein OS=Listeria monocytogenes serovar 1/2a (strain ATCC BAA-679 / EGD-e) OX=169963 GN=mreB PE=4 SV=1

Query	Observed	Mr(expt)	Mr(calc)	ppm	Miss	Score	Expect	Rank	Unique	Peptide
332	373.211	744.408	744.413	-6.21	0	35	0.0022	1	U	R.AVIDATR.Q
1705	438.727	875.439	875.442	-3.65	0	26	0.012	1	U	R.EPSVVAMK.K
2498	468.232	934.449	934.443	6.02	0	30	0.0029	1	U	K.MEIGSASPK.G
2836	482.265	962.515	962.519	-3.16	0	44	0.00015	1	U	K.YNLLIGDR.T
4624	552.804	1103.594	1103.598	-2.97	0	29	0.0049	1	U	K.GLDLSPFSIR.G
4914	563.852	1125.688	1125.687	1.31	0	49	1.2e-05	1	U	K.GIVLTGGGALLR.N
7371	438.253	1311.738	1311.733	3.27	0	37	0.00033	1	U	R.TPGNIVAIRPMK.D
7662	666.837	1331.659	1331.657	1.56	0	62	2.1e-06	1	U	K.DTQEIVAVGSDAK.N
9498	753.384	1504.753	1504.763	-6.48	0	56	8.2e-06	1	U	R.VMICVPSGITGVEK.R

47. sp|P66054 **Mass:** 14908 **Score:** 225 **Matches:** 4(4) **Sequences:** 4(4) **emPAI:** 2.04
50S ribosomal protein L11 OS=Listeria monocytogenes serovar 1/2a (strain ATCC BAA-679 / EGD-e) OX=169963 GN=rplK PE=3 SV=1

Query	Observed	Mr(expt)	Mr(calc)	ppm	Miss	Score	Expect	Rank	Unique	Peptide
1291	422.234	842.454	842.454	-0.34	0	34	0.00076	1	U	R.SFTFITK.T
2199	455.291	908.568	908.569	-1.83	0	64	4.3e-07	1	U	K.TPPAAVLLK.K
4760	558.797	1115.580	1115.582	-1.78	0	65	1.2e-06	1	U	R.AQVQEIAETK.M
15699	686.382	2056.124	2056.120	1.86	0	108	2.3e-11	1	U	R.TADQAGLIIPVVITVFEDR.S

48. sp|Q8Y444 **Mass:** 19388 **Score:** 214 **Matches:** 7(7) **Sequences:** 7(7) **emPAI:** 3.48
50S ribosomal protein L6 OS=Listeria monocytogenes serovar 1/2a (strain ATCC BAA-679 / EGD-e) OX=169963 GN=rplF PE=3 SV=1

Query	Observed	Mr(expt)	Mr(calc)	ppm	Miss	Score	Expect	Rank	Unique	Peptide
3495	510.294	1018.573	1018.581	-8.39	0	40	0.00033	1	U	K.LELIGVGYR.A
4459	545.793	1089.571	1089.571	0.19	0	42	0.00032	1	U	K.EFNPEITIK.I
5165	574.343	1146.671	1146.676	-4.55	1	57	3.3e-06	1	U	K.KLELIGVGYR.A
5957	604.824	1207.633	1207.631	1.69	0	21	0.026	1	U	K.EHVGELAANIR.A
10373	791.443	1580.871	1580.877	-4.18	0	39	0.00022	1	U	K.GVDIEVPANTQVIVK.G
12620	869.938	1737.861	1737.861	0.05	0	85	8.4e-09	1	U	R.AILNNMVVGVSEGYEK.K
14991	971.564	1941.113	1941.115	-1.07	0	29	0.0013	1	U	K.TIVIPAGVTVTLNGSTATVK.G

49. sp|Q8Y766 **Mass:** 31446 **Score:** 212 **Matches:** 6(6) **Sequences:** 6(6) **emPAI:** 1.23
4-hydroxy-tetrahydrodipicolinate synthase OS=Listeria monocytogenes serovar 1/2a (strain ATCC BAA-679 / EGD-e) OX=169963 GN=dapA PE=3 SV=1

Query	Observed	Mr(expt)	Mr(calc)	ppm	Miss	Score	Expect	Rank	Unique	Peptide
3625	516.258	1030.502	1030.504	-1.85	0	51	1.9e-05	1	U	R.QVIETNDGR.A
4184	536.317	1070.619	1070.634	-13.47	0	76	1.1e-07	1	U	K.LQAILEGLSK.-
5359	582.283	1162.551	1162.547	3.58	0	66	6.2e-07	1	U	K.ESSGNLDNISK.I
8394	699.388	1396.762	1396.756	4.33	0	22	0.015	1	U	R.LPLVDLNAEQGTK.L
9910	772.428	1542.841	1542.852	-6.70	0	55	8.4e-06	1	U	K.YLLNQQGISVGPVR.L
13464	603.650	1807.928	1807.932	-2.08	0	25	0.0092	1	U	K.LIAGTGSNNTAETIAFTK.E

50. sp|Q8Y7J7 **Mass:** 28727 **Score:** 198 **Matches:** 7(6) **Sequences:** 7(6) **emPAI:** 1.40
GTP-sensing transcriptional pleiotropic repressor CodY OS=Listeria monocytogenes serovar 1/2a (strain ATCC BAA-679 / EGD-e) OX=169963 GN=codY PE=3

Query	Observed	Mr(expt)	Mr(calc)	ppm	Miss	Score	Expect	Rank	Unique	Peptide
903	408.742	815.469	815.475	-8.27	0	64	2.8e-06	1	U	K.EGLLVASK.I
1661	436.263	870.511	870.529	-20.52	0	36	0.00072	1	U	R.SVIVNALR.K
1669	436.781	871.548	871.549	-1.06	0	48	2.4e-05	1	U	R.LGTLILSR.L

1714	439.250	876.486	876.496	-10.77	0	18	0.065	1	U	K.FLVELEK.L
5148	573.796	1145.577	1145.586	-8.13	0	31	0.0042	1	U	K.INAMLQNAAGK.T
8095	684.894	1367.774	1367.777	-2.35	0	30	0.0017	1	U	K.GLTTIVPIVGGGER.L
10462	795.425	1588.836	1588.810	16.3	0	71	3e-07	1	U	K.LLGYSEALPIENDR.M

---

51. sp|Q8YA96 **Mass:** 135340 **Score:** 192 **Matches:** 9(9) **Sequences:** 9(9) **emPAI:** 0.33

DNA-directed RNA polymerase subunit beta' OS=Listeria monocytogenes serovar 1/2a (strain ATCC BAA-679 / EGD-e) OX=169963 GN=rpoC PE=3 SV=1

Query	Observed	Mr(expt)	Mr(calc)	ppm	Miss	Score	Expect	Rank	Unique	Peptide
333	373.212	744.409	744.413	-5.52	0	22	0.041	1	U	K.QLLSER.E
1049	415.259	828.503	828.507	-4.17	0	23	0.017	1	U	K.LILSLDR.L
3338	503.267	1004.520	1004.529	-8.98	0	25	0.013	1	U	R.IQEIFEAR.N
3555	513.323	1024.631	1024.628	2.50	0	41	8.6e-05	1	U	R.SVIVVGPNLK.M
6045	608.802	1215.590	1215.577	10.6	0	19	0.027	1	U	K.YFVDTTTDVR.A
8565	707.854	1413.693	1413.699	-4.34	0	27	0.0064	1	U	R.EGTEIIEPLEER.L
8994	727.921	1453.827	1453.833	-3.78	0	76	5.5e-08	1	U	R.ILMLAAQNILNPK.D
9764	765.422	1528.830	1528.825	3.28	0	49	6.5e-05	1	U	R.LGIQAFEPTLVEGR.A
5765	598.312	1791.914	1791.916	-1.05	0	46	0.00024	1	U	R.EGLTVLEYFISTHGAR.K

---

52. sp|Q8YAA3 **Mass:** 12462 **Score:** 188 **Matches:** 5(5) **Sequences:** 5(5) **emPAI:** 4.24

50S ribosomal protein L7/L12 OS=Listeria monocytogenes serovar 1/2a (strain ATCC BAA-679 / EGD-e) OX=169963 GN=rplL PE=3 SV=1

Query	Observed	Mr(expt)	Mr(calc)	ppm	Miss	Score	Expect	Rank	Unique	Peptide
1064	415.751	829.487	829.491	-4.17	0	56	9.6e-06	1	U	R.EITGLGLK.E
1839	443.239	884.464	884.460	4.16	0	23	0.013	1	U	K.ELVDNAPK.A
5654	593.819	1185.623	1185.624	-0.82	0	62	2e-06	1	U	K.LEEVGANVEVK.-
7155	650.377	1298.739	1298.745	-4.05	0	60	2e-06	1	U	M.ALNIEEIIASVK.E
7635	665.366	1328.717	1328.719	-1.02	0	56	1e-05	1	U	K.EASVLELNDLVK.A

---

53. tr|Q8Y864 **Mass:** 35288 **Score:** 185 **Matches:** 7(7) **Sequences:** 7(7) **emPAI:** 1.30

PdhB protein OS=Listeria monocytogenes serovar 1/2a (strain ATCC BAA-679 / EGD-e) OX=169963 GN=PdhB PE=4 SV=1

Query	Observed	Mr(expt)	Mr(calc)	ppm	Miss	Score	Expect	Rank	Unique	Peptide
812	403.244	804.474	804.475	-1.12	1	22	0.018	1	U	R.VKEVIAF.-
1289	421.775	841.535	841.539	-4.26	0	33	0.0011	1	U	K.GLLISAIR.D
2925	486.278	970.541	970.545	-3.39	0	54	1.6e-05	1	U	R.AVVVQEAQK.Q
5837	601.324	1200.634	1200.635	-1.01	0	31	0.003	1	U	K.DGVSVEVIDLR.T
9166	737.355	1472.696	1472.697	-0.47	0	52	1.4e-05	1	U	R.DNDPVIFLEHMK.L
10412	793.456	1584.897	1584.897	-0.38	0	67	2.1e-07	1	U	R.TISPIDVETIIASVK.K
20487	965.122	2892.345	2892.353	-3.05	0	22	0.0064	1	U	R.APFGGGVHTPEMHADNLEGLMAQSPGLK.V

---

54. sp|P33380 **Mass:** 34284 **Score:** 176 **Matches:** 6(6) **Sequences:** 5(5) **emPAI:** 0.84

L-lactate dehydrogenase 1 OS=Listeria monocytogenes serovar 1/2a (strain ATCC BAA-679 / EGD-e) OX=169963 GN=ldh1 PE=3 SV=2

Query	Observed	Mr(expt)	Mr(calc)	ppm	Miss	Score	Expect	Rank	Unique	Peptide
246	365.213	728.411	728.418	-9.62	0	30	0.0024	1	U	R.LDLVNR.N
2758	478.741	955.467	955.468	-1.61	0	31	0.0022	1	U	R.MSIADYLK.V 2757
5389	582.816	1163.617	1163.619	-1.57	1	50	3.4e-05	1	U	R.DAAYEIINKK.G
5508	588.813	1175.611	1175.615	-2.74	0	71	3.1e-07	1	U	R.VIGSGTSLDTAR.F
6714	634.342	1266.670	1266.672	-1.35	0	54	1.1e-05	1	U	K.GATFYGVAAALAR.I

---

55. tr|Q8Y690 **Mass:** 26289 **Score:** 176 **Matches:** 5(4) **Sequences:** 5(4) **emPAI:** 0.89

FabG protein OS=Listeria monocytogenes serovar 1/2a (strain ATCC BAA-679 / EGD-e) OX=169963 GN=fabG PE=1 SV=1

Query	Observed	Mr(expt)	Mr(calc)	ppm	Miss	Score	Expect	Rank	Unique	Peptide
1317	423.242	844.469	844.477	-9.64	0	53	1.9e-05	1	U	K.VAVVTGGSR.G
1487	429.248	856.482	856.502	-23.44	0	18	0.06	1	U	R.DIAINLAK.E
6935	642.866	1283.718	1283.720	-1.07	0	75	1e-07	1	U	R.VDILVNNAGITR.D
18194	822.412	2464.213	2464.216	-1.16	1	54	8.6e-06	1	U	R.GINVNAVAPGFITTDMTDKLDEK.T
16034	1066.198	3195.571	3195.601	-9.44	0	44	7.3e-05	1	U	K.EAMLAQIPLGAYGTTEDIANAVLFLASDASK.Y

---

56. sp|Q8YAF2 **Mass:** 75661 **Score:** 175 **Matches:** 6(6) **Sequences:** 5(5) **emPAI:** 0.32

Methionine--tRNA ligase OS=Listeria monocytogenes serovar 1/2a (strain ATCC BAA-679 / EGD-e) OX=169963 GN=metG PE=3 SV=1

Query	Observed	Mr(expt)	Mr(calc)	ppm	Miss	Score	Expect	Rank	Unique	Peptide
234	364.211	726.407	726.406	0.93	0	18	0.015	1	U	R.YYLLR.E
829	404.221	806.428	806.429	-1.25	0	32	0.0055	1	U	R.YGLDALR.Y
9010	728.905	1455.796	1455.793	2.07	0	59	3.7e-06	1	U	K.LSVIEASSALPNGAK.V 9008
11938	844.442	1686.870	1686.869	0.44	0	68	6.2e-07	1	U	R.VNFDLANDLGNLLNR.T
15884	698.362	2092.064	2092.027	17.8	0	37	0.00062	1	U	R.LVEYYNSHPEFILPESR.K

---

57. sp|Q8Y6T6 **Mass:** 22721 **Score:** 174 **Matches:** 8(8) **Sequences:** 7(7) **emPAI:** 2.61

30S ribosomal protein S4 OS=Listeria monocytogenes serovar 1/2a (strain ATCC BAA-679 / EGD-e) OX=169963 GN=rpsD PE=3 SV=1

Query	Observed	Mr(expt)	Mr(calc)	ppm	Miss	Score	Expect	Rank	Unique	Peptide
435	380.719	759.423	759.424	-1.48	0	32	0.0041	1	U	K.LTGSLNR.L 434
1965	446.744	891.474	891.481	-8.16	0	34	0.0014	1	U	R.LDNIVYR.L
2465	466.774	931.534	931.534	0.22	0	64	1.4e-06	1	U	R.LGISLSGTGK.E
3516	511.744	1021.472	1021.465	7.23	0	22	0.014	1	U	R.HMYGLTER.Q
6684	633.320	1264.625	1264.630	-4.01	0	22	0.021	1	U	K.ISEYGLQQAEK.Q
9083	366.942	1463.740	1463.738	1.33	0	55	7.1e-06	1	U	R.RPYAPGQHGPTQR.K
13868	923.487	1844.960	1845.000	-21.53	0	46	6.8e-05	1	U	R.VDIPSYQVSVGQVISVR.E

---

58. tr|Q8Y6L1 **Mass:** 30176 **Score:** 168 **Matches:** 3(3) **Sequences:** 3(3) **emPAI:** 0.52

1,4-dihydroxy-2-naphthoyl-CoA synthase OS=Listeria monocytogenes serovar 1/2a (strain ATCC BAA-679 / EGD-e) OX=169963 GN=menB PE=3 SV=1

Query	Observed	Mr(expt)	Mr(calc)	ppm	Miss	Score	Expect	Rank	Unique	Peptide
2906	485.788	969.561	969.561	-0.13	0	53	7.8e-06	1	U	R.LNVLDLQR.L

9397	748.875	1495.736	1495.734	1.06	0	61	3.7e-06	1	U	K.TVTEMIDAFNLAR.D
16103	1073.189	3216.545	3216.546	-0.45	0	83	8.3e-09	1	U	K.AAFNADTDGLAGIQQLAGDATLLYYTTDEAK.E

59. tr|Q8Y730 **Mass:** 68922 **Score:** 159 **Matches:** 3(3) **Sequences:** 3(3) **emPAI:** 0.20
Lmo1493 protein OS=Listeria monocytogenes serovar 1/2a (strain ATCC BAA-679 / EGD-e) OX=169963 GN=lmo1493 PE=3 SV=1

Query	Observed	Mr(expt)	Mr(calc)	ppm	Miss	Score	Expect	Rank	Unique	Peptide
1329	423.734	845.453	845.449	4.63	0	40	0.00048	1	U	R.LDEQTLK.S
12411	860.430	1718.845	1718.870	-14.37	0	135	9.8e-14	1	U	K.DVSAGQALNLLNHPDR.A
14996	648.340	1942.000	1942.009	-4.76	0	20	0.025	1	U	K.LSFYDVESPLVFSSKPK.K

60. sp|Q9RLT9 **Mass:** 132692 **Score:** 155 **Matches:** 7(7) **Sequences:** 7(7) **emPAI:** 0.25
DNA-directed RNA polymerase subunit beta OS=Listeria monocytogenes serovar 1/2a (strain ATCC BAA-679 / EGD-e) OX=169963 GN=rpoB PE=3 SV=1

Query	Observed	Mr(expt)	Mr(calc)	ppm	Miss	Score	Expect	Rank	Unique	Peptide
2241	457.290	912.565	912.576	-11.75	0	28	0.0032	1	U	R.VIVSQLVR.S
2884	484.744	967.473	967.476	-3.42	0	20	0.023	1	U	R.SPGVYFNGK.L
3348	503.782	1005.549	1005.549	-0.70	0	42	0.00028	1	U	K.ALLEIYER.L
3865	524.281	1046.547	1046.551	-4.03	0	48	5.5e-05	1	U	K.GFGSTVIPNR.G
13066	885.895	1769.775	1769.778	-1.63	0	32	0.00066	1	U	K.LDEQGTFTEEEVMAR.F
14403	944.946	1887.878	1887.900	-11.67	0	63	9.6e-07	1	U	K.GEILGNGPSMDSGELALGR.N
20882	1043.511	3127.510	3127.492	5.87	0	18	0.028	1	U	R.ILPEEDMPFMPDGTPVDIMLNPLGVPSR.M

61. tr|Q8Y3P3 **Mass:** 29075 **Score:** 148 **Matches:** 4(4) **Sequences:** 4(4) **emPAI:** 0.78
Lmo2792 protein OS=Listeria monocytogenes serovar 1/2a (strain ATCC BAA-679 / EGD-e) OX=169963 GN=lmo2792 PE=4 SV=1

Query	Observed	Mr(expt)	Mr(calc)	ppm	Miss	Score	Expect	Rank	Unique	Peptide
3965	528.807	1055.600	1055.598	2.71	0	54	1.1e-05	1	U	K.SDLSPVIIGR.L
7840	449.279	1344.816	1344.813	1.95	0	36	0.00026	1	U	K.QILPDVHLIIGK.S
9796	767.362	1532.710	1532.739	-19.29	0	56	4.3e-06	1	U	R.VETTVDQPIQEMK.A
16063	712.699	2135.076	2135.053	10.5	0	48	4.3e-05	1	U	K.ALENEGIAHYLESFSASLGK.Y

62. tr|Q8Y7I0 **Mass:** 50714 **Score:** 146 **Matches:** 4(4) **Sequences:** 4(4) **emPAI:** 0.39
Glutamine synthetase OS=Listeria monocytogenes serovar 1/2a (strain ATCC BAA-679 / EGD-e) OX=169963 GN=glnA PE=3 SV=1

Query	Observed	Mr(expt)	Mr(calc)	ppm	Miss	Score	Expect	Rank	Unique	Peptide
6633	630.883	1259.752	1259.749	2.12	0	24	0.0044	1	U	R.LQFTDILGIIK.N
6744	635.772	1269.530	1269.530	0.57	0	48	1.5e-05	1	U	R.NIYGMNEEER.E
9165	737.353	1472.692	1472.697	-3.09	0	73	1.2e-07	1	U	K.IMFDGSSIEGFVR.I
9552	756.892	1511.769	1511.762	4.47	0	47	8e-05	1	U	R.GYTAVTNPTINSFK.R

63. sp|P02662 **Mass:** 24570 **Score:** 145 **Matches:** 5(5) **Sequences:** 3(3) **emPAI:** 0.67
Alpha-S1-casein (Laboratory-Cont) OS=Bos taurus GN=CSN1S1 PE=1 SV=2

Query	Observed	Mr(expt)	Mr(calc)	ppm	Miss	Score	Expect	Rank	Unique	Peptide
1065	416.196	830.377	830.377	0.42	0	36	0.00028	1	U	K.EDVPSER.Y
6716	634.356	1266.697	1266.697	-0.54	0	44	6.8e-05	1	U	R.YLGYLEQLLR.L 6717
8257	692.869	1383.723	1383.723	-0.06	0	46	7.5e-05	1	U	R.FFVAPFPEVFGK.E 8258

64. sp|P66611 **Mass:** 17805 **Score:** 144 **Matches:** 3(3) **Sequences:** 3(3) **emPAI:** 1.02
30S ribosomal protein S7 OS=Listeria monocytogenes serovar 1/2a (strain ATCC BAA-679 / EGD-e) OX=169963 GN=rpsG PE=3 SV=1

Query	Observed	Mr(expt)	Mr(calc)	ppm	Miss	Score	Expect	Rank	Unique	Peptide
4196	536.807	1071.599	1071.600	-0.38	0	28	0.005	1	U	K.NIMPLLEVK.A
8428	701.376	1400.737	1400.741	-2.90	0	78	5.8e-08	1	U	R.VGGANYQVPIEVR.A
8783	718.836	1435.658	1435.661	-2.20	0	72	1.2e-07	1	U	R.EIMDAANNTGASVK.K

65. tr|Q8Y8T0 **Mass:** 32527 **Score:** 144 **Matches:** 3(3) **Sequences:** 3(3) **emPAI:** 0.47
Lmo0814 protein OS=Listeria monocytogenes serovar 1/2a (strain ATCC BAA-679 / EGD-e) OX=169963 GN=lmo0814 PE=4 SV=1

Query	Observed	Mr(expt)	Mr(calc)	ppm	Miss	Score	Expect	Rank	Unique	Peptide
4576	550.811	1099.608	1099.624	-13.97	0	74	1.5e-07	1	U	K.VGIVTTGAGTPK.T
12771	876.519	1751.023	1751.027	-1.83	0	51	8.6e-06	1	U	K.EAGIIVIPVVPSVMIAK.R
17150	768.397	2302.170	2302.129	17.7	1	51	2.1e-05	1	U	R.LEEENADRDTLEELTLGSLR.K

66. sp|O53083 **Mass:** 13109 **Score:** 141 **Matches:** 4(4) **Sequences:** 4(4) **emPAI:** 2.52
50S ribosomal protein L19 OS=Listeria monocytogenes serovar 1/2a (strain ATCC BAA-679 / EGD-e) OX=169963 GN=rplS PE=3 SV=1

Query	Observed	Mr(expt)	Mr(calc)	ppm	Miss	Score	Expect	Rank	Unique	Peptide
1070	416.239	830.463	830.475	-14.58	0	33	0.0025	1	U	K.LIDEITK.S
2803	480.757	959.499	959.504	-4.60	0	46	0.00011	1	U	K.ISNSVGVER.T
5050	569.298	1136.582	1136.583	-0.87	0	58	5.3e-06	1	U	R.GAGISETFTVR.K
14711	637.995	1910.964	1910.960	2.36	0	59	3.4e-06	1	U	K.SQLNPDVPNFRPGDTVR.V

67. sp|Q9RQI6 **Mass:** 21605 **Score:** 137 **Matches:** 5(5) **Sequences:** 5(5) **emPAI:** 1.62
ATP-dependent Clp protease proteolytic subunit OS=Listeria monocytogenes serovar 1/2a (strain ATCC BAA-679 / EGD-e) OX=169963 GN=clpP PE=1 SV=1

Query	Observed	Mr(expt)	Mr(calc)	ppm	Miss	Score	Expect	Rank	Unique	Peptide
1859	444.226	886.438	886.418	22.1	0	27	0.0048	1	U	R.AYDIYSR.L
4995	567.301	1132.588	1132.588	0.01	0	47	6.8e-05	1	U	K.TGQPYEVIAR.D
8329	696.370	1390.726	1390.734	-5.83	0	59	6.2e-06	1	U	K.DYGLIDDIIINK.S
9601	759.402	1516.790	1516.792	-1.56	0	46	0.00011	1	U	-.MNLIPTVIEQTSR.G
11435	829.355	1656.696	1656.705	-5.51	1	27	0.0021	1	U	R.DTDRDNFMTAQEAK.D

68. tr|Q8YAW1 **Mass:** 42403 **Score:** 135 **Matches:** 4(4) **Sequences:** 3(3) **emPAI:** 0.35
Beta sliding clamp OS=Listeria monocytogenes serovar 1/2a (strain ATCC BAA-679 / EGD-e) OX=169963 GN=dnaN PE=3 SV=1

Query	Observed	Mr(expt)	Mr(calc)	ppm	Miss	Score	Expect	Rank	Unique	Peptide

2478	467.259	932.503	932.508	-4.94	0	33	0.0039	1	U	K.AFLQAIDR.A
4937	564.823	1127.630	1127.630	0.55	0	44	0.00013	1	U	R.LVQAVNEVTR.A
13002	882.473	1762.931	1762.958	-15.05	0	66	7.8e-07	1	U	K.DAANPNEILQLITPVR.T
13003	588.659	1762.955	1762.958	-1.39	0	(45)	5.3e-05	1	U	K.DAANPNEILQLITPVR.T

69. sp|P66699 **Mass:** 34941 **Score:** 133 **Matches:** 5(5) **Sequences:** 5(5) **emPAI:** 0.82
DNA-directed RNA polymerase subunit alpha OS=Listeria monocytogenes serovar 1/2a (strain ATCC BAA-679 / EGD-e) OX=169963 GN=rpoA PE=3 SV=1

Query	Observed	Mr(expt)	Mr(calc)	ppm	Miss	Score	Expect	Rank	Unique	Peptide
2772	479.284	956.554	956.565	-12.24	0	49	5.2e-05	1	U	K.LADLGLSLR.N
3130	494.776	987.537	987.539	-1.46	0	28	0.0081	1	U	K.FVVEPLER.G
4840	561.784	1121.553	1121.547	5.41	0	17	0.056	1	U	R.VNYQVENTR.V
5066	569.793	1137.572	1137.578	-4.74	0	25	0.014	1	U	R.GYGTTLGNSLR.R
15909	700.381	2098.120	2098.113	3.14	0	80	1.6e-08	1	U	R.ENMPIGVLPVDSIFSPVIR.V

70. sp|P0DJP0 **Mass:** 37971 **Score:** 132 **Matches:** 4(4) **Sequences:** 4(4) **emPAI:** 0.56
Protein RecA OS=Listeria monocytogenes serovar 1/2a (strain ATCC BAA-679 / EGD-e) OX=169963 GN=recA PE=3 SV=1

Query	Observed	Mr(expt)	Mr(calc)	ppm	Miss	Score	Expect	Rank	Unique	Peptide
1106	417.726	833.437	833.443	-7.05	0	42	0.00026	1	U	R.LMSQALR.K
2770	479.266	956.518	956.529	-11.94	0	55	8.7e-06	1	U	R.QAALDQALK.Q
9931	773.888	1545.762	1545.761	0.53	0	36	0.00092	1	U	K.VGVMFGNPEITPGGR.A
5804	600.289	1797.844	1797.832	7.08	0	52	4.1e-05	1	U	R.AEIEGEMGDAHVGLQAR.L

71. sp|Q8Y634 **Mass:** 30173 **Score:** 128 **Matches:** 3(3) **Sequences:** 3(3) **emPAI:** 0.52
Putative pyruvate, phosphate dikinase regulatory protein 2 OS=Listeria monocytogenes serovar 1/2a (strain ATCC BAA-679 / EGD-e) OX=169963 GN=lmo18[illegible]

Query	Observed	Mr(expt)	Mr(calc)	ppm	Miss	Score	Expect	Rank	Unique	Peptide
2812	481.291	960.568	960.564	3.39	0	32	0.00083	1	U	R.IAAIEFAVK.Y
8694	713.914	1425.814	1425.819	-3.32	0	35	0.0005	1	U	R.GLLDADIVLVGVSR.T
18446	838.145	2511.413	2511.410	1.20	0	89	1.3e-09	1	U	K.VANVPLVPEIPIPAELFQIPAER.I

72. sp|P66383 **Mass:** 13701 **Score:** 127 **Matches:** 4(4) **Sequences:** 4(4) **emPAI:** 2.35
30S ribosomal protein S13 OS=Listeria monocytogenes serovar 1/2a (strain ATCC BAA-679 / EGD-e) OX=169963 GN=rpsM PE=3 SV=1

Query	Observed	Mr(expt)	Mr(calc)	ppm	Miss	Score	Expect	Rank	Unique	Peptide
1005	413.742	825.469	825.471	-2.12	0	44	5.4e-05	1	U	R.IAGVDVPR.E
1047	415.241	828.468	828.470	-2.73	0	19	0.038	1	U	R.EVNLNIK.R
2686	475.767	949.519	949.523	-4.41	0	35	0.0011	1	U	R.LIEIGSYR.G
8157	688.341	1374.667	1374.663	2.98	0	78	3.9e-08	1	U	K.EVLAEAGVSEDTR.T

73. sp|P02666 **Mass:** 25148 **Score:** 126 **Matches:** 3(3) **Sequences:** 3(3) **emPAI:** 0.65
Beta-casein (Laboratory-Cont) OS=Bos taurus GN=CSN2 PE=1 SV=2

Query	Observed	Mr(expt)	Mr(calc)	ppm	Miss	Score	Expect	Rank	Unique	Peptide
314	371.729	741.443	741.443	0.25	0	36	0.00038	1	U	R.GPFPIIV.-
589	390.754	779.493	779.491	2.78	0	27	0.002	1	U	K.VLPVPQK.A
16445	734.726	2201.155	2201.155	-0.27	0	90	1.4e-09	1	U	R.DMPIQAFLLYQEPVLGPVR.G

74. tr|Q7AP52 **Mass:** 62606 **Score:** 123 **Matches:** 3(3) **Sequences:** 3(3) **emPAI:** 0.22
Lmo2196 protein OS=Listeria monocytogenes serovar 1/2a (strain ATCC BAA-679 / EGD-e) OX=169963 GN=lmo2196 PE=4 SV=1

Query	Observed	Mr(expt)	Mr(calc)	ppm	Miss	Score	Expect	Rank	Unique	Peptide
6881	640.822	1279.629	1279.641	-9.13	0	56	9.8e-06	1	U	R.TVLSADYAAQNK.N
15125	980.496	1958.978	1958.963	7.65	0	59	6.1e-06	1	U	R.AVDPNTAATYSYLFDAIK.N
16621	744.386	2230.137	2230.133	1.76	0	42	0.00018	1	U	K.ASGEQVLNLTESALIPSADSTK.A

75. sp|Q8Y5E6 **Mass:** 48628 **Score:** 116 **Matches:** 2(2) **Sequences:** 2(2) **emPAI:** 0.19
Phosphoglucosamine mutase OS=Listeria monocytogenes serovar 1/2a (strain ATCC BAA-679 / EGD-e) OX=169963 GN=glmM PE=3 SV=1

Query	Observed	Mr(expt)	Mr(calc)	ppm	Miss	Score	Expect	Rank	Unique	Peptide
9479	752.393	1502.770	1502.773	-1.60	0	67	1.4e-06	1	U	R.GVANSELTPELAFR.L
10252	787.390	1572.766	1572.763	1.90	0	66	8.5e-07	1	U	K.ELEIEDVQTAVGDR.Y

76. sp|Q8Y450 **Mass:** 15205 **Score:** 115 **Matches:** 7(7) **Sequences:** 3(3) **emPAI:** 1.26
50S ribosomal protein L17 OS=Listeria monocytogenes serovar 1/2a (strain ATCC BAA-679 / EGD-e) OX=169963 GN=rplQ PE=3 SV=1

Query	Observed	Mr(expt)	Mr(calc)	ppm	Miss	Score	Expect	Rank	Unique	Peptide
2460	466.744	931.474	931.476	-2.65	0	23	0.021	1	U	K.LFDDVAPR.Y 2462
7060	646.348	1290.682	1290.682	-0.21	0	52	2.1e-05	1	U	R.DLATDLIVFER.I 7058 7059 7061
8275	693.375	1384.736	1384.738	-2.07	1	53	3.3e-05	1	U	R.RGDGAPMVIIELV.-

77. tr|Q8Y5B3 **Mass:** 40192 **Score:** 111 **Matches:** 5(5) **Sequences:** 4(4) **emPAI:** 0.52
Ribonucleoside-diphosphate reductase subunit beta OS=Listeria monocytogenes serovar 1/2a (strain ATCC BAA-679 / EGD-e) OX=169963 GN=lmo2154 PE=3 S[illegible]

Query	Observed	Mr(expt)	Mr(calc)	ppm	Miss	Score	Expect	Rank	Unique	Peptide
272	367.714	733.413	733.412	0.44	0	29	0.0051	1	U	R.VFELAR.T
351	374.209	746.403	746.417	-19.23	0	30	0.0063	1	U	K.TGETLVK.A
1542	431.757	861.499	861.507	-10.06	0	44	8.5e-05	1	U	K.IISFINR.D
4554	549.819	1097.624	1097.623	0.62	0	54	5.5e-06	1	U	K.ILEPLFPNR.S 4555

78. sp|P66352 **Mass:** 13834 **Score:** 107 **Matches:** 4(4) **Sequences:** 4(4) **emPAI:** 2.29
30S ribosomal protein S11 OS=Listeria monocytogenes serovar 1/2a (strain ATCC BAA-679 / EGD-e) OX=169963 GN=rpsK PE=3 SV=1

Query	Observed	Mr(expt)	Mr(calc)	ppm	Miss	Score	Expect	Rank	Unique	Peptide
684	395.239	788.464	788.464	-1.02	0	23	0.015	2	U	K.TLEVTVK.G
4683	555.306	1108.597	1108.599	-1.29	0	27	0.015	1	U	K.NIESGIAHIR.S
6938	642.879	1283.743	1283.745	-1.73	0	63	9.6e-07	1	U	R.ALQAAGLEVTAIK.D

9396	748.858	1495.701	1495.698	2.41	0	42	0.00016	1	U	K.STPFAAQMAAESAAK.S

79. tr|Q8Y7F9 **Mass:** 41715 **Score:** 105 **Matches:** 2(2) **Sequences:** 2(2) **emPAI:** 0.22
Transcription termination/antitermination protein NusA OS=Listeria monocytogenes serovar 1/2a (strain ATCC BAA-679 / EGD-e) OX=169963 GN=nusA PE=3

Query	Observed	Mr(expt)	Mr(calc)	ppm	Miss	Score	Expect	Rank	Unique	Peptide
8776	718.370	1434.726	1434.735	-6.33	0	32	0.0057	1	U	K.SETVATELGIYPR.N
12230	853.962	1705.909	1705.914	-2.98	0	89	4e-09	1	U	R.EVLVEAIEAALTSAYK.R

80. sp|Q8Y4I3 **Mass:** 27090 **Score:** 104 **Matches:** 4(3) **Sequences:** 4(3) **emPAI:** 0.59
Triosephosphate isomerase 1 OS=Listeria monocytogenes serovar 1/2a (strain ATCC BAA-679 / EGD-e) OX=169963 GN=tpiA1 PE=3 SV=1

Query	Observed	Mr(expt)	Mr(calc)	ppm	Miss	Score	Expect	Rank	Unique	Peptide
2107	452.738	903.462	903.466	-4.72	0	18	0.08	1	U	R.LTEGTDLR.V
3468	509.261	1016.508	1016.514	-5.47	0	66	9.6e-07	1	U	R.AEVADAVSQK.A
7794	671.881	1341.747	1341.750	-2.46	0	30	0.0032	1	U	R.AALAGLTEEQVIK.S
18925	875.803	2624.388	2624.381	2.74	0	41	0.00011	1	U	K.NNVPSSDAVESVVAAPALFLQELVR.L

81. sp|Q8Y4C0 **Mass:** 55110 **Score:** 101 **Matches:** 6(5) **Sequences:** 6(5) **emPAI:** 0.47
ATP synthase subunit alpha 2 OS=Listeria monocytogenes serovar 1/2a (strain ATCC BAA-679 / EGD-e) OX=169963 GN=atpA2 PE=3 SV=1

Query	Observed	Mr(expt)	Mr(calc)	ppm	Miss	Score	Expect	Rank	Unique	Peptide
773	401.722	801.429	801.434	-7.19	0	20	0.057	1	U	R.NAVETLR.H
965	411.226	820.438	820.444	-7.18	0	27	0.021	1	U	R.LDLAAYR.E
1295	422.267	842.519	842.523	-3.98	0	27	0.0051	1	U	R.ELSLLLR.R
3542	512.811	1023.607	1023.608	-0.82	0	46	2.6e-05	1	U	K.AIDALVPIGR.G
8411	700.381	1398.748	1398.754	-4.07	0	39	0.0004	1	U	K.IMEVPVGEALIGR.V
10772	811.917	1621.819	1621.831	-7.39	0	26	0.007	1	U	K.VSDVGTVTYIGDGIAR.A

82. tr|Q8Y6Q0 **Mass:** 95135 **Score:** 100 **Matches:** 5(5) **Sequences:** 5(5) **emPAI:** 0.25
Aldehyde-alcohol dehydrogenase OS=Listeria monocytogenes serovar 1/2a (strain ATCC BAA-679 / EGD-e) OX=169963 GN=lmo1634 PE=3 SV=1

Query	Observed	Mr(expt)	Mr(calc)	ppm	Miss	Score	Expect	Rank	Unique	Peptide
641	393.259	784.503	784.506	-3.17	0	36	0.00063	1	U	K.ILVAEIK.G
5322	580.271	1158.527	1158.552	-21.21	0	22	0.032	1	U	R.ESVLTGDPDAR.E
5883	602.329	1202.643	1202.666	-18.75	0	36	0.00098	1	U	R.AIELVFENLR.E
7967	679.347	1356.680	1356.688	-6.30	0	50	3.5e-05	1	U	K.ENAAQEVLEVQK.V
9398	748.895	1495.776	1495.775	0.91	0	27	0.005	1	U	R.VFIVTDPGMVQFK.Y

83. sp|Q48793 **Mass:** 35275 **Score:** 100 **Matches:** 2(2) **Sequences:** 1(1) **emPAI:** 0.13
Ribose-phosphate pyrophosphokinase 1 OS=Listeria monocytogenes serovar 1/2a (strain ATCC BAA-679 / EGD-e) OX=169963 GN=prs1 PE=3 SV=1

Query	Observed	Mr(expt)	Mr(calc)	ppm	Miss	Score	Expect	Rank	Unique	Peptide
7619	664.873	1327.731	1327.746	-11.40	0	65	1.1e-06	1	U	K.LVANLIETAGATR.M 7620

84. sp|Q8Y573 **Mass:** 34006 **Score:** 97 **Matches:** 3(3) **Sequences:** 3(3) **emPAI:** 0.45
3-oxoacyl-[acyl-carrier-protein] synthase 3 OS=Listeria monocytogenes serovar 1/2a (strain ATCC BAA-679 / EGD-e) OX=169963 GN=fabH PE=3 SV=1

Query	Observed	Mr(expt)	Mr(calc)	ppm	Miss	Score	Expect	Rank	Unique	Peptide
1250	421.736	841.457	841.454	2.77	0	37	0.00023	1	U	R.LNLPEEK.L
4284	539.789	1077.564	1077.571	-5.95	0	50	5e-05	1	U	K.IVTNFDLEK.I
16102	537.046	2144.156	2144.159	-1.45	1	42	0.0001	1	U	R.AGLEKEDLDLLIPHQANIR.I

85. sp|Q8Y6Y9 **Mass:** 11207 **Score:** 97 **Matches:** 2(2) **Sequences:** 2(2) **emPAI:** 1.08
50S ribosomal protein L21 OS=Listeria monocytogenes serovar 1/2a (strain ATCC BAA-679 / EGD-e) OX=169963 GN=rplU PE=3 SV=1

Query	Observed	Mr(expt)	Mr(calc)	ppm	Miss	Score	Expect	Rank	Unique	Peptide
3176	496.773	991.532	991.534	-2.19	0	38	0.00063	1	U	K.VLFVGGDSAK.V
8148	687.880	1373.746	1373.755	-6.79	0	77	6.3e-08	1	U	K.VGVPFVEGATVTAK.V

86. sp|Q8Y441 **Mass:** 10909 **Score:** 94 **Matches:** 2(2) **Sequences:** 2(2) **emPAI:** 1.12
50S ribosomal protein L23 OS=Listeria monocytogenes serovar 1/2a (strain ATCC BAA-679 / EGD-e) OX=169963 GN=rplW PE=3 SV=1

Query	Observed	Mr(expt)	Mr(calc)	ppm	Miss	Score	Expect	Rank	Unique	Peptide
1820	442.232	882.449	882.427	24.9	0	34	0.00094	1	U	K.VNVMNYK.G
13543	606.325	1815.954	1815.958	-2.23	1	77	5.2e-08	1	U	K.RPVVTEESTSILDDKK.Y

87. sp|Q8Y493 **Mass:** 62797 **Score:** 90 **Matches:** 3(3) **Sequences:** 3(3) **emPAI:** 0.22
Arginine--tRNA ligase OS=Listeria monocytogenes serovar 1/2a (strain ATCC BAA-679 / EGD-e) OX=169963 GN=argS PE=3 SV=1

Query	Observed	Mr(expt)	Mr(calc)	ppm	Miss	Score	Expect	Rank	Unique	Peptide
1737	440.220	878.425	878.413	13.7	0	19	0.047	1	U	R.ADLEEFR.V
7143	649.867	1297.720	1297.724	-3.04	0	57	5.9e-06	1	U	R.NGLTLLGVSAPEK.M
9328	745.397	1488.779	1488.782	-2.14	0	49	4.9e-05	1	U	K.VLGEFADVVAEAAAK.R

88. sp|Q927L7 **Mass:** 13332 **Score:** 89 **Matches:** 5(4) **Sequences:** 4(4) **emPAI:** 2.45
50S ribosomal protein L14 OS=Listeria monocytogenes serovar 1/2a (strain ATCC BAA-679 / EGD-e) OX=169963 GN=rplN PE=3 SV=1

Query	Observed	Mr(expt)	Mr(calc)	ppm	Miss	Score	Expect	Rank	Unique	Peptide
26	351.723	701.432	701.432	0.00	0	33	0.0019	1	U	R.EVLTIK.V
428	380.230	758.446	758.444	2.79	0	23	0.035	1	U	R.IFGPVAR.E 427
1474	428.748	855.482	855.481	0.25	0	47	3.7e-05	1	U	K.QATPGGVVK.K
2580	470.788	939.562	939.564	-1.83	0	37	0.00021	1	U	K.IVSLAPEVL.-

89. sp|Q8Y754 **Mass:** 78516 **Score:** 88 **Matches:** 4(4) **Sequences:** 4(4) **emPAI:** 0.24
Glycine--tRNA ligase beta subunit OS=Listeria monocytogenes serovar 1/2a (strain ATCC BAA-679 / EGD-e) OX=169963 GN=glyS PE=3 SV=1

Query	Observed	Mr(expt)	Mr(calc)	ppm	Miss	Score	Expect	Rank	Unique	Peptide

441	381.221	760.427	760.433	-8.06	0	23	0.016	1	U	K.LGTLTEK.M
3654	517.280	1032.545	1032.524	20.0	0	29	0.0054	1	U	K.VDPADLTFR.D
12362	572.334	1713.982	1713.978	2.28	0	68	2.8e-07	1	U	K.YALLQGEKPAIATAIR.E
16910	757.406	2269.198	2269.207	-3.98	0	20	0.028	1	U	R.HDIIDAVIGGDPNVIPQLIDR.A

---

90. sp|Q927L2 **Mass:** 12866 **Score:** 88 **Matches:** 3(3) **Sequences:** 3(3) **emPAI:** 1.61

50S ribosomal protein L22 OS=Listeria monocytogenes serovar 1/2a (strain ATCC BAA-679 / EGD-e) OX=169963 GN=rplV PE=3 SV=1

Query	Observed	Mr(expt)	Mr(calc)	ppm	Miss	Score	Expect	Rank	Unique	Peptide
1249	421.281	840.547	840.543	3.92	0	34	0.0004	1	U	R.IVIDLIR.G
1299	422.735	843.455	843.470	-18.12	0	27	0.0079	1	U	R.SASPIIEK.V
3769	521.316	1040.617	1040.623	-5.69	0	59	2.6e-06	1	U	K.QVGEAIAILK.Y

---

91. sp|Q8Y709 **Mass:** 66455 **Score:** 88 **Matches:** 5(4) **Sequences:** 5(4) **emPAI:** 0.29

Aspartate--tRNA ligase OS=Listeria monocytogenes serovar 1/2a (strain ATCC BAA-679 / EGD-e) OX=169963 GN=aspS PE=3 SV=1

Query	Observed	Mr(expt)	Mr(calc)	ppm	Miss	Score	Expect	Rank	Unique	Peptide
1355	425.227	848.440	848.439	0.78	0	25	0.014	1	U	R.DYLVPSR.V
4601	551.791	1101.566	1101.567	-0.08	0	35	0.0012	1	U	K.EALEIADSVR.N
8925	724.395	1446.774	1446.772	1.81	0	43	0.00018	1	U	R.FGLELQNVSDVVK.D
9183	738.367	1474.719	1474.704	10.1	0	18	0.1	1	U	K.EEDIPLLETDSSK.V
15065	975.988	1949.962	1949.937	12.8	0	36	0.00068	1	U	K.TPPFYIEDGVNVSDELR.L

---

92. tr|Q8Y851 **Mass:** 68799 **Score:** 86 **Matches:** 3(3) **Sequences:** 3(3) **emPAI:** 0.20

Lmo1067 protein OS=Listeria monocytogenes serovar 1/2a (strain ATCC BAA-679 / EGD-e) OX=169963 GN=lmo1067 PE=4 SV=1

Query	Observed	Mr(expt)	Mr(calc)	ppm	Miss	Score	Expect	Rank	Unique	Peptide
3007	489.267	976.519	976.516	3.04	0	38	0.00075	1	U	R.LQFMVPAR.G
3978	529.290	1056.564	1056.582	-16.13	0	46	5.4e-05	1	U	R.EGDIAVNIVK.A
9850	769.435	1536.856	1536.888	-20.78	0	37	0.0005	1	U	K.ALEQNLTPIVVVNK.I

---

93. sp|Q8Y7K9 **Mass:** 46768 **Score:** 86 **Matches:** 1(1) **Sequences:** 1(1) **emPAI:** 0.09

ATP-dependent Clp protease ATP-binding subunit ClpX OS=Listeria monocytogenes serovar 1/2a (strain ATCC BAA-679 / EGD-e) OX=169963 GN=clpX PE=3 SV=

Query	Observed	Mr(expt)	Mr(calc)	ppm	Miss	Score	Expect	Rank	Unique	Peptide
7813	672.358	1342.702	1342.709	-5.39	0	86	6.1e-09	1	U	R.DVSGEGVQQALLK.I

---

94. tr|Q8Y4M3 **Mass:** 48072 **Score:** 85 **Matches:** 2(2) **Sequences:** 2(2) **emPAI:** 0.19

Lmo2414 protein OS=Listeria monocytogenes serovar 1/2a (strain ATCC BAA-679 / EGD-e) OX=169963 GN=lmo2414 PE=4 SV=1

Query	Observed	Mr(expt)	Mr(calc)	ppm	Miss	Score	Expect	Rank	Unique	Peptide
4575	550.801	1099.588	1099.587	0.53	0	53	1.5e-05	1	U	K.VANLVDLDNK.E
8066	683.843	1365.671	1365.682	-7.61	0	50	3.7e-05	1	U	K.AYGELDLPFVDK.T

---

95. sp|Q8Y7N6 **Mass:** 26767 **Score:** 83 **Matches:** 2(2) **Sequences:** 2(2) **emPAI:** 0.37

Ribonuclease PH OS=Listeria monocytogenes serovar 1/2a (strain ATCC BAA-679 / EGD-e) OX=169963 GN=rph PE=3 SV=1

Query	Observed	Mr(expt)	Mr(calc)	ppm	Miss	Score	Expect	Rank	Unique	Peptide
3143	495.268	988.521	988.523	-2.39	0	36	0.0024	1	U	K.LDEAVPFAK.F
5302	579.313	1156.611	1156.609	1.48	0	65	2.2e-06	1	U	R.AVVDLDALGER.T

---

96. tr|Q8Y4U6 **Mass:** 66230 **Score:** 80 **Matches:** 3(3) **Sequences:** 3(3) **emPAI:** 0.21

FruA protein OS=Listeria monocytogenes serovar 1/2a (strain ATCC BAA-679 / EGD-e) OX=169963 GN=fruA PE=4 SV=1

Query	Observed	Mr(expt)	Mr(calc)	ppm	Miss	Score	Expect	Rank	Unique	Peptide
684	395.239	788.464	788.464	-1.00	0	20	0.027	3	U	R.ITDLLSK.D
3005	489.265	976.515	976.523	-7.83	0	19	0.054	1	U	K.INDEVLFK.K
14014	930.161	2787.460	2787.444	5.53	0	75	5.7e-08	1	U	R.DAGLTNSILGASFITEGAIPFAAADPLR.M

---

97. sp|Q8Y459 **Mass:** 14363 **Score:** 77 **Matches:** 3(3) **Sequences:** 3(3) **emPAI:** 1.38

30S ribosomal protein S9 OS=Listeria monocytogenes serovar 1/2a (strain ATCC BAA-679 / EGD-e) OX=169963 GN=rpsI PE=3 SV=1

Query	Observed	Mr(expt)	Mr(calc)	ppm	Miss	Score	Expect	Rank	Unique	Peptide
5636	592.783	1183.551	1183.562	-9.07	0	21	0.018	1	U	M.AQVQYYGTGR.R
9387	748.362	1494.710	1494.714	-3.11	0	19	0.026	1	U	R.DWEDYIPFAALR.E
10191	523.644	1567.911	1567.909	1.31	0	67	2.3e-07	1	U	R.ALLQVAPEYRPALK.S

---

98. sp|P65927 **Mass:** 26150 **Score:** 76 **Matches:** 5(5) **Sequences:** 5(5) **emPAI:** 1.22

Uridylate kinase OS=Listeria monocytogenes serovar 1/2a (strain ATCC BAA-679 / EGD-e) OX=169963 GN=pyrH PE=3 SV=1

Query	Observed	Mr(expt)	Mr(calc)	ppm	Miss	Score	Expect	Rank	Unique	Peptide
2850	483.241	964.467	964.465	1.87	0	31	0.0042	1	U	R.VQTSIDMR.Q
3147	495.277	988.540	988.534	5.95	0	24	0.023	1	U	R.QIAEPYIR.R
7236	653.303	1304.591	1304.600	-6.35	0	31	0.0013	1	U	K.NNVDGVYNADPK.L
8298	695.365	1388.716	1388.722	-4.40	0	36	0.00097	1	U	R.AAEIEADVILMAK.N
9437	750.386	1498.758	1498.792	-22.34	1	26	0.0048	1	U	K.KYEELSYLDVIK.E

---

99. tr|Q8YAB6 **Mass:** 91166 **Score:** 75 **Matches:** 2(2) **Sequences:** 2(2) **emPAI:** 0.10

Endopeptidase Clp ATP-binding chain C OS=Listeria monocytogenes serovar 1/2a (strain ATCC BAA-679 / EGD-e) OX=169963 GN=clpC PE=3 SV=1

Query	Observed	Mr(expt)	Mr(calc)	ppm	Miss	Score	Expect	Rank	Unique	Peptide
7981	679.368	1356.722	1356.725	-2.42	0	73	2e-07	1	U	R.VAITDEALEAAVR.L
11223	549.320	1644.938	1644.935	1.49	0	18	0.02	1	U	K.RPIGSFIFLGPTGVGK.T

---

100. tr|Q8YAC3 **Mass:** 32198 **Score:** 72 **Matches:** 1(1) **Sequences:** 1(1) **emPAI:** 0.14

Cysteine synthase OS=Listeria monocytogenes serovar 1/2a (strain ATCC BAA-679 / EGD-e) OX=169963 GN=cysK PE=3 SV=1

Query	Observed	Mr(expt)	Mr(calc)	ppm	Miss	Score	Expect	Rank	Unique	Peptide

10855	816.435	1630.855	1630.857	-0.73	0	72	4.1e-07	1	U	K.IQGIGAGFVPDTLDTK.V

---

101. sp|Q8Y6X9 **Mass:** 102200 **Score:** 71 **Matches:** 2(2) **Sequences:** 2(2) **emPAI:** 0.09

Valine--tRNA ligase OS=Listeria monocytogenes serovar 1/2a (strain ATCC BAA-679 / EGD-e) OX=169963 GN=valS PE=3 SV=1

Query	Observed	Mr(expt)	Mr(calc)	ppm	Miss	Score	Expect	Rank	Unique	Peptide
3067	492.274	982.534	982.549	-14.63	0	26	0.004	1	U	K.VFVTLYNK.G
8810	719.366	1436.718	1436.715	2.22	0	63	3.9e-06	1	U	R.YTLATGSSPGQDLK.F

---

102. tr|Q8YAJ0 **Mass:** 58308 **Score:** 71 **Matches:** 3(2) **Sequences:** 3(2) **emPAI:** 0.16

Lmo0135 protein OS=Listeria monocytogenes serovar 1/2a (strain ATCC BAA-679 / EGD-e) OX=169963 GN=lmo0135 PE=1 SV=1

Query	Observed	Mr(expt)	Mr(calc)	ppm	Miss	Score	Expect	Rank	Unique	Peptide
1364	425.247	848.479	848.480	-1.20	0	20	0.069	1	U	K.TFFPIPK.H
8923	724.377	1446.740	1446.747	-4.91	0	39	0.00041	1	U	K.ASAVNIITYPENR.L
10386	792.404	1582.793	1582.773	12.8	0	46	0.0001	1	U	K.SLDISDDNLTYTVK.L

---

103. sp|P66372 **Mass:** 15231 **Score:** 71 **Matches:** 3(3) **Sequences:** 3(3) **emPAI:** 1.26

30S ribosomal protein S12 OS=Listeria monocytogenes serovar 1/2a (strain ATCC BAA-679 / EGD-e) OX=169963 GN=rpsL PE=3 SV=1

Query	Observed	Mr(expt)	Mr(calc)	ppm	Miss	Score	Expect	Rank	Unique	Peptide
1825	442.271	882.526	882.529	-2.51	1	18	0.016	1	U	R.VKDLPGVR.Y
2579	470.781	939.548	939.550	-2.69	0	42	6.9e-05	1	U	M.PTINQLVR.K
4958	565.785	1129.556	1129.562	-5.31	0	40	0.00027	1	U	R.ELTDVNSPQK.R

---

104. tr|Q8Y786 **Mass:** 83943 **Score:** 70 **Matches:** 3(3) **Sequences:** 3(3) **emPAI:** 0.16

Pyruvate formate-lyase OS=Listeria monocytogenes serovar 1/2a (strain ATCC BAA-679 / EGD-e) OX=169963 GN=pflB PE=4 SV=1

Query	Observed	Mr(expt)	Mr(calc)	ppm	Miss	Score	Expect	Rank	Unique	Peptide
2781	479.778	957.542	957.540	2.66	0	31	0.0033	1	U	R.ALQPFGGIR.M
3305	502.291	1002.568	1002.571	-3.38	0	39	0.00044	1	U	K.GALASLSSVAK.L
7248	653.346	1304.678	1304.672	4.52	0	36	0.001	1	U	K.SGVITGLPDAYGR.G

---

105. sp|Q8Y6M4 **Mass:** 91895 **Score:** 68 **Matches:** 2(2) **Sequences:** 2(2) **emPAI:** 0.10

Leucine--tRNA ligase OS=Listeria monocytogenes serovar 1/2a (strain ATCC BAA-679 / EGD-e) OX=169963 GN=leuS PE=3 SV=1

Query	Observed	Mr(expt)	Mr(calc)	ppm	Miss	Score	Expect	Rank	Unique	Peptide
840	405.204	808.393	808.408	-18.54	0	48	7.1e-05	1	U	K.ITAYADR.L
5492	588.292	1174.570	1174.583	-11.39	0	39	0.00041	1	U	K.VTTDANANLEK.A

---

106. sp|Q8YAD3 **Mass:** 22641 **Score:** 66 **Matches:** 1(1) **Sequences:** 1(1) **emPAI:** 0.20

50S ribosomal protein L25 OS=Listeria monocytogenes serovar 1/2a (strain ATCC BAA-679 / EGD-e) OX=169963 GN=rplY PE=3 SV=1

Query	Observed	Mr(expt)	Mr(calc)	ppm	Miss	Score	Expect	Rank	Unique	Peptide
3940	527.316	1052.617	1052.623	-5.69	0	66	4e-07	1	U	R.VVLVGDAPGVK.A

---

107. tr|Q8Y6T2 **Mass:** 39831 **Score:** 66 **Matches:** 2(2) **Sequences:** 2(2) **emPAI:** 0.24

3-deoxy-D-arabino-heptulosonate 7-phosphate synthase OS=Listeria monocytogenes serovar 1/2a (strain ATCC BAA-679 / EGD-e) OX=169963 GN=aroA PE=1 S\

Query	Observed	Mr(expt)	Mr(calc)	ppm	Miss	Score	Expect	Rank	Unique	Peptide
4409	544.293	1086.571	1086.567	3.88	0	28	0.0055	1	U	M.VNTNLEELR.T
6928	642.380	1282.745	1282.750	-3.59	0	54	6.8e-06	1	U	R.NTLDISAVPILK.K

---

108. sp|Q8Y458 **Mass:** 16190 **Score:** 66 **Matches:** 1(1) **Sequences:** 1(1) **emPAI:** 0.29

50S ribosomal protein L13 OS=Listeria monocytogenes serovar 1/2a (strain ATCC BAA-679 / EGD-e) OX=169963 GN=rplM PE=3 SV=1

Query	Observed	Mr(expt)	Mr(calc)	ppm	Miss	Score	Expect	Rank	Unique	Peptide
4226	537.810	1073.606	1073.608	-1.51	0	66	1.1e-06	1	U	R.LSSEVASILR.G

---

109. sp|Q8Y6M0 **Mass:** 43638 **Score:** 66 **Matches:** 1(1) **Sequences:** 1(1) **emPAI:** 0.10

S-adenosylmethionine synthase OS=Listeria monocytogenes serovar 1/2a (strain ATCC BAA-679 / EGD-e) OX=169963 GN=metK PE=3 SV=1

Query	Observed	Mr(expt)	Mr(calc)	ppm	Miss	Score	Expect	Rank	Unique	Peptide
8724	715.392	1428.769	1428.773	-2.18	0	66	7.6e-07	1	U	R.FVIGGPLGDAGLTGR.K

---

110. tr|Q8Y9H0 **Mass:** 37533 **Score:** 65 **Matches:** 3(3) **Sequences:** 3(3) **emPAI:** 0.40

Lmo0558 protein OS=Listeria monocytogenes serovar 1/2a (strain ATCC BAA-679 / EGD-e) OX=169963 GN=lmo0558 PE=4 SV=1

Query	Observed	Mr(expt)	Mr(calc)	ppm	Miss	Score	Expect	Rank	Unique	Peptide
2038	449.744	897.474	897.471	4.00	0	27	0.0054	1	U	R.FLYVSNR.G
2358	462.233	922.451	922.451	0.25	0	26	0.0069	1	U	K.AESQGIYR.L
4214	537.314	1072.614	1072.613	0.67	0	46	8.8e-05	1	U	K.ETGALTLLQK.D

---

111. sp|Q8Y7Q1 **Mass:** 88184 **Score:** 64 **Matches:** 2(2) **Sequences:** 2(2) **emPAI:** 0.10

Phenylalanine--tRNA ligase beta subunit OS=Listeria monocytogenes serovar 1/2a (strain ATCC BAA-679 / EGD-e) OX=169963 GN=pheT PE=3 SV=1

Query	Observed	Mr(expt)	Mr(calc)	ppm	Miss	Score	Expect	Rank	Unique	Peptide
1775	441.229	880.444	880.440	3.95	0	53	1.8e-05	1	U	R.SASYNIAR.K
3569	513.819	1025.623	1025.623	-0.36	0	27	0.002	1	U	R.TSIVPQLIR.S

---

112. tr|Q8Y7E4 **Mass:** 33862 **Score:** 64 **Matches:** 1(1) **Sequences:** 1(1) **emPAI:** 0.13

Lmo1339 protein OS=Listeria monocytogenes serovar 1/2a (strain ATCC BAA-679 / EGD-e) OX=169963 GN=lmo1339 PE=4 SV=1

Query	Observed	Mr(expt)	Mr(calc)	ppm	Miss	Score	Expect	Rank	Unique	Peptide
10391	792.428	1582.841	1582.831	5.81	0	64	2.7e-06	1	U	K.IILDNDANVAALGER.W

---

113. sp|Q8Y443 **Mass:** 11172 **Score:** 64 **Matches:** 3(2) **Sequences:** 2(2) **emPAI:** 1.08

50S ribosomal protein L24 OS=Listeria monocytogenes serovar 1/2a (strain ATCC BAA-679 / EGD-e) OX=169963 GN=rplX PE=3 SV=1

Query	Observed	Mr(expt)	Mr(calc)	ppm	Miss	Score	Expect	Rank	Unique	Peptide
337	373.232	744.449	744.453	-5.58	0	36	0.0011	1	U	K.VLAAFPK.K
4978	566.326	1130.637	1130.637	0.01	0	46	8.1e-05	1	U	R.VLIEGINMVK.K 4977

114. tr|Q8Y685 **Mass:** 31123 **Score:** 62 **Matches:** 2(2) **Sequences:** 2(2) **emPAI:** 0.31
Lmo1812 protein OS=Listeria monocytogenes serovar 1/2a (strain ATCC BAA-679 / EGD-e) OX=169963 GN=lmo1812 PE=4 SV=1

Query	Observed	Mr(expt)	Mr(calc)	ppm	Miss	Score	Expect	Rank	Unique	Peptide
4097	533.253	1064.491	1064.481	9.32	0	24	0.013	1	U	R.EEITAAMER.N
7645	665.832	1329.649	1329.652	-2.51	0	56	9e-06	1	U	R.ETAEGGLAATPTGR.A

115. tr|Q8YAV6 **Mass:** 94684 **Score:** 62 **Matches:** 1(1) **Sequences:** 1(1) **emPAI:** 0.05
DNA gyrase subunit A OS=Listeria monocytogenes serovar 1/2a (strain ATCC BAA-679 / EGD-e) OX=169963 GN=gyrA PE=3 SV=1

Query	Observed	Mr(expt)	Mr(calc)	ppm	Miss	Score	Expect	Rank	Unique	Peptide
5800	599.856	1197.697	1197.697	-0.29	0	62	1.3e-06	1	U	R.IALDNIDAIIK.L

116. tr|Q8Y7B2 **Mass:** 45197 **Score:** 59 **Matches:** 2(2) **Sequences:** 2(2) **emPAI:** 0.20
Dihydrolipoamide acetyltransferase component of pyruvate dehydrogenase complex OS=Listeria monocytogenes serovar 1/2a (strain ATCC BAA-679 / EGD-e)

Query	Observed	Mr(expt)	Mr(calc)	ppm	Miss	Score	Expect	Rank	Unique	Peptide
1821	442.236	882.458	882.456	2.71	0	32	0.0055	1	U	K.EATPNPVR.S
9355	746.924	1491.834	1491.830	2.93	0	43	6.5e-05	1	U	K.DLLQVIENGPVAPK.R

117. sp|Q9NSB2 **Mass:** 65942 **Score:** 59 **Matches:** 2(2) **Sequences:** 2(2) **emPAI:** 0.14
Keratin, type II cuticular Hb4 (Contact-Cont) OS=Homo sapiens GN=KRT84 PE=2 SV=2

Query	Observed	Mr(expt)	Mr(calc)	ppm	Miss	Score	Expect	Rank	Unique	Peptide
1020	414.219	826.423	826.423	0.23	0	40	0.00024	1		K.FASFIDK.V
2138	453.737	905.459	905.461	-1.81	0	37	0.0009	1	U	R.FLEQQNK.L

118. tr|Q8Y6W5 **Mass:** 46290 **Score:** 57 **Matches:** 4(4) **Sequences:** 4(4) **emPAI:** 0.44
Isocitrate dehydrogenase [NADP] OS=Listeria monocytogenes serovar 1/2a (strain ATCC BAA-679 / EGD-e) OX=169963 GN=citC PE=4 SV=1

Query	Observed	Mr(expt)	Mr(calc)	ppm	Miss	Score	Expect	Rank	Unique	Peptide
725	399.241	796.467	796.481	-16.67	0	28	0.0017	1	U	K.SLTLVHK.G
4787	560.265	1118.516	1118.509	5.85	0	29	0.0026	1	U	K.EAEGTEVADAK.Q
5069	569.831	1137.647	1137.651	-3.05	0	25	0.0031	1	U	K.GPLTTPIGGGIR.S
9170	491.947	1472.819	1472.824	-3.45	0	18	0.037	1	U	R.FPETSGIGIKPISK.E

119. tr|Q8Y5M5 **Mass:** 41325 **Score:** 57 **Matches:** 2(2) **Sequences:** 2(2) **emPAI:** 0.23
Cell division protein FtsZ OS=Listeria monocytogenes serovar 1/2a (strain ATCC BAA-679 / EGD-e) OX=169963 GN=ftsZ PE=3 SV=1

Query	Observed	Mr(expt)	Mr(calc)	ppm	Miss	Score	Expect	Rank	Unique	Peptide
2788	480.252	958.490	958.497	-7.22	0	20	0.033	1	U	R.EQIEEALK.G
8470	703.362	2107.065	2107.077	-5.63	0	54	3.2e-05	1	U	K.EMGALTVGVVTRPFGFEGPK.R

120. tr|Q8Y4G8 **Mass:** 64188 **Score:** 57 **Matches:** 2(2) **Sequences:** 2(2) **emPAI:** 0.14
Lmo2475 protein OS=Listeria monocytogenes serovar 1/2a (strain ATCC BAA-679 / EGD-e) OX=169963 GN=lmo2475 PE=3 SV=1

Query	Observed	Mr(expt)	Mr(calc)	ppm	Miss	Score	Expect	Rank	Unique	Peptide
10138	783.389	1564.764	1564.756	5.45	0	28	0.0048	1	U	R.EQLPTSMGGFVVER.A
12286	855.409	1708.803	1708.794	4.84	0	46	7e-05	1	U	K.VDEQFVNDPDFGTVK.S

121. sp|P02663 **Mass:** 26173 **Score:** 56 **Matches:** 2(2) **Sequences:** 2(2) **emPAI:** 0.38
Alpha-S2-casein (Laboratory-Cont) OS=Bos taurus GN=CSN1S2 PE=1 SV=2

Query	Observed	Mr(expt)	Mr(calc)	ppm	Miss	Score	Expect	Rank	Unique	Peptide
3025	490.283	978.551	978.554	-3.13	0	43	0.00011	1	U	K.FALPQYLK.T
8077	684.349	1366.683	1366.688	-4.05	0	31	0.003	1	U	K.ALNEINQFYQK.F

122. tr|Q8Y6J6 **Mass:** 27226 **Score:** 55 **Matches:** 2(2) **Sequences:** 2(2) **emPAI:** 0.36
Lmo1688 protein OS=Listeria monocytogenes serovar 1/2a (strain ATCC BAA-679 / EGD-e) OX=169963 GN=lmo1688 PE=4 SV=1

Query	Observed	Mr(expt)	Mr(calc)	ppm	Miss	Score	Expect	Rank	Unique	Peptide
1900	445.250	888.485	888.503	-19.98	0	36	0.0013	1	U	K.VALVTGSSR.G
5142	573.349	1144.683	1144.682	1.61	0	34	0.00044	1	U	K.IISLSSIGSIR.Y

123. sp|Q8Y4C4 **Mass:** 46007 **Score:** 55 **Matches:** 2(2) **Sequences:** 2(2) **emPAI:** 0.20
UDP-N-acetylglucosamine 1-carboxyvinyltransferase 1 OS=Listeria monocytogenes serovar 1/2a (strain ATCC BAA-679 / EGD-e) OX=169963 GN=murA1 PE=1 S

Query	Observed	Mr(expt)	Mr(calc)	ppm	Miss	Score	Expect	Rank	Unique	Peptide
4179	536.296	1070.577	1070.572	4.86	0	42	0.00022	1	U	K.LQALGADVER.V
9243	740.957	1479.900	1479.902	-1.90	0	28	0.0014	1	U	K.NAVLPVIAATLLASK.G

124. sp|P0DJM1 **Mass:** 41508 **Score:** 53 **Matches:** 2(2) **Sequences:** 2(2) **emPAI:** 0.22
Chaperone protein DnaJ OS=Listeria monocytogenes serovar 1/2a (strain ATCC BAA-679 / EGD-e) OX=169963 GN=dnaJ PE=3 SV=1

Query	Observed	Mr(expt)	Mr(calc)	ppm	Miss	Score	Expect	Rank	Unique	Peptide
6987	644.312	1286.609	1286.604	4.25	0	49	3e-05	1	U	K.VPAGVNDGQQMR.V
8616	709.856	1417.698	1417.695	2.03	0	21	0.031	1	U	K.GSINVEQNTPFGR.V

125. tr|Q8Y770 **Mass:** 60432 **Score:** 52 **Matches:** 1(1) **Sequences:** 1(1) **emPAI:** 0.07
Lmo1431 protein OS=Listeria monocytogenes serovar 1/2a (strain ATCC BAA-679 / EGD-e) OX=169963 GN=lmo1431 PE=4 SV=1

Query	Observed	Mr(expt)	Mr(calc)	ppm	Miss	Score	Expect	Rank	Unique	Peptide
7062	646.358	1290.701	1290.693	5.79	0	52	3.3e-05	1	U	K.ILDNLSFSINR.N

126. sp|Q8Y699 **Mass:** 10359 **Score:** 51 **Matches:** 1(1) **Sequences:** 1(1) **emPAI:** 0.49
30S ribosomal protein S16 OS=Listeria monocytogenes serovar 1/2a (strain ATCC BAA-679 / EGD-e) OX=169963 GN=rpsP PE=3 SV=1

Query	Observed	Mr(expt)	Mr(calc)	ppm	Miss	Score	Expect	Rank	Unique	Peptide
426	380.223	758.431	758.429	3.64	0	51	4.6e-05	1	U	R.IVVADSR.F

---

127. sp|Q8Y5W9 **Mass:** 36702 **Score:** 51 **Matches:** 2(1) **Sequences:** 2(1) **emPAI:** 0.12
Glycerol-3-phosphate dehydrogenase [NAD(P)+] OS=Listeria monocytogenes serovar 1/2a (strain ATCC BAA-679 / EGD-e) OX=169963 GN=gpsA PE=3 SV=1

Query	Observed	Mr(expt)	Mr(calc)	ppm	Miss	Score	Expect	Rank	Unique	Peptide
3591	514.772	1027.530	1027.530	0.05	0	17	0.092	1	U	K.GIEPETNLR.M
9219	740.346	1478.677	1478.681	-2.96	0	51	3.3e-05	1	U	R.MSEVIEEEIDAAK.R

---

128. tr|Q8Y436 **Mass:** 32778 **Score:** 49 **Matches:** 1(1) **Sequences:** 1(1) **emPAI:** 0.14
Lmo2637 protein OS=Listeria monocytogenes serovar 1/2a (strain ATCC BAA-679 / EGD-e) OX=169963 GN=lmo2637 PE=4 SV=1

Query	Observed	Mr(expt)	Mr(calc)	ppm	Miss	Score	Expect	Rank	Unique	Peptide
8020	681.355	1360.696	1360.720	-17.61	0	49	5.1e-05	1	U	K.ADTTTISINNLAK.M

---

129. sp|Q8Y688 **Mass:** 36498 **Score:** 48 **Matches:** 1(1) **Sequences:** 1(1) **emPAI:** 0.12
Phosphate acyltransferase OS=Listeria monocytogenes serovar 1/2a (strain ATCC BAA-679 / EGD-e) OX=169963 GN=plsX PE=3 SV=1

Query	Observed	Mr(expt)	Mr(calc)	ppm	Miss	Score	Expect	Rank	Unique	Peptide
17581	795.394	2383.160	2383.165	-2.31	0	48	2.8e-05	1	U	R.DLLMDVADVVVTDGFTGNMVLK.S

---

130. tr|Q8Y5V7 **Mass:** 27493 **Score:** 48 **Matches:** 5(4) **Sequences:** 2(2) **emPAI:** 0.36
ResD protein OS=Listeria monocytogenes serovar 1/2a (strain ATCC BAA-679 / EGD-e) OX=169963 GN=resD PE=4 SV=1

Query	Observed	Mr(expt)	Mr(calc)	ppm	Miss	Score	Expect	Rank	Unique	Peptide
961	411.224	820.433	820.444	-13.61	1	23	0.019	1	U	R.ELREFK.S 958 960 962
8921	724.357	2170.049	2170.054	-2.23	0	35	0.0027	1	U	K.QSSEESAGGTPGDIITFPHLK.I

---

131. tr|Q8Y782 **Mass:** 41165 **Score:** 48 **Matches:** 1(1) **Sequences:** 1(1) **emPAI:** 0.11
Lmo1414 protein OS=Listeria monocytogenes serovar 1/2a (strain ATCC BAA-679 / EGD-e) OX=169963 GN=lmo1414 PE=3 SV=1

Query	Observed	Mr(expt)	Mr(calc)	ppm	Miss	Score	Expect	Rank	Unique	Peptide
6794	637.354	1272.694	1272.693	0.85	0	48	5.3e-05	1	U	K.DISAVDLGATALK.G

---

132. tr|Q8Y4U2 **Mass:** 32682 **Score:** 47 **Matches:** 2(2) **Sequences:** 2(2) **emPAI:** 0.29
Pseudouridine-5'-phosphate glycosidase OS=Listeria monocytogenes serovar 1/2a (strain ATCC BAA-679 / EGD-e) OX=169963 GN=psuG PE=3 SV=1

Query	Observed	Mr(expt)	Mr(calc)	ppm	Miss	Score	Expect	Rank	Unique	Peptide
1666	436.747	871.480	871.476	4.33	0	32	0.0021	1	U	R.DVEQIIR.D
6013	607.362	1212.709	1212.708	0.91	0	32	0.0015	1	U	K.VINDVIQTALK.E

---

133. tr|Q8Y701 **Mass:** 12007 **Score:** 47 **Matches:** 1(1) **Sequences:** 1(1) **emPAI:** 0.41
Lmo1529 protein OS=Listeria monocytogenes serovar 1/2a (strain ATCC BAA-679 / EGD-e) OX=169963 GN=lmo1529 PE=4 SV=1

Query	Observed	Mr(expt)	Mr(calc)	ppm	Miss	Score	Expect	Rank	Unique	Peptide
6497	625.805	1249.595	1249.597	-1.92	0	47	7.2e-05	1	U	K.EVQNMQSSLAK.G

---

134. sp|P66401 **Mass:** 7370 **Score:** 47 **Matches:** 1(1) **Sequences:** 1(1) **emPAI:** 0.73
30S ribosomal protein S14 type Z OS=Listeria monocytogenes serovar 1/2a (strain ATCC BAA-679 / EGD-e) OX=169963 GN=rpsZ PE=3 SV=1

Query	Observed	Mr(expt)	Mr(calc)	ppm	Miss	Score	Expect	Rank	Unique	Peptide
2920	486.260	970.506	970.487	19.8	0	47	0.00015	1	U	K.YAVQAYTR.C

---

135. sp|Q8Y8K6 **Mass:** 17487 **Score:** 46 **Matches:** 1(1) **Sequences:** 1(1) **emPAI:** 0.27
Serine-protein kinase RsbW OS=Listeria monocytogenes serovar 1/2a (strain ATCC BAA-679 / EGD-e) OX=169963 GN=rsbW PE=3 SV=1

Query	Observed	Mr(expt)	Mr(calc)	ppm	Miss	Score	Expect	Rank	Unique	Peptide
2100	452.269	902.524	902.519	6.05	0	46	8e-05	1	U	R.LSLSGIASR.A

---

136. sp|Q8Y6X8 **Mass:** 46688 **Score:** 45 **Matches:** 2(2) **Sequences:** 2(2) **emPAI:** 0.20
Glutamate-1-semialdehyde 2,1-aminomutase 1 OS=Listeria monocytogenes serovar 1/2a (strain ATCC BAA-679 / EGD-e) OX=169963 GN=hemL1 PE=3 SV=1

Query	Observed	Mr(expt)	Mr(calc)	ppm	Miss	Score	Expect	Rank	Unique	Peptide
2222	456.780	911.545	911.544	0.55	0	33	0.00054	1	U	R.VPSIEIVR.M
11796	840.926	1679.838	1679.825	7.74	0	29	0.0064	1	U	K.GTSFGTPTEIETELAK.L

---

137. sp|Q8Y445 **Mass:** 13088 **Score:** 45 **Matches:** 1(1) **Sequences:** 1(1) **emPAI:** 0.37
50S ribosomal protein L18 OS=Listeria monocytogenes serovar 1/2a (strain ATCC BAA-679 / EGD-e) OX=169963 GN=rplR PE=3 SV=1

Query	Observed	Mr(expt)	Mr(calc)	ppm	Miss	Score	Expect	Rank	Unique	Peptide
159	358.219	714.424	714.428	-4.78	0	45	0.00018	1	U	K.VGELVAK.R

---

138. sp|Q8Y7C5 **Mass:** 31002 **Score:** 45 **Matches:** 1(1) **Sequences:** 1(1) **emPAI:** 0.15
Bifunctional protein FolD OS=Listeria monocytogenes serovar 1/2a (strain ATCC BAA-679 / EGD-e) OX=169963 GN=folD PE=3 SV=1

Query	Observed	Mr(expt)	Mr(calc)	ppm	Miss	Score	Expect	Rank	Unique	Peptide
10573	800.419	1598.824	1598.840	-10.47	0	45	9.9e-05	1	U	K.SVLIELPENVTEEK.L

---

139. sp|P60384 **Mass:** 24185 **Score:** 44 **Matches:** 2(1) **Sequences:** 2(1) **emPAI:** 0.19
Redox-sensing transcriptional repressor Rex OS=Listeria monocytogenes serovar 1/2a (strain ATCC BAA-679 / EGD-e) OX=169963 GN=rex PE=3 SV=1

Query	Observed	Mr(expt)	Mr(calc)	ppm	Miss	Score	Expect	Rank	Unique	Peptide
3132	494.783	987.552	987.550	1.45	0	18	0.064	1	U	K.GILNFTPAR.I
5140	573.313	1144.612	1144.613	-0.33	0	44	0.00017	1	U	K.IVAAFDVDPAK.V

---

140. sp|Q8Y6U0 **Mass:** 45116 **Score:** 43 **Matches:** 1(1) **Sequences:** 1(1) **emPAI:** 0.10
Probable tRNA sulfurtransferase OS=Listeria monocytogenes serovar 1/2a (strain ATCC BAA-679 / EGD-e) OX=169963 GN=thiI PE=3 SV=1

Query	Observed	Mr(expt)	Mr(calc)	ppm	Miss	Score	Expect	Rank	Unique	Peptide
14363	628.975	1883.904	1883.938	-17.83	0	43	0.00013	1	U	K.AAALALVQDAHEENGTFK.V

141. sp|Q8Y447 **Mass:** 15772 **Score:** 43 **Matches:** 2(2) **Sequences:** 2(2) **emPAI:** 0.69
50S ribosomal protein L15 OS=Listeria monocytogenes serovar 1/2a (strain ATCC BAA-679 / EGD-e) OX=169963 GN=rplO PE=3 SV=1

Query	Observed	Mr(expt)	Mr(calc)	ppm	Miss	Score	Expect	Rank	Unique	Peptide
951	411.220	820.426	820.419	8.63	0	27	0.01	1	U	R.GFTNINR.K
15999	1059.552	2117.089	2117.089	-0.09	0	31	0.0023	1	U	R.FEDGTEVTPELLVETGIIR.N

142. sp|P66484 **Mass:** 10468 **Score:** 43 **Matches:** 1(1) **Sequences:** 1(1) **emPAI:** 0.48
30S ribosomal protein S19 OS=Listeria monocytogenes serovar 1/2a (strain ATCC BAA-679 / EGD-e) OX=169963 GN=rpsS PE=3 SV=1

Query	Observed	Mr(expt)	Mr(calc)	ppm	Miss	Score	Expect	Rank	Unique	Peptide
15211	986.516	1971.017	1971.010	3.27	0	43	0.00015	1	U	R.STIFPTFVGQTIAVYDGR.K

143. tr|Q8Y6B8 **Mass:** 49225 **Score:** 42 **Matches:** 1(1) **Sequences:** 1(1) **emPAI:** 0.09
Adenylosuccinate lyase OS=Listeria monocytogenes serovar 1/2a (strain ATCC BAA-679 / EGD-e) OX=169963 GN=purB PE=3 SV=1

Query	Observed	Mr(expt)	Mr(calc)	ppm	Miss	Score	Expect	Rank	Unique	Peptide
8022	681.370	1360.726	1360.724	1.59	0	42	0.00022	1	U	K.DLENFIAIIGEK.A

144. sp|Q8Y3T8 **Mass:** 23138 **Score:** 42 **Matches:** 1(1) **Sequences:** 1(1) **emPAI:** 0.20
Probable transaldolase 1 OS=Listeria monocytogenes serovar 1/2a (strain ATCC BAA-679 / EGD-e) OX=169963 GN=tal1 PE=3 SV=1

Query	Observed	Mr(expt)	Mr(calc)	ppm	Miss	Score	Expect	Rank	Unique	Peptide
4446	545.305	1088.595	1088.587	7.95	0	42	0.00047	1	U	K.AGADIATVPFK.V

145. sp|P66623 **Mass:** 14635 **Score:** 42 **Matches:** 1(1) **Sequences:** 1(1) **emPAI:** 0.33
30S ribosomal protein S8 OS=Listeria monocytogenes serovar 1/2a (strain ATCC BAA-679 / EGD-e) OX=169963 GN=rpsH PE=3 SV=1

Query	Observed	Mr(expt)	Mr(calc)	ppm	Miss	Score	Expect	Rank	Unique	Peptide
10656	805.373	1608.731	1608.727	2.73	0	42	0.00023	1	U	R.DVEYIEDDNAGTIR.V

146. sp|Q8Y8C0 **Mass:** 59476 **Score:** 38 **Matches:** 1(1) **Sequences:** 1(1) **emPAI:** 0.07
Peptide chain release factor 3 OS=Listeria monocytogenes serovar 1/2a (strain ATCC BAA-679 / EGD-e) OX=169963 GN=prfC PE=3 SV=1

Query	Observed	Mr(expt)	Mr(calc)	ppm	Miss	Score	Expect	Rank	Unique	Peptide
15801	693.995	2078.963	2078.955	4.28	0	38	0.0002	1	U	R.INILDTPGHSDFSEDTYR.T

147. tr|Q8Y764 **Mass:** 37690 **Score:** 38 **Matches:** 1(1) **Sequences:** 1(1) **emPAI:** 0.12
Aspartate-semialdehyde dehydrogenase OS=Listeria monocytogenes serovar 1/2a (strain ATCC BAA-679 / EGD-e) OX=169963 GN=asd PE=3 SV=1

Query	Observed	Mr(expt)	Mr(calc)	ppm	Miss	Score	Expect	Rank	Unique	Peptide
16156	717.407	2149.200	2149.163	17.1	0	38	0.00015	1	U	R.IIVSTYQAVSGSGVSAIQELK.D

148. sp|P28764 **Mass:** 22617 **Score:** 38 **Matches:** 1(1) **Sequences:** 1(1) **emPAI:** 0.20
Superoxide dismutase [Mn] OS=Listeria monocytogenes serovar 1/2a (strain ATCC BAA-679 / EGD-e) OX=169963 GN=sodA PE=3 SV=1

Query	Observed	Mr(expt)	Mr(calc)	ppm	Miss	Score	Expect	Rank	Unique	Peptide
694	396.215	790.416	790.409	9.58	0	38	0.00073	1	U	K.FNAAAAAR.F

149. sp|P58695 **Mass:** 49082 **Score:** 37 **Matches:** 3(3) **Sequences:** 3(3) **emPAI:** 0.29
Asparagine--tRNA ligase OS=Listeria monocytogenes serovar 1/2a (strain ATCC BAA-679 / EGD-e) OX=169963 GN=asnS PE=3 SV=1

Query	Observed	Mr(expt)	Mr(calc)	ppm	Miss	Score	Expect	Rank	Unique	Peptide
3328	503.240	1004.466	1004.460	5.74	0	31	0.0021	1	U	R.ASYEFFNK.E
3973	529.276	1056.538	1056.539	-1.10	0	18	0.042	1	U	K.VFSFGPTFR.A
7276	653.845	1305.675	1305.693	-13.47	0	22	0.02	1	U	K.ITINQASEFVGK.E

150. tr|Q8Y8V1 **Mass:** 24025 **Score:** 36 **Matches:** 1(1) **Sequences:** 1(1) **emPAI:** 0.19
Lmo0791 protein OS=Listeria monocytogenes serovar 1/2a (strain ATCC BAA-679 / EGD-e) OX=169963 GN=lmo0791 PE=4 SV=1

Query	Observed	Mr(expt)	Mr(calc)	ppm	Miss	Score	Expect	Rank	Unique	Peptide
7839	673.357	1344.700	1344.714	-10.40	0	36	0.0018	1	U	K.GVQTELVEELTK.A

151. tr|Q8Y7B3 **Mass:** 35828 **Score:** 34 **Matches:** 1(1) **Sequences:** 1(1) **emPAI:** 0.12
Lmo1373 protein OS=Listeria monocytogenes serovar 1/2a (strain ATCC BAA-679 / EGD-e) OX=169963 GN=lmo1373 PE=4 SV=1

Query	Observed	Mr(expt)	Mr(calc)	ppm	Miss	Score	Expect	Rank	Unique	Peptide
3618	515.797	1029.579	1029.571	8.11	0	34	0.0016	1	U	K.VLLVTEDNK.Q

152. tr|Q8Y835 **Mass:** 37050 **Score:** 33 **Matches:** 1(1) **Sequences:** 1(1) **emPAI:** 0.12
dTDP-glucose 4,6-dehydratase OS=Listeria monocytogenes serovar 1/2a (strain ATCC BAA-679 / EGD-e) OX=169963 GN=lmo1083 PE=3 SV=1

Query	Observed	Mr(expt)	Mr(calc)	ppm	Miss	Score	Expect	Rank	Unique	Peptide
14952	646.353	1936.039	1936.027	6.21	0	33	0.00085	1	U	R.TNNEIVHIIVDDLNLSK.D

153. tr|Q8Y846 **Mass:** 128425 **Score:** 33 **Matches:** 2(2) **Sequences:** 2(2) **emPAI:** 0.07
Pyruvate carboxylase OS=Listeria monocytogenes serovar 1/2a (strain ATCC BAA-679 / EGD-e) OX=169963 GN=pycA PE=4 SV=1

Query	Observed	Mr(expt)	Mr(calc)	ppm	Miss	Score	Expect	Rank	Unique	Peptide
6969	643.377	1284.740	1284.755	-12.15	0	19	0.026	1	U	K.TNIPFLLNVVR.H
7773	671.334	1340.653	1340.625	21.0	0	30	0.0022	1	U	K.AAFGNDEVYVEK.C

154. sp|Q9AGE7 **Mass:** 10058 **Score:** 32 **Matches:** 1(1) **Sequences:** 1(1) **emPAI:** 0.50

10 kDa chaperonin OS=Listeria monocytogenes serovar 1/2a (strain ATCC BAA-679 / EGD-e) OX=169963 GN=groS PE=3 SV=2

Query	Observed	Mr(expt)	Mr(calc)	ppm	Miss	Score	Expect	Rank	Unique	Peptide
5314	579.822	1157.629	1157.629	0.15	0	32	0.0024	1	U	K.TASGIVLPDSAK.E

---

155. tr|Q8Y7C7 **Mass:** 50297 **Score:** 31 **Matches:** 2(2) **Sequences:** 2(2) **emPAI:** 0.18

Acetyl-CoA carboxylase subunit (Biotin carboxylase subunit) OS=Listeria monocytogenes serovar 1/2a (strain ATCC BAA-679 / EGD-e) OX=169963 GN=lmo13

Query	Observed	Mr(expt)	Mr(calc)	ppm	Miss	Score	Expect	Rank	Unique	Peptide
2246	457.731	913.447	913.450	-4.34	0	22	0.025	1	U	R.INAENPEK.N
15831	695.376	2083.105	2083.110	-2.29	0	27	0.0048	1	U	R.ALSEFAIDGIPSTIPFHLR.V

---

156. sp|P0DJP1 **Mass:** 6842 **Score:** 31 **Matches:** 1(1) **Sequences:** 1(1) **emPAI:** 0.79

30S ribosomal protein S21 OS=Listeria monocytogenes serovar 1/2a (strain ATCC BAA-679 / EGD-e) OX=169963 GN=rpsU PE=3 SV=1

Query	Observed	Mr(expt)	Mr(calc)	ppm	Miss	Score	Expect	Rank	Unique	Peptide
4899	563.789	1125.564	1125.571	-6.23	0	31	0.0019	1	U	R.EFYEKPSVK.R

---

157. sp|Q8Y722 **Mass:** 98030 **Score:** 31 **Matches:** 2(2) **Sequences:** 2(2) **emPAI:** 0.09

Alanine--tRNA ligase OS=Listeria monocytogenes serovar 1/2a (strain ATCC BAA-679 / EGD-e) OX=169963 GN=alaS PE=3 SV=1

Query	Observed	Mr(expt)	Mr(calc)	ppm	Miss	Score	Expect	Rank	Unique	Peptide
3977	529.288	1056.562	1056.564	-2.02	0	29	0.0092	1	U	R.QLFLDFFK.E
8199	690.341	1378.667	1378.652	10.8	0	18	0.043	1	U	K.YFDGSVIPDNPR.M

---

158. tr|Q8Y791 **Mass:** 29937 **Score:** 31 **Matches:** 1(1) **Sequences:** 1(1) **emPAI:** 0.15

Lmo1401 protein OS=Listeria monocytogenes serovar 1/2a (strain ATCC BAA-679 / EGD-e) OX=169963 GN=lmo1401 PE=4 SV=1

Query	Observed	Mr(expt)	Mr(calc)	ppm	Miss	Score	Expect	Rank	Unique	Peptide
13610	456.238	1820.921	1820.913	4.66	0	31	0.0029	1	U	R.VTAVVGTHTHVQTSDNR.I

---

159. tr|Q8Y7J1 **Mass:** 73026 **Score:** 31 **Matches:** 1(1) **Sequences:** 1(1) **emPAI:** 0.06

DNA topoisomerase 4 subunit B OS=Listeria monocytogenes serovar 1/2a (strain ATCC BAA-679 / EGD-e) OX=169963 GN=parE PE=3 SV=1

Query	Observed	Mr(expt)	Mr(calc)	ppm	Miss	Score	Expect	Rank	Unique	Peptide
2773	479.294	956.573	956.581	-7.62	0	31	0.0031	1	U	R.FQAILPLR.G

---

160. sp|Q8Y6V0 **Mass:** 43879 **Score:** 29 **Matches:** 2(1) **Sequences:** 2(1) **emPAI:** 0.10

Acetate kinase 1 OS=Listeria monocytogenes serovar 1/2a (strain ATCC BAA-679 / EGD-e) OX=169963 GN=ackA1 PE=3 SV=1

Query	Observed	Mr(expt)	Mr(calc)	ppm	Miss	Score	Expect	Rank	Unique	Peptide
34	352.694	703.373	703.375	-2.68	0	18	0.1	2	U	R.DVETIK.N
5350	581.324	1160.633	1160.640	-6.18	0	29	0.0055	1	U	K.TIAINAGSSSLK.F

---

161. tr|Q929C7 **Mass:** 24060 **Score:** 29 **Matches:** 1(1) **Sequences:** 1(1) **emPAI:** 0.19

Lmo2248 protein OS=Listeria monocytogenes serovar 1/2a (strain ATCC BAA-679 / EGD-e) OX=169963 GN=lmo2248 PE=4 SV=1

Query	Observed	Mr(expt)	Mr(calc)	ppm	Miss	Score	Expect	Rank	Unique	Peptide
10310	788.921	1575.827	1575.851	-15.03	0	29	0.0042	1	U	K.FIQAIQASTVEIEK.A

---

162. sp|Q8Y5X5 **Mass:** 42260 **Score:** 28 **Matches:** 1(1) **Sequences:** 1(1) **emPAI:** 0.10

Chorismate synthase OS=Listeria monocytogenes serovar 1/2a (strain ATCC BAA-679 / EGD-e) OX=169963 GN=aroC PE=3 SV=1

Query	Observed	Mr(expt)	Mr(calc)	ppm	Miss	Score	Expect	Rank	Unique	Peptide
2823	481.782	961.549	961.560	-10.75	0	28	0.0045	1	U	R.AIVSINAFK.G

---

163. tr|Q8Y838 **Mass:** 71500 **Score:** 28 **Matches:** 2(2) **Sequences:** 2(2) **emPAI:** 0.13

Lmo1080 protein OS=Listeria monocytogenes serovar 1/2a (strain ATCC BAA-679 / EGD-e) OX=169963 GN=lmo1080 PE=4 SV=1

Query	Observed	Mr(expt)	Mr(calc)	ppm	Miss	Score	Expect	Rank	Unique	Peptide
893	408.231	814.447	814.438	11.9	0	20	0.058	1	U	K.LFNFFK.D
1497	429.749	857.483	857.486	-3.20	0	28	0.0081	1	U	R.EQILIDK.T

---

164. tr|Q8Y781 **Mass:** 43445 **Score:** 27 **Matches:** 1(1) **Sequences:** 1(1) **emPAI:** 0.10

Lmo1415 protein OS=Listeria monocytogenes serovar 1/2a (strain ATCC BAA-679 / EGD-e) OX=169963 GN=lmo1415 PE=4 SV=1

Query	Observed	Mr(expt)	Mr(calc)	ppm	Miss	Score	Expect	Rank	Unique	Peptide
12533	865.443	1728.872	1728.878	-3.80	0	27	0.005	1	U	K.ALDTIIETAPSDVQEK.L

---

165. tr|Q8Y5Q0 **Mass:** 61673 **Score:** 27 **Matches:** 1(1) **Sequences:** 1(1) **emPAI:** 0.07

AlsS protein OS=Listeria monocytogenes serovar 1/2a (strain ATCC BAA-679 / EGD-e) OX=169963 GN=alsS PE=3 SV=1

Query	Observed	Mr(expt)	Mr(calc)	ppm	Miss	Score	Expect	Rank	Unique	Peptide
18385	832.121	2493.341	2493.323	7.26	0	27	0.0022	1	U	K.SGADLVVDSLINQGVTHVFGIPGAK.I

---

166. sp|P13128 **Mass:** 58709 **Score:** 27 **Matches:** 1(1) **Sequences:** 1(1) **emPAI:** 0.07

Listeriolysin O OS=Listeria monocytogenes serovar 1/2a (strain ATCC BAA-679 / EGD-e) OX=169963 GN=hly PE=1 SV=1

Query	Observed	Mr(expt)	Mr(calc)	ppm	Miss	Score	Expect	Rank	Unique	Peptide
8722	715.370	1428.725	1428.732	-5.11	0	27	0.016	1	U	K.SNVNNAVNTLVER.W

---

167. sp|Q8Y624 **Mass:** 60333 **Score:** 27 **Matches:** 1(1) **Sequences:** 1(1) **emPAI:** 0.07

Formate--tetrahydrofolate ligase OS=Listeria monocytogenes serovar 1/2a (strain ATCC BAA-679 / EGD-e) OX=169963 GN=fhs PE=3 SV=2

Query	Observed	Mr(expt)	Mr(calc)	ppm	Miss	Score	Expect	Rank	Unique	Peptide
466	382.220	762.425	762.428	-3.93	0	27	0.0098	1	U	K.QIVEFK.K

---

168. sp|Q8Y9C1 **Mass:** 23011 **Score:** 27 **Matches:** 1(1) **Sequences:** 1(1) **emPAI:** 0.20

FMN-dependent NADH-azoreductase 1 OS=Listeria monocytogenes serovar 1/2a (strain ATCC BAA-679 / EGD-e) OX=169963 GN=azoR1 PE=3 SV=1

Query	Observed	Mr(expt)	Mr(calc)	ppm	Miss	Score	Expect	Rank	Unique	Peptide
1022	414.226	826.437	826.430	8.86	0	27	0.0052	1	U	K.ANGLPAER.S

169. sp|Q8Y723 **Mass:** 15396 **Score:** 26 **Matches:** 1(1) **Sequences:** 1(1) **emPAI:** 0.31
Putative pre-16S rRNA nuclease OS=Listeria monocytogenes serovar 1/2a (strain ATCC BAA-679 / EGD-e) OX=169963 GN=lmo1502 PE=3 SV=1

Query	Observed	Mr(expt)	Mr(calc)	ppm	Miss	Score	Expect	Rank	Unique	Peptide
3300	502.281	1002.548	1002.535	13.1	0	26	0.011	1	U	R.TLIEADVSR.K

170. tr|Q8Y9D8 **Mass:** 19958 **Score:** 26 **Matches:** 1(1) **Sequences:** 1(1) **emPAI:** 0.23
Lmo0592 protein OS=Listeria monocytogenes serovar 1/2a (strain ATCC BAA-679 / EGD-e) OX=169963 GN=lmo0592 PE=4 SV=1

Query	Observed	Mr(expt)	Mr(calc)	ppm	Miss	Score	Expect	Rank	Unique	Peptide
2683	475.749	949.483	949.487	-3.67	0	26	0.01	1	U	K.DALTVFER.T

171. sp|Q8YAR1 **Mass:** 47804 **Score:** 26 **Matches:** 1(1) **Sequences:** 1(1) **emPAI:** 0.09
Adenylosuccinate synthetase OS=Listeria monocytogenes serovar 1/2a (strain ATCC BAA-679 / EGD-e) OX=169963 GN=purA PE=3 SV=1

Query	Observed	Mr(expt)	Mr(calc)	ppm	Miss	Score	Expect	Rank	Unique	Peptide
1525	431.229	860.444	860.435	10.2	0	26	0.019	1	U	K.GVDTSNLR.I

172. tr|Q8Y5F0 **Mass:** 28353 **Score:** 26 **Matches:** 1(1) **Sequences:** 1(1) **emPAI:** 0.16
Lmo2114 protein OS=Listeria monocytogenes serovar 1/2a (strain ATCC BAA-679 / EGD-e) OX=169963 GN=lmo2114 PE=4 SV=1

Query	Observed	Mr(expt)	Mr(calc)	ppm	Miss	Score	Expect	Rank	Unique	Peptide
5755	597.871	1193.728	1193.738	-8.53	0	26	0.0027	1	U	R.ENIILPLALAK.R

173. tr|Q8Y3T6 **Mass:** 63084 **Score:** 25 **Matches:** 1(1) **Sequences:** 1(1) **emPAI:** 0.07
Lmo2745 protein OS=Listeria monocytogenes serovar 1/2a (strain ATCC BAA-679 / EGD-e) OX=169963 GN=lmo2745 PE=4 SV=1

Query	Observed	Mr(expt)	Mr(calc)	ppm	Miss	Score	Expect	Rank	Unique	Peptide
1952	446.724	891.434	891.412	24.6	0	25	0.011	1	U	K.MATEVGER.G

174. tr|Q8Y765 **Mass:** 43169 **Score:** 25 **Matches:** 1(1) **Sequences:** 1(1) **emPAI:** 0.10
Aspartokinase OS=Listeria monocytogenes serovar 1/2a (strain ATCC BAA-679 / EGD-e) OX=169963 GN=lmo1436 PE=3 SV=1

Query	Observed	Mr(expt)	Mr(calc)	ppm	Miss	Score	Expect	Rank	Unique	Peptide
2108	452.742	903.469	903.466	3.47	0	25	0.015	1	U	K.ITEVDTAR.L

175. tr|Q8Y5P2 **Mass:** 100803 **Score:** 25 **Matches:** 1(1) **Sequences:** 1(1) **emPAI:** 0.04
Lmo2014 protein OS=Listeria monocytogenes serovar 1/2a (strain ATCC BAA-679 / EGD-e) OX=169963 GN=lmo2014 PE=4 SV=1

Query	Observed	Mr(expt)	Mr(calc)	ppm	Miss	Score	Expect	Rank	Unique	Peptide
23	351.692	701.370	701.371	-1.80	0	25	0.018	1	U	K.LADVER.F

176. tr|Q8YAV7 **Mass:** 72883 **Score:** 25 **Matches:** 1(1) **Sequences:** 1(1) **emPAI:** 0.06
DNA gyrase subunit B OS=Listeria monocytogenes serovar 1/2a (strain ATCC BAA-679 / EGD-e) OX=169963 GN=gyrB PE=3 SV=1

Query	Observed	Mr(expt)	Mr(calc)	ppm	Miss	Score	Expect	Rank	Unique	Peptide
2771	479.270	956.525	956.529	-4.56	0	25	0.008	1	U	R.ILANEEIR.T

177. tr|Q8Y616 **Mass:** 57320 **Score:** 25 **Matches:** 1(1) **Sequences:** 1(1) **emPAI:** 0.08
Metal-dependent carboxypeptidase OS=Listeria monocytogenes serovar 1/2a (strain ATCC BAA-679 / EGD-e) OX=169963 GN=lmo1886 PE=3 SV=1

Query	Observed	Mr(expt)	Mr(calc)	ppm	Miss	Score	Expect	Rank	Unique	Peptide
4382	543.315	1084.615	1084.613	2.36	0	25	0.0099	1	U	K.ALINGELEVK.D

178. DECOY_tr|Q8Y9L1 **Mass:** 53476 **Score:** 24 **Matches:** 1(1) **Sequences:** 1(1) **emPAI:** 0.08
Lmo0516 protein OS=Listeria monocytogenes serovar 1/2a (strain ATCC BAA-679 / EGD-e) OX=169963 GN=lmo0516 PE=4 SV=1

Query	Observed	Mr(expt)	Mr(calc)	ppm	Miss	Score	Expect	Rank	Unique	Peptide
260	366.699	731.384	731.381	3.86	0	24	0.02	1	U	K.EVSELR.H

179. tr|Q8Y993 **Mass:** 34137 **Score:** 24 **Matches:** 1(1) **Sequences:** 1(1) **emPAI:** 0.13
Lmo0640 protein OS=Listeria monocytogenes serovar 1/2a (strain ATCC BAA-679 / EGD-e) OX=169963 GN=lmo0640 PE=4 SV=1

Query	Observed	Mr(expt)	Mr(calc)	ppm	Miss	Score	Expect	Rank	Unique	Peptide
1346	424.720	847.425	847.440	-17.98	0	24	0.016	1	U	K.ASDVTLSR.E

180. DECOY_tr|Q8Y8I5 **Mass:** 56050 **Score:** 24 **Matches:** 1(1) **Sequences:** 1(1) **emPAI:** 0.08
Lmo0917 protein OS=Listeria monocytogenes serovar 1/2a (strain ATCC BAA-679 / EGD-e) OX=169963 GN=lmo0917 PE=3 SV=1

Query	Observed	Mr(expt)	Mr(calc)	ppm	Miss	Score	Expect	Rank	Unique	Peptide
1728	439.728	877.441	877.429	12.9	0	24	0.027	1	U	R.TWDLSTR.F

181. tr|Q928M6 **Mass:** 52711 **Score:** 24 **Matches:** 1(1) **Sequences:** 1(1) **emPAI:** 0.08
Lmo2411 protein OS=Listeria monocytogenes serovar 1/2a (strain ATCC BAA-679 / EGD-e) OX=169963 GN=lmo2411 PE=4 SV=1

Query	Observed	Mr(expt)	Mr(calc)	ppm	Miss	Score	Expect	Rank	Unique	Peptide
5969	605.317	1208.620	1208.619	1.17	0	24	0.035	1	U	K.VDTPLQAYFR.I

182. DECOY_tr|Q8Y5Y6 **Mass:** 86080 **Score:** 24 **Matches:** 2(2) **Sequences:** 1(1) **emPAI:** 0.05
PflA protein OS=Listeria monocytogenes serovar 1/2a (strain ATCC BAA-679 / EGD-e) OX=169963 GN=pflA PE=4 SV=1

Query	Observed	Mr(expt)	Mr(calc)	ppm	Miss	Score	Expect	Rank	Unique	Peptide
1437	427.200	852.386	852.398	-13.90	0	22	0.012	1	U	K.IDDTYAR.F 1438

183. sp|Q8Y5G1 **Mass:** 20544 **Score:** 24 **Matches:** 1(1) **Sequences:** 1(1) **emPAI:** 0.23
Pyridoxal 5'-phosphate synthase subunit PdxT OS=Listeria monocytogenes serovar 1/2a (strain ATCC BAA-679 / EGD-e) OX=169963 GN=pdxT PE=3 SV=1

Query	Observed	Mr(expt)	Mr(calc)	ppm	Miss	Score	Expect	Rank	Unique	Peptide
9011	729.054	2184.140	2184.143	-1.10	0	24	0.0091	1	U	R.APYLIEPSNEVAVLATVENR.I

184. DECOY_tr|Q8Y859 **Mass:** 20029 **Score:** 23 **Matches:** 2(2) **Sequences:** 1(1) **emPAI:** 0.23
Lmo1059 protein OS=Listeria monocytogenes serovar 1/2a (strain ATCC BAA-679 / EGD-e) OX=169963 GN=lmo1059 PE=4 SV=1

Query	Observed	Mr(expt)	Mr(calc)	ppm	Miss	Score	Expect	Rank	Unique	Peptide
4868	562.314	1122.613	1122.603	8.77	1	22	0.018	1	U	K.SYIKNITER.T 4865

185. DECOY_tr|Q8Y3V9 **Mass:** 28563 **Score:** 23 **Matches:** 1(1) **Sequences:** 1(1) **emPAI:** 0.16
Lmo2722 protein OS=Listeria monocytogenes serovar 1/2a (strain ATCC BAA-679 / EGD-e) OX=169963 GN=lmo2722 PE=4 SV=1

Query	Observed	Mr(expt)	Mr(calc)	ppm	Miss	Score	Expect	Rank	Unique	Peptide
777	401.739	801.463	801.475	-15.15	1	23	0.025	1	U	R.DILWKK.R

186. DECOY_sp|P00921 **Mass:** 29096 **Score:** 22 **Matches:** 1(1) **Sequences:** 1(1) **emPAI:** 0.16
Carbonic anhydrase 2 (MW-Marker) OS=Bos taurus GN=CA2 PE=1 SV=3

Query	Observed	Mr(expt)	Mr(calc)	ppm	Miss	Score	Expect	Rank	Unique	Peptide
2961	487.286	972.557	972.549	7.61	0	22	0.033	1	U	K.ISDLADLVK.Q

187. tr|Q8Y4S2 **Mass:** 97191 **Score:** 22 **Matches:** 1(1) **Sequences:** 1(1) **emPAI:** 0.04
Transmembrane protein OS=Listeria monocytogenes serovar 1/2a (strain ATCC BAA-679 / EGD-e) OX=169963 GN=lmo2360 PE=4 SV=1

Query	Observed	Mr(expt)	Mr(calc)	ppm	Miss	Score	Expect	Rank	Unique	Peptide
4488	547.296	1092.577	1092.564	12.4	0	22	0.021	1	U	K.TFNMIAAPTK.L

188. tr|Q8Y3P4 **Mass:** 32149 **Score:** 22 **Matches:** 1(1) **Sequences:** 1(1) **emPAI:** 0.14
Partition protein ParB homolg OS=Listeria monocytogenes serovar 1/2a (strain ATCC BAA-679 / EGD-e) OX=169963 GN=parB PE=3 SV=1

Query	Observed	Mr(expt)	Mr(calc)	ppm	Miss	Score	Expect	Rank	Unique	Peptide
324	372.744	743.473	743.469	4.68	0	22	0.013	1	U	R.VPIFIR.E

189. tr|Q8Y4C5 **Mass:** 35785 **Score:** 22 **Matches:** 1(1) **Sequences:** 1(1) **emPAI:** 0.12
Mbl protein OS=Listeria monocytogenes serovar 1/2a (strain ATCC BAA-679 / EGD-e) OX=169963 GN=mbl PE=4 SV=1

Query	Observed	Mr(expt)	Mr(calc)	ppm	Miss	Score	Expect	Rank	Unique	Peptide
5838	601.326	1200.638	1200.646	-7.24	0	22	0.052	1	U	K.TGQVLAVGTEAR.D

190. tr|Q7AP59 **Mass:** 18435 **Score:** 22 **Matches:** 1(1) **Sequences:** 1(1) **emPAI:** 0.25
Lmo1601 protein OS=Listeria monocytogenes serovar 1/2a (strain ATCC BAA-679 / EGD-e) OX=169963 GN=lmo1601 PE=4 SV=1

Query	Observed	Mr(expt)	Mr(calc)	ppm	Miss	Score	Expect	Rank	Unique	Peptide
12176	568.280	1701.819	1701.817	1.36	1	22	0.016	1	U	K.EVVSQNKEENQEAAK.K

191. tr|Q8Y9F6 **Mass:** 39936 **Score:** 22 **Matches:** 1(1) **Sequences:** 1(1) **emPAI:** 0.11
Lmo0572 protein OS=Listeria monocytogenes serovar 1/2a (strain ATCC BAA-679 / EGD-e) OX=169963 GN=lmo0572 PE=4 SV=1

Query	Observed	Mr(expt)	Mr(calc)	ppm	Miss	Score	Expect	Rank	Unique	Peptide
2298	460.239	918.462	918.466	-3.65	0	22	0.025	1	U	K.GITGTDLDK.G

192. tr|Q8YAV5 **Mass:** 56730 **Score:** 22 **Matches:** 1(1) **Sequences:** 1(1) **emPAI:** 0.08
Cardiolipin synthase OS=Listeria monocytogenes serovar 1/2a (strain ATCC BAA-679 / EGD-e) OX=169963 GN=lmo0008 PE=3 SV=1

Query	Observed	Mr(expt)	Mr(calc)	ppm	Miss	Score	Expect	Rank	Unique	Peptide
2110	452.757	903.498	903.518	-21.41	0	22	0.024	1	U	R.LLLFDQR.N

193. DECOY_sp|Q8Y789 **Mass:** 98598 **Score:** 22 **Matches:** 1(1) **Sequences:** 1(1) **emPAI:** 0.04
DNA mismatch repair protein MutS OS=Listeria monocytogenes serovar 1/2a (strain ATCC BAA-679 / EGD-e) OX=169963 GN=mutS PE=3 SV=1

Query	Observed	Mr(expt)	Mr(calc)	ppm	Miss	Score	Expect	Rank	Unique	Peptide
514	385.229	768.444	768.461	-22.24	1	22	0.0066	1	U	K.RDILPR.D

194. sp|Q8Y4I2 **Mass:** 42135 **Score:** 22 **Matches:** 1(1) **Sequences:** 1(1) **emPAI:** 0.10
Phosphoglycerate kinase OS=Listeria monocytogenes serovar 1/2a (strain ATCC BAA-679 / EGD-e) OX=169963 GN=pgk PE=3 SV=1

Query	Observed	Mr(expt)	Mr(calc)	ppm	Miss	Score	Expect	Rank	Unique	Peptide
195	360.707	719.400	719.397	5.08	0	22	0.022	1	U	K.FVPTTR.G

195. tr|Q8Y7L4 **Mass:** 8455 **Score:** 21 **Matches:** 2(1) **Sequences:** 1(1) **emPAI:** 0.62
Lmo1263 protein OS=Listeria monocytogenes serovar 1/2a (strain ATCC BAA-679 / EGD-e) OX=169963 GN=lmo1263 PE=4 SV=1

Query	Observed	Mr(expt)	Mr(calc)	ppm	Miss	Score	Expect	Rank	Unique	Peptide
1367	425.729	849.443	849.438	6.28	1	21	0.036	1	U	-.MKTVDTR.R 1366

196. tr|Q8Y6V1 **Mass:** 16971 **Score:** 21 **Matches:** 1(1) **Sequences:** 1(1) **emPAI:** 0.28
Universal stress protein OS=Listeria monocytogenes serovar 1/2a (strain ATCC BAA-679 / EGD-e) OX=169963 GN=lmo1580 PE=3 SV=1

Query	Observed	Mr(expt)	Mr(calc)	ppm	Miss	Score	Expect	Rank	Unique	Peptide
1867	444.262	886.510	886.512	-2.60	0	21	0.043	1	U	R.VLVAVDGSK.E

197. sp|Q8Y6T4 **Mass:** 47678 **Score:** 21 **Matches:** 1(1) **Sequences:** 1(1) **emPAI:** 0.09
Tyrosine--tRNA ligase OS=Listeria monocytogenes serovar 1/2a (strain ATCC BAA-679 / EGD-e) OX=169963 GN=tyrS PE=3 SV=1

Query	Observed	Mr(expt)	Mr(calc)	ppm	Miss	Score	Expect	Rank	Unique	Peptide
5474	587.368	1172.721	1172.717	3.46	0	21	0.0081	1	U	K.AFGLTIPLLTK.A

198. sp|Q8Y832 **Mass:** 26809 **Score:** 21 **Matches:** 1(1) **Sequences:** 1(1) **emPAI:** 0.17
Ribitol-5-phosphate cytidylyltransferase OS=Listeria monocytogenes serovar 1/2a (strain ATCC BAA-679 / EGD-e) OX=169963 GN=tarI PE=3 SV=1

Query	Observed	Mr(expt)	Mr(calc)	ppm	Miss	Score	Expect	Rank	Unique	Peptide
394	378.251	754.488	754.495	-9.31	0	21	0.0087	1	U	K.IIIATPK.D

199. sp|Q8Y757 **Mass:** 33911 **Score:** 20 **Matches:** 1(1) **Sequences:** 1(1) **emPAI:** 0.13
Probable manganese-dependent inorganic pyrophosphatase OS=Listeria monocytogenes serovar 1/2a (strain ATCC BAA-679 / EGD-e) OX=169963 GN=ppaC PE=3

Query	Observed	Mr(expt)	Mr(calc)	ppm	Miss	Score	Expect	Rank	Unique	Peptide
3602	515.275	1028.536	1028.525	10.5	0	20	0.032	1	U	K.AQGADIEAVR.L

200. tr|Q8YAM0 **Mass:** 33447 **Score:** 20 **Matches:** 1(1) **Sequences:** 1(1) **emPAI:** 0.13
Lmo0098 protein OS=Listeria monocytogenes serovar 1/2a (strain ATCC BAA-679 / EGD-e) OX=169963 GN=lmo0098 PE=4 SV=1

Query	Observed	Mr(expt)	Mr(calc)	ppm	Miss	Score	Expect	Rank	Unique	Peptide
10445	794.920	1587.825	1587.836	-6.78	0	20	0.024	1	U	K.ITDDLSGGLLQDITK.G

201. sp|Q8Y4F5 **Mass:** 75686 **Score:** 20 **Matches:** 1(1) **Sequences:** 1(1) **emPAI:** 0.06
UvrABC system protein B OS=Listeria monocytogenes serovar 1/2a (strain ATCC BAA-679 / EGD-e) OX=169963 GN=uvrB PE=3 SV=1

Query	Observed	Mr(expt)	Mr(calc)	ppm	Miss	Score	Expect	Rank	Unique	Peptide
245	365.209	728.403	728.418	-20.94	0	20	0.023	1	U	K.LLEAQR.L

202. sp|Q8YAC7 **Mass:** 74379 **Score:** 20 **Matches:** 1(1) **Sequences:** 1(1) **emPAI:** 0.06
Bifunctional protein TilS/HprT OS=Listeria monocytogenes serovar 1/2a (strain ATCC BAA-679 / EGD-e) OX=169963 GN=tilS/hprT PE=3 SV=1

Query	Observed	Mr(expt)	Mr(calc)	ppm	Miss	Score	Expect	Rank	Unique	Peptide
7184	651.831	1301.648	1301.671	-18.07	0	20	0.029	1	U	K.VLISEDELQEK.I

203. tr|Q8Y6P6 **Mass:** 36021 **Score:** 20 **Matches:** 1(1) **Sequences:** 1(1) **emPAI:** 0.12
Lmo1638 protein OS=Listeria monocytogenes serovar 1/2a (strain ATCC BAA-679 / EGD-e) OX=169963 GN=lmo1638 PE=1 SV=1

Query	Observed	Mr(expt)	Mr(calc)	ppm	Miss	Score	Expect	Rank	Unique	Peptide
169	359.188	716.362	716.345	23.0	0	20	0.022	1	U	K.QGDEIR.I

204. sp|Q8Y5W8 **Mass:** 49113 **Score:** 20 **Matches:** 1(1) **Sequences:** 1(1) **emPAI:** 0.09
GTPase Der OS=Listeria monocytogenes serovar 1/2a (strain ATCC BAA-679 / EGD-e) OX=169963 GN=der PE=3 SV=1

Query	Observed	Mr(expt)	Mr(calc)	ppm	Miss	Score	Expect	Rank	Unique	Peptide
6993	644.347	1286.680	1286.683	-2.71	0	20	0.043	1	U	K.SSILNALLGEDR.V

205. tr|Q8Y5K9 **Mass:** 83024 **Score:** 20 **Matches:** 1(1) **Sequences:** 1(1) **emPAI:** 0.05
Lmo2050 protein OS=Listeria monocytogenes serovar 1/2a (strain ATCC BAA-679 / EGD-e) OX=169963 GN=lmo2050 PE=4 SV=1

Query	Observed	Mr(expt)	Mr(calc)	ppm	Miss	Score	Expect	Rank	Unique	Peptide
8	350.214	698.413	698.396	23.4	0	20	0.02	1	U	K.LLDPNK.S

206. sp|Q8Y3L0 **Mass:** 39687 **Score:** 19 **Matches:** 1(1) **Sequences:** 1(1) **emPAI:** 0.11
Phosphoserine aminotransferase OS=Listeria monocytogenes serovar 1/2a (strain ATCC BAA-679 / EGD-e) OX=169963 GN=serC PE=3 SV=1

Query	Observed	Mr(expt)	Mr(calc)	ppm	Miss	Score	Expect	Rank	Unique	Peptide
6519	626.296	1250.577	1250.585	-6.70	0	19	0.027	1	U	R.ELMEIPDNYK.V

207. tr|Q8Y9K6 **Mass:** 49245 **Score:** 19 **Matches:** 2(0) **Sequences:** 2(0)
Lmo0521 protein OS=Listeria monocytogenes serovar 1/2a (strain ATCC BAA-679 / EGD-e) OX=169963 GN=lmo0521 PE=3 SV=1

Query	Observed	Mr(expt)	Mr(calc)	ppm	Miss	Score	Expect	Rank	Unique	Peptide
5396	583.296	1164.577	1164.578	-0.69	0	17	0.059	1	U	K.DADFVTTQLR.V
8249	692.373	1382.732	1382.734	-1.46	0	19	0.085	1	U	K.VVGLCNGPIGIER.N

208. tr|Q8Y8E3 **Mass:** 46091 **Score:** 19 **Matches:** 1(1) **Sequences:** 1(1) **emPAI:** 0.10
Lmo0961 protein OS=Listeria monocytogenes serovar 1/2a (strain ATCC BAA-679 / EGD-e) OX=169963 GN=lmo0961 PE=4 SV=1

Query	Observed	Mr(expt)	Mr(calc)	ppm	Miss	Score	Expect	Rank	Unique	Peptide
1046	415.238	828.462	828.482	-23.71	0	19	0.043	1	U	K.IATLQQR.N

209. DECOY_tr|Q8Y587 **Mass:** 29388 **Score:** 19 **Matches:** 1(1) **Sequences:** 1(1) **emPAI:** 0.15
Lmo2182 protein OS=Listeria monocytogenes serovar 1/2a (strain ATCC BAA-679 / EGD-e) OX=169963 GN=lmo2182 PE=4 SV=1

Query	Observed	Mr(expt)	Mr(calc)	ppm	Miss	Score	Expect	Rank	Unique	Peptide
844	405.217	808.419	808.412	9.33	0	19	0.052	1	U	K.WEFISK.G

210. sp|Q8Y5F1 **Mass:** 28947 **Score:** 18 **Matches:** 1(1) **Sequences:** 1(1) **emPAI:** 0.16
Putative heme-dependent peroxidase lmo2113 OS=Listeria monocytogenes serovar 1/2a (strain ATCC BAA-679 / EGD-e) OX=169963 GN=lmo2113 PE=1 SV=1

Query	Observed	Mr(expt)	Mr(calc)	ppm	Miss	Score	Expect	Rank	Unique	Peptide
4329	541.308	1080.601	1080.597	4.31	0	18	0.017	1	U	K.ADLVFFTLR.D

211. tr|Q8Y840 **Mass:** 32510 **Score:** 18 **Matches:** 1(1) **Sequences:** 1(1) **emPAI:** 0.14
UTP--glucose-1-phosphate uridylyltransferase OS=Listeria monocytogenes serovar 1/2a (strain ATCC BAA-679 / EGD-e) OX=169963 GN=lmo1078 PE=3 SV=1

Query	Observed	Mr(expt)	Mr(calc)	ppm	Miss	Score	Expect	Rank	Unique	Peptide
4867	562.314	1122.613	1122.632	-17.56	0	18	0.043	1	U	R.YLLTPEIFK.Y

212. tr|Q8Y4A4 **Mass:** 46370 **Score:** 18 **Matches:** 1(0) **Sequences:** 1(0)
Homoserine dehydrogenase OS=Listeria monocytogenes serovar 1/2a (strain ATCC BAA-679 / EGD-e) OX=169963 GN=hom PE=3 SV=1

Query	Observed	Mr(expt)	Mr(calc)	ppm	Miss	Score	Expect	Rank	Unique	Peptide
3629	516.290	1030.566	1030.566	0.34	0	18	0.13	1	U	R.TIVNSLAADK.I

213. tr|Q8YAA2 **Mass:** 15019 **Score:** 18 **Matches:** 1(0) **Sequences:** 1(0)

Lmo0252 protein OS=Listeria monocytogenes serovar 1/2a (strain ATCC BAA-679 / EGD-e) OX=169963 GN=lmo0252 PE=4 SV=1

Query	Observed	Mr(expt)	Mr(calc)	ppm	Miss	Score	Expect	Rank	Unique	Peptide
1401	426.218	850.422	850.411	13.7	0	18	0.16	1	U	K.SELEVMK.I

214. tr|Q8Y971 **Mass:** 28915 **Score:** 17 **Matches:** 1(1) **Sequences:** 1(1) **emPAI:** 0.16

ThiD protein OS=Listeria monocytogenes serovar 1/2a (strain ATCC BAA-679 / EGD-e) OX=169963 GN=thiD PE=4 SV=1

Query	Observed	Mr(expt)	Mr(calc)	ppm	Miss	Score	Expect	Rank	Unique	Peptide
7010	645.326	1288.637	1288.652	-11.87	0	17	0.055	1	U	K.NVVIDPVMVCK.G

Mascot: http://www.matrixscience.com/
