# Supplementary material for: Impact of osmotic stress on the phosphorylation and subcellular location of Listeria monocytogenes stressosome proteins
Source: Sci Rep. 2020 Nov 30;10:20837. doi: 10.1038/s41598-020-77738-z (PMC7705745; doi:10.1038/s41598-020-77738-z)
Supplement: Supplementary file 1 — Supplementary Information. [file 41598_2020_77738_MOESM1_ESM.zip › Dessaux-et-al-Supp-Information/MS-Proteomics-3-assays/WT-membrane-assay-exp3.pdf]

MATRIX SCIENCE Mascot Search Results

User	:
Email	:
Search title	: 20181004_Charlotte_CNB_Mb_WT
MS data file	: 20181004_Charlotte_CNB_Mb_WT.mgf
Database	: UKBsp_p169963_TD p169963_TD_20180702 (5856 sequences; 1791414 residues)
Timestamp	: 5 Oct 2018 at 14:58:26 GMT
Enzyme	: Trypsin
Fixed modifications	: Carbamidomethyl (C)
Variable modifications	: Acetyl (Protein N-term),Oxidation (M)
Mass values	: Monoisotopic
Protein Mass	: Unrestricted
Peptide Mass Tolerance	: ± 25 ppm
Fragment Mass Tolerance	: ± 0.1 Da
Max Missed Cleavages	: 1
Instrument type	: ESI-QUAD-TOF
Number of queries	: 20553

Protein hits :

sp|P02769	Serum albumin (Laboratory-Cont) OS=Bos taurus GN=ALB PE=1 SV=4
tr|Q8Y8K9	RsbR protein OS=Listeria monocytogenes serovar 1/2a (strain ATCC BAA-679 / EGD-e) OX=169963 GN=RsbR PE=4 SV=1
sp|P04264	Keratin, type II cytoskeletal 1 (Contact-Cont) OS=Homo sapiens GN=KRT1 PE=1 SV=6
sp|P13645	Keratin, type I cytoskeletal 10 (Contact-Cont) OS=Homo sapiens GN=KRT10 PE=1 SV=6
sp|P35908	Keratin, type II cytoskeletal 2 epidermal (Contact-Cont) OS=Homo sapiens GN=KRT2 PE=1 SV=2
sp|P00761	Trypsin (Laboratory-Cont) OS=Sus scrofa PE=1 SV=1
tr|Q8Y863	Dihydrolipoamide acetyltransferase component of pyruvate dehydrogenase complex OS=Listeria monocytogenes serovar
sp|P58724	Blue-light photoreceptor OS=Listeria monocytogenes serovar 1/2a (strain ATCC BAA-679 / EGD-e) OX=169963 GN=lmo07
sp|P35527	Keratin, type I cytoskeletal 9 (Contact-Cont) OS=Homo sapiens GN=KRT9 PE=1 SV=3
sp|Q8Y822	GMP synthase [glutamine-hydrolyzing] OS=Listeria monocytogenes serovar 1/2a (strain ATCC BAA-679 / EGD-e) OX=169
tr|Q8Y4U6	FruA protein OS=Listeria monocytogenes serovar 1/2a (strain ATCC BAA-679 / EGD-e) OX=169963 GN=fruA PE=4 SV=1
tr|Q8Y435	Lmo2638 protein OS=Listeria monocytogenes serovar 1/2a (strain ATCC BAA-679 / EGD-e) OX=169963 GN=lmo2638 PE=4 S
tr|Q8YAG5	Lmo0161 protein OS=Listeria monocytogenes serovar 1/2a (strain ATCC BAA-679 / EGD-e) OX=169963 GN=lmo0161 PE=4 S
sp|Q48754	CD4+ T-cell-stimulating antigen OS=Listeria monocytogenes serovar 1/2a (strain ATCC BAA-679 / EGD-e) OX=169963 G
sp|Q8Y6M6	30S ribosomal protein S2 OS=Listeria monocytogenes serovar 1/2a (strain ATCC BAA-679 / EGD-e) OX=169963 GN=rpsB
tr|Q8Y6P2	Lmo1642 protein OS=Listeria monocytogenes serovar 1/2a (strain ATCC BAA-679 / EGD-e) OX=169963 GN=lmo1642 PE=4 S
sp|Q8Y422	Elongation factor Tu OS=Listeria monocytogenes serovar 1/2a (strain ATCC BAA-679 / EGD-e) OX=169963 GN=tuf PE=1
tr|Q8YAM0	Lmo0098 protein OS=Listeria monocytogenes serovar 1/2a (strain ATCC BAA-679 / EGD-e) OX=169963 GN=lmo0098 PE=4 S
tr|Q7AP52	Lmo2196 protein OS=Listeria monocytogenes serovar 1/2a (strain ATCC BAA-679 / EGD-e) OX=169963 GN=lmo2196 PE=4 S
sp|Q8Y440	50S ribosomal protein L3 OS=Listeria monocytogenes serovar 1/2a (strain ATCC BAA-679 / EGD-e) OX=169963 GN=rplC
tr|Q8Y7B2	Dihydrolipoamide acetyltransferase component of pyruvate dehydrogenase complex OS=Listeria monocytogenes serovar
sp|O77727	Keratin, type I cytoskeletal 15 (Contact-Cont) OS=Ovis aries GN=KRT15 PE=2 SV=1
sp|P0DJM2	Chaperone protein DnaK OS=Listeria monocytogenes serovar 1/2a (strain ATCC BAA-679 / EGD-e) OX=169963 GN=dnaK PE
tr|Q8Y865	PdhA protein OS=Listeria monocytogenes serovar 1/2a (strain ATCC BAA-679 / EGD-e) OX=169963 GN=pdhA PE=4 SV=1
tr|Q8YAJ0	Lmo0135 protein OS=Listeria monocytogenes serovar 1/2a (strain ATCC BAA-679 / EGD-e) OX=169963 GN=lmo0135 PE=1 S
sp|Q92EP8	UPF0365 protein lmo0392 OS=Listeria monocytogenes serovar 1/2a (strain ATCC BAA-679 / EGD-e) OX=169963 GN=lmo039
sp|Q8Y4C1	ATP synthase subunit beta 2 OS=Listeria monocytogenes serovar 1/2a (strain ATCC BAA-679 / EGD-e) OX=169963 GN=at
sp|Q9AGE6	60 kDa chaperonin OS=Listeria monocytogenes serovar 1/2a (strain ATCC BAA-679 / EGD-e) OX=169963 GN=groL PE=3 SV
sp|Q8Y446	30S ribosomal protein S5 OS=Listeria monocytogenes serovar 1/2a (strain ATCC BAA-679 / EGD-e) OX=169963 GN=rpsE
tr|Q8Y4P6	Lmo2389 protein OS=Listeria monocytogenes serovar 1/2a (strain ATCC BAA-679 / EGD-e) OX=169963 GN=lmo2389 PE=4 S
tr|Q8Y864	PdhB protein OS=Listeria monocytogenes serovar 1/2a (strain ATCC BAA-679 / EGD-e) OX=169963 GN=PdhB PE=4 SV=1
tr|Q8Y841	Lmo1077 protein OS=Listeria monocytogenes serovar 1/2a (strain ATCC BAA-679 / EGD-e) OX=169963 GN=lmo1077 PE=4 S
sp|Q8YAA4	50S ribosomal protein L1 OS=Listeria monocytogenes serovar 1/2a (strain ATCC BAA-679 / EGD-e) OX=169963 GN=rplA
tr|Q7AP53	Lmo2193 protein OS=Listeria monocytogenes serovar 1/2a (strain ATCC BAA-679 / EGD-e) OX=169963 GN=lmo2193 PE=3 S
sp|Q8Y4B3	Uracil phosphoribosyltransferase OS=Listeria monocytogenes serovar 1/2a (strain ATCC BAA-679 / EGD-e) OX=169963
sp|Q8Y6M7	Elongation factor Ts OS=Listeria monocytogenes serovar 1/2a (strain ATCC BAA-679 / EGD-e) OX=169963 GN=tsf PE=3
sp|P66548	30S ribosomal protein S3 OS=Listeria monocytogenes serovar 1/2a (strain ATCC BAA-679 / EGD-e) OX=169963 GN=rpsC
sp|Q927L9	50S ribosomal protein L5 OS=Listeria monocytogenes serovar 1/2a (strain ATCC BAA-679 / EGD-e) OX=169963 GN=rplE
sp|P61055	50S ribosomal protein L4 OS=Listeria monocytogenes serovar 1/2a (strain ATCC BAA-679 / EGD-e) OX=169963 GN=rplD
sp|P66042	50S ribosomal protein L10 OS=Listeria monocytogenes serovar 1/2a (strain ATCC BAA-679 / EGD-e) OX=169963 GN=rplJ
tr|Q8Y5H3	Lmo2089 protein OS=Listeria monocytogenes serovar 1/2a (strain ATCC BAA-679 / EGD-e) OX=169963 GN=lmo2089 PE=4 S
sp|Q8Y6Y9	50S ribosomal protein L21 OS=Listeria monocytogenes serovar 1/2a (strain ATCC BAA-679 / EGD-e) OX=169963 GN=rplU
sp|Q8Y4C0	ATP synthase subunit alpha 2 OS=Listeria monocytogenes serovar 1/2a (strain ATCC BAA-679 / EGD-e) OX=169963 GN=a
tr|Q8Y795	Lmo1395 protein OS=Listeria monocytogenes serovar 1/2a (strain ATCC BAA-679 / EGD-e) OX=169963 GN=lmo1395 PE=4 S
sp|Q927L7	50S ribosomal protein L14 OS=Listeria monocytogenes serovar 1/2a (strain ATCC BAA-679 / EGD-e) OX=169963 GN=rplN
tr|Q7AP59	Lmo1601 protein OS=Listeria monocytogenes serovar 1/2a (strain ATCC BAA-679 / EGD-e) OX=169963 GN=lmo1601 PE=4 S
tr|Q8YAC6	ATP-dependent zinc metalloprotease FtsH OS=Listeria monocytogenes serovar 1/2a (strain ATCC BAA-679 / EGD-e) OX=
tr|Q8Y436	Lmo2637 protein OS=Listeria monocytogenes serovar 1/2a (strain ATCC BAA-679 / EGD-e) OX=169963 GN=lmo2637 PE=4 S
tr|Q8Y7I0	Glutamine synthetase OS=Listeria monocytogenes serovar 1/2a (strain ATCC BAA-679 / EGD-e) OX=169963 GN=glnA PE=3
tr|Q8Y4I1	Glyceraldehyde-3-phosphate dehydrogenase OS=Listeria monocytogenes serovar 1/2a (strain ATCC BAA-679 / EGD-e) OX
tr|Q8Y6Y3	MreB protein OS=Listeria monocytogenes serovar 1/2a (strain ATCC BAA-679 / EGD-e) OX=169963 GN=mreB PE=4 SV=1
tr|Q8Y6Q0	Aldehyde-alcohol dehydrogenase OS=Listeria monocytogenes serovar 1/2a (strain ATCC BAA-679 / EGD-e) OX=169963 GN
tr|Q8Y581	Lmo2192 protein OS=Listeria monocytogenes serovar 1/2a (strain ATCC BAA-679 / EGD-e) OX=169963 GN=lmo2192 PE=3 S
tr|Q8Y8V1	Lmo0791 protein OS=Listeria monocytogenes serovar 1/2a (strain ATCC BAA-679 / EGD-e) OX=169963 GN=lmo0791 PE=4 S
sp|P66054	50S ribosomal protein L11 OS=Listeria monocytogenes serovar 1/2a (strain ATCC BAA-679 / EGD-e) OX=169963 GN=rplK
sp|P66611	30S ribosomal protein S7 OS=Listeria monocytogenes serovar 1/2a (strain ATCC BAA-679 / EGD-e) OX=169963 GN=rpsG
sp|P64074	Enolase OS=Listeria monocytogenes serovar 1/2a (strain ATCC BAA-679 / EGD-e) OX=169963 GN=eno PE=1 SV=1
sp|Q92DC5	RsbS protein OS=Listeria monocytogenes serovar 1/2a (strain ATCC BAA-679 / EGD-e) OX=169963 GN=rsbS PE=4 SV=1
sp|Q8Y6T6	30S ribosomal protein S4 OS=Listeria monocytogenes serovar 1/2a (strain ATCC BAA-679 / EGD-e) OX=169963 GN=rpsD
sp|P02662	Alpha-S1-casein (Laboratory-Cont) OS=Bos taurus GN=CSN1S1 PE=1 SV=2
sp|P02663	Alpha-S2-casein (Laboratory-Cont) OS=Bos taurus GN=CSN1S2 PE=1 SV=2
sp|Q927L2	50S ribosomal protein L22 OS=Listeria monocytogenes serovar 1/2a (strain ATCC BAA-679 / EGD-e) OX=169963 GN=rplV
tr|Q8Y6W1	Pyruvate kinase OS=Listeria monocytogenes serovar 1/2a (strain ATCC BAA-679 / EGD-e) OX=169963 GN=pykA PE=3 SV=1
tr|Q8Y4C5	Mbl protein OS=Listeria monocytogenes serovar 1/2a (strain ATCC BAA-679 / EGD-e) OX=169963 GN=mbl PE=4 SV=1
tr|Q926Y9	Inosine-5'-monophosphate dehydrogenase OS=Listeria monocytogenes serovar 1/2a (strain ATCC BAA-679 / EGD-e) OX=1
sp|P66352	30S ribosomal protein S11 OS=Listeria monocytogenes serovar 1/2a (strain ATCC BAA-679 / EGD-e) OX=169963 GN=rpsK
tr|Q7AP76	GbuA protein OS=Listeria monocytogenes serovar 1/2a (strain ATCC BAA-679 / EGD-e) OX=169963 GN=gbuA PE=4 SV=1
tr|Q8Y701	Lmo1529 protein OS=Listeria monocytogenes serovar 1/2a (strain ATCC BAA-679 / EGD-e) OX=169963 GN=lmo1529 PE=4 S
sp|Q8Y9L8	Putative ribose-phosphate pyrophosphokinase 2 OS=Listeria monocytogenes serovar 1/2a (strain ATCC BAA-679 / EGD-
sp|Q8Y444	50S ribosomal protein L6 OS=Listeria monocytogenes serovar 1/2a (strain ATCC BAA-679 / EGD-e) OX=169963 GN=rplF
sp|Q8Y450	50S ribosomal protein L17 OS=Listeria monocytogenes serovar 1/2a (strain ATCC BAA-679 / EGD-e) OX=169963 GN=rplQ
tr|Q8Y5M4	Cell division protein FtsA OS=Listeria monocytogenes serovar 1/2a (strain ATCC BAA-679 / EGD-e) OX=169963 GN=fts
sp|P66330	30S ribosomal protein S10 OS=Listeria monocytogenes serovar 1/2a (strain ATCC BAA-679 / EGD-e) OX=169963 GN=rpsJ
sp|Q8Y447	50S ribosomal protein L15 OS=Listeria monocytogenes serovar 1/2a (strain ATCC BAA-679 / EGD-e) OX=169963 GN=rplO
sp|Q8YAA3	50S ribosomal protein L7/L12 OS=Listeria monocytogenes serovar 1/2a (strain ATCC BAA-679 / EGD-e) OX=169963 GN=r
sp|P0DJP0	Protein RecA OS=Listeria monocytogenes serovar 1/2a (strain ATCC BAA-679 / EGD-e) OX=169963 GN=recA PE=3 SV=1
tr|Q8Y980	Lmo0653 protein OS=Listeria monocytogenes serovar 1/2a (strain ATCC BAA-679 / EGD-e) OX=169963 GN=lmo0653 PE=4 S
tr|Q8Y7B5	Dihydrolipoyl dehydrogenase OS=Listeria monocytogenes serovar 1/2a (strain ATCC BAA-679 / EGD-e) OX=169963 GN=lm
sp|Q9AGE7	10 kDa chaperonin OS=Listeria monocytogenes serovar 1/2a (strain ATCC BAA-679 / EGD-e) OX=169963 GN=groS PE=3 SV
tr|Q8YAH4	Lmo0152 protein OS=Listeria monocytogenes serovar 1/2a (strain ATCC BAA-679 / EGD-e) OX=169963 GN=lmo0152 PE=4 S
tr|Q8Y4R3	Lmo2371 protein OS=Listeria monocytogenes serovar 1/2a (strain ATCC BAA-679 / EGD-e) OX=169963 GN=lmo2371 PE=4 S
tr|Q8Y6M1	AnsB protein OS=Listeria monocytogenes serovar 1/2a (strain ATCC BAA-679 / EGD-e) OX=169963 GN=ansB PE=4 SV=1
tr|Q8Y8E2	Listeria epitope LemA OS=Listeria monocytogenes serovar 1/2a (strain ATCC BAA-679 / EGD-e) OX=169963 GN=lemA PE=
sp|Q8Y699	30S ribosomal protein S16 OS=Listeria monocytogenes serovar 1/2a (strain ATCC BAA-679 / EGD-e) OX=169963 GN=rpsP
tr|Q8YAM1	Lmo0097 protein OS=Listeria monocytogenes serovar 1/2a (strain ATCC BAA-679 / EGD-e) OX=169963 GN=lmo0097 PE=4 S
tr|Q8Y580	Lmo2194 protein OS=Listeria monocytogenes serovar 1/2a (strain ATCC BAA-679 / EGD-e) OX=169963 GN=lmo2194 PE=3 S
tr|Q8Y7N8	Lmo1236 protein OS=Listeria monocytogenes serovar 1/2a (strain ATCC BAA-679 / EGD-e) OX=169963 GN=lmo1236 PE=4 S

tr|Q927C3	CydA protein OS=Listeria monocytogenes serovar 1/2a (strain ATCC BAA-679 / EGD-e) OX=169963 GN=cydA PE=4 SV=1
tr|Q8Y693	Signal recognition particle receptor FtsY OS=Listeria monocytogenes serovar 1/2a (strain ATCC BAA-679 / EGD-e) O
tr|Q8Y6Y7	Site-determining protein OS=Listeria monocytogenes serovar 1/2a (strain ATCC BAA-679 / EGD-e) OX=169963 GN=minD
tr|Q8Y5F0	Lmo2114 protein OS=Listeria monocytogenes serovar 1/2a (strain ATCC BAA-679 / EGD-e) OX=169963 GN=lmo2114 PE=4 S
tr|Q8Y8P9	Lmo0847 protein OS=Listeria monocytogenes serovar 1/2a (strain ATCC BAA-679 / EGD-e) OX=169963 GN=lmo0847 PE=3 S
tr|Q8YAB6	Endopeptidase Clp ATP-binding chain C OS=Listeria monocytogenes serovar 1/2a (strain ATCC BAA-679 / EGD-e) OX=16
tr|Q8Y5W4	Lmo1941 protein OS=Listeria monocytogenes serovar 1/2a (strain ATCC BAA-679 / EGD-e) OX=169963 GN=lmo1941 PE=4 S
sp|P47847	Protein translocase subunit SecA 1 OS=Listeria monocytogenes serovar 1/2a (strain ATCC BAA-679 / EGD-e) OX=16996
sp|P02666	Beta-casein (Laboratory-Cont) OS=Bos taurus GN=CSN2 PE=1 SV=2
sp|Q8Y458	50S ribosomal protein L13 OS=Listeria monocytogenes serovar 1/2a (strain ATCC BAA-679 / EGD-e) OX=169963 GN=rplM
tr|Q8YA67	Lmo0292 protein OS=Listeria monocytogenes serovar 1/2a (strain ATCC BAA-679 / EGD-e) OX=169963 GN=lmo0292 PE=4 S
sp|Q8Y421	Elongation factor G OS=Listeria monocytogenes serovar 1/2a (strain ATCC BAA-679 / EGD-e) OX=169963 GN=fusA PE=3
tr|Q8Y4E0	FtsE protein OS=Listeria monocytogenes serovar 1/2a (strain ATCC BAA-679 / EGD-e) OX=169963 GN=ftsE PE=4 SV=1
tr|Q8YAM2	Lmo0096 protein OS=Listeria monocytogenes serovar 1/2a (strain ATCC BAA-679 / EGD-e) OX=169963 GN=lmo0096 PE=4 S
tr|Q8YA11	Lmo0355 protein OS=Listeria monocytogenes serovar 1/2a (strain ATCC BAA-679 / EGD-e) OX=169963 GN=lmo0355 PE=4 S
sp|P66623	30S ribosomal protein S8 OS=Listeria monocytogenes serovar 1/2a (strain ATCC BAA-679 / EGD-e) OX=169963 GN=rpsH
tr|Q7AP78	Protein DltD OS=Listeria monocytogenes serovar 1/2a (strain ATCC BAA-679 / EGD-e) OX=169963 GN=dltD PE=3 SV=1
sp|P0DJM1	Chaperone protein DnaJ OS=Listeria monocytogenes serovar 1/2a (strain ATCC BAA-679 / EGD-e) OX=169963 GN=dnaJ PE
sp|Q9RLT9	DNA-directed RNA polymerase subunit beta OS=Listeria monocytogenes serovar 1/2a (strain ATCC BAA-679 / EGD-e) OX
sp|P66383	30S ribosomal protein S13 OS=Listeria monocytogenes serovar 1/2a (strain ATCC BAA-679 / EGD-e) OX=169963 GN=rpsM
tr|Q8Y703	Multifunctional fusion protein OS=Listeria monocytogenes serovar 1/2a (strain ATCC BAA-679 / EGD-e) OX=169963 GN
tr|Q8Y498	FbaA protein OS=Listeria monocytogenes serovar 1/2a (strain ATCC BAA-679 / EGD-e) OX=169963 GN=fbaA PE=3 SV=1
sp|Q8Y443	50S ribosomal protein L24 OS=Listeria monocytogenes serovar 1/2a (strain ATCC BAA-679 / EGD-e) OX=169963 GN=rplX
tr|Q8Y5Q0	AlsS protein OS=Listeria monocytogenes serovar 1/2a (strain ATCC BAA-679 / EGD-e) OX=169963 GN=alsS PE=3 SV=1
tr|Q8Y6S2	Lmo1611 protein OS=Listeria monocytogenes serovar 1/2a (strain ATCC BAA-679 / EGD-e) OX=169963 GN=lmo1611 PE=3 S
sp|Q8Y459	30S ribosomal protein S9 OS=Listeria monocytogenes serovar 1/2a (strain ATCC BAA-679 / EGD-e) OX=169963 GN=rpsI
tr|Q929C7	Lmo2248 protein OS=Listeria monocytogenes serovar 1/2a (strain ATCC BAA-679 / EGD-e) OX=169963 GN=lmo2248 PE=4 S
sp|Q8Y5G2	Pyridoxal 5'-phosphate synthase subunit PdxS OS=Listeria monocytogenes serovar 1/2a (strain ATCC BAA-679 / EGD-e
sp|Q8Y5K2	Heme A synthase OS=Listeria monocytogenes serovar 1/2a (strain ATCC BAA-679 / EGD-e) OX=169963 GN=ctaA PE=3 SV=1
tr|Q8Y862	Dihydrolipoyl dehydrogenase OS=Listeria monocytogenes serovar 1/2a (strain ATCC BAA-679 / EGD-e) OX=169963 GN=Pd
tr|Q8Y4K7	Lmo2431 protein OS=Listeria monocytogenes serovar 1/2a (strain ATCC BAA-679 / EGD-e) OX=169963 GN=lmo2431 PE=4 S
sp|Q8Y7G3	Putative zinc metalloprotease Lmo1318 OS=Listeria monocytogenes serovar 1/2a (strain ATCC BAA-679 / EGD-e) OX=16
sp|Q8YA96	DNA-directed RNA polymerase subunit beta' OS=Listeria monocytogenes serovar 1/2a (strain ATCC BAA-679 / EGD-e) O
tr|Q8Y7L9	Lmo1255 protein OS=Listeria monocytogenes serovar 1/2a (strain ATCC BAA-679 / EGD-e) OX=169963 GN=lmo1255 PE=4 S
sp|O53083	50S ribosomal protein L19 OS=Listeria monocytogenes serovar 1/2a (strain ATCC BAA-679 / EGD-e) OX=169963 GN=rplS
tr|Q8Y6T3	Catabolite control protein A OS=Listeria monocytogenes serovar 1/2a (strain ATCC BAA-679 / EGD-e) OX=169963 GN=c
tr|Q8Y9I7	Lmo0541 protein OS=Listeria monocytogenes serovar 1/2a (strain ATCC BAA-679 / EGD-e) OX=169963 GN=lmo0541 PE=4 S
sp|Q8Y8N0	ATP-dependent RNA helicase CshA OS=Listeria monocytogenes serovar 1/2a (strain ATCC BAA-679 / EGD-e) OX=169963 G
sp|Q8Y651	Manganese transport system ATP-binding protein MntB OS=Listeria monocytogenes serovar 1/2a (strain ATCC BAA-679
tr|Q8Y4S2	Transmembrane protein OS=Listeria monocytogenes serovar 1/2a (strain ATCC BAA-679 / EGD-e) OX=169963 GN=lmo2360
tr|Q8Y5E9	Lmo2115 protein OS=Listeria monocytogenes serovar 1/2a (strain ATCC BAA-679 / EGD-e) OX=169963 GN=lmo2115 PE=4 S
tr|Q8YAR3	Lmo0052 protein OS=Listeria monocytogenes serovar 1/2a (strain ATCC BAA-679 / EGD-e) OX=169963 GN=lmo0052 PE=4 S
sp|Q8Y915	Glutamine--fructose-6-phosphate aminotransferase [isomerizing] OS=Listeria monocytogenes serovar 1/2a (strain AT
sp|P66372	30S ribosomal protein S12 OS=Listeria monocytogenes serovar 1/2a (strain ATCC BAA-679 / EGD-e) OX=169963 GN=rpsL
sp|P60384	Redox-sensing transcriptional repressor Rex OS=Listeria monocytogenes serovar 1/2a (strain ATCC BAA-679 / EGD-e)
sp|Q8Y7F1	Polyribonucleotide nucleotidyltransferase OS=Listeria monocytogenes serovar 1/2a (strain ATCC BAA-679 / EGD-e) O
tr|Q7AP48	Lmo2550 protein OS=Listeria monocytogenes serovar 1/2a (strain ATCC BAA-679 / EGD-e) OX=169963 GN=lmo2550 PE=4 S
sp|Q48793	Ribose-phosphate pyrophosphokinase 1 OS=Listeria monocytogenes serovar 1/2a (strain ATCC BAA-679 / EGD-e) OX=169
tr|Q8Y4E1	Cell division protein FtsX OS=Listeria monocytogenes serovar 1/2a (strain ATCC BAA-679 / EGD-e) OX=169963 GN=fts
sp|Q8Y5V1	Phosphopentomutase OS=Listeria monocytogenes serovar 1/2a (strain ATCC BAA-679 / EGD-e) OX=169963 GN=deoB PE=3 S
sp|Q927W3	ATP synthase gamma chain OS=Listeria monocytogenes serovar 1/2a (strain ATCC BAA-679 / EGD-e) OX=169963 GN=atpG
tr|Q8Y854	Lmo1064 protein OS=Listeria monocytogenes serovar 1/2a (strain ATCC BAA-679 / EGD-e) OX=169963 GN=lmo1064 PE=4 S
sp|P60426	50S ribosomal protein L2 OS=Listeria monocytogenes serovar 1/2a (strain ATCC BAA-679 / EGD-e) OX=169963 GN=rplB
sp|Q8Y7J8	ATP-dependent protease ATPase subunit HslU OS=Listeria monocytogenes serovar 1/2a (strain ATCC BAA-679 / EGD-e)
tr|Q8Y7C7	Acetyl-CoA carboxylase subunit (Biotin carboxylase subunit) OS=Listeria monocytogenes serovar 1/2a (strain ATCC
tr|Q8Y7H4	Transketolase OS=Listeria monocytogenes serovar 1/2a (strain ATCC BAA-679 / EGD-e) OX=169963 GN=tkt PE=3 SV=1
tr|Q8Y5T9	Lmo1966 protein OS=Listeria monocytogenes serovar 1/2a (strain ATCC BAA-679 / EGD-e) OX=169963 GN=lmo1966 PE=4 S
DECOY_tr|Q8Y8I5	Lmo0917 protein OS=Listeria monocytogenes serovar 1/2a (strain ATCC BAA-679 / EGD-e) OX=169963 GN=lmo0917 PE=3 S
sp|Q8YAB8	Lysine--tRNA ligase OS=Listeria monocytogenes serovar 1/2a (strain ATCC BAA-679 / EGD-e) OX=169963 GN=lysS PE=3
sp|Q8YAD4	Bifunctional protein GlmU OS=Listeria monocytogenes serovar 1/2a (strain ATCC BAA-679 / EGD-e) OX=169963 GN=glmU
sp|P66401	30S ribosomal protein S14 type Z OS=Listeria monocytogenes serovar 1/2a (strain ATCC BAA-679 / EGD-e) OX=169963
sp|Q8Y441	50S ribosomal protein L23 OS=Listeria monocytogenes serovar 1/2a (strain ATCC BAA-679 / EGD-e) OX=169963 GN=rplW
sp|Q8Y766	4-hydroxy-tetrahydrodipicolinate synthase OS=Listeria monocytogenes serovar 1/2a (strain ATCC BAA-679 / EGD-e) O
DECOY_tr|Q8Y8B9	Lmo0989 protein OS=Listeria monocytogenes serovar 1/2a (strain ATCC BAA-679 / EGD-e) OX=169963 GN=lmo0989 PE=4 S
tr|Q8Y561	Lmo2215 protein OS=Listeria monocytogenes serovar 1/2a (strain ATCC BAA-679 / EGD-e) OX=169963 GN=lmo2215 PE=4 S
DECOY_tr|Q8Y5B2	Ribonucleoside-diphosphate reductase OS=Listeria monocytogenes serovar 1/2a (strain ATCC BAA-679 / EGD-e) OX=169
sp|Q8Y557	Foldase protein PrsA 2 OS=Listeria monocytogenes serovar 1/2a (strain ATCC BAA-679 / EGD-e) OX=169963 GN=prsA2 P
sp|Q8Y624	Formate--tetrahydrofolate ligase OS=Listeria monocytogenes serovar 1/2a (strain ATCC BAA-679 / EGD-e) OX=169963
tr|Q8Y765	Aspartokinase OS=Listeria monocytogenes serovar 1/2a (strain ATCC BAA-679 / EGD-e) OX=169963 GN=lmo1436 PE=3 SV=
tr|Q8Y5V7	ResD protein OS=Listeria monocytogenes serovar 1/2a (strain ATCC BAA-679 / EGD-e) OX=169963 GN=resD PE=4 SV=1
tr|Q8Y486	Lmo2569 protein OS=Listeria monocytogenes serovar 1/2a (strain ATCC BAA-679 / EGD-e) OX=169963 GN=lmo2569 PE=4 S
tr|Q8Y7L4	Lmo1263 protein OS=Listeria monocytogenes serovar 1/2a (strain ATCC BAA-679 / EGD-e) OX=169963 GN=lmo1263 PE=4 S
sp|Q8Y7Q1	Phenylalanine--tRNA ligase beta subunit OS=Listeria monocytogenes serovar 1/2a (strain ATCC BAA-679 / EGD-e) OX=
tr|Q8Y725	Endolytic murein transglycosylase OS=Listeria monocytogenes serovar 1/2a (strain ATCC BAA-679 / EGD-e) OX=169963
tr|Q8Y5P2	Lmo2014 protein OS=Listeria monocytogenes serovar 1/2a (strain ATCC BAA-679 / EGD-e) OX=169963 GN=lmo2014 PE=4 S
DECOY_tr|Q8Y3V9	Lmo2722 protein OS=Listeria monocytogenes serovar 1/2a (strain ATCC BAA-679 / EGD-e) OX=169963 GN=lmo2722 PE=4 S
sp|P02668	Kappa-casein (Laboratory-Cont) OS=Bos taurus GN=CSN3 PE=1 SV=1
tr|Q8Y3T6	Lmo2745 protein OS=Listeria monocytogenes serovar 1/2a (strain ATCC BAA-679 / EGD-e) OX=169963 GN=lmo2745 PE=4 S
tr|Q8YAV5	Cardiolipin synthase OS=Listeria monocytogenes serovar 1/2a (strain ATCC BAA-679 / EGD-e) OX=169963 GN=lmo0008 P
tr|Q8YAD0	Transcription-repair-coupling factor OS=Listeria monocytogenes serovar 1/2a (strain ATCC BAA-679 / EGD-e) OX=169
sp|Q8Y653	Manganese-binding lipoprotein MntA OS=Listeria monocytogenes serovar 1/2a (strain ATCC BAA-679 / EGD-e) OX=16996
sp|Q8Y6Y2	UPF0758 protein lmo1549 OS=Listeria monocytogenes serovar 1/2a (strain ATCC BAA-679 / EGD-e) OX=169963 GN=lmo154
DECOY_tr|Q8YA10	Lmo0356 protein OS=Listeria monocytogenes serovar 1/2a (strain ATCC BAA-679 / EGD-e) OX=169963 GN=lmo0356 PE=4 S
tr|Q7AP65	OpuCA protein OS=Listeria monocytogenes serovar 1/2a (strain ATCC BAA-679 / EGD-e) OX=169963 GN=opuCA PE=4 SV=1
tr|Q8Y851	Lmo1067 protein OS=Listeria monocytogenes serovar 1/2a (strain ATCC BAA-679 / EGD-e) OX=169963 GN=lmo1067 PE=4 S
tr|Q8Y6I9	Phosphatidylglycerol lysyltransferase OS=Listeria monocytogenes serovar 1/2a (strain ATCC BAA-679 / EGD-e) OX=16
tr|Q8Y6J6	Lmo1688 protein OS=Listeria monocytogenes serovar 1/2a (strain ATCC BAA-679 / EGD-e) OX=169963 GN=lmo1688 PE=4 S
DECOY_tr|Q8Y8S2	Lmo0822 protein OS=Listeria monocytogenes serovar 1/2a (strain ATCC BAA-679 / EGD-e) OX=169963 GN=lmo0822 PE=4 S
DECOY_tr|Q8Y8S9	Lmo0815 protein OS=Listeria monocytogenes serovar 1/2a (strain ATCC BAA-679 / EGD-e) OX=169963 GN=lmo0815 PE=1 S
sp|Q8Y4A2	UDP-N-acetylglucosamine 1-carboxyvinyltransferase 2 OS=Listeria monocytogenes serovar 1/2a (strain ATCC BAA-679
sp|P67055	Demethylmenaquinone methyltransferase OS=Listeria monocytogenes serovar 1/2a (strain ATCC BAA-679 / EGD-e) OX=16
tr|Q8Y544	Lmo2232 protein OS=Listeria monocytogenes serovar 1/2a (strain ATCC BAA-679 / EGD-e) OX=169963 GN=lmo2232 PE=4 S
tr|Q8Y745	Oxygen-independent coproporphyrinogen-III oxidase-like protein OS=Listeria monocytogenes serovar 1/2a (strain AT
DECOY_tr|Q8Y4U7	Lmo2334 protein OS=Listeria monocytogenes serovar 1/2a (strain ATCC BAA-679 / EGD-e) OX=169963 GN=lmo2334 PE=4 S
sp|Q8Y5M0	Phospho-N-acetylmuramoyl-pentapeptide-transferase OS=Listeria monocytogenes serovar 1/2a (strain ATCC BAA-679 /
DECOY_tr|Q8Y859	Lmo1059 protein OS=Listeria monocytogenes serovar 1/2a (strain ATCC BAA-679 / EGD-e) OX=169963 GN=lmo1059 PE=4 S
sp|Q8Y6M4	Leucine--tRNA ligase OS=Listeria monocytogenes serovar 1/2a (strain ATCC BAA-679 / EGD-e) OX=169963 GN=leuS PE=3
DECOY_tr|Q8Y5I2	Lmo2080 protein OS=Listeria monocytogenes serovar 1/2a (strain ATCC BAA-679 / EGD-e) OX=169963 GN=lmo2080 PE=4 S
tr|Q8Y5A8	Lmo2160 protein OS=Listeria monocytogenes serovar 1/2a (strain ATCC BAA-679 / EGD-e) OX=169963 GN=lmo2160 PE=4 S

Select Summary Report

Format As Select Summary (protein hits) Help

Significance threshold p< 0.05 Max. number of hits AUTO

Standard scoring ○ MudPIT scoring ◉ Ions score or expect cut-off 17 Show sub-sets 0

Show pop-ups ◉ Suppress pop-ups ○ Require bold red ☑

Preferred taxonomy All entries

Re-Search ◉ All queries ○ Unassigned ○ Below homology threshold ○ Below identity threshold

1. sp|P02769 **Mass:** 71244 **Score:** 6961 **Matches:** 183(180) **Sequences:** 48(48) **emPAI:** 16.35
Serum albumin (Laboratory-Cont) OS=Bos taurus GN=ALB PE=1 SV=4

Query	Observed	Mr(expt)	Mr(calc)	ppm	Miss	Score	Expect	Rank	Unique	Peptide
448	379.714	757.414	757.416	-2.17	0	45	7.7e-05	1	U	K.GACLLPK.I 447
751	395.238	788.462	788.464	-3.48	0	48	4.7e-05	1	U	K.LVTDLTK.V 749 752 753 754 755 757
1431	424.252	846.490	846.496	-7.32	1	27	0.0047	1	U	R.LSQKFPK.A
1966	443.710	885.405	885.408	-3.30	0	57	2.9e-06	1	U	K.DDSPDLPK.L 1965 1967 1968
2171	449.743	897.470	897.474	-4.17	0	38	0.00041	1	U	R.LCVLHEK.T
2500	461.747	921.479	921.481	-2.10	0	49	3.6e-05	1	U	K.AEFVEVTK.L 2495 2496 2499 2501 2502
2565	464.250	926.485	926.486	-1.49	0	33	0.00099	1	U	K.YLYEIAR.R 2561 2564 2566 2567 2568 2571 2572 2574 2576 2578 2579 2580
3237	487.732	973.449	973.451	-1.72	0	44	6.5e-05	1	U	K.DLGEEHFK.G 3236 3238
3595	501.795	1001.575	1001.576	-0.84	0	33	0.002	1	U	K.LVVSTQTALA.- 3593 3596 3597
3741	507.810	1013.606	1013.612	-5.73	0	73	1.3e-07	1	U	K.QTALVELLK.H 3742 3743 3744 3745 3746 3747
4473	358.174	1071.502	1071.502	-0.30	0	53	7.9e-06	1	U	K.SHCIAEVEK.D 4474
4947	554.259	1106.503	1106.507	-3.43	0	67	4e-07	1	U	K.EACFAVEGPK.L
5365	569.751	1137.487	1137.491	-3.16	0	53	4.7e-06	1	U	K.CCTESLVNR.R
5413	571.859	1141.704	1141.707	-2.30	1	81	8.3e-09	1	U	K.KQTALVELLK.H 5412 5414 5415
5676	582.318	1162.622	1162.623	-1.01	0	70	3.7e-07	1	U	K.LVNELTEFAK.T 5657 5658 5659 5660 5661 5662 5663 5665 5666 5667 5668 5669 567
6870	417.212	1248.614	1248.614	0.43	1	51	2.5e-05	1	U	R.FKDLGEEHFK.G 6869
7320	642.356	1282.697	1282.703	-4.71	0	(33)	0.0014	1	U	R.HPEYAVSVLLR.L 7318
7321	428.574	1282.701	1282.703	-1.76	0	70	2.5e-07	1	U	R.HPEYAVSVLLR.L
7420	431.205	1290.593	1290.595	-1.07	0	58	3e-06	1	U	K.ECCDKPLLEK.S
7619	653.360	1304.706	1304.709	-2.27	0	(74)	1.3e-07	1	U	K.HLVDEPQNLIK.Q 7614 7615 7616 7617 7618 7620 7621 7622 7623 7624 7626 7627 76
7630	435.910	1304.709	1304.709	-0.16	0	81	1.7e-08	1	U	K.HLVDEPQNLIK.Q
9044	708.346	1414.678	1414.680	-1.94	0	94	1.2e-09	1	U	K.TVMENFVAFVDK.C 9045 9046 9047 9048 9050 9052
9092	473.902	1418.685	1418.686	-0.66	0	80	3e-08	1	U	K.SLHTLFGDELCK.V 9093
9094	710.350	1418.686	1418.686	-0.25	0	(62)	1.8e-06	1	U	K.SLHTLFGDELCK.V 9089
9307	480.610	1438.808	1438.804	2.27	1	91	1.8e-09	1	U	R.RHPEYAVSVLLR.L
9351	722.324	1442.633	1442.635	-0.87	0	84	3.6e-09	1	U	K.YICDNQDTISSK.L 9348 9349 9350
9352	481.885	1442.634	1442.635	-0.87	0	(43)	5.3e-05	1	U	K.YICDNQDTISSK.L
9535	488.534	1462.581	1462.582	-0.58	0	74	4.2e-08	1	U	K.TCVADESHAGCEK.S 9533
9701	740.402	1478.789	1478.788	0.49	0	121	2.2e-12	1	U	K.LGEYGFQNALIVR.Y 9688 9690 9694 9695 9696 9697 9698 9699 9700 9702
9703	493.937	1478.790	1478.788	1.28	0	(30)	0.0025	1	U	K.LGEYGFQNALIVR.Y
9843	747.760	1493.506	1493.511	-3.02	0	75	3.1e-08	1	U	R.ETYGDMADCCEK.Q
9938	751.809	1501.603	1501.606	-2.47	0	74	4.1e-08	1	U	K.EYEATLEECCAK.D 9937
10054	756.425	1510.835	1510.836	-0.57	0	106	4.3e-11	1	U	K.VPQVSTPTLVEVSR.S 10053
10321	511.597	1531.770	1531.774	-2.36	1	68	4.5e-07	1	U	K.LKECCDKPLLEK.S
10323	383.951	1531.774	1531.774	-0.02	1	(37)	0.00054	1	U	K.LKECCDKPLLEK.S
10569	518.890	1553.648	1553.646	1.51	0	63	4.7e-07	1	U	K.DDPHACYSTVFDK.L
10720	784.374	1566.733	1566.735	-1.66	0	85	5.3e-09	1	U	K.DAFLGSFLYEYSR.R 10721 10722 10723 10724 10726
10725	523.253	1566.736	1566.735	0.17	0	(26)	0.0045	1	U	K.DAFLGSFLYEYSR.R
10864	526.261	1575.760	1575.760	-0.08	0	78	4e-08	1	U	K.LKPDPNTLCDEFK.A 10863 10865
11703	820.469	1638.924	1638.930	-3.79	1	(53)	7e-06	1	U	R.KVPQVSTPTLVEVSR.S 11705
11709	547.317	1638.929	1638.930	-1.05	1	105	4.6e-11	1	U	R.KVPQVSTPTLVEVSR.S 11704 11706 11707 11708
11710	410.740	1638.931	1638.930	0.35	1	(80)	1.3e-08	1	U	R.KVPQVSTPTLVEVSR.S
12567	564.987	1691.938	1691.935	2.23	1	84	5.3e-09	1	U	K.AEFVEVTKLVTDLTK.V
13227	580.947	1739.819	1739.822	-1.61	0	61	1.9e-06	1	U	R.MPCTEDYLSLILNR.L
13228	870.917	1739.820	1739.822	-1.55	0	(41)	0.00021	1	U	R.MPCTEDYLSLILNR.L 13231
13291	874.355	1746.695	1746.698	-1.47	0	80	1.1e-08	1	U	K.YNGVFQECCQAEDK.G 13290
13308	583.891	1748.650	1748.655	-3.11	0	36	0.00023	1	U	K.ECCHGDLLECADDR.A
13881	599.288	1794.841	1794.825	9.34	1	65	6.7e-07	1	U	K.DDPHACYSTVFDKLK.H
14779	940.962	1879.910	1879.914	-2.26	0	60	2.7e-06	1	U	R.RPCFSALTPDETYVPK.A
14783	627.645	1879.913	1879.914	-0.43	0	(44)	0.00012	1	U	R.RPCFSALTPDETYVPK.A
15046	476.222	1900.859	1900.863	-1.74	1	101	8.7e-11	1	U	R.NECFLSHKDDSPDLPK.L
15103	636.643	1906.906	1906.914	-3.69	0	80	2.2e-08	1	U	K.LFTFHADICTLPDTEK.Q
15349	643.270	1926.789	1926.791	-1.29	1	111	7.1e-12	1	U	K.CCAADDKEACFAVEGPK.L 15346 15347 15348
15569	978.483	1954.951	1954.952	-0.85	0	79	3.4e-08	1	U	K.DAIPENLPPLTADFAEDK.D 15568
15905	505.748	2018.962	2018.962	0.12	1	80	2.2e-08	1	U	K.LKPDPNTLCDEFKADEK.K
9132	711.941	2132.802	2132.809	-2.96	1	65	2.9e-07	1	U	R.ETYGDMADCCEKQEPER.N
16849	562.742	2246.940	2246.935	2.11	1	62	6.2e-07	1	U	K.ECCHGDLLECADDRADLAK.Y
17946	615.300	2457.173	2457.173	-0.23	1	(31)	0.0014	1	U	K.DAIPENLPPLTADFAEDKDVCK.N
17948	820.065	2457.173	2457.173	-0.23	1	112	1.2e-11	1	U	K.DAIPENLPPLTADFAEDKDVCK.N 17947
18126	831.426	2491.256	2491.257	-0.44	0	81	1.8e-08	1	U	K.GLVLIAFSQYLQQCPFDEHVK.L

2. tr|Q8Y8K9 **Mass:** 31657 **Score:** 5006 **Matches:** 131(129) **Sequences:** 25(25) **emPAI:** 30.71
RsbR protein OS=Listeria monocytogenes serovar 1/2a (strain ATCC BAA-679 / EGD-e) OX=169963 GN=RsbR PE=4 SV=1

Query	Observed	Mr(expt)	Mr(calc)	ppm	Miss	Score	Expect	Rank	Unique	Peptide
453	379.729	757.443	757.445	-1.77	0	54	1.5e-05	1	U	R.ALALTNR.E 451 452
1197	419.202	836.389	836.392	-2.53	0	45	9.3e-05	1	U	K.LDDFAEK.V 1196 1198
1861	440.226	878.438	878.439	-0.81	0	53	2.2e-05	1	U	R.DEDLFLK.R 1858 1859 1860 1862 1863 1865 1866 1867
1912	441.726	881.438	881.440	-1.65	0	47	4.8e-05	1	U	K.DFANFIR.T 1904 1905 1907 1908 1909 1910 1913
3990	518.276	1034.537	1034.540	-2.74	1	70	3.4e-07	1	U	R.DEDLFLKR.E 3991 3992 3993
6806	622.827	1243.638	1243.641	-1.90	0	88	4e-09	1	U	K.QSDQLINDIAK.E 6797 6798 6799 6801 6803 6804 6805 6807 6808 6809
7301	641.818	1281.621	1281.624	-2.47	0	77	4.8e-08	1	U	K.VVTAYADTWEK.T 7299 7300
7349	643.351	1284.688	1284.690	-1.28	0	60	2.8e-06	1	U	R.VFGLLVYTAMR.D 7344 7346 7347 7348 7350 7351 7352 7353
7845	660.822	1319.629	1319.633	-2.94	1	(20)	0.027	1	U	-.MYKDFANFIR.T
7848	440.885	1319.633	1319.633	-0.33	1	60	2.5e-06	1	U	-.MYKDFANFIR.T 7847
7919	662.433	1322.852	1322.854	-1.44	0	103	4.9e-11	1	U	K.LIIENLLIGVVK.N 7906 7907 7908 7909 7910 7911 7912 7913 7914 7915 7916 7917 7

7922	441.958	1322.853	1322.854	-0.18	0	(46)	2.3e-05	1	U	K.LIIENLLIGVVK.N
8292	678.327	1354.640	1354.640	-0.49	1	86	6.3e-09	1	U	K.FNEKLDDFAEK.V 8293
8297	452.555	1354.642	1354.640	1.15	1	(73)	1.3e-07	1	U	K.FNEKLDDFAEK.V 8295 8296
8396	681.828	1361.642	1361.644	-1.48	1	(54)	6.9e-06	1	U	-.MYKDFANFIR.T 8395
9024	472.208	1413.602	1413.605	-1.88	0	(44)	3.7e-05	1	U	R.EEKPEDDAYYR.F 9026
9025	707.809	1413.603	1413.605	-1.39	0	99	1.4e-10	1	U	R.EEKPEDDAYYR.F
9115	711.324	1420.632	1420.633	-0.56	0	47	3e-05	1	U	R.FETWLSSMYNK.V 9114
10142	508.336	1521.988	1521.986	1.19	1	59	1.2e-06	1	U	R.AKLIIENLLIGVVK.N
10655	780.918	1559.822	1559.823	-0.34	0	105	9.9e-11	1	U	K.ISVMPLIGTIDTER.A 10645 10649 10651 10652 10653 10654 10656 10657 10658 1065
10765	524.242	1569.704	1569.706	-1.29	1	(30)	0.001	1	U	K.REEKPEDDAYYR.F
10767	393.434	1569.706	1569.706	0.12	1	57	2.2e-06	1	U	K.REEKPEDDAYYR.F
11711	820.849	1639.683	1639.686	-1.97	0	71	8.7e-08	1	U	K.ADLLNDWMNEMEK.Q 11712
11713	547.569	1639.685	1639.686	-0.55	0	(54)	4.3e-06	1	U	K.ADLLNDWMNEMEK.Q
13332	875.946	1749.877	1749.878	-1.10	0	96	8.2e-10	1	U	K.EFVDLIVSNVTENGSK.F 13333 13334 13335
13336	584.300	1749.878	1749.878	-0.25	0	(53)	1.7e-05	1	U	K.EFVDLIVSNVTENGSK.F 5719 13331 13338
13400	586.001	1754.981	1754.982	-0.22	0	(92)	8e-10	1	U	K.SALQELSAPLLPIFEK.I 13394 13397 13398
13402	878.498	1754.982	1754.982	0.19	0	96	4.1e-10	1	U	K.SALQELSAPLLPIFEK.I 13391 13392 13393 13396 13399 13401 13403 13404
15730	661.965	1982.873	1982.871	0.89	1	40	0.0001	1	U	R.TNKADLLNDWMNEMEK.Q
16330	716.047	2145.121	2145.118	1.21	1	30	0.0029	1	U	R.VFGLLVYTAMRDEDLFLK.R 16331
17705	804.794	2411.362	2411.368	-2.45	1	54	3.6e-06	1	U	K.TVSIQKSALQELSAPLLPIFEK.I
19536	709.585	2834.311	2834.317	-1.94	1	(42)	0.0001	1	U	K.EAMYEETSKEFVDLIVSNVTENGSK.F
19538	945.778	2834.312	2834.317	-1.62	1	102	1.1e-10	1	U	K.EAMYEETSKEFVDLIVSNVTENGSK.F 19537
19594	956.112	2865.313	2865.316	-1.01	1	29	0.0018	1	U	K.ADLLNDWMNEMEKQSDQLINDIAK.E 19593
19987	804.435	3213.712	3213.707	1.73	0	67	2.5e-07	1	U	R.SEVVLIDITGVPVVDTMVAHHIIQASEAVR.L 19986

3. sp|P04264 **Mass:** 66170 **Score:** 1880 **Matches:** 46(45) **Sequences:** 28(28) **emPAI:** 5.00
Keratin, type II cytoskeletal 1 (Contact-Cont) OS=Homo sapiens GN=KRT1 PE=1 SV=6

Query	Observed	Mr(expt)	Mr(calc)	ppm	Miss	Score	Expect	Rank	Unique	Peptide
32	352.694	703.373	703.375	-3.34	0	22	0.032	1	U	R.LDSELK.N
1800	437.753	873.491	873.492	-1.29	0	55	1.7e-05	1	U	R.SLVNLGGSK.S 1799
3225	487.268	972.522	972.524	-1.88	0	67	8.4e-07	1		K.IEISELNR.V 3221 3223 3224 3226 3228
3749	508.222	1014.429	1014.433	-3.32	0	50	1e-05	1	U	K.DVDGAYMTK.V
3965	517.261	1032.507	1032.509	-1.92	0	56	9e-06	1	U	R.TLLEGEESR.M 3964
4389	533.262	1064.509	1064.514	-4.21	0	59	5.2e-06	1	U	K.AQYEDIAQK.S
4402	356.176	1065.505	1065.509	-3.49	1	32	0.002	1		K.YEDEINKR.T
4497	358.535	1072.583	1072.588	-4.14	1	37	0.00074	1	U	R.LRSEIDNVK.K
5202	563.273	1124.531	1124.535	-3.24	0	72	2.2e-07	1	U	K.AEAESLYQSK.Y
5582	579.259	1156.503	1156.507	-3.14	0	57	2.9e-06	1	U	R.DYQELMNTK.L
7084	633.321	1264.627	1264.630	-2.66	0	81	2.5e-08	1	U	R.TNAENEFVTIK.K 7083
7251	639.358	1276.701	1276.703	-1.39	0	55	8.3e-06	1	U	K.LALDLEIATYR.T
7569	651.860	1301.704	1301.708	-2.56	0	83	2.2e-08	1	U	R.SLDLDSIIAEVK.A 7568 7570
8017	666.761	1331.508	1331.512	-3.34	0	61	8.5e-07	1	U	K.NMQDMVEDYR.N 8018
8115	447.560	1339.657	1339.662	-3.33	1	67	7.4e-07	1	U	K.SKAEAESLYQSK.Y
8116	670.836	1339.658	1339.662	-2.63	1	(57)	6.8e-06	1	U	K.SKAEAESLYQSK.Y
8333	679.351	1356.688	1356.688	-0.38	0	86	7.5e-09	1	U	K.LNDLEDALQQAK.E 8332
8652	692.349	1382.683	1382.683	-0.31	0	74	1.1e-07	1	U	K.SLNNQFASFIDK.V 8653
8819	465.249	1392.725	1392.725	-0.04	1	88	5.8e-09	1	U	R.TNAENEFVTIKK.D
9648	738.378	1474.741	1474.742	-0.75	0	74	1e-07	1	U	K.WELLQQVDTSTR.T
9655	738.396	1474.778	1474.778	-0.01	0	76	8.1e-08	1		R.FLEQQNQVLQTK.W 9652 9653 9654
9656	492.601	1474.781	1474.778	1.88	0	(32)	0.0017	1		R.FLEQQNQVLQTK.W
11123	800.420	1598.826	1598.826	-0.33	1	99	3.4e-10	1	U	K.NKLNDLEDALQQAK.E
11665	546.962	1637.864	1637.853	6.79	1	52	1.7e-05	1	U	K.SLNNQFASFIDKVR.F
12957	858.928	1715.842	1715.844	-0.88	0	130	3.1e-13	1	U	K.QISNLQQSISDAEQR.G
13580	883.377	1764.739	1764.727	6.60	0	157	2.1e-16	1	U	R.FSSCGGGGGSFGAGGGFGSR.S
15449	648.002	1940.984	1940.980	1.82	1	57	4.2e-06	1	U	K.LNDLEDALQQAKEDLAR.L
15896	673.295	2016.864	2016.877	-6.31	1	32	0.00064	1	U	R.LDSELKNMQDMVEDYR.N
17365	777.060	2328.157	2328.167	-4.05	1	22	0.016	1	U	K.QISNLQQSISDAEQRGENALK.D
17589	795.322	2382.943	2382.945	-0.74	0	68	1.5e-07	1	U	R.GGGGGGYGSGGSSYGSGGGSYGSGGGGGGGR.G

4. sp|P13645 **Mass:** 59020 **Score:** 1651 **Matches:** 34(34) **Sequences:** 24(24) **emPAI:** 4.58
Keratin, type I cytoskeletal 10 (Contact-Cont) OS=Homo sapiens GN=KRT10 PE=1 SV=6

Query	Observed	Mr(expt)	Mr(calc)	ppm	Miss	Score	Expect	Rank	Unique	Peptide
897	404.203	806.391	806.392	-1.82	0	45	0.00011	1		R.LAADDFR.L 896 898 900
3477	497.256	992.498	992.493	5.49	0	52	2e-05	1	U	K.YENEVALR.Q
3504	498.266	994.518	994.512	6.00	1	46	9.4e-05	1	U	K.IKEWYEK.H
3947	516.302	1030.590	1030.591	-1.10	0	53	1.9e-05	1		R.VLDELTLTK.A 3946 3948
4383	532.808	1063.602	1063.603	-0.92	1	39	0.00021	1	U	R.LASYLDKVR.A
4933	553.765	1105.516	1105.519	-2.49	0	84	9.4e-09	1	U	K.VTMQNLNDR.L
4977	555.248	1108.482	1108.483	-0.51	0	31	0.0012	1	U	K.DAEAWFNEK.S
5702	583.296	1164.577	1164.577	-0.72	0	72	1.9e-07	1	U	R.LENEIQTYR.S
6167	601.311	1200.607	1200.610	-2.23	0	74	1.4e-07	1		R.QSVEADINGLR.R
7033	631.800	1261.585	1261.590	-3.85	0	72	1.6e-07	1	U	R.SLLEGEGSSGGGGR.G
8338	453.244	1356.711	1356.711	-0.16	1	54	1.3e-05	1		R.QSVEADINGLRR.V
8436	683.323	1364.631	1364.632	-0.73	0	63	1.2e-06	1	U	R.SQYEQLAEQNR.K
8624	691.326	1380.637	1380.641	-2.98	0	97	4.6e-10	1	U	R.ALEESNYELEGK.I 8623 8625
8761	695.843	1389.671	1389.674	-2.18	0	104	1.1e-10	1	U	K.QSLEASLAETEGR.Y 8760
9238	717.889	1433.763	1433.763	-0.09	1	52	1.7e-05	1	U	K.IRLENEIQTYR.S
9835	747.368	1492.721	1492.727	-3.94	1	79	4.4e-08	1	U	R.SQYEQLAEQNRK.D
12811	854.391	1706.767	1706.765	1.42	0	163	1.9e-16	1	U	K.GSLGGGFSSGGFSGGSFSR.G
13896	599.675	1796.003	1796.004	-0.87	0	21	0.011	1	U	R.NVQALEIELQSQLALK.Q
15798	666.329	1995.966	1995.964	0.98	0	(79)	2.5e-08	1	U	K.ELTTEIDNNIEQISSYK.S
15799	998.993	1995.971	1995.964	3.44	0	104	8.1e-11	1	U	K.ELTTEIDNNIEQISSYK.S
16115	1041.985	2081.956	2081.958	-0.70	0	124	7.2e-13	1	U	R.AETECQNTEYQQLLDIK.I
16661	738.038	2211.092	2211.091	0.63	1	102	8e-11	1	U	K.SKELTTEIDNNIEQISSYK.S 16660

17420	1172.011	2342.008	2341.977	13.3	0	88	1.5e-09	1	U	R.GSSGGGCFGGSSGGYGGLGGFGGGSFR.G
17499	789.744	2366.209	2366.255	-19.39	0	30	0.0029	1	U	K.NQILNLTTDNANILLQIDNAR.L
15412	968.802	2903.385	2903.375	3.29	0	86	5.9e-09	1	U	R.NVSTGDVNVEMNAAPGVDLTQLLNNMR.S

---

5. sp|P35908 **Mass:** 65678 **Score:** 1271 **Matches:** 40(39) **Sequences:** 25(25) **emPAI:** 4.01
Keratin, type II cytoskeletal 2 epidermal (Contact-Cont) OS=Homo sapiens GN=KRT2 PE=1 SV=2

Query	Observed	Mr(expt)	Mr(calc)	ppm	Miss	Score	Expect	Rank	Unique	Peptide
1081	414.218	826.422	826.423	-0.96	0	41	0.0002	1	U	K.FASFIDK.V
1127	416.249	830.483	830.486	-3.43	0	40	0.00037	1	U	R.SLVGLGGTK.S 1126
3225	487.268	972.522	972.524	-1.88	0	67	8.4e-07	1		K.IEISELNR.V 3221 3223 3224 3226 3228
3641	503.235	1004.456	1004.460	-3.97	0	31	0.0015	1	U	K.LLEGEECR.M
4013	519.266	1036.518	1036.519	-0.79	0	46	8.9e-05	1	U	R.YLDGLTAER.T
4070	521.279	1040.542	1040.550	-7.43	0	39	0.00028	1	U	K.VDPEIQNVK.A
4402	356.176	1065.505	1065.509	-3.49	1	32	0.002	1		K.YEDEINKR.T
4652	542.754	1083.493	1083.491	2.51	0	45	4.2e-05	1	U	K.DVDNAYMIK.V
4952	554.273	1106.531	1106.536	-4.17	0	38	0.00048	1	U	K.AQYEEIAQR.S 4951
5282	566.256	1130.498	1130.503	-3.89	0	50	1e-05	1	U	R.STSSFSCLSR.H
5556	578.271	1154.528	1154.528	0.25	0	46	4e-05	1	U	R.DYQELMNVK.L 5557
6064	597.311	1192.608	1192.609	-0.64	0	60	5.2e-06	1	U	K.YEELQVTVGR.H
6111	599.276	1196.537	1196.542	-4.76	0	59	4e-06	1	U	K.GGSISGGGYGSGGGK.H
6300	604.810	1207.606	1207.608	-2.34	0	83	2.3e-08	1	U	R.TAAENDFVTLK.K
6946	627.807	1253.599	1253.600	-0.96	0	41	0.00034	1	U	R.GFSSGSAVVSGGSR.R
7836	440.866	1319.577	1319.576	1.20	0	54	4.5e-06	1	U	R.HGGGGGGFGGGGFGSR.S
7985	665.325	1328.636	1328.632	2.87	0	68	9.8e-07	1	U	K.NVQDAIADAEQR.G
7995	665.365	1328.716	1328.719	-2.42	0	81	2.9e-08	1	U	R.NLDLDSIIAEVK.A 7993 7994
8076	668.853	1335.692	1335.703	-8.44	1	56	6.4e-06	1	U	R.TAAENDFVTLKK.D
8501	686.358	1370.702	1370.704	-1.35	0	41	0.00023	1	U	K.LNDLEEALQQAK.E
8772	464.564	1390.671	1390.673	-1.14	1	59	3.1e-06	1	U	R.SKEEAEALYHSK.Y
9523	730.903	1459.791	1459.792	-0.76	0	84	1.6e-08	1	U	K.VDLLNQEIEFLK.V 9522
9655	738.396	1474.778	1474.778	-0.01	0	76	8.1e-08	1		R.FLEQQNQVLQTK.W 9652 9653 9654
9656	492.601	1474.781	1474.778	1.88	0	(32)	0.0017	1		R.FLEQQNQVLQTK.W
13237	871.378	1740.742	1740.741	0.40	0	100	9.4e-11	1	U	R.GGSGGGGSISGGGYGSGGGSGGR.Y
10320	766.878	3063.482	3063.482	0.15	0	22	0.025	1	U	K.VLYDAEISQIHQSVTDTNVILSMDNSR.N

---

6. sp|P00761 **Mass:** 25078 **Score:** 1066 **Matches:** 37(36) **Sequences:** 2(2) **emPAI:** 0.40
Trypsin (Laboratory-Cont) OS=Sus scrofa PE=1 SV=1

Query	Observed	Mr(expt)	Mr(calc)	ppm	Miss	Score	Expect	Rank	Unique	Peptide
1369	421.758	841.502	841.502	-0.74	0	57	4.7e-06	1	U	R.VATVSLPR.S 1342 1344 1345 1346 1347 1348 1349 1350 1351 1352 1353 1354 1355 1
4133	523.284	1044.554	1044.556	-1.90	0	47	7.9e-05	1	U	K.LSSPATLNSR.V 4125 4126 4130 4131

---

7. tr|Q8Y863 **Mass:** 58159 **Score:** 950 **Matches:** 23(23) **Sequences:** 16(16) **emPAI:** 2.20
Dihydrolipoamide acetyltransferase component of pyruvate dehydrogenase complex OS=Listeria monocytogenes serovar 1/2a (strain ATCC BAA-679 / EGD-e)

Query	Observed	Mr(expt)	Mr(calc)	ppm	Miss	Score	Expect	Rank	Unique	Peptide
334	370.702	739.390	739.390	-0.14	0	38	0.00027	1	U	K.GIFEFK.L
353	372.241	742.467	742.470	-3.93	0	43	0.00015	1	U	K.ALVATLR.D
4879	551.298	1100.581	1100.583	-1.83	0	72	2.4e-07	1	U	K.GVNIAEVAGSGK.N 4878
8353	453.580	1357.717	1357.720	-2.36	1	53	1.8e-05	1	U	R.EKGVNIAEVAGSGK.N
8681	692.865	1383.715	1383.718	-2.45	0	54	1.2e-05	1	U	K.DPNGLVIAMPSVR.K 8682
9030	707.877	1413.739	1413.742	-2.51	0	56	7.7e-06	1	U	R.LLNDPELLLMEV.- 9031
9099	710.837	1419.659	1419.663	-2.85	0	67	3.4e-07	1	U	K.QPVASSDAYPETR.E
10061	504.944	1511.810	1511.813	-2.20	1	63	1.5e-06	1	U	K.KDPNGLVIAMPSVR.K 10059
10314	766.365	1530.715	1530.716	-0.83	0	89	2.6e-09	1	U	K.AESTESTPAPAQASGK.G 10313
10772	785.913	1569.812	1569.844	-20.43	1	38	0.00047	1	U	K.RLLNDPELLLMEV.-
11183	535.954	1604.839	1604.841	-1.34	0	82	2.1e-08	1	U	K.LPDIGEGIHEGEIVK.W 11182
11808	825.422	1648.829	1648.831	-1.31	0	88	7.4e-09	1	U	K.SVFQISDEINELAGK.A
13297	583.291	1746.851	1746.854	-1.54	1	89	4e-09	1	U	K.AAGKQPVASSDAYPETR.E
15860	1005.525	2009.036	2009.058	-10.96	0	35	0.00059	1	U	K.DGEIVAAPVLALSLSFDHR.V
16467	728.363	2182.069	2182.068	0.17	0	54	1e-05	1	U	R.DFPVLNTTLDDATEELVYK.H
18125	831.397	2491.170	2491.171	-0.42	0	110	1.6e-11	1	U	K.ADIDAFLNGEQPAAATTTAQTEEK.A 18124

---

8. sp|P58724 **Mass:** 28912 **Score:** 861 **Matches:** 18(18) **Sequences:** 11(11) **emPAI:** 3.96
Blue-light photoreceptor OS=Listeria monocytogenes serovar 1/2a (strain ATCC BAA-679 / EGD-e) OX=169963 GN=lmo0799 PE=3 SV=1

Query	Observed	Mr(expt)	Mr(calc)	ppm	Miss	Score	Expect	Rank	Unique	Peptide
558	385.689	769.364	769.365	-1.11	0	20	0.014	1	U	K.EALQFY.-
997	410.225	818.436	818.439	-3.68	0	49	7.4e-05	1	U	K.SLTEIEK.L 996
1710	434.241	866.467	866.468	-2.00	0	44	0.00011	1	U	K.FAMTLIR.Y
3141	484.313	966.611	966.611	-0.69	0	52	6.4e-06	1	U	K.LSTPIVPIK.E 3140
7476	647.853	1293.692	1293.697	-3.60	0	67	6.3e-07	1	U	M.TAYPQFDVILK.A 7475
9318	720.936	1439.857	1439.860	-1.89	0	99	1.2e-10	1	U	K.LTGVELIITGISPK.F 9317
12981	860.407	1718.799	1718.800	-0.47	0	(29)	0.0026	1	U	K.DVTTEHDYQLELEK.S
12982	573.940	1718.799	1718.800	-0.32	0	87	3.9e-09	1	U	K.DVTTEHDYQLELEK.S
13137	866.935	1731.856	1731.857	-0.58	0	88	6.4e-09	1	U	R.YEENLASLTTYSTIK.E
13777	892.493	1782.971	1782.973	-0.81	0	116	4.6e-12	1	U	K.ALNLSSVGVIITDPEQK.D 13776
13778	595.331	1782.972	1782.973	-0.56	0	(40)	0.00018	1	U	K.ALNLSSVGVIITDPEQK.D
16222	1057.033	2112.051	2112.053	-0.99	0	92	2.1e-09	1	U	K.DNPIIFVNTGFENITGYAK.E
18384	864.723	2591.148	2591.145	1.49	1	92	6.2e-10	1	U	K.EEALGSNCHFLQGDDTDKEEVAK.I

---

9. sp|P35527 **Mass:** 62255 **Score:** 831 **Matches:** 16(16) **Sequences:** 13(13) **emPAI:** 1.42
Keratin, type I cytoskeletal 9 (Contact-Cont) OS=Homo sapiens GN=KRT9 PE=1 SV=3

Query	Observed	Mr(expt)	Mr(calc)	ppm	Miss	Score	Expect	Rank	Unique	Peptide
367	373.215	744.415	744.413	2.33	0	31	0.0043	1	U	K.EVTQLR.H
2365	457.207	912.399	912.401	-2.10	0	36	0.00024	1	U	R.MTLDDFR.I 2366

4311	530.784	1059.554	1059.556	-1.49	0	70	4.2e-07	1	U	K.TLLDIDNTR.M 4312
5592	579.299	1156.583	1156.584	-0.54	0	79	3.6e-08	1	U	R.QGVDADINGLR.Q 5591
6258	603.803	1205.592	1205.596	-3.82	0	71	3.6e-07	1	U	R.QVLDNLTMEK.S
6617	616.799	1231.584	1231.591	-5.48	0	112	1.1e-11	1	U	R.SGGGGGGGLGSGGSIR.S
6692	618.265	1234.515	1234.521	-4.85	0	74	4e-08	1	U	R.FSSSSGYGGGSSR.V
7900	441.897	1322.668	1322.665	2.00	1	59	4.7e-06	1	U	R.IKFEMEQNLR.Q
10969	793.885	1585.756	1585.758	-1.50	0	96	4.5e-10	1	U	K.VQALEEANNDLENK.I
13830	896.361	1790.708	1790.720	-6.91	0	106	2.4e-11	1	U	R.GGSGGSYGGGGSGGGYGGGSGSR.G
14616	934.470	1866.925	1866.915	5.51	1	24	0.015	1	U	K.TLNDMRQEYEQLIAK.N
17562	793.064	2376.171	2376.181	-4.09	1	25	0.0081	1	U	R.LASYLDKVQALEEANNDLENK.I
18177	837.386	2509.137	2509.124	5.04	0	163	6.4e-17	1	U	K.EIETYHNLLEGGQEDFESSGAGK.I

10. sp|Q8Y822 **Mass:** 58363 **Score:** 772 **Matches:** 22(22) **Sequences:** 19(19) **emPAI:** 2.96
GMP synthase [glutamine-hydrolyzing] OS=Listeria monocytogenes serovar 1/2a (strain ATCC BAA-679 / EGD-e) OX=169963 GN=guaA PE=3 SV=1

Query	Observed	Mr(expt)	Mr(calc)	ppm	Miss	Score	Expect	Rank	Unique	Peptide
1880	441.221	880.428	880.429	-0.82	0	54	4.2e-05	1	U	R.DSDYILR.E
3774	509.269	1016.524	1016.539	-14.82	0	53	2.7e-05	1	U	R.VLGEITEEK.L
3907	515.300	1028.586	1028.587	-1.02	1	43	0.00019	1	U	K.IQEIVGDKK.V
4091	522.269	1042.524	1042.529	-5.27	0	73	8.7e-08	1	U	K.LAGVSDPEQK.R
4712	545.280	1088.546	1088.561	-14.43	0	34	0.00096	1	U	R.TYDHTVVVR.A
5603	386.873	1157.598	1157.604	-5.10	1	51	2e-05	1	U	R.EEIKNAGLER.E
5624	580.306	1158.597	1158.603	-5.31	0	51	3.6e-05	1	U	R.HSVYGNELLK.N 5625
5860	589.807	1177.599	1177.620	-17.81	0	43	0.00019	1	U	K.ADIHVEKPNR.L
5995	594.353	1186.692	1186.696	-3.60	0	40	0.00017	1	U	K.LIEPLNTLFK.D 5996
6077	398.879	1193.614	1193.615	-1.12	0	69	5.4e-07	1	U	R.IVNEVDHVNR.V
7792	659.304	1316.594	1316.603	-6.95	0	69	1.7e-07	1	U	K.SCPIAGIADEER.S
8610	690.853	1379.692	1379.693	-0.81	1	60	2.2e-06	1	U	R.DSDYILREEIK.N
9201	716.368	1430.722	1430.731	-6.07	0	73	1.5e-07	1	U	R.SLYGVQFHPEVR.H 9200
11387	543.313	1626.917	1626.919	-1.16	1	72	9.3e-08	1	U	R.VLGEITEEKLEIVR.D
11751	822.926	1643.838	1643.834	2.60	0	63	1.7e-06	1	U	R.ALGTELGMPDAIVWR.Q
12473	562.985	1685.934	1685.935	-0.54	1	70	2.1e-07	1	U	K.LIEPLNTLFKDEVR.A
6942	627.351	1879.031	1879.020	5.73	0	48	6.5e-05	1	U	K.IIVLDFGSQYNQLITR.R
17794	809.725	2426.154	2426.146	3.26	1	42	0.00012	1	U	R.KGEADQVMETLQGEFNMNIIK.V
17865	1220.603	2439.191	2439.171	8.23	0	39	0.00054	1	U	K.ALNPTGIIFSGGPNSVYDEDAFR.A

11. tr|Q8Y4U6 **Mass:** 66230 **Score:** 700 **Matches:** 15(15) **Sequences:** 12(12) **emPAI:** 1.15
FruA protein OS=Listeria monocytogenes serovar 1/2a (strain ATCC BAA-679 / EGD-e) OX=169963 GN=fruA PE=4 SV=1

Query	Observed	Mr(expt)	Mr(calc)	ppm	Miss	Score	Expect	Rank	Unique	Peptide
758	395.239	788.463	788.464	-1.15	0	44	0.00013	1	U	R.ITDLLSK.D
3285	489.269	976.524	976.523	0.92	0	44	0.00031	1	U	K.INDEVLFK.K
4928	553.312	1104.609	1104.618	-7.90	1	32	0.0019	1	U	K.INDEVLFKK.A
5644	581.805	1161.596	1161.603	-5.70	0	63	2e-06	1	U	K.EAISGNAPIYK.A
5808	587.817	1173.619	1173.639	-17.71	0	36	0.0013	1	U	K.AVNEPTVVFAK.S 5810
7808	659.374	1316.733	1316.734	-0.95	0	76	5e-08	1	U	K.LGSYNEIPALLK.Q 7807 7809
8472	685.334	1368.654	1368.663	-6.21	0	62	1.5e-06	1	U	K.DVMIMSLQATTK.E
8598	460.608	1378.803	1378.809	-3.79	0	83	5e-09	1	U	R.LLVHPAFVQSLR.D
13787	595.953	1784.836	1784.836	-0.29	0	97	5e-10	1	U	R.EAQSSTGVGEGIAMPHAK.T
16520	731.695	2192.062	2192.061	0.73	0	53	1.3e-05	1	U	K.SVVAVTACPTGIAHTYMAAEK.L
17084	761.363	2281.067	2281.071	-1.60	0	82	1.1e-08	1	U	K.AEEGSQATESADGLSIGQQIYK.H
19366	930.160	2787.457	2787.444	4.56	0	129	1.8e-13	1	U	R.DAGLTNSILGASFITEGAIPFAAADPLR.M

12. tr|Q8Y435 **Mass:** 70459 **Score:** 629 **Matches:** 14(14) **Sequences:** 13(13) **emPAI:** 1.18
Lmo2638 protein OS=Listeria monocytogenes serovar 1/2a (strain ATCC BAA-679 / EGD-e) OX=169963 GN=lmo2638 PE=4 SV=1

Query	Observed	Mr(expt)	Mr(calc)	ppm	Miss	Score	Expect	Rank	Unique	Peptide
109	353.693	705.371	705.373	-2.84	0	28	0.0067	1	U	K.GIEIMK.N
602	388.213	774.411	774.412	-1.39	0	24	0.023	1	U	K.IDASEIK.L
4291	529.795	1057.575	1057.577	-2.12	0	35	0.001	1	U	K.DVNITLIDR.H
4450	535.778	1069.542	1069.540	1.32	0	69	5.1e-07	1	U	R.NHIEETVTK.A
5302	566.794	1131.573	1131.577	-3.98	1	56	1.1e-05	1	U	K.DNKIDASEIK.L
7103	633.812	1265.609	1265.617	-6.28	0	69	2.8e-07	1	U	K.SIIVEMSGTGEK.E
7562	434.575	1300.704	1300.699	4.15	1	47	7.1e-05	1	U	K.DKDVNITLIDR.H
7596	652.847	1303.679	1303.677	1.54	0	68	6.2e-07	1	U	R.VEPTAIQYDLR.R
8000	665.386	1328.757	1328.760	-2.59	0	69	2.2e-07	1	U	R.YGNVLWVLPLR.V
10643	520.892	1559.654	1559.652	1.01	1	103	4.7e-11	1	U	R.ANSDTKDYGMESAR.A
11231	537.321	1608.940	1608.945	-3.10	0	63	5.5e-07	1	U	K.NAAIVEVKPESIVLK.S
13779	892.500	1782.986	1782.991	-3.26	0	(34)	0.00071	1	U	K.LVVVEAAPTILNMLER.R
13780	595.336	1782.986	1782.991	-2.73	0	92	1.1e-09	1	U	K.LVVVEAAPTILNMLER.R
14072	908.971	1815.927	1815.937	-5.10	0	90	2.6e-09	1	U	K.SGEELPTSTLIWTAGVR.A

13. tr|Q8YAG5 **Mass:** 31945 **Score:** 628 **Matches:** 15(15) **Sequences:** 12(12) **emPAI:** 3.85
Lmo0161 protein OS=Listeria monocytogenes serovar 1/2a (strain ATCC BAA-679 / EGD-e) OX=169963 GN=lmo0161 PE=4 SV=1

Query	Observed	Mr(expt)	Mr(calc)	ppm	Miss	Score	Expect	Rank	Unique	Peptide
1809	438.243	874.471	874.476	-5.24	0	65	1.6e-06	1	U	K.LSEITANK.C
2613	465.751	929.488	929.493	-5.14	0	60	3.3e-06	1	U	K.AVLQDQTR.T
3557	500.303	998.592	998.595	-2.25	0	52	1.8e-05	1	U	K.LAILPLMGR.V 3556 3558
9611	736.428	1470.841	1470.845	-2.17	0	54	6.5e-06	1	U	K.LLGVEAFISGIQPK.M
10828	788.340	1574.666	1574.670	-2.60	0	79	1.3e-08	1	U	-.MNESNGSMELYLR.E
12011	829.465	1656.916	1656.919	-1.82	0	82	1.4e-08	1	U	R.LIEEISTPVISITDK.L 12010
13857	897.422	1792.829	1792.834	-3.09	0	72	1.5e-07	1	U	R.INEAFSAMYYNDIVK.H
6470	610.307	1827.898	1827.889	5.02	0	35	0.0028	1	U	R.ADSEQTADLIISYFAGK.K
14309	922.469	1842.923	1842.928	-2.90	0	25	0.0088	1	U	R.MENEVPLPEVITTLDK.L
15814	667.349	1999.026	1999.030	-1.75	1	58	4.1e-06	1	U	R.RMENEVPLPEVITTLDK.L

15830	668.315	2001.924	2001.936	-5.80	0	42	0.00012	1	U	K.IYENTYTSFVYSPQYK.D
18199	839.403	2515.187	2515.191	-1.31	1	127	5e-13	1	U	K.IYENTYTSFVYSPQYKDELR.A

14. sp|Q48754 **Mass:** 38448 **Score:** 565 **Matches:** 13(13) **Sequences:** 12(12) **emPAI:** 2.73
CD4+ T-cell-stimulating antigen OS=Listeria monocytogenes serovar 1/2a (strain ATCC BAA-679 / EGD-e) OX=169963 GN=tcsA PE=3 SV=2

Query	Observed	Mr(expt)	Mr(calc)	ppm	Miss	Score	Expect	Rank	Unique	Peptide
459	380.215	758.416	758.417	-2.12	0	33	0.0031	1	U	K.IVDGDIK.V
477	381.727	761.439	761.444	-5.46	0	17	0.055	1	U	K.VGFVGGVK.G
1443	425.208	848.401	848.403	-2.43	0	54	3.4e-05	1	U	K.AGDFPGGTK.I
2004	444.741	887.468	887.482	-16.07	0	71	4.4e-07	1	U	K.TNLNTAVR.S
3296	489.764	977.514	977.518	-4.09	0	38	0.0013	1	U	R.DNVVSIGFK.D
6176	601.317	1200.620	1200.635	-12.31	0	69	5.3e-07	1	U	R.VDIAVEDLATR.A
7695	437.250	1308.728	1308.729	-0.54	1	59	1.3e-06	1	U	K.IVDGDIKVPEKP.- 7696
8342	679.375	1356.735	1356.736	-1.02	1	69	5e-07	1	U	K.RVDIAVEDLATR.A
8941	703.845	1405.675	1405.677	-1.10	0	66	7.1e-07	1	U	R.SDYDLIYGIGYK.L
12924	857.886	1713.757	1713.752	3.38	0	98	1.7e-10	1	U	K.DFTVAMVTDTGGVDDR.S
14787	941.410	1880.806	1880.806	-0.04	0	77	1.9e-08	1	U	K.GTDGYNYLQSASEADYK.T
15692	989.486	1976.957	1976.959	-0.89	0	95	7.6e-10	1	U	K.AVNPNAQIDVQYANDFAK.A

15. sp|Q8Y6M6 **Mass:** 28414 **Score:** 564 **Matches:** 16(15) **Sequences:** 10(9) **emPAI:** 2.78
30S ribosomal protein S2 OS=Listeria monocytogenes serovar 1/2a (strain ATCC BAA-679 / EGD-e) OX=169963 GN=rpsB PE=3 SV=1

Query	Observed	Mr(expt)	Mr(calc)	ppm	Miss	Score	Expect	Rank	Unique	Peptide
275	365.214	728.414	728.418	-6.00	0	58	5.1e-06	1	U	R.IAVAEAR.K
1090	414.715	827.416	827.418	-2.26	0	32	0.00096	1	U	K.YIFTER.N
4949	554.271	1106.528	1106.526	2.21	0	19	0.092	1	U	R.SGQYFVNHR.W
5831	588.834	1175.654	1175.655	-1.25	0	33	0.0017	1	U	R.NGIYIIDLQK.T
7177	424.872	1271.594	1271.597	-2.60	1	37	0.00034	1	U	K.KVDEAFNFMR.E
7737	656.865	1311.716	1311.719	-2.41	0	66	4.2e-07	1	U	K.GLPDALFIVDPR.K 7734 7735 7736
8259	676.822	1351.630	1351.633	-2.44	0	95	7.4e-10	1	U	R.MEADGTFEVLPK.K 8258
8899	701.355	1400.695	1400.701	-3.95	1	35	0.0013	1	U	K.QAQESVRDEAIR.S
8901	467.909	1400.704	1400.701	2.22	1	(28)	0.0052	1	U	K.QAQESVRDEAIR.S
10527	775.905	1549.796	1549.799	-1.71	0	87	5.4e-09	1	U	R.EVASDNGTILFVGTK.K 10526
11208	804.424	1606.834	1606.836	-0.92	0	66	9.6e-07	1	U	R.WLGGTLTNFETIQK.R

16. tr|Q8Y6P2 **Mass:** 30453 **Score:** 546 **Matches:** 16(15) **Sequences:** 11(11) **emPAI:** 3.57
Lmo1642 protein OS=Listeria monocytogenes serovar 1/2a (strain ATCC BAA-679 / EGD-e) OX=169963 GN=lmo1642 PE=4 SV=1

Query	Observed	Mr(expt)	Mr(calc)	ppm	Miss	Score	Expect	Rank	Unique	Peptide
513	382.718	763.421	763.423	-2.72	0	51	3.2e-05	1	U	K.EFLISR.R 510
784	398.199	794.383	794.381	2.77	0	25	0.01	1	U	R.DFESIGK.R
2733	468.791	935.567	935.569	-2.57	0	29	0.0014	1	U	K.YIITSLVK.W
3038	480.278	958.542	958.545	-3.25	0	39	0.00061	1	U	K.EIIQLSTR.I 3039
4156	523.789	1045.563	1045.577	-12.93	0	27	0.01	1	U	R.TIQVGSNISK.L
6243	603.302	1204.590	1204.594	-2.93	0	59	3.6e-06	1	U	R.LSSGEEEVSIR.A
7505	649.285	1296.556	1296.548	6.41	0	36	0.00028	1	U	K.HMEDMIQSFK.L 7503
12308	839.436	1676.857	1676.862	-3.15	0	60	3.5e-06	1	U	K.LESFGSLEQALTNLGV.-
14405	926.480	1850.945	1850.948	-1.77	0	82	1.9e-08	1	U	R.ALSTASCGMIIDVITGVK.K
16646	736.742	2207.204	2207.205	-0.34	0	118	2.1e-12	1	U	R.IIPITDSIGVLPIVGSLDDDR.G 16647
16648	1104.610	2207.206	2207.205	0.39	0	(97)	3.1e-10	1	U	R.IIPITDSIGVLPIVGSLDDDR.G
16883	751.700	2252.079	2252.074	2.00	0	61	1.9e-06	1	U	K.DTLSSIQQQLLEGFMQENR.N

17. sp|Q8Y422 **Mass:** 43429 **Score:** 530 **Matches:** 11(11) **Sequences:** 9(9) **emPAI:** 1.40
Elongation factor Tu OS=Listeria monocytogenes serovar 1/2a (strain ATCC BAA-679 / EGD-e) OX=169963 GN=tuf PE=1 SV=1

Query	Observed	Mr(expt)	Mr(calc)	ppm	Miss	Score	Expect	Rank	Unique	Peptide
5296	566.321	1130.626	1130.630	-2.74	0	76	1.1e-07	1	U	R.TVGAGVVSNISK.- 5295
5524	576.808	1151.601	1151.601	0.28	0	61	2.7e-06	1	U	K.VVVTGVEMFR.K
7588	652.394	1302.774	1302.776	-1.67	0	64	3.6e-07	1	U	K.TTLTAAITTVLAK.K
12772	852.449	1702.883	1702.889	-3.38	0	99	4.7e-10	1	U	K.LLDYAEAGDNIGALLR.G
13438	587.302	1758.884	1758.889	-2.44	1	90	3e-09	1	U	K.VGDEVEVIGIEEESKK.V
13703	593.296	1776.865	1776.864	0.39	0	80	4.2e-08	1	U	R.GITISTAHVEYQTDSR.H
15618	982.499	1962.983	1962.983	0.27	0	56	8.1e-06	1	U	R.DLLTEYEFPGDDIPVIK.G 15617
15787	665.320	1992.938	1992.935	1.46	0	27	0.0041	1	U	K.IDELMEAVDSYIPTPER.D
16690	739.681	2216.021	2216.013	3.80	0	78	3.2e-08	1	U	R.DTDKPFMMPVEDVFSITGR.G

18. tr|Q8YAM0 **Mass:** 33447 **Score:** 483 **Matches:** 7(7) **Sequences:** 4(4) **emPAI:** 0.66
Lmo0098 protein OS=Listeria monocytogenes serovar 1/2a (strain ATCC BAA-679 / EGD-e) OX=169963 GN=lmo0098 PE=4 SV=1

Query	Observed	Mr(expt)	Mr(calc)	ppm	Miss	Score	Expect	Rank	Unique	Peptide
9058	708.390	1414.766	1414.797	-21.78	0	50	2.4e-05	1	U	R.WVNIQFAPIISK.V
9768	743.382	1484.749	1484.747	1.13	0	93	1.2e-09	1	U	R.ANGAEVDDVAIQGVK.V
10999	794.925	1587.835	1587.836	-0.54	0	114	1.1e-11	1	U	K.ITDDLSGGLLQDITK.G 10998
12691	567.308	1698.903	1698.915	-7.34	0	(57)	5.3e-06	1	U	K.TALEQQQAGLALSEIK.V
12695	850.461	1698.907	1698.915	-4.65	0	108	3.9e-11	1	U	K.TALEQQQAGLALSEIK.V 12694

19. tr|Q7AP52 **Mass:** 62606 **Score:** 360 **Matches:** 7(7) **Sequences:** 7(7) **emPAI:** 0.61
Lmo2196 protein OS=Listeria monocytogenes serovar 1/2a (strain ATCC BAA-679 / EGD-e) OX=169963 GN=lmo2196 PE=4 SV=1

Query	Observed	Mr(expt)	Mr(calc)	ppm	Miss	Score	Expect	Rank	Unique	Peptide
7282	640.825	1279.635	1279.641	-4.78	0	75	1.3e-07	1	U	R.TVLSADYAAQNK.N
7305	428.237	1281.690	1281.682	6.47	1	41	0.00016	1	U	K.ISDDKTVYTIK.L
13418	879.492	1756.970	1756.972	-1.41	0	71	2.1e-07	1	U	K.ILLTDDVAIQPLYQR.S
14389	617.344	1849.011	1849.020	-4.93	1	34	0.00074	1	U	K.AIALAIDKQSYTDTVLK.N
15588	980.488	1958.962	1958.963	-0.14	0	91	2.1e-09	1	U	R.AVDPNTAATYSYLFDAIK.N
15834	1002.463	2002.912	2002.909	1.22	0	59	1.9e-06	1	U	K.YAQNSDNMLFNGPFELK.D

16756	744.384	2230.131	2230.133	-0.93	0	86	7.8e-09	1	U	K.ASGEQVLNLTESALIPSADSTK.A

20. sp|Q8Y440 **Mass:** 22811 **Score:** 330 **Matches:** 6(6) **Sequences:** 6(6) **emPAI:** 2.00
50S ribosomal protein L3 OS=Listeria monocytogenes serovar 1/2a (strain ATCC BAA-679 / EGD-e) OX=169963 GN=rplC PE=3 SV=1

Query	Observed	Mr(expt)	Mr(calc)	ppm	Miss	Score	Expect	Rank	Unique	Peptide
816	400.271	798.528	798.533	-5.35	1	35	0.00034	1	U	K.KALVQIK.T
6949	418.883	1253.626	1253.630	-3.17	0	61	2.4e-06	1	U	R.RPGSMGPVAPNR.V
11047	797.395	1592.776	1592.757	11.8	0	62	1.6e-06	1	U	R.DVNLDEYEIGAEVK.V
12487	844.944	1687.873	1687.881	-5.15	0	72	1.9e-07	1	U	R.MGGEQITIQNLEIVK.V
13173	868.440	1734.866	1734.868	-0.86	0	107	7.3e-11	1	U	K.VDVFAEGDIIDATGVSK.G
16166	1048.557	3142.649	3142.658	-3.08	0	76	9.1e-08	1	U	K.VGMTQVFTENGELIPVTVIEAAQNVVLQK.K

21. tr|Q8Y7B2 **Mass:** 45197 **Score:** 327 **Matches:** 8(8) **Sequences:** 8(8) **emPAI:** 1.11
Dihydrolipoamide acetyltransferase component of pyruvate dehydrogenase complex OS=Listeria monocytogenes serovar 1/2a (strain ATCC BAA-679 / EGD-e)

Query	Observed	Mr(expt)	Mr(calc)	ppm	Miss	Score	Expect	Rank	Unique	Peptide
215	360.226	718.437	718.438	-1.58	0	19	0.025	1	U	K.FLQAIK.A
747	395.235	788.455	788.454	0.51	0	32	0.0028	1	U	R.FSPAVLR.I
1419	423.727	845.439	845.449	-12.62	0	42	0.00029	1	U	R.EISELAGK.A
1936	442.233	882.451	882.456	-5.68	0	43	9.9e-05	1	U	K.EATPNPVR.S
2153	449.262	896.510	896.508	1.96	0	32	0.00089	1	U	R.EIPINGVR.K
2184	450.282	898.549	898.549	0.80	0	45	6.3e-05	1	U	R.ILDGLLAGK.F
9826	746.919	1491.824	1491.830	-3.68	0	92	1.1e-09	1	U	K.DLLQVIENGPVAPK.R
12970	859.434	1716.852	1716.853	-0.30	0	137	7.4e-14	1	U	R.IAGENNIDLSTVEGTGK.G

22. sp|O77727 **Mass:** 48740 **Score:** 304 **Matches:** 10(10) **Sequences:** 5(5) **emPAI:** 0.54
Keratin, type I cytoskeletal 15 (Contact-Cont) OS=Ovis aries GN=KRT15 PE=2 SV=1

Query	Observed	Mr(expt)	Mr(calc)	ppm	Miss	Score	Expect	Rank	Unique	Peptide
897	404.203	806.391	806.392	-1.82	0	45	0.00011	1		R.LAADDFR.L 896 898 900
3947	516.302	1030.590	1030.591	-1.10	0	53	1.9e-05	1		R.VLDELTLTK.T 3946 3948
6167	601.311	1200.607	1200.610	-2.23	0	74	1.4e-07	1		R.QSVEADINGLR.R
7558	651.330	1300.645	1300.651	-4.39	0	67	1.6e-06	1	U	R.ALEEANADLEVK.I
8338	453.244	1356.711	1356.711	-0.16	1	54	1.3e-05	1		R.QSVEADINGLRR.V

23. sp|P0DJM2 **Mass:** 66104 **Score:** 298 **Matches:** 7(6) **Sequences:** 7(6) **emPAI:** 0.47
Chaperone protein DnaK OS=Listeria monocytogenes serovar 1/2a (strain ATCC BAA-679 / EGD-e) OX=169963 GN=dnaK PE=3 SV=1

Query	Observed	Mr(expt)	Mr(calc)	ppm	Miss	Score	Expect	Rank	Unique	Peptide
1628	431.231	860.447	860.460	-16.01	0	18	0.098	1	U	R.NTTIPTSK.S
1976	443.753	885.491	885.492	-0.98	0	43	0.00018	1	U	K.IAGLEVER.I
7998	665.372	1328.730	1328.730	0.13	0	70	6.2e-07	1	U	R.AAITNPNTISSIK.R
8419	682.345	1362.675	1362.678	-2.35	0	57	5.8e-06	1	U	R.NNADQLVFTVDK.T
10699	782.907	1563.800	1563.805	-2.81	0	55	1.2e-05	1	U	K.AVITVPAYFNDAQR.Q
12571	847.430	1692.846	1692.839	3.71	0	35	0.00097	1	U	R.IINEPTAAALAYGMDK.T
15947	1016.010	2030.005	2030.028	-11.40	0	121	1.8e-12	1	U	K.DANLSASDIDQVILVGGSTR.I

24. tr|Q8Y865 **Mass:** 41241 **Score:** 295 **Matches:** 8(8) **Sequences:** 8(8) **emPAI:** 1.26
PdhA protein OS=Listeria monocytogenes serovar 1/2a (strain ATCC BAA-679 / EGD-e) OX=169963 GN=pdhA PE=4 SV=1

Query	Observed	Mr(expt)	Mr(calc)	ppm	Miss	Score	Expect	Rank	Unique	Peptide
335	370.710	739.405	739.402	3.96	0	37	0.00079	1	U	K.AFLFSR.G
2432	459.268	916.522	916.523	-1.46	0	59	4.3e-06	1	U	K.QTVTDLLK.N
3392	494.259	986.503	986.503	-0.61	0	26	0.0071	1	U	K.ENAVIDQAK.E
4150	523.775	1045.536	1045.540	-4.10	0	83	2.5e-08	1	U	K.QSAAETLAQK.A
7584	652.344	1302.673	1302.678	-4.14	1	31	0.0028	1	U	R.EKQSAAETLAQK.A
8380	680.874	1359.734	1359.740	-3.96	0	87	8.6e-09	1	U	K.QFELVQILNEK.G
11276	539.640	1615.899	1615.909	-6.20	0	35	0.00061	1	U	R.DVPQLIWHGLPLTK.A
17462	786.752	2357.234	2357.230	1.50	0	60	2.2e-06	1	U	K.AVAAGIPGVQVDGMDPLAVYAVTK.F

25. tr|Q8YAJ0 **Mass:** 58308 **Score:** 295 **Matches:** 10(10) **Sequences:** 9(9) **emPAI:** 0.92
Lmo0135 protein OS=Listeria monocytogenes serovar 1/2a (strain ATCC BAA-679 / EGD-e) OX=169963 GN=lmo0135 PE=1 SV=1

Query	Observed	Mr(expt)	Mr(calc)	ppm	Miss	Score	Expect	Rank	Unique	Peptide
1453	425.246	848.477	848.480	-3.00	0	21	0.024	1	U	K.TFFPIPK.H
4539	538.779	1075.543	1075.540	3.04	0	37	0.001	1	U	K.VEAVDDTTVK.F
6361	607.823	1213.632	1213.634	-1.94	0	70	2.7e-07	1	U	K.LTVYYLNNSK.S
6588	615.795	1229.576	1229.572	3.65	0	19	0.025	1	U	R.FNDYFDGKPK.L
6681	617.808	1233.602	1233.618	-12.41	0	23	0.021	1	U	K.AATPQPVTMFR.D
9393	724.379	1446.744	1446.747	-1.67	0	69	3.9e-07	1	U	K.ASAVNIITYPENR.L
10944	792.395	1582.775	1582.773	1.62	0	93	1.9e-09	1	U	K.SLDISDDNLTYTVK.L 10943
14154	913.472	1824.930	1824.933	-1.57	0	62	1.9e-06	1	U	K.DQNAANLALQNGEINLK.S
17338	775.064	2322.170	2322.174	-2.04	1	40	0.00026	1	U	R.GNFVFDDKPVKVEAVDDTTVK.F

26. sp|Q92EP8 **Mass:** 33645 **Score:** 273 **Matches:** 6(6) **Sequences:** 6(6) **emPAI:** 1.12
UPF0365 protein lmo0392 OS=Listeria monocytogenes serovar 1/2a (strain ATCC BAA-679 / EGD-e) OX=169963 GN=lmo0392 PE=3 SV=1

Query	Observed	Mr(expt)	Mr(calc)	ppm	Miss	Score	Expect	Rank	Unique	Peptide
1594	429.245	856.475	856.477	-1.81	0	55	8.2e-06	1	U	R.AAAIDLAGR.D
6573	614.861	1227.708	1227.701	5.54	0	65	1.2e-06	1	U	R.VPVGLGTLIGMR.L
6978	629.342	1256.670	1256.672	-2.05	0	88	5.3e-09	1	U	R.IVGGAGEDTVIAR.V
7585	652.347	1302.679	1302.678	0.57	0	80	3.3e-08	1	U	R.VGEAVVSTVGETR.E
7742	657.276	1312.538	1312.539	-0.60	0	40	9.4e-05	1	U	K.MENVQSDTAMR.E
10833	788.400	1574.785	1574.794	-5.78	0	26	0.018	1	U	R.TPEFTGVAQNGVEVK.V

27. sp|Q8Y4C1 **Mass:** 51576 **Score:** 270 **Matches:** 7(7) **Sequences:** 7(7) **emPAI:** 0.77
ATP synthase subunit beta 2 OS=Listeria monocytogenes serovar 1/2a (strain ATCC BAA-679 / EGD-e) OX=169963 GN=atpD2 PE=3 SV=1

Query	Observed	Mr(expt)	Mr(calc)	ppm	Miss	Score	Expect	Rank	Unique	Peptide
3258	488.284	974.553	974.555	-1.81	0	30	0.0038	1	U	K.IGLFGGAGVGK.T
7081	633.310	1264.605	1264.608	-2.72	0	61	5.5e-06	1	U	R.TIAMASTDGVQR.G
9254	718.377	1434.739	1434.747	-5.66	0	90	3.9e-09	1	U	R.FTQAGSEVSALLGR.M
16005	682.700	2045.077	2045.079	-1.26	1	41	0.00018	1	U	R.KLTEQGIYPAVDPLASTSR.A
16453	726.715	2177.123	2177.110	5.79	0	30	0.0036	1	U	R.EAPTFDQLATTTEILETGIK.V
16475	728.707	2183.098	2183.086	5.64	0	84	1.1e-08	1	U	R.ALSPDIVGEEHYAVATEVQR.L
17411	780.370	2338.087	2338.093	-2.52	0	38	0.00021	1	U	R.MPSAVGYQPTLATEMGQLQER.I

28. sp|Q9AGE6 **Mass:** 57332 **Score:** 268 **Matches:** 8(8) **Sequences:** 8(8) **emPAI:** 0.80
60 kDa chaperonin OS=Listeria monocytogenes serovar 1/2a (strain ATCC BAA-679 / EGD-e) OX=169963 GN=groL PE=3 SV=1

Query	Observed	Mr(expt)	Mr(calc)	ppm	Miss	Score	Expect	Rank	Unique	Peptide
948	407.259	812.503	812.512	-11.53	0	26	0.0049	1	U	K.LAGGVAVVK.V
1136	416.740	831.465	831.470	-6.45	0	48	0.0001	1	U	K.LVSEVASK.T
3739	507.783	1013.552	1013.551	0.98	0	36	0.00064	1	U	R.GVDQLANAVK.V
6466	609.820	1217.625	1217.625	-0.46	0	60	6.3e-06	1	U	K.TATVDQLGTANK.V
8375	680.849	1359.683	1359.688	-4.16	0	69	5.7e-07	1	U	R.VGNDGVITIEESK.G
12563	564.978	1691.911	1691.895	9.45	0	41	0.00012	1	U	R.QIAHNAGLEGSVIVER.L
12951	572.921	1715.742	1715.731	6.42	1	33	0.0005	1	U	R.AQMEETTSEFDREK.L
13819	895.444	1788.873	1788.874	-0.90	0	72	2.8e-07	1	U	K.ESIAQVAAISSGDEEVGK.L

29. sp|Q8Y446 **Mass:** 17445 **Score:** 261 **Matches:** 7(7) **Sequences:** 7(7) **emPAI:** 4.32
30S ribosomal protein S5 OS=Listeria monocytogenes serovar 1/2a (strain ATCC BAA-679 / EGD-e) OX=169963 GN=rpsE PE=3 SV=1

Query	Observed	Mr(expt)	Mr(calc)	ppm	Miss	Score	Expect	Rank	Unique	Peptide
15	351.217	700.419	700.423	-5.89	0	40	0.00054	1	U	R.VVTINR.V
204	359.209	716.404	716.407	-4.04	0	42	0.00039	1	U	R.ATIDGIK.Q
3537	499.762	997.509	997.519	-10.02	0	35	0.0015	1	U	K.AQEVPDAIR.K
4178	524.803	1047.592	1047.596	-4.45	0	40	0.00032	1	U	R.FTALVVVGDK.N
5218	376.211	1125.611	1125.614	-2.93	1	39	0.00021	1	U	K.AQEVPDAIRK.A
7592	652.833	1303.652	1303.655	-2.41	0	84	1.5e-08	1	U	K.SLGSNTPINMVR.A
8355	679.877	1357.740	1357.745	-3.90	0	86	8.3e-09	1	U	R.AVLELAGVADVSSK.S

30. tr|Q8Y4P6 **Mass:** 44200 **Score:** 258 **Matches:** 6(6) **Sequences:** 6(6) **emPAI:** 0.77
Lmo2389 protein OS=Listeria monocytogenes serovar 1/2a (strain ATCC BAA-679 / EGD-e) OX=169963 GN=lmo2389 PE=4 SV=1

Query	Observed	Mr(expt)	Mr(calc)	ppm	Miss	Score	Expect	Rank	Unique	Peptide
608	389.197	776.379	776.382	-3.78	0	35	0.0011	1	U	K.QYDVPR.E
4803	548.748	1095.481	1095.483	-1.74	0	59	1.9e-06	1	U	K.YAESENEVR.E
4830	549.752	1097.489	1097.489	0.82	0	44	5.1e-05	1	U	R.IYCMEAAPK.V
5517	576.316	1150.617	1150.623	-5.38	0	38	0.00044	1	U	K.TTFIQDTVVK.I
7530	649.903	1297.792	1297.797	-3.48	0	70	1e-07	1	U	R.ALLLIGGSGVLASK.G
8057	667.852	1333.689	1333.688	0.81	0	90	3.2e-09	1	U	K.LVDYGVGVLEDR.G

31. tr|Q8Y864 **Mass:** 35288 **Score:** 249 **Matches:** 7(7) **Sequences:** 5(5) **emPAI:** 0.82
PdhB protein OS=Listeria monocytogenes serovar 1/2a (strain ATCC BAA-679 / EGD-e) OX=169963 GN=PdhB PE=4 SV=1

Query	Observed	Mr(expt)	Mr(calc)	ppm	Miss	Score	Expect	Rank	Unique	Peptide
890	403.244	804.473	804.475	-2.22	1	26	0.0069	1	U	R.VKEVIAF.-
1378	421.775	841.535	841.539	-4.59	0	33	0.0011	1	U	K.GLLISAIR.D
6015	595.326	1188.637	1188.639	-1.37	0	53	1.7e-05	1	U	K.VVIPSTPYDAK.G
9625	491.905	1472.692	1472.697	-3.18	0	49	2.6e-05	1	U	R.DNDPVIFLEHMK.L
10966	793.454	1584.893	1584.897	-2.99	0	91	1.1e-09	1	U	R.TISPIDVETIIASVK.K 10965
10967	529.308	1584.902	1584.897	3.05	0	(46)	2.7e-05	1	U	R.TISPIDVETIIASVK.K

32. tr|Q8Y841 **Mass:** 65665 **Score:** 248 **Matches:** 8(8) **Sequences:** 7(7) **emPAI:** 0.57
Lmo1077 protein OS=Listeria monocytogenes serovar 1/2a (strain ATCC BAA-679 / EGD-e) OX=169963 GN=lmo1077 PE=4 SV=1

Query	Observed	Mr(expt)	Mr(calc)	ppm	Miss	Score	Expect	Rank	Unique	Peptide
2466	460.762	919.509	919.513	-4.57	0	59	5e-06	1	U	K.NNSVVIFK.M
2530	462.743	923.471	923.471	-0.49	0	28	0.005	1	U	R.ILFSSDSR.A
5563	578.325	1154.635	1154.630	4.36	0	43	0.00021	1	U	K.ESSILPTGVPR.T
6045	596.342	1190.670	1190.681	-9.12	0	46	3.8e-05	1	U	K.QVILFAPTFR.G 6044
8269	677.369	1352.724	1352.734	-7.32	0	46	6.2e-05	1	U	K.SIFYTSQALVPK.N
4340	531.603	1591.786	1591.788	-1.29	0	67	5.7e-07	1	U	R.TLTDEFHFSPTGIK.L
15936	1014.526	2027.038	2027.025	6.30	0	49	5.6e-05	1	U	R.AIVASDTDIPFYAEAFGIK.E

33. sp|Q8YAA4 **Mass:** 24517 **Score:** 242 **Matches:** 8(8) **Sequences:** 7(7) **emPAI:** 2.31
50S ribosomal protein L1 OS=Listeria monocytogenes serovar 1/2a (strain ATCC BAA-679 / EGD-e) OX=169963 GN=rplA PE=3 SV=1

Query	Observed	Mr(expt)	Mr(calc)	ppm	Miss	Score	Expect	Rank	Unique	Peptide
319	369.194	736.374	736.376	-2.23	0	29	0.0055	1	U	K.VSFDAAK.L 318
616	389.215	776.415	776.418	-4.49	0	29	0.0062	1	U	K.LVENFR.T
3726	506.790	1011.566	1011.571	-4.88	0	35	0.00065	1	U	R.GAVVLPNGTGK.T
4426	534.761	1067.507	1067.517	-9.07	0	77	5.4e-08	1	U	K.TGTVTMDVTK.A
7890	661.844	1321.673	1321.677	-2.32	0	74	1.9e-07	1	U	K.VYTAEEAVELAK.K
8061	667.866	1333.718	1333.724	-4.64	0	46	8.3e-05	1	U	K.NLSVTTTFGPGIK.V
15039	950.944	1899.873	1899.837	18.7	0	55	5.9e-06	1	U	K.EAEAAGADYVGESEFVEK.I

34. tr|Q7AP53 **Mass:** 39745 **Score:** 238 **Matches:** 7(7) **Sequences:** 7(7) **emPAI:** 1.10
Lmo2193 protein OS=Listeria monocytogenes serovar 1/2a (strain ATCC BAA-679 / EGD-e) OX=169963 GN=lmo2193 PE=3 SV=1

Query	Observed	Mr(expt)	Mr(calc)	ppm	Miss	Score	Expect	Rank	Unique	Peptide
1306	420.717	839.418	839.421	-3.17	0	54	6.6e-06	1	U	R.VAVMYGGK.I

2586	464.276	926.538	926.544	-6.40	0	36	0.00044	1	U	K.QISEPLIK.H
2998	478.258	954.501	954.481	20.5	0	17	0.046	1	U	R.GVNFDLYK.G
4847	550.302	1098.590	1098.592	-1.90	0	36	0.00045	1	U	R.LLPEGNSEIK.S
7594	652.838	1303.661	1303.662	-0.59	0	44	0.00017	1	U	K.GETLAIVGESGSGK.S
8962	705.357	1408.699	1408.702	-2.15	0	64	1.3e-06	1	U	K.SGQILFNGMDIAK.A
9165	713.403	1424.792	1424.799	-4.52	0	83	8.6e-09	1	U	R.LLQLVGIANAEER.I

35. sp|Q8Y4B3 **Mass:** 23044 **Score:** 236 **Matches:** 6(6) **Sequences:** 5(5) **emPAI:** 1.48
Uracil phosphoribosyltransferase OS=Listeria monocytogenes serovar 1/2a (strain ATCC BAA-679 / EGD-e) OX=169963 GN=upp PE=3 SV=1

Query	Observed	Mr(expt)	Mr(calc)	ppm	Miss	Score	Expect	Rank	Unique	Peptide
1874	440.803	879.591	879.591	0.35	0	43	4.7e-05	1	U	K.LGIVPILR.A
2828	472.737	943.459	943.461	-2.28	0	60	2e-06	1	U	K.LPSDVEER.L
5094	559.796	1117.578	1117.580	-1.57	0	74	1.5e-07	1	U	R.AGLGMQDGILK.L
14621	934.966	1867.918	1867.924	-3.20	0	38	0.00059	1	U	R.ELVDEVATLMAYEITR.D
16448	1089.527	2177.039	2177.041	-0.89	0	61	2.1e-06	1	U	R.DMELEDIQVETPLQTTTAK.T
16450	726.692	2177.054	2177.041	5.94	0	(42)	0.00019	1	U	R.DMELEDIQVETPLQTTTAK.T

36. sp|Q8Y6M7 **Mass:** 32675 **Score:** 219 **Matches:** 6(5) **Sequences:** 6(5) **emPAI:** 0.91
Elongation factor Ts OS=Listeria monocytogenes serovar 1/2a (strain ATCC BAA-679 / EGD-e) OX=169963 GN=tsf PE=3 SV=1

Query	Observed	Mr(expt)	Mr(calc)	ppm	Miss	Score	Expect	Rank	Unique	Peptide
2201	451.257	900.499	900.503	-4.18	0	51	3.4e-05	1	U	K.IGENISLR.R
3677	504.263	1006.512	1006.497	14.7	0	22	0.063	1	U	R.FEVGEGIEK.K
6726	619.286	1236.558	1236.554	2.77	0	60	2.3e-06	1	U	K.ALVETEGDMEK.A
7485	648.336	1294.657	1294.670	-9.98	0	34	0.0031	1	U	K.DVAMHIAAINPK.Y
9647	738.377	1474.739	1474.742	-1.85	0	85	2.8e-08	1	U	K.NDNFQQLVDALAK.Q
13128	866.473	1730.931	1730.967	-20.49	0	57	7e-06	1	U	R.IGVLTLLEGTTDTTVAK.D

37. sp|P66548 **Mass:** 24527 **Score:** 214 **Matches:** 8(8) **Sequences:** 8(8) **emPAI:** 2.92
30S ribosomal protein S3 OS=Listeria monocytogenes serovar 1/2a (strain ATCC BAA-679 / EGD-e) OX=169963 GN=rpsC PE=3 SV=1

Query	Observed	Mr(expt)	Mr(calc)	ppm	Miss	Score	Expect	Rank	Unique	Peptide
351	372.217	742.420	742.423	-3.71	0	37	0.00063	1	U	R.GEVLPTK.K
576	386.718	771.422	771.424	-2.18	0	55	1e-05	1	U	R.LGGADIAR.A
1154	417.718	833.421	833.424	-4.25	0	55	1.3e-05	1	U	R.LSDASVSR.V
2427	459.237	916.460	916.461	-1.67	0	59	1.3e-05	1	U	K.GGSEVEALR.K
3037	480.258	958.501	958.508	-7.37	0	40	0.00033	1	U	K.NLNELTQK.R
4123	523.281	1044.548	1044.556	-8.27	1	28	0.0058	1	U	K.GGSEVEALRK.N
8808	697.327	1392.640	1392.631	6.65	0	36	0.00044	1	U	K.DYADFLHEDLR.I
12865	428.221	1708.854	1708.853	0.32	0	30	0.0033	1	U	R.AEHYSEGTVPLHTLR.A

38. sp|Q927L9 **Mass:** 19983 **Score:** 203 **Matches:** 5(5) **Sequences:** 5(5) **emPAI:** 1.84
50S ribosomal protein L5 OS=Listeria monocytogenes serovar 1/2a (strain ATCC BAA-679 / EGD-e) OX=169963 GN=rplE PE=3 SV=1

Query	Observed	Mr(expt)	Mr(calc)	ppm	Miss	Score	Expect	Rank	Unique	Peptide
1869	440.236	878.458	878.461	-3.70	0	30	0.0045	1	U	R.GNYTLGVR.E
1950	442.782	883.549	883.549	-0.40	0	40	0.00013	1	U	K.LVTVSLPR.V
3608	502.280	1002.546	1002.542	3.69	0	30	0.0036	1	U	K.EIVPALMSK.F
9355	722.392	1442.769	1442.773	-2.66	0	117	1.2e-11	1	U	K.IVINTGVGDATANAK.V
16735	742.087	2223.240	2223.273	-14.87	0	53	6e-06	1	U	K.VLDSAVEELALITGQKPVITK.A

39. sp|P61055 **Mass:** 22590 **Score:** 196 **Matches:** 6(6) **Sequences:** 5(5) **emPAI:** 1.52
50S ribosomal protein L4 OS=Listeria monocytogenes serovar 1/2a (strain ATCC BAA-679 / EGD-e) OX=169963 GN=rplD PE=3 SV=1

Query	Observed	Mr(expt)	Mr(calc)	ppm	Miss	Score	Expect	Rank	Unique	Peptide
1050	413.224	824.433	824.443	-12.17	0	46	0.00022	1	U	K.EFAAFLK.N
4097	522.284	1042.553	1042.556	-2.58	0	53	1.7e-05	1	U	R.GGGVVFGPTPR.S
8908	701.400	1400.786	1400.792	-3.82	0	75	4.8e-08	1	U	K.LVVLEGLTFDAPK.T 8907
16109	1041.100	2080.185	2080.178	3.33	0	47	2.1e-05	1	U	R.NLQGITVIPAESISVLEVAK.H
17969	1231.586	2461.157	2461.161	-1.50	0	20	0.021	1	U	K.QDGTNAGEITLNDTVFGIEPNEK.V

40. sp|P66042 **Mass:** 17739 **Score:** 195 **Matches:** 6(6) **Sequences:** 6(6) **emPAI:** 3.08
50S ribosomal protein L10 OS=Listeria monocytogenes serovar 1/2a (strain ATCC BAA-679 / EGD-e) OX=169963 GN=rplJ PE=3 SV=1

Query	Observed	Mr(expt)	Mr(calc)	ppm	Miss	Score	Expect	Rank	Unique	Peptide
1003	410.728	819.441	819.449	-10.21	0	35	0.002	1	U	K.ILNDFAK.D
1097	414.749	827.484	827.486	-3.09	0	51	1.7e-05	1	U	K.ALATLPSR.E
2008	444.755	887.495	887.496	-1.92	0	40	0.00046	1	U	K.VASLEEIK.A
5303	378.204	1131.589	1131.614	-21.25	1	29	0.0057	1	U	K.QSAVEEIKTK.L
7766	657.859	1313.703	1313.730	-20.73	1	28	0.0058	1	U	R.GLNVGEITDLRK.Q
8629	691.368	1380.721	1380.725	-2.49	0	102	1.4e-10	1	U	K.LSASASTVIVDYR.G

41. tr|Q8Y5H3 **Mass:** 38432 **Score:** 195 **Matches:** 6(6) **Sequences:** 6(6) **emPAI:** 0.93
Lmo2089 protein OS=Listeria monocytogenes serovar 1/2a (strain ATCC BAA-679 / EGD-e) OX=169963 GN=lmo2089 PE=4 SV=1

Query	Observed	Mr(expt)	Mr(calc)	ppm	Miss	Score	Expect	Rank	Unique	Peptide
913	404.713	807.411	807.413	-1.45	0	52	3.9e-05	1	U	K.ASQEFVK.N
5780	586.337	1170.659	1170.676	-14.47	1	34	0.0012	1	U	R.KYDPLVAPIR.S
6021	595.782	1189.550	1189.557	-6.02	0	66	5.3e-07	1	U	K.NTSPLSDVDSR.Y
9399	724.847	1447.679	1447.683	-3.03	0	26	0.0045	1	U	K.DYNPEVLENLDK.A
16870	751.018	2250.033	2250.040	-3.01	0	32	0.0014	1	U	R.DASVLYPSMDEFAEGYVLTK.E
17282	772.419	2314.236	2314.238	-0.74	0	66	3.4e-07	1	U	K.SSDIIVAGDSVGGNLATVVTQIAK.S

42. sp|Q8Y6Y9 **Mass:** 11207 **Score:** 188 **Matches:** 4(4) **Sequences:** 4(4) **emPAI:** 3.33
50S ribosomal protein L21 OS=Listeria monocytogenes serovar 1/2a (strain ATCC BAA-679 / EGD-e) OX=169963 GN=rplU PE=3 SV=1

Query	Observed	Mr(expt)	Mr(calc)	ppm	Miss	Score	Expect	Rank	Unique	Peptide

3462	496.775	991.535	991.534	0.78	0	41	0.00024	1	U	K.VLFVGGDSAK.V
7067	632.813	1263.612	1263.635	-17.97	0	28	0.0063	1	U	K.VEAGQEIYVEK.L
8222	675.348	1348.682	1348.687	-3.97	0	73	2.6e-07	1	U	K.LAGEVGDVVTFDK.V
8524	687.882	1373.749	1373.755	-4.55	0	100	2.7e-10	1	U	K.VGVPFVEGATVTAK.V

43. sp|Q8Y4C0 **Mass:** 55110 **Score:** 187 **Matches:** 8(8) **Sequences:** 8(8) **emPAI:** 0.85
ATP synthase subunit alpha 2 OS=Listeria monocytogenes serovar 1/2a (strain ATCC BAA-679 / EGD-e) OX=169963 GN=atpA2 PE=3 SV=1

Query	Observed	Mr(expt)	Mr(calc)	ppm	Miss	Score	Expect	Rank	Unique	Peptide
1025	411.227	820.439	820.444	-6.88	0	30	0.0047	1	U	R.LDLAAYR.E
1385	422.268	842.521	842.523	-2.08	0	36	0.00055	1	U	R.ELSLLLR.R
3432	495.279	988.543	988.544	-1.14	0	28	0.013	1	U	K.AEEISSIIK.Q
3851	512.808	1023.602	1023.608	-5.20	0	39	0.00014	1	U	K.AIDALVPIGR.G
7748	657.360	1312.706	1312.699	5.48	0	76	1.4e-07	1	U	R.QSVNEPLQTGIK.A
8880	700.382	1398.749	1398.754	-3.64	0	43	0.00016	1	U	K.IMEVPVGEALIGR.V
11334	541.610	1621.807	1621.831	-14.83	0	27	0.0054	1	U	K.VSDVGTVTYIGDGIAR.A
20136	844.448	3373.761	3373.766	-1.47	0	25	0.0031	1	U	R.VVNSLGQPVDGLGPIETTGTRPIEAVAPGVMQR.Q

44. tr|Q8Y795 **Mass:** 34168 **Score:** 187 **Matches:** 5(5) **Sequences:** 5(5) **emPAI:** 0.85
Lmo1395 protein OS=Listeria monocytogenes serovar 1/2a (strain ATCC BAA-679 / EGD-e) OX=169963 GN=lmo1395 PE=4 SV=1

Query	Observed	Mr(expt)	Mr(calc)	ppm	Miss	Score	Expect	Rank	Unique	Peptide
5212	563.328	1124.641	1124.644	-3.06	0	48	1.7e-05	1	U	K.QLELAPDLVK.Q
7971	664.820	1327.626	1327.626	0.16	0	51	1.3e-05	1	U	K.GETSGNATTYTVK.N
8008	665.862	1329.709	1329.714	-3.81	0	66	1e-06	1	U	K.GLSLDDLQQITK.I
10079	757.939	1513.864	1513.872	-4.94	0	18	0.029	1	U	K.TVSIVIGNSPVTTVK.I
11126	800.877	1599.740	1599.742	-1.63	0	65	5.8e-07	1	U	R.NPMPAQSVGNQANTR.N

45. sp|Q927L7 **Mass:** 13332 **Score:** 176 **Matches:** 6(6) **Sequences:** 5(5) **emPAI:** 3.72
50S ribosomal protein L14 OS=Listeria monocytogenes serovar 1/2a (strain ATCC BAA-679 / EGD-e) OX=169963 GN=rplN PE=3 SV=1

Query	Observed	Mr(expt)	Mr(calc)	ppm	Miss	Score	Expect	Rank	Unique	Peptide
23	351.723	701.432	701.432	-0.83	0	38	0.00059	1	U	R.EVLTIK.V
462	380.228	758.442	758.444	-2.19	0	26	0.018	1	U	R.IFGPVAR.E
1582	428.746	855.478	855.481	-4.05	0	46	4.3e-05	1	U	K.QATPGGVVK.K
2779	470.788	939.562	939.564	-2.43	0	45	3.3e-05	1	U	K.IVSLAPEVL.- 2780
8546	688.361	1374.706	1374.718	-8.21	0	72	1.9e-07	1	U	K.TANIGDVVVCTVK.Q

46. tr|Q7AP59 **Mass:** 18435 **Score:** 175 **Matches:** 3(3) **Sequences:** 3(3) **emPAI:** 0.97
Lmo1601 protein OS=Listeria monocytogenes serovar 1/2a (strain ATCC BAA-679 / EGD-e) OX=169963 GN=lmo1601 PE=4 SV=1

Query	Observed	Mr(expt)	Mr(calc)	ppm	Miss	Score	Expect	Rank	Unique	Peptide
7226	638.322	1274.630	1274.635	-4.02	0	70	3.1e-07	1	U	R.EDLNTQVDTIK.E
7611	653.355	1304.695	1304.698	-1.96	0	53	1.9e-05	1	U	K.LVDSAGGFVDVVK.D
12761	568.280	1701.817	1701.817	0.30	1	87	4.8e-09	1	U	K.EVVSQNKEENQEAAK.K

47. tr|Q8YAC6 **Mass:** 76305 **Score:** 169 **Matches:** 7(7) **Sequences:** 7(7) **emPAI:** 0.48
ATP-dependent zinc metalloprotease FtsH OS=Listeria monocytogenes serovar 1/2a (strain ATCC BAA-679 / EGD-e) OX=169963 GN=ftsH PE=3 SV=1

Query	Observed	Mr(expt)	Mr(calc)	ppm	Miss	Score	Expect	Rank	Unique	Peptide
853	402.215	802.416	802.418	-3.07	0	43	0.00054	1	U	K.VETLDAR.Q
2190	450.792	899.570	899.569	0.72	0	27	0.0019	1	U	K.LIAETLLK.V
5587	579.294	1156.573	1156.576	-2.80	0	40	0.00021	1	U	R.SVYTINGEFK.S
5802	587.786	1173.557	1173.555	1.35	0	26	0.0042	1	U	K.DISYSEFVSK.L
6388	406.214	1215.622	1215.628	-5.36	0	29	0.0042	1	U	R.QIMVDRPDVK.G
8805	464.937	1391.789	1391.788	0.69	0	43	6.9e-05	1	U	R.ADVLDPALLRPGR.F
16376	1079.495	2156.976	2156.986	-4.56	0	57	4.2e-06	1	U	R.VAEEVTFGEVTTGASNDFER.A

48. tr|Q8Y436 **Mass:** 32778 **Score:** 163 **Matches:** 6(6) **Sequences:** 6(6) **emPAI:** 1.16
Lmo2637 protein OS=Listeria monocytogenes serovar 1/2a (strain ATCC BAA-679 / EGD-e) OX=169963 GN=lmo2637 PE=4 SV=1

Query	Observed	Mr(expt)	Mr(calc)	ppm	Miss	Score	Expect	Rank	Unique	Peptide
2558	463.735	925.455	925.458	-3.00	0	33	0.0013	1	U	K.GFMSIEVK.D
2715	468.740	935.466	935.460	6.35	0	33	0.0023	1	U	K.VYNLVEAE.-
2774	470.745	939.475	939.474	1.04	0	39	0.0003	1	U	R.VVFSMDVK.D
2882	474.265	946.516	946.512	3.98	0	26	0.011	1	U	K.EYIPALNK.S
8391	681.366	1360.718	1360.720	-1.31	0	52	2.4e-05	1	U	K.ADTTTISINNLAK.M
15965	1017.515	2033.016	2033.018	-1.09	0	68	8.1e-07	1	U	K.IYAAQLQNAAQNGNTDTIK.V

49. tr|Q8Y7I0 **Mass:** 50714 **Score:** 152 **Matches:** 6(6) **Sequences:** 6(6) **emPAI:** 0.65
Glutamine synthetase OS=Listeria monocytogenes serovar 1/2a (strain ATCC BAA-679 / EGD-e) OX=169963 GN=glnA PE=3 SV=1

Query	Observed	Mr(expt)	Mr(calc)	ppm	Miss	Score	Expect	Rank	Unique	Peptide
2908	475.732	949.450	949.451	-0.75	0	27	0.006	1	U	R.FADEQNVK.F
5225	563.826	1125.637	1125.639	-1.84	0	29	0.0025	1	U	K.NVEIPVSQLK.K
7017	630.880	1259.746	1259.749	-1.96	0	22	0.007	1	U	R.LQFTDILGIIK.N
10056	756.884	1511.753	1511.762	-6.03	0	60	2.8e-06	1	U	R.GYTAVTNPTINSFK.R
13970	902.980	1803.946	1803.944	1.02	0	44	8.5e-05	1	U	R.SVDPSANPYLAMAVLLK.A
14230	612.668	1834.982	1834.979	1.92	1	50	2.6e-05	1	U	K.AGLSGIKDELTPPAPVDR.N

50. tr|Q8Y4I1 **Mass:** 36435 **Score:** 150 **Matches:** 4(4) **Sequences:** 4(4) **emPAI:** 0.59
Glyceraldehyde-3-phosphate dehydrogenase OS=Listeria monocytogenes serovar 1/2a (strain ATCC BAA-679 / EGD-e) OX=169963 GN=gap PE=3 SV=1

Query	Observed	Mr(expt)	Mr(calc)	ppm	Miss	Score	Expect	Rank	Unique	Peptide
4059	520.820	1039.626	1039.628	-1.81	0	45	3.1e-05	1	U	K.AIGEVLPTLK.G
6234	602.818	1203.622	1203.617	3.97	0	18	0.052	1	U	K.VVISAPATGDMK.T
9327	721.382	1440.749	1440.757	-5.93	0	62	1.6e-06	1	U	R.AAAENIIPNTTGAAK.A
10885	789.372	1576.730	1576.708	13.8	0	71	1.4e-07	1	U	K.GMTFGSLFDETQTK.V

51. tr|Q8Y6Y3 **Mass:** 35595 **Score:** 145 **Matches:** 8(7) **Sequences:** 7(6) **emPAI:** 1.03
MreB protein OS=Listeria monocytogenes serovar 1/2a (strain ATCC BAA-679 / EGD-e) OX=169963 GN=mreB PE=4 SV=1

Query	Observed	Mr(expt)	Mr(calc)	ppm	Miss	Score	Expect	Rank	Unique	Peptide
1814	438.728	875.442	875.442	0.07	0	19	0.091	1	U	R.EPSVVAMK.K
2675	468.230	934.445	934.443	2.19	0	23	0.015	1	U	K.MEIGSASPK.G
3089	482.265	962.515	962.519	-4.01	0	43	0.0002	1	U	K.YNLLIGDR.T
4914	552.814	1103.613	1103.598	14.4	0	27	0.0085	1	U	K.GLDLSPFSIR.G
5226	563.849	1125.684	1125.687	-2.71	0	52	6.8e-06	1	U	K.GIVLTGGGALLR.N
8022	666.835	1331.655	1331.657	-1.33	0	35	0.0019	1	U	K.DTQEIVAVGSDAK.N 8026
9986	753.388	1504.761	1504.763	-1.11	0	30	0.0033	1	U	R.VMICVPSGITGVEK.R

52. tr|Q8Y6Q0 **Mass:** 95135 **Score:** 143 **Matches:** 6(6) **Sequences:** 6(6) **emPAI:** 0.31
Aldehyde-alcohol dehydrogenase OS=Listeria monocytogenes serovar 1/2a (strain ATCC BAA-679 / EGD-e) OX=169963 GN=lmo1634 PE=3 SV=1

Query	Observed	Mr(expt)	Mr(calc)	ppm	Miss	Score	Expect	Rank	Unique	Peptide
700	393.260	784.505	784.506	-0.59	0	28	0.0034	1	U	K.ILVAEIK.G
1791	437.737	873.460	873.456	5.48	0	33	0.0023	1	U	K.LAVEETGR.G
2415	458.771	915.527	915.528	-0.69	0	32	0.0022	1	U	K.SLVDEIIK.L
3413	494.782	987.549	987.560	-10.69	0	38	0.00062	1	U	R.SVNDIILSK.S
8871	700.303	1398.592	1398.591	1.16	0	47	1.9e-05	1	U	R.AFMDQCTTANPK.Q
9219	716.880	1431.746	1431.761	-10.44	0	50	4.4e-05	1	U	R.IIGFPAATTEEGVK.S

53. tr|Q8Y581 **Mass:** 36712 **Score:** 139 **Matches:** 4(4) **Sequences:** 4(4) **emPAI:** 0.58
Lmo2192 protein OS=Listeria monocytogenes serovar 1/2a (strain ATCC BAA-679 / EGD-e) OX=169963 GN=lmo2192 PE=3 SV=1

Query	Observed	Mr(expt)	Mr(calc)	ppm	Miss	Score	Expect	Rank	Unique	Peptide
1303	420.702	839.389	839.385	4.61	0	17	0.027	1	U	K.EMLEFR.R
5834	393.186	1176.538	1176.531	5.41	0	22	0.011	1	U	R.YPHEFSGGQR.Q
9548	732.900	1463.785	1463.787	-1.48	0	57	4.7e-06	1	U	K.QVYDLLETVGLSK.E
9913	750.374	1498.733	1498.730	1.99	0	87	2.8e-09	1	U	R.LYDATGGEVIYNGK.D

54. tr|Q8Y8V1 **Mass:** 24025 **Score:** 139 **Matches:** 2(2) **Sequences:** 2(2) **emPAI:** 0.42
Lmo0791 protein OS=Listeria monocytogenes serovar 1/2a (strain ATCC BAA-679 / EGD-e) OX=169963 GN=lmo0791 PE=4 SV=1

Query	Observed	Mr(expt)	Mr(calc)	ppm	Miss	Score	Expect	Rank	Unique	Peptide
8168	673.366	1344.717	1344.714	2.14	0	56	8.1e-06	1	U	K.GVQTELVEELTK.A
8747	695.325	1388.635	1388.642	-4.96	0	98	2.2e-10	1	U	K.AATADPSIASDDQK.M

55. sp|P66054 **Mass:** 14908 **Score:** 137 **Matches:** 3(3) **Sequences:** 3(3) **emPAI:** 1.31
50S ribosomal protein L11 OS=Listeria monocytogenes serovar 1/2a (strain ATCC BAA-679 / EGD-e) OX=169963 GN=rplK PE=3 SV=1

Query	Observed	Mr(expt)	Mr(calc)	ppm	Miss	Score	Expect	Rank	Unique	Peptide
2327	455.292	908.569	908.569	-0.90	0	53	4.9e-06	1	U	K.TPPAAVLLK.K
5077	558.796	1115.578	1115.582	-3.95	0	47	7.4e-05	1	U	R.AQVQEIAETK.M
16047	686.381	2056.120	2056.120	-0.10	0	68	2.3e-07	1	U	R.TADQAGLIIPVVITVFEDR.S

56. sp|P66611 **Mass:** 17805 **Score:** 135 **Matches:** 2(2) **Sequences:** 2(2) **emPAI:** 0.60
30S ribosomal protein S7 OS=Listeria monocytogenes serovar 1/2a (strain ATCC BAA-679 / EGD-e) OX=169963 GN=rpsG PE=3 SV=1

Query	Observed	Mr(expt)	Mr(calc)	ppm	Miss	Score	Expect	Rank	Unique	Peptide
8905	701.376	1400.737	1400.741	-2.77	0	80	3.7e-08	1	U	R.VGGANYQVPIEVR.A
9257	718.829	1435.644	1435.661	-11.94	0	72	1.3e-07	1	U	R.EIMDAANNTGASVK.K

57. sp|P64074 **Mass:** 46444 **Score:** 134 **Matches:** 3(3) **Sequences:** 3(3) **emPAI:** 0.31
Enolase OS=Listeria monocytogenes serovar 1/2a (strain ATCC BAA-679 / EGD-e) OX=169963 GN=eno PE=1 SV=1

Query	Observed	Mr(expt)	Mr(calc)	ppm	Miss	Score	Expect	Rank	Unique	Peptide
2593	464.793	927.572	927.575	-3.56	0	52	1.7e-05	1	U	K.GIANSILIK.V
6051	398.198	1191.573	1191.577	-3.24	0	48	5.2e-05	1	U	K.DAGYKPGEEVK.L
9808	745.833	1489.652	1489.651	0.66	0	66	3.2e-07	1	U	K.LAMDAASSEFYNR.E

58. tr|Q92DC5 **Mass:** 12646 **Score:** 129 **Matches:** 5(5) **Sequences:** 3(3) **emPAI:** 1.66
RsbS protein OS=Listeria monocytogenes serovar 1/2a (strain ATCC BAA-679 / EGD-e) OX=169963 GN=rsbS PE=4 SV=1

Query	Observed	Mr(expt)	Mr(calc)	ppm	Miss	Score	Expect	Rank	Unique	Peptide
727	394.240	786.465	786.467	-2.95	0	41	0.00047	1	U	-.MGIPILK.L 728
960	408.726	815.437	815.439	-2.66	1	53	2.2e-05	1	U	K.LKQELGE.- 961
4373	532.785	1063.555	1063.558	-3.01	0	51	3.3e-05	1	U	K.ILGDVVSMSK.L

59. sp|Q8Y6T6 **Mass:** 22721 **Score:** 125 **Matches:** 7(6) **Sequences:** 6(5) **emPAI:** 1.51
30S ribosomal protein S4 OS=Listeria monocytogenes serovar 1/2a (strain ATCC BAA-679 / EGD-e) OX=169963 GN=rpsD PE=3 SV=1

Query	Observed	Mr(expt)	Mr(calc)	ppm	Miss	Score	Expect	Rank	Unique	Peptide
469	380.717	759.420	759.424	-5.35	0	46	0.00015	1	U	K.LTGSLNR.L 468
2648	466.773	931.532	931.534	-1.71	0	51	2.9e-05	1	U	R.LGISLSGTGK.E
3827	511.740	1021.465	1021.465	-0.13	0	19	0.022	1	U	R.HMYGLTER.Q
7087	633.338	1264.661	1264.630	24.4	0	51	5.4e-05	1	U	K.ISEYGLQQAEK.Q
3231	487.279	1458.815	1458.804	7.37	1	21	0.071	1	U	R.LGISLSGTGKELER.R
14332	923.513	1845.012	1845.000	6.72	0	24	0.013	1	U	R.VDIPSYQVSVGQVISVR.E

60. sp|P02662 **Mass:** 24570 **Score:** 124 **Matches:** 5(5) **Sequences:** 3(3) **emPAI:** 0.67
Alpha-S1-casein (Laboratory-Cont) OS=Bos taurus GN=CSN1S1 PE=1 SV=2

Query	Observed	Mr(expt)	Mr(calc)	ppm	Miss	Score	Expect	Rank	Unique	Peptide
1118	416.196	830.378	830.377	0.97	0	40	0.00011	1	U	K.EDVPSER.Y
7122	634.356	1266.698	1266.697	0.31	0	49	4.8e-05	1	U	R.YLGYLEQLLR.L

8683	692.867	1383.719	1383.723	-2.97	0	49	3.8e-05	1	U	R.FFVAPFPEVFGK.E 8684 8686

---

61. sp|P02663 **Mass:** 26173 **Score:** 117 **Matches:** 4(4) **Sequences:** 4(4) **emPAI:** 0.90
Alpha-S2-casein (Laboratory-Cont) OS=Bos taurus GN=CSN1S2 PE=1 SV=2

Query	Observed	Mr(expt)	Mr(calc)	ppm	Miss	Score	Expect	Rank	Unique	Peptide
373	373.726	745.438	745.449	-14.58	0	30	0.0032	1	U	K.VIPYVR.Y
3310	490.284	978.552	978.554	-1.42	0	29	0.0024	1	U	K.FALPQYLK.T
6099	598.343	1194.671	1194.672	-1.26	0	54	5e-06	1	U	R.NAVPITPTLNR.E
8452	684.347	1366.680	1366.688	-5.67	0	53	1.6e-05	1	U	K.ALNEINQFYQK.F

---

62. sp|Q927L2 **Mass:** 12866 **Score:** 116 **Matches:** 4(4) **Sequences:** 3(3) **emPAI:** 1.62
50S ribosomal protein L22 OS=Listeria monocytogenes serovar 1/2a (strain ATCC BAA-679 / EGD-e) OX=169963 GN=rplV PE=3 SV=1

Query	Observed	Mr(expt)	Mr(calc)	ppm	Miss	Score	Expect	Rank	Unique	Peptide
1335	421.278	840.542	840.543	-1.41	0	42	6.9e-05	1	U	R.IVIDLIR.G
1392	422.740	843.466	843.470	-4.80	0	54	2.8e-05	1	U	R.SASPIIEK.V
4076	521.317	1040.620	1040.623	-2.71	0	46	5e-05	1	U	K.QVGEAIAILK.Y 4075

---

63. tr|Q8Y6W1 **Mass:** 62673 **Score:** 115 **Matches:** 3(3) **Sequences:** 3(3) **emPAI:** 0.22
Pyruvate kinase OS=Listeria monocytogenes serovar 1/2a (strain ATCC BAA-679 / EGD-e) OX=169963 GN=pykA PE=3 SV=1

Query	Observed	Mr(expt)	Mr(calc)	ppm	Miss	Score	Expect	Rank	Unique	Peptide
4369	532.767	1063.519	1063.522	-3.22	0	65	9.3e-07	1	U	R.VSMTPVEGTK.E
5653	582.308	1162.600	1162.598	1.91	0	65	1.5e-06	1	U	K.LEFQTGDVVR.V
13852	598.325	1791.954	1791.962	-4.17	0	21	0.021	1	U	R.GDLGVEIPAEEVPIVQK.E

---

64. tr|Q8Y4C5 **Mass:** 35785 **Score:** 106 **Matches:** 3(3) **Sequences:** 3(3) **emPAI:** 0.42
Mbl protein OS=Listeria monocytogenes serovar 1/2a (strain ATCC BAA-679 / EGD-e) OX=169963 GN=mbl PE=4 SV=1

Query	Observed	Mr(expt)	Mr(calc)	ppm	Miss	Score	Expect	Rank	Unique	Peptide
3286	489.269	976.524	976.534	-10.47	0	42	0.00029	1	U	K.YNLLIGER.T
5094	559.796	1117.578	1117.566	11.5	0	27	0.0068	2	U	K.QVFLEEEPK.V
10371	768.943	1535.871	1535.867	2.61	0	72	1.1e-07	1	U	R.GIVVNEPAVVAINNK.T

---

65. tr|Q926Y9 **Mass:** 52637 **Score:** 106 **Matches:** 4(4) **Sequences:** 4(4) **emPAI:** 0.38
Inosine-5'-monophosphate dehydrogenase OS=Listeria monocytogenes serovar 1/2a (strain ATCC BAA-679 / EGD-e) OX=169963 GN=guaB PE=1 SV=1

Query	Observed	Mr(expt)	Mr(calc)	ppm	Miss	Score	Expect	Rank	Unique	Peptide
643	391.200	780.385	780.402	-20.86	0	17	0.056	1	U	K.YSGDIVK.A
1963	443.277	884.539	884.544	-6.12	0	34	0.0015	1	U	K.LVGILTNR.D
5728	584.345	1166.676	1166.691	-13.03	0	42	0.00011	1	U	K.LPLVDEAGILK.G
10094	758.915	1515.816	1515.818	-1.36	0	63	1.5e-06	1	U	K.EGLTFDDVLLVPAK.S

---

66. sp|P66352 **Mass:** 13834 **Score:** 105 **Matches:** 2(2) **Sequences:** 2(2) **emPAI:** 0.82
30S ribosomal protein S11 OS=Listeria monocytogenes serovar 1/2a (strain ATCC BAA-679 / EGD-e) OX=169963 GN=rpsK PE=3 SV=1

Query	Observed	Mr(expt)	Mr(calc)	ppm	Miss	Score	Expect	Rank	Unique	Peptide
7331	642.879	1283.743	1283.745	-1.56	0	66	4.6e-07	1	U	R.ALQAAGLEVTAIK.D
9867	748.853	1495.692	1495.698	-3.54	0	55	7.6e-06	1	U	K.STPFAAQMAAESAAK.S

---

67. tr|Q7AP76 **Mass:** 43599 **Score:** 103 **Matches:** 3(3) **Sequences:** 3(3) **emPAI:** 0.34
GbuA protein OS=Listeria monocytogenes serovar 1/2a (strain ATCC BAA-679 / EGD-e) OX=169963 GN=gbuA PE=4 SV=1

Query	Observed	Mr(expt)	Mr(calc)	ppm	Miss	Score	Expect	Rank	Unique	Peptide
3427	495.260	988.505	988.519	-14.50	0	48	7.5e-05	1	U	K.ETGATIGVNK.A
9669	739.344	1476.674	1476.677	-1.97	0	39	0.00027	1	U	K.DMQDQLLDLQDK.M
18303	857.086	2568.237	2568.234	0.96	0	51	2.7e-05	1	U	R.DGSVVQTGSPEEILAHPANEYVEK.F

---

68. tr|Q8Y701 **Mass:** 12007 **Score:** 95 **Matches:** 2(2) **Sequences:** 2(2) **emPAI:** 0.99
Lmo1529 protein OS=Listeria monocytogenes serovar 1/2a (strain ATCC BAA-679 / EGD-e) OX=169963 GN=lmo1529 PE=4 SV=1

Query	Observed	Mr(expt)	Mr(calc)	ppm	Miss	Score	Expect	Rank	Unique	Peptide
6886	625.804	1249.594	1249.597	-2.45	0	76	9.5e-08	1	U	K.EVQNMQSSLAK.G
9991	502.924	1505.749	1505.751	-1.37	1	38	0.00058	1	U	R.QKEVQNMQSSLAK.G

---

69. sp|Q8Y9L8 **Mass:** 34437 **Score:** 95 **Matches:** 3(2) **Sequences:** 3(2) **emPAI:** 0.28
Putative ribose-phosphate pyrophosphokinase 2 OS=Listeria monocytogenes serovar 1/2a (strain ATCC BAA-679 / EGD-e) OX=169963 GN=prs2 PE=3 SV=1

Query	Observed	Mr(expt)	Mr(calc)	ppm	Miss	Score	Expect	Rank	Unique	Peptide
3223	487.268	972.522	972.524	-2.23	0	19	0.058	2	U	K.INIEESIR.G
3243	487.775	973.536	973.538	-1.98	0	48	8.1e-05	1	U	K.LMANLIQR.A
4781	547.837	1093.660	1093.661	-0.67	0	63	4.9e-07	1	U	R.LNAPIAILNR.K

---

70. sp|Q8Y444 **Mass:** 19388 **Score:** 95 **Matches:** 5(5) **Sequences:** 5(5) **emPAI:** 1.93
50S ribosomal protein L6 OS=Listeria monocytogenes serovar 1/2a (strain ATCC BAA-679 / EGD-e) OX=169963 GN=rplF PE=3 SV=1

Query	Observed	Mr(expt)	Mr(calc)	ppm	Miss	Score	Expect	Rank	Unique	Peptide
3804	510.296	1018.577	1018.581	-4.11	0	34	0.001	1	U	K.LELIGVGYR.A
4728	545.793	1089.572	1089.571	1.11	0	42	0.00036	1	U	K.EFNPEITIK.I
5465	574.340	1146.664	1146.676	-10.10	1	37	0.00046	1	U	K.KLELIGVGYR.A
10934	791.446	1580.878	1580.877	0.11	0	21	0.012	1	U	K.GVDIEVPANTQVIVK.G
13208	580.304	1737.892	1737.861	17.7	0	28	0.0036	1	U	R.AILNNMVVGVSEGYEK.K

---

71. sp|Q8Y450 **Mass:** 15205 **Score:** 93 **Matches:** 4(4) **Sequences:** 3(3) **emPAI:** 1.27
50S ribosomal protein L17 OS=Listeria monocytogenes serovar 1/2a (strain ATCC BAA-679 / EGD-e) OX=169963 GN=rplQ PE=3 SV=1

Query	Observed	Mr(expt)	Mr(calc)	ppm	Miss	Score	Expect	Rank	Unique	Peptide

2647	466.746	931.477	931.476	1.05	0	39	0.0007	1	U	K.LFDDVAPR.Y 2646
7433	646.347	1290.680	1290.682	-1.67	0	54	1.6e-05	1	U	R.DLATDLIVFER.I
8712	693.375	1384.736	1384.738	-1.64	1	32	0.0021	1	U	R.RGDGAPMVIIELV.-

72. tr|Q8Y5M4 **Mass:** 46016 **Score:** 92 **Matches:** 3(3) **Sequences:** 3(3) **emPAI:** 0.32
Cell division protein FtsA OS=Listeria monocytogenes serovar 1/2a (strain ATCC BAA-679 / EGD-e) OX=169963 GN=ftsA PE=3 SV=1

Query	Observed	Mr(expt)	Mr(calc)	ppm	Miss	Score	Expect	Rank	Unique	Peptide
10500	774.398	1546.781	1546.759	14.1	0	39	0.00084	1	U	K.DLSLGLNTSTANADR.V
11782	824.434	1646.853	1646.888	-21.50	0	43	0.00015	1	U	R.LTYTGVIPVGGDNITK.D
17709	805.097	2412.270	2412.254	6.61	0	41	0.00014	1	U	R.EIINTIPDQFVVDGLTGITDPR.G

73. sp|P66330 **Mass:** 11674 **Score:** 91 **Matches:** 2(2) **Sequences:** 2(2) **emPAI:** 1.03
30S ribosomal protein S10 OS=Listeria monocytogenes serovar 1/2a (strain ATCC BAA-679 / EGD-e) OX=169963 GN=rpsJ PE=3 SV=1

Query	Observed	Mr(expt)	Mr(calc)	ppm	Miss	Score	Expect	Rank	Unique	Peptide
2220	452.240	902.466	902.471	-5.38	0	56	1.2e-05	1	U	R.ILDQSAEK.I
9305	720.380	1438.745	1438.767	-14.98	0	53	1.4e-05	1	U	R.SGASVSGPIPLPTEK.S

74. sp|Q8Y447 **Mass:** 15772 **Score:** 91 **Matches:** 1(1) **Sequences:** 1(1) **emPAI:** 0.30
50S ribosomal protein L15 OS=Listeria monocytogenes serovar 1/2a (strain ATCC BAA-679 / EGD-e) OX=169963 GN=rplO PE=3 SV=1

Query	Observed	Mr(expt)	Mr(calc)	ppm	Miss	Score	Expect	Rank	Unique	Peptide
8053	667.366	1332.717	1332.719	-1.83	0	91	2.6e-09	1	U	R.LGFEGGQLPLFR.R

75. sp|Q8YAA3 **Mass:** 12462 **Score:** 91 **Matches:** 2(2) **Sequences:** 2(2) **emPAI:** 0.94
50S ribosomal protein L7/L12 OS=Listeria monocytogenes serovar 1/2a (strain ATCC BAA-679 / EGD-e) OX=169963 GN=rplL PE=3 SV=1

Query	Observed	Mr(expt)	Mr(calc)	ppm	Miss	Score	Expect	Rank	Unique	Peptide
7541	650.377	1298.739	1298.745	-4.28	0	63	9.1e-07	1	U	M.ALNIEEIIASVK.E
7996	665.368	1328.721	1328.719	1.42	0	44	0.00013	1	U	K.EASVLELNDLVK.A

76. sp|P0DJP0 **Mass:** 37971 **Score:** 90 **Matches:** 2(2) **Sequences:** 2(2) **emPAI:** 0.25
Protein RecA OS=Listeria monocytogenes serovar 1/2a (strain ATCC BAA-679 / EGD-e) OX=169963 GN=recA PE=3 SV=1

Query	Observed	Mr(expt)	Mr(calc)	ppm	Miss	Score	Expect	Rank	Unique	Peptide
280	365.725	729.435	729.438	-5.28	0	38	0.00078	1	U	K.LSGVINK.S
10485	773.884	1545.754	1545.761	-4.68	0	71	2.8e-07	1	U	K.VGVMFGNPEITPGGR.A

77. tr|Q8Y980 **Mass:** 33268 **Score:** 89 **Matches:** 2(2) **Sequences:** 2(2) **emPAI:** 0.29
Lmo0653 protein OS=Listeria monocytogenes serovar 1/2a (strain ATCC BAA-679 / EGD-e) OX=169963 GN=lmo0653 PE=4 SV=1

Query	Observed	Mr(expt)	Mr(calc)	ppm	Miss	Score	Expect	Rank	Unique	Peptide
11265	808.396	1614.778	1614.785	-3.99	0	86	6.8e-09	1	U	R.DNPNISALDAITESR.H
15219	958.969	1915.923	1915.941	-9.50	0	21	0.025	1	U	K.EPETPVVPEAPETPEAPK.D

78. tr|Q8Y7B5 **Mass:** 51170 **Score:** 88 **Matches:** 4(4) **Sequences:** 4(4) **emPAI:** 0.39
Dihydrolipoyl dehydrogenase OS=Listeria monocytogenes serovar 1/2a (strain ATCC BAA-679 / EGD-e) OX=169963 GN=lmo1371 PE=3 SV=1

Query	Observed	Mr(expt)	Mr(calc)	ppm	Miss	Score	Expect	Rank	Unique	Peptide
354	372.241	742.468	742.470	-3.23	0	22	0.02	1	U	K.ILVSVGR.S
5200	563.261	1124.508	1124.499	8.76	0	47	5.9e-05	1	U	K.GEEQTFTADK.I
5753	585.347	1168.679	1168.693	-11.94	0	23	0.0065	1	U	K.NLIIATGSKPR.T
7529	649.849	1297.683	1297.655	20.9	0	44	0.00011	1	U	K.ALVYGESDGFIK.I

79. sp|Q9AGE7 **Mass:** 10058 **Score:** 87 **Matches:** 2(2) **Sequences:** 2(2) **emPAI:** 1.25
10 kDa chaperonin OS=Listeria monocytogenes serovar 1/2a (strain ATCC BAA-679 / EGD-e) OX=169963 GN=groS PE=3 SV=2

Query	Observed	Mr(expt)	Mr(calc)	ppm	Miss	Score	Expect	Rank	Unique	Peptide
3431	495.279	988.542	988.544	-1.58	0	51	5.5e-05	1	U	R.ESDILAITK.-
5610	579.816	1157.618	1157.629	-9.72	0	55	1.2e-05	1	U	K.TASGIVLPDSAK.E

80. tr|Q8YAH4 **Mass:** 62089 **Score:** 86 **Matches:** 2(2) **Sequences:** 2(2) **emPAI:** 0.15
Lmo0152 protein OS=Listeria monocytogenes serovar 1/2a (strain ATCC BAA-679 / EGD-e) OX=169963 GN=lmo0152 PE=4 SV=1

Query	Observed	Mr(expt)	Mr(calc)	ppm	Miss	Score	Expect	Rank	Unique	Peptide
9204	716.377	1430.740	1430.741	-0.40	0	53	1.3e-05	1	U	K.AQFEDNLPGVTIK.V
9568	734.358	1466.701	1466.679	14.7	0	49	3.1e-05	1	U	K.DFVQNPTTGADFR.T

81. tr|Q8Y4R3 **Mass:** 40303 **Score:** 85 **Matches:** 3(3) **Sequences:** 3(3) **emPAI:** 0.37
Lmo2371 protein OS=Listeria monocytogenes serovar 1/2a (strain ATCC BAA-679 / EGD-e) OX=169963 GN=lmo2371 PE=4 SV=1

Query	Observed	Mr(expt)	Mr(calc)	ppm	Miss	Score	Expect	Rank	Unique	Peptide
1776	436.745	871.476	871.476	-0.68	0	49	3.6e-05	1	U	K.VDALDAIR.G
8430	682.854	1363.693	1363.698	-4.08	0	37	0.001	1	U	R.ELTVVGFTENQK.Y
8751	695.349	1388.683	1388.697	-9.92	0	38	0.0012	1	U	K.IGDVLMDDILNR.E

82. tr|Q8Y6M1 **Mass:** 72024 **Score:** 82 **Matches:** 4(4) **Sequences:** 3(3) **emPAI:** 0.19
AnsB protein OS=Listeria monocytogenes serovar 1/2a (strain ATCC BAA-679 / EGD-e) OX=169963 GN=ansB PE=4 SV=1

Query	Observed	Mr(expt)	Mr(calc)	ppm	Miss	Score	Expect	Rank	Unique	Peptide
5934	592.280	1182.545	1182.537	6.83	0	25	0.0072	1	U	R.MTMANSLEVR.V 5935
9713	494.260	1479.758	1479.757	0.49	1	49	4e-05	1	U	R.ITEITGADKETFR.Q
3972	517.288	1548.844	1548.830	8.82	1	37	0.0013	1	U	R.VPFLDKEVYNVAR.N

83. tr|Q8Y8E2 **Mass:** 21181 **Score:** 82 **Matches:** 2(2) **Sequences:** 2(2) **emPAI:** 0.48
Listeria epitope LemA OS=Listeria monocytogenes serovar 1/2a (strain ATCC BAA-679 / EGD-e) OX=169963 GN=lemA PE=4 SV=1

Query	Observed	Mr(expt)	Mr(calc)	ppm	Miss	Score	Expect	Rank	Unique	Peptide

4770	547.732	1093.449	1093.453	-3.74	0	34	0.00036	1	U	K.MMEVPADNR.Q
9139	712.376	1422.738	1422.739	-1.26	0	62	1.5e-06	1	U	K.SIFALGEAYPDLK.A

84. sp|Q8Y699 **Mass:** 10359 **Score:** 81 **Matches:** 2(2) **Sequences:** 2(2) **emPAI:** 1.21
30S ribosomal protein S16 OS=Listeria monocytogenes serovar 1/2a (strain ATCC BAA-679 / EGD-e) OX=169963 GN=rpsP PE=3 SV=1

Query	Observed	Mr(expt)	Mr(calc)	ppm	Miss	Score	Expect	Rank	Unique	Peptide
458	380.215	758.415	758.429	-18.25	0	56	3.3e-05	1	U	R.IVVADSR.F
15754	663.358	1987.051	1987.051	0.02	0	46	6.9e-05	1	U	R.SIETIGTYNPLLDPVEVK.I

85. tr|Q8YAM1 **Mass:** 27506 **Score:** 79 **Matches:** 2(2) **Sequences:** 2(2) **emPAI:** 0.36
Lmo0097 protein OS=Listeria monocytogenes serovar 1/2a (strain ATCC BAA-679 / EGD-e) OX=169963 GN=lmo0097 PE=4 SV=1

Query	Observed	Mr(expt)	Mr(calc)	ppm	Miss	Score	Expect	Rank	Unique	Peptide
5321	567.768	1133.522	1133.524	-1.95	0	43	0.00011	1	U	R.DPLGDILNDY.-
8615	460.927	1379.758	1379.760	-1.07	0	52	1.1e-05	1	U	R.TLAVPIVHLMDR.A

86. tr|Q8Y580 **Mass:** 38117 **Score:** 78 **Matches:** 2(2) **Sequences:** 2(2) **emPAI:** 0.25
Lmo2194 protein OS=Listeria monocytogenes serovar 1/2a (strain ATCC BAA-679 / EGD-e) OX=169963 GN=lmo2194 PE=3 SV=1

Query	Observed	Mr(expt)	Mr(calc)	ppm	Miss	Score	Expect	Rank	Unique	Peptide
2539	463.213	924.412	924.412	-0.03	0	47	2.2e-05	1	U	R.VDNFMQR.V
9580	490.253	1467.736	1467.736	0.33	0	47	7.1e-05	1	U	R.FQPAHILDAEAEK.I

87. tr|Q8Y7N8 **Mass:** 19292 **Score:** 77 **Matches:** 3(3) **Sequences:** 3(3) **emPAI:** 0.91
Lmo1236 protein OS=Listeria monocytogenes serovar 1/2a (strain ATCC BAA-679 / EGD-e) OX=169963 GN=lmo1236 PE=4 SV=1

Query	Observed	Mr(expt)	Mr(calc)	ppm	Miss	Score	Expect	Rank	Unique	Peptide
5779	586.335	1170.655	1170.661	-4.87	0	47	8.4e-05	1	U	R.LLLEGETVAAR.F
7554	650.884	1299.754	1299.755	-0.49	0	33	0.0011	1	U	K.ALVPSFGLDLLR.D
10673	521.288	1560.843	1560.815	18.4	0	33	0.0019	1	U	K.LEAGFIAEQLQSQK.K

88. tr|Q927C3 **Mass:** 52821 **Score:** 74 **Matches:** 1(1) **Sequences:** 1(1) **emPAI:** 0.08
CydA protein OS=Listeria monocytogenes serovar 1/2a (strain ATCC BAA-679 / EGD-e) OX=169963 GN=cydA PE=4 SV=1

Query	Observed	Mr(expt)	Mr(calc)	ppm	Miss	Score	Expect	Rank	Unique	Peptide
15732	661.996	1982.968	1982.947	10.2	1	74	1.2e-07	1	U	K.AEISVDPFDKGDESFVTK.-

89. tr|Q8Y693 **Mass:** 36441 **Score:** 74 **Matches:** 1(1) **Sequences:** 1(1) **emPAI:** 0.12
Signal recognition particle receptor FtsY OS=Listeria monocytogenes serovar 1/2a (strain ATCC BAA-679 / EGD-e) OX=169963 GN=ftsY PE=3 SV=1

Query	Observed	Mr(expt)	Mr(calc)	ppm	Miss	Score	Expect	Rank	Unique	Peptide
5650	582.289	1162.564	1162.583	-16.05	0	74	1.4e-07	1	U	K.ITQQTDSVSGK.F

90. tr|Q8Y6Y7 **Mass:** 29219 **Score:** 73 **Matches:** 2(2) **Sequences:** 2(2) **emPAI:** 0.33
Site-determining protein OS=Listeria monocytogenes serovar 1/2a (strain ATCC BAA-679 / EGD-e) OX=169963 GN=minD PE=3 SV=1

Query	Observed	Mr(expt)	Mr(calc)	ppm	Miss	Score	Expect	Rank	Unique	Peptide
8306	678.396	1354.777	1354.782	-3.56	0	47	4.6e-05	1	U	K.AIVVTTPEISAVR.D
14002	904.475	1806.936	1806.940	-2.47	0	42	0.00045	1	U	R.FDDLLFLLPAAQTTDK.N

91. tr|Q8Y5F0 **Mass:** 28353 **Score:** 71 **Matches:** 4(4) **Sequences:** 4(4) **emPAI:** 0.81
Lmo2114 protein OS=Listeria monocytogenes serovar 1/2a (strain ATCC BAA-679 / EGD-e) OX=169963 GN=lmo2114 PE=4 SV=1

Query	Observed	Mr(expt)	Mr(calc)	ppm	Miss	Score	Expect	Rank	Unique	Peptide
566	386.251	770.488	770.490	-2.50	0	22	0.012	1	U	K.ILDVLAK.L
586	387.241	772.468	772.469	-1.54	0	22	0.025	1	U	K.LAIISEK.F
4900	552.306	1102.597	1102.598	-1.01	0	31	0.0035	1	U	K.SATNLLESLR.D
6084	597.873	1193.732	1193.738	-4.98	0	45	2.9e-05	1	U	R.ENIILPLALAK.R

92. tr|Q8Y8P9 **Mass:** 53376 **Score:** 70 **Matches:** 2(2) **Sequences:** 2(2) **emPAI:** 0.17
Lmo0847 protein OS=Listeria monocytogenes serovar 1/2a (strain ATCC BAA-679 / EGD-e) OX=169963 GN=lmo0847 PE=3 SV=1

Query	Observed	Mr(expt)	Mr(calc)	ppm	Miss	Score	Expect	Rank	Unique	Peptide
2132	448.286	894.558	894.565	-8.33	0	40	0.0001	1	U	K.VVLPQAIR.M
3259	488.285	974.555	974.555	0.15	0	46	9e-05	1	U	K.FNAGLVNIK.A

93. tr|Q8YAB6 **Mass:** 91166 **Score:** 70 **Matches:** 2(2) **Sequences:** 2(2) **emPAI:** 0.10
Endopeptidase Clp ATP-binding chain C OS=Listeria monocytogenes serovar 1/2a (strain ATCC BAA-679 / EGD-e) OX=169963 GN=clpC PE=3 SV=1

Query	Observed	Mr(expt)	Mr(calc)	ppm	Miss	Score	Expect	Rank	Unique	Peptide
8341	679.367	1356.719	1356.725	-4.48	0	61	3.2e-06	1	U	R.VAITDEALEAAVR.L
8483	685.392	1368.770	1368.772	-2.09	0	27	0.0061	1	U	K.TAIAEGLAQQIVR.N

94. tr|Q8Y5W4 **Mass:** 25820 **Score:** 70 **Matches:** 1(1) **Sequences:** 1(1) **emPAI:** 0.18
Lmo1941 protein OS=Listeria monocytogenes serovar 1/2a (strain ATCC BAA-679 / EGD-e) OX=169963 GN=lmo1941 PE=4 SV=1

Query	Observed	Mr(expt)	Mr(calc)	ppm	Miss	Score	Expect	Rank	Unique	Peptide
7686	655.318	1308.622	1308.631	-6.95	0	70	3e-07	1	U	R.STYGQAGAAAGVEK.I

95. sp|P47847 **Mass:** 94828 **Score:** 69 **Matches:** 1(1) **Sequences:** 1(1) **emPAI:** 0.05
Protein translocase subunit SecA 1 OS=Listeria monocytogenes serovar 1/2a (strain ATCC BAA-679 / EGD-e) OX=169963 GN=secA1 PE=3 SV=2

Query	Observed	Mr(expt)	Mr(calc)	ppm	Miss	Score	Expect	Rank	Unique	Peptide
8741	694.866	1387.718	1387.719	-0.91	0	69	4.5e-07	1	U	R.TSEDIQNLILDK.I

96. sp|P02666 **Mass:** 25148 **Score:** 69 **Matches:** 4(4) **Sequences:** 4(4) **emPAI:** 0.95
Beta-casein (Laboratory-Cont) OS=Bos taurus GN=CSN2 PE=1 SV=2

Query	Observed	Mr(expt)	Mr(calc)	ppm	Miss	Score	Expect	Rank	Unique	Peptide
347	371.728	741.442	741.443	-1.18	0	24	0.0062	1	U	R.GPFPIIV.-
506	382.690	763.366	763.357	11.6	0	26	0.0072	1	U	K.EMPFPK.Y
639	390.751	779.488	779.491	-3.28	0	40	0.00011	1	U	K.VLPVPQK.A
1112	415.726	829.437	829.445	-9.71	0	26	0.009	1	U	K.AVPYPQR.D

---

97. sp|Q8Y458 **Mass:** 16190 **Score:** 68 **Matches:** 1(1) **Sequences:** 1(1) **emPAI:** 0.29
50S ribosomal protein L13 OS=Listeria monocytogenes serovar 1/2a (strain ATCC BAA-679 / EGD-e) OX=169963 GN=rplM PE=3 SV=1

Query	Observed	Mr(expt)	Mr(calc)	ppm	Miss	Score	Expect	Rank	Unique	Peptide
4515	537.810	1073.606	1073.608	-2.36	0	68	6.3e-07	1	U	R.LSSEVASILR.G

---

98. tr|Q8YA67 **Mass:** 52809 **Score:** 68 **Matches:** 2(2) **Sequences:** 2(2) **emPAI:** 0.17
Lmo0292 protein OS=Listeria monocytogenes serovar 1/2a (strain ATCC BAA-679 / EGD-e) OX=169963 GN=lmo0292 PE=4 SV=1

Query	Observed	Mr(expt)	Mr(calc)	ppm	Miss	Score	Expect	Rank	Unique	Peptide
5505	576.292	1150.570	1150.572	-1.33	0	21	0.04	1	U	K.VSVDTTSDVTK.A
7051	421.888	1262.641	1262.637	3.63	0	63	2.1e-06	1	U	K.ESEPARPAPGPR.R

---

99. sp|Q8Y421 **Mass:** 76973 **Score:** 66 **Matches:** 3(3) **Sequences:** 3(3) **emPAI:** 0.18
Elongation factor G OS=Listeria monocytogenes serovar 1/2a (strain ATCC BAA-679 / EGD-e) OX=169963 GN=fusA PE=3 SV=1

Query	Observed	Mr(expt)	Mr(calc)	ppm	Miss	Score	Expect	Rank	Unique	Peptide
1741	435.247	868.480	868.481	-1.01	0	21	0.046	1	U	R.ILFYTGR.I
3457	496.757	991.499	991.509	-10.10	0	55	1.3e-05	1	U	R.QATTYGVPR.V
4550	539.272	1076.529	1076.514	14.0	0	29	0.0046	1	U	K.LAEEDPTFR.A

---

100. tr|Q8Y4E0 **Mass:** 25903 **Score:** 64 **Matches:** 2(2) **Sequences:** 2(2) **emPAI:** 0.38
FtsE protein OS=Listeria monocytogenes serovar 1/2a (strain ATCC BAA-679 / EGD-e) OX=169963 GN=ftsE PE=4 SV=1

Query	Observed	Mr(expt)	Mr(calc)	ppm	Miss	Score	Expect	Rank	Unique	Peptide
1769	436.244	870.474	870.492	-20.52	0	19	0.035	1	U	R.VIAIENGR.I
7387	644.862	1287.710	1287.711	-1.01	0	63	1.8e-06	1	U	R.VMEVLDLVNLK.H

---

101. tr|Q8YAM2 **Mass:** 34972 **Score:** 64 **Matches:** 2(2) **Sequences:** 2(2) **emPAI:** 0.27
Lmo0096 protein OS=Listeria monocytogenes serovar 1/2a (strain ATCC BAA-679 / EGD-e) OX=169963 GN=lmo0096 PE=4 SV=1

Query	Observed	Mr(expt)	Mr(calc)	ppm	Miss	Score	Expect	Rank	Unique	Peptide
5172	561.818	1121.621	1121.644	-21.21	0	21	0.021	1	U	K.LIEQAAPPGVK.A
10272	764.421	1526.827	1526.846	-12.44	0	59	2.8e-06	1	U	K.ALLLFENPQDVLR.A

---

102. tr|Q8YA11 **Mass:** 54486 **Score:** 61 **Matches:** 5(3) **Sequences:** 5(3) **emPAI:** 0.26
Lmo0355 protein OS=Listeria monocytogenes serovar 1/2a (strain ATCC BAA-679 / EGD-e) OX=169963 GN=lmo0355 PE=4 SV=1

Query	Observed	Mr(expt)	Mr(calc)	ppm	Miss	Score	Expect	Rank	Unique	Peptide
1062	413.231	824.447	824.443	5.07	0	22	0.049	1	U	K.APYYAIK.I
1419	423.727	845.439	845.449	-12.64	0	18	0.071	2	U	K.TIDELAGK.I
3681	504.279	1006.543	1006.545	-1.78	0	17	0.075	1	U	K.SYLIGEAVR.G
4880	551.308	1100.602	1100.623	-19.42	0	23	0.024	1	U	K.IPVFVNADVK.E
8815	697.354	1392.694	1392.692	1.47	0	50	8.5e-05	1	U	K.LNNLTITGGMSEK.R

---

103. sp|P66623 **Mass:** 14635 **Score:** 59 **Matches:** 1(1) **Sequences:** 1(1) **emPAI:** 0.33
30S ribosomal protein S8 OS=Listeria monocytogenes serovar 1/2a (strain ATCC BAA-679 / EGD-e) OX=169963 GN=rpsH PE=3 SV=1

Query	Observed	Mr(expt)	Mr(calc)	ppm	Miss	Score	Expect	Rank	Unique	Peptide
8833	697.853	1393.691	1393.691	-0.27	0	59	4.5e-06	1	U	M.VMTDPIADFLTR.I

---

104. tr|Q7AP78 **Mass:** 48456 **Score:** 59 **Matches:** 1(1) **Sequences:** 1(1) **emPAI:** 0.09
Protein DltD OS=Listeria monocytogenes serovar 1/2a (strain ATCC BAA-679 / EGD-e) OX=169963 GN=dltD PE=3 SV=1

Query	Observed	Mr(expt)	Mr(calc)	ppm	Miss	Score	Expect	Rank	Unique	Peptide
10701	782.929	1563.843	1563.851	-4.94	0	59	4.3e-06	1	U	R.LLSFQVVQSDSTLK.K

---

105. sp|P0DJM1 **Mass:** 41508 **Score:** 57 **Matches:** 2(2) **Sequences:** 2(2) **emPAI:** 0.23
Chaperone protein DnaJ OS=Listeria monocytogenes serovar 1/2a (strain ATCC BAA-679 / EGD-e) OX=169963 GN=dnaJ PE=3 SV=1

Query	Observed	Mr(expt)	Mr(calc)	ppm	Miss	Score	Expect	Rank	Unique	Peptide
7370	644.308	1286.601	1286.604	-2.07	0	24	0.0079	1	U	K.VPAGVNDGQQMR.V
9078	709.857	1417.699	1417.695	2.49	0	49	4.3e-05	1	U	K.GSINVEQNTPFGR.V

---

106. sp|Q9RLT9 **Mass:** 132692 **Score:** 56 **Matches:** 3(3) **Sequences:** 3(3) **emPAI:** 0.10
DNA-directed RNA polymerase subunit beta OS=Listeria monocytogenes serovar 1/2a (strain ATCC BAA-679 / EGD-e) OX=169963 GN=rpoB PE=3 SV=1

Query	Observed	Mr(expt)	Mr(calc)	ppm	Miss	Score	Expect	Rank	Unique	Peptide
395	375.226	748.437	748.448	-15.51	0	19	0.037	1	U	R.VYIVQK.R
3441	495.760	989.505	989.493	12.1	0	26	0.0073	1	U	R.DANYAAPLR.V
4073	521.304	1040.593	1040.598	-4.83	0	46	9.1e-05	1	U	R.LSALGPGGLTR.E

---

107. sp|P66383 **Mass:** 13701 **Score:** 55 **Matches:** 2(2) **Sequences:** 2(2) **emPAI:** 0.83
30S ribosomal protein S13 OS=Listeria monocytogenes serovar 1/2a (strain ATCC BAA-679 / EGD-e) OX=169963 GN=rpsM PE=3 SV=1

Query	Observed	Mr(expt)	Mr(calc)	ppm	Miss	Score	Expect	Rank	Unique	Peptide
1071	413.741	825.467	825.471	-5.17	0	46	3.2e-05	1	U	R.IAGVDVPR.E
2914	475.768	949.521	949.523	-2.05	0	26	0.014	1	U	R.LIEIGSYR.G

---

108. tr|Q8Y703 **Mass:** 82563 **Score:** 55 **Matches:** 2(2) **Sequences:** 2(2) **emPAI:** 0.11
Multifunctional fusion protein OS=Listeria monocytogenes serovar 1/2a (strain ATCC BAA-679 / EGD-e) OX=169963 GN=secD PE=3 SV=1

Query	Observed	Mr(expt)	Mr(calc)	ppm	Miss	Score	Expect	Rank	Unique	Peptide

3955	516.762	1031.509	1031.525	-15.05	0	43	0.00022	1	U	K.GTLSQNDVAK.F
8340	679.363	1356.712	1356.700	9.13	0	30	0.0032	1	U	R.VQLAGVTDQAEAR.K

109. tr|Q8Y498 **Mass:** 30219 **Score:** 54 **Matches:** 1(1) **Sequences:** 1(1) **emPAI:** 0.15
FbaA protein OS=Listeria monocytogenes serovar 1/2a (strain ATCC BAA-679 / EGD-e) OX=169963 GN=fbaA PE=3 SV=1

Query	Observed	Mr(expt)	Mr(calc)	ppm	Miss	Score	Expect	Rank	Unique	Peptide
4635	541.334	1080.653	1080.654	-1.03	0	54	4e-06	1	U	K.VIGPGVDAIIK.T

110. sp|Q8Y443 **Mass:** 11172 **Score:** 52 **Matches:** 2(2) **Sequences:** 2(2) **emPAI:** 1.08
50S ribosomal protein L24 OS=Listeria monocytogenes serovar 1/2a (strain ATCC BAA-679 / EGD-e) OX=169963 GN=rplX PE=3 SV=1

Query	Observed	Mr(expt)	Mr(calc)	ppm	Miss	Score	Expect	Rank	Unique	Peptide
369	373.233	744.451	744.453	-3.03	0	30	0.0036	1	U	K.VLAAFPK.K
5297	566.325	1130.636	1130.637	-1.00	0	40	0.00039	1	U	R.VLIEGINMVK.K

111. tr|Q8Y5Q0 **Mass:** 61673 **Score:** 52 **Matches:** 1(1) **Sequences:** 1(1) **emPAI:** 0.07
AlsS protein OS=Listeria monocytogenes serovar 1/2a (strain ATCC BAA-679 / EGD-e) OX=169963 GN=alsS PE=3 SV=1

Query	Observed	Mr(expt)	Mr(calc)	ppm	Miss	Score	Expect	Rank	Unique	Peptide
3888	514.311	1026.608	1026.626	-17.62	0	52	1.4e-05	1	U	K.LPVLLLGMR.A

112. tr|Q8Y6S2 **Mass:** 38795 **Score:** 52 **Matches:** 1(1) **Sequences:** 1(1) **emPAI:** 0.11
Lmo1611 protein OS=Listeria monocytogenes serovar 1/2a (strain ATCC BAA-679 / EGD-e) OX=169963 GN=lmo1611 PE=3 SV=1

Query	Observed	Mr(expt)	Mr(calc)	ppm	Miss	Score	Expect	Rank	Unique	Peptide
9420	725.891	1449.767	1449.773	-4.23	0	52	2.7e-05	1	U	K.SQFGQIGQGFLLR.I

113. sp|Q8Y459 **Mass:** 14363 **Score:** 51 **Matches:** 1(1) **Sequences:** 1(1) **emPAI:** 0.34
30S ribosomal protein S9 OS=Listeria monocytogenes serovar 1/2a (strain ATCC BAA-679 / EGD-e) OX=169963 GN=rpsI PE=3 SV=1

Query	Observed	Mr(expt)	Mr(calc)	ppm	Miss	Score	Expect	Rank	Unique	Peptide
10752	523.644	1567.909	1567.909	0.24	0	51	1e-05	1	U	R.ALLQVAPEYRPALK.S

114. tr|Q929C7 **Mass:** 24060 **Score:** 50 **Matches:** 2(2) **Sequences:** 2(2) **emPAI:** 0.42
Lmo2248 protein OS=Listeria monocytogenes serovar 1/2a (strain ATCC BAA-679 / EGD-e) OX=169963 GN=lmo2248 PE=4 SV=1

Query	Observed	Mr(expt)	Mr(calc)	ppm	Miss	Score	Expect	Rank	Unique	Peptide
10870	788.922	1575.829	1575.851	-13.69	0	48	5.9e-05	1	U	K.FIQAIQASTVEIEK.A
11347	406.698	1622.761	1622.761	-0.04	1	20	0.026	1	U	K.IKEYETAGDSMVHK.M

115. sp|Q8Y5G2 **Mass:** 31814 **Score:** 49 **Matches:** 2(2) **Sequences:** 2(2) **emPAI:** 0.30
Pyridoxal 5'-phosphate synthase subunit PdxS OS=Listeria monocytogenes serovar 1/2a (strain ATCC BAA-679 / EGD-e) OX=169963 GN=pdxS PE=3 SV=1

Query	Observed	Mr(expt)	Mr(calc)	ppm	Miss	Score	Expect	Rank	Unique	Peptide
9379	723.870	1445.725	1445.718	4.80	0	27	0.0056	1	U	K.GGVIMDVVNAEQAK.I
10270	509.942	1526.805	1526.805	-0.05	1	39	0.00038	1	U	R.TKGEPGTGNIVEAVR.H

116. sp|Q8Y5K2 **Mass:** 34929 **Score:** 48 **Matches:** 1(1) **Sequences:** 1(1) **emPAI:** 0.13
Heme A synthase OS=Listeria monocytogenes serovar 1/2a (strain ATCC BAA-679 / EGD-e) OX=169963 GN=ctaA PE=3 SV=1

Query	Observed	Mr(expt)	Mr(calc)	ppm	Miss	Score	Expect	Rank	Unique	Peptide
2210	451.747	901.480	901.476	5.07	0	48	7.7e-05	1	U	R.LTDVTPEK.L

117. tr|Q8Y862 **Mass:** 49571 **Score:** 47 **Matches:** 1(1) **Sequences:** 1(1) **emPAI:** 0.09
Dihydrolipoyl dehydrogenase OS=Listeria monocytogenes serovar 1/2a (strain ATCC BAA-679 / EGD-e) OX=169963 GN=PdhD PE=3 SV=1

Query	Observed	Mr(expt)	Mr(calc)	ppm	Miss	Score	Expect	Rank	Unique	Peptide
7201	637.837	1273.659	1273.667	-5.93	0	47	8.3e-05	1	U	R.ALSLDAPEGFVR.L

118. tr|Q8Y4K7 **Mass:** 34436 **Score:** 45 **Matches:** 1(1) **Sequences:** 1(1) **emPAI:** 0.13
Lmo2431 protein OS=Listeria monocytogenes serovar 1/2a (strain ATCC BAA-679 / EGD-e) OX=169963 GN=lmo2431 PE=4 SV=1

Query	Observed	Mr(expt)	Mr(calc)	ppm	Miss	Score	Expect	Rank	Unique	Peptide
6832	623.794	1245.574	1245.584	-7.67	0	45	4.7e-05	1	U	R.QTTDASGVPDGAK.K

119. sp|Q8Y7G3 **Mass:** 46744 **Score:** 44 **Matches:** 1(1) **Sequences:** 1(1) **emPAI:** 0.09
Putative zinc metalloprotease Lmo1318 OS=Listeria monocytogenes serovar 1/2a (strain ATCC BAA-679 / EGD-e) OX=169963 GN=lmo1318 PE=3 SV=1

Query	Observed	Mr(expt)	Mr(calc)	ppm	Miss	Score	Expect	Rank	Unique	Peptide
8904	701.376	1400.737	1400.740	-2.00	0	44	0.00016	1	U	R.VGLELTPEETVSK.I

120. sp|Q8YA96 **Mass:** 135340 **Score:** 42 **Matches:** 2(2) **Sequences:** 2(2) **emPAI:** 0.06
DNA-directed RNA polymerase subunit beta' OS=Listeria monocytogenes serovar 1/2a (strain ATCC BAA-679 / EGD-e) OX=169963 GN=rpoC PE=3 SV=1

Query	Observed	Mr(expt)	Mr(calc)	ppm	Miss	Score	Expect	Rank	Unique	Peptide
3652	503.268	1004.521	1004.529	-7.86	0	33	0.0021	1	U	R.IQEIFEAR.N
7388	644.867	1287.719	1287.719	0.34	0	27	0.0051	1	U	R.IVELPITSNFR.E

121. tr|Q8Y7L9 **Mass:** 53628 **Score:** 42 **Matches:** 1(1) **Sequences:** 1(1) **emPAI:** 0.08
Lmo1255 protein OS=Listeria monocytogenes serovar 1/2a (strain ATCC BAA-679 / EGD-e) OX=169963 GN=lmo1255 PE=4 SV=1

Query	Observed	Mr(expt)	Mr(calc)	ppm	Miss	Score	Expect	Rank	Unique	Peptide
560	386.218	770.421	770.429	-9.53	0	42	8.6e-05	1	U	K.VDTAPIR.T

122. sp|O53083 **Mass:** 13109 **Score:** 41 **Matches:** 2(2) **Sequences:** 2(2) **emPAI:** 0.88
50S ribosomal protein L19 OS=Listeria monocytogenes serovar 1/2a (strain ATCC BAA-679 / EGD-e) OX=169963 GN=rplS PE=3 SV=1

Query	Observed	Mr(expt)	Mr(calc)	ppm	Miss	Score	Expect	Rank	Unique	Peptide

1125	416.241	830.468	830.475	-8.56	0	36	0.0017	1	U	K.LIDEITK.S
15168	956.463	1910.912	1910.960	-24.95	0	23	0.009	1	U	K.SQLNPDVPNFRPGDTVR.V

123. tr|Q8Y6T3 **Mass:** 36960 **Score:** 41 **Matches:** 1(1) **Sequences:** 1(1) **emPAI:** 0.12
Catabolite control protein A OS=Listeria monocytogenes serovar 1/2a (strain ATCC BAA-679 / EGD-e) OX=169963 GN=ccpA PE=4 SV=1

Query	Observed	Mr(expt)	Mr(calc)	ppm	Miss	Score	Expect	Rank	Unique	Peptide
8828	465.268	1392.781	1392.784	-1.63	0	41	0.00012	1	U	R.VVNGNPNVKPVTR.K

124. tr|Q8Y9I7 **Mass:** 33598 **Score:** 41 **Matches:** 3(3) **Sequences:** 3(3) **emPAI:** 0.46
Lmo0541 protein OS=Listeria monocytogenes serovar 1/2a (strain ATCC BAA-679 / EGD-e) OX=169963 GN=lmo0541 PE=4 SV=1

Query	Observed	Mr(expt)	Mr(calc)	ppm	Miss	Score	Expect	Rank	Unique	Peptide
3218	487.262	972.509	972.513	-3.92	0	20	0.047	1	U	K.DLLENLEK.V
9520	487.579	1459.714	1459.715	-1.15	0	22	0.015	1	U	K.GTESVGNDKPSIEK.I
14069	908.942	1815.869	1815.868	0.79	0	34	0.0014	1	U	K.VFDGLYSGAGFTPTDAAK.A

125. sp|Q8Y8N0 **Mass:** 57274 **Score:** 40 **Matches:** 1(1) **Sequences:** 1(1) **emPAI:** 0.08
ATP-dependent RNA helicase CshA OS=Listeria monocytogenes serovar 1/2a (strain ATCC BAA-679 / EGD-e) OX=169963 GN=cshA PE=2 SV=1

Query	Observed	Mr(expt)	Mr(calc)	ppm	Miss	Score	Expect	Rank	Unique	Peptide
8850	698.886	1395.757	1395.783	-18.81	0	40	0.00027	1	U	K.SNNVQALIIAPTR.E

126. sp|Q8Y651 **Mass:** 26478 **Score:** 39 **Matches:** 1(1) **Sequences:** 1(1) **emPAI:** 0.17
Manganese transport system ATP-binding protein MntB OS=Listeria monocytogenes serovar 1/2a (strain ATCC BAA-679 / EGD-e) OX=169963 GN=mntB PE=3 SV=

Query	Observed	Mr(expt)	Mr(calc)	ppm	Miss	Score	Expect	Rank	Unique	Peptide
4645	542.311	1082.607	1082.608	-1.69	0	39	0.00033	1	U	K.LTGIVGPNGAGK.S

127. tr|Q8Y4S2 **Mass:** 97191 **Score:** 37 **Matches:** 1(1) **Sequences:** 1(1) **emPAI:** 0.04
Transmembrane protein OS=Listeria monocytogenes serovar 1/2a (strain ATCC BAA-679 / EGD-e) OX=169963 GN=lmo2360 PE=4 SV=1

Query	Observed	Mr(expt)	Mr(calc)	ppm	Miss	Score	Expect	Rank	Unique	Peptide
7804	659.359	1316.704	1316.694	8.07	0	37	0.00072	1	U	K.LVSGVGQLADGSSK.I

128. tr|Q8Y5E9 **Mass:** 71596 **Score:** 37 **Matches:** 1(1) **Sequences:** 1(1) **emPAI:** 0.06
Lmo2115 protein OS=Listeria monocytogenes serovar 1/2a (strain ATCC BAA-679 / EGD-e) OX=169963 GN=lmo2115 PE=4 SV=1

Query	Observed	Mr(expt)	Mr(calc)	ppm	Miss	Score	Expect	Rank	Unique	Peptide
17974	821.467	2461.378	2461.379	-0.56	0	37	0.00021	1	U	K.SVVQNSPISTIPGLLVLPDSQVAK.I

129. tr|Q8YAR3 **Mass:** 74800 **Score:** 36 **Matches:** 1(1) **Sequences:** 1(1) **emPAI:** 0.06
Lmo0052 protein OS=Listeria monocytogenes serovar 1/2a (strain ATCC BAA-679 / EGD-e) OX=169963 GN=lmo0052 PE=4 SV=1

Query	Observed	Mr(expt)	Mr(calc)	ppm	Miss	Score	Expect	Rank	Unique	Peptide
4085	522.258	1042.501	1042.508	-6.85	0	36	0.00039	1	U	R.TFDAASYLR.S

130. sp|Q8Y915 **Mass:** 66125 **Score:** 34 **Matches:** 2(2) **Sequences:** 2(2) **emPAI:** 0.14
Glutamine--fructose-6-phosphate aminotransferase [isomerizing] OS=Listeria monocytogenes serovar 1/2a (strain ATCC BAA-679 / EGD-e) OX=169963 GN=g]

Query	Observed	Mr(expt)	Mr(calc)	ppm	Miss	Score	Expect	Rank	Unique	Peptide
7030	631.340	1260.665	1260.656	7.00	0	30	0.0088	1	U	K.TIIDEILSSDR.I
9787	744.404	1486.794	1486.799	-3.73	0	20	0.031	1	U	R.TLTLTNVPGSTLDR.E

131. sp|P66372 **Mass:** 15231 **Score:** 34 **Matches:** 2(2) **Sequences:** 2(2) **emPAI:** 0.73
30S ribosomal protein S12 OS=Listeria monocytogenes serovar 1/2a (strain ATCC BAA-679 / EGD-e) OX=169963 GN=rpsL PE=3 SV=1

Query	Observed	Mr(expt)	Mr(calc)	ppm	Miss	Score	Expect	Rank	Unique	Peptide
1942	442.267	882.519	882.529	-10.94	1	20	0.0095	1	U	R.VKDLPGVR.Y
2775	470.781	939.547	939.550	-3.05	0	27	0.0024	1	U	M.PTINQLVR.K

132. sp|P60384 **Mass:** 24185 **Score:** 34 **Matches:** 1(1) **Sequences:** 1(1) **emPAI:** 0.19
Redox-sensing transcriptional repressor Rex OS=Listeria monocytogenes serovar 1/2a (strain ATCC BAA-679 / EGD-e) OX=169963 GN=rex PE=3 SV=1

Query	Observed	Mr(expt)	Mr(calc)	ppm	Miss	Score	Expect	Rank	Unique	Peptide
5445	573.315	1144.615	1144.613	1.68	0	34	0.0038	1	U	K.IVAAFDVDPAK.V

133. sp|Q8Y7F1 **Mass:** 79780 **Score:** 33 **Matches:** 1(1) **Sequences:** 1(1) **emPAI:** 0.05
Polyribonucleotide nucleotidyltransferase OS=Listeria monocytogenes serovar 1/2a (strain ATCC BAA-679 / EGD-e) OX=169963 GN=pnp PE=3 SV=1

Query	Observed	Mr(expt)	Mr(calc)	ppm	Miss	Score	Expect	Rank	Unique	Peptide
1621	430.735	859.455	859.465	-11.58	0	33	0.0034	1	U	K.LGDEVTVK.V

134. tr|Q7AP48 **Mass:** 36179 **Score:** 33 **Matches:** 1(1) **Sequences:** 1(1) **emPAI:** 0.12
Lmo2550 protein OS=Listeria monocytogenes serovar 1/2a (strain ATCC BAA-679 / EGD-e) OX=169963 GN=lmo2550 PE=4 SV=1

Query	Observed	Mr(expt)	Mr(calc)	ppm	Miss	Score	Expect	Rank	Unique	Peptide
2079	446.747	891.480	891.481	-1.37	0	33	0.0018	1	U	R.VGFVDLSR.N

135. sp|Q48793 **Mass:** 35275 **Score:** 32 **Matches:** 1(1) **Sequences:** 1(1) **emPAI:** 0.13
Ribose-phosphate pyrophosphokinase 1 OS=Listeria monocytogenes serovar 1/2a (strain ATCC BAA-679 / EGD-e) OX=169963 GN=prs1 PE=3 SV=1

Query	Observed	Mr(expt)	Mr(calc)	ppm	Miss	Score	Expect	Rank	Unique	Peptide
5257	565.279	1128.544	1128.545	-1.04	0	32	0.0039	1	U	R.LLSDYFSER.H

136. tr|Q8Y4E1 **Mass:** 33138 **Score:** 32 **Matches:** 2(1) **Sequences:** 2(1) **emPAI:** 0.14
Cell division protein FtsX OS=Listeria monocytogenes serovar 1/2a (strain ATCC BAA-679 / EGD-e) OX=169963 GN=ftsX PE=3 SV=1

Query	Observed	Mr(expt)	Mr(calc)	ppm	Miss	Score	Expect	Rank	Unique	Peptide

149	354.208	706.401	706.401	-0.68	0	19	0.096	1	U	K.LVGAYGK.N
795	399.214	796.414	796.412	2.04	0	27	0.0042	1	U	K.NFELFK.Q

137. sp|Q8Y5V1 **Mass:** 43832 **Score:** 32 **Matches:** 1(1) **Sequences:** 1(1) **emPAI:** 0.10
Phosphopentomutase OS=Listeria monocytogenes serovar 1/2a (strain ATCC BAA-679 / EGD-e) OX=169963 GN=deoB PE=3 SV=1

Query	Observed	Mr(expt)	Mr(calc)	ppm	Miss	Score	Expect	Rank	Unique	Peptide
11597	819.388	1636.762	1636.794	-19.80	0	32	0.0014	1	U	K.ISDIFDGEGVTESIR.T

138. sp|Q927W3 **Mass:** 32234 **Score:** 30 **Matches:** 1(1) **Sequences:** 1(1) **emPAI:** 0.14
ATP synthase gamma chain OS=Listeria monocytogenes serovar 1/2a (strain ATCC BAA-679 / EGD-e) OX=169963 GN=atpG PE=3 SV=1

Query	Observed	Mr(expt)	Mr(calc)	ppm	Miss	Score	Expect	Rank	Unique	Peptide
463	380.232	758.450	758.454	-4.92	0	30	0.0044	1	U	M.ASLIDIK.Q

139. tr|Q8Y854 **Mass:** 36716 **Score:** 30 **Matches:** 1(1) **Sequences:** 1(1) **emPAI:** 0.12
Lmo1064 protein OS=Listeria monocytogenes serovar 1/2a (strain ATCC BAA-679 / EGD-e) OX=169963 GN=lmo1064 PE=4 SV=1

Query	Observed	Mr(expt)	Mr(calc)	ppm	Miss	Score	Expect	Rank	Unique	Peptide
4881	551.309	1100.604	1100.608	-3.09	0	30	0.0047	1	U	K.LIELEEVTR.N

140. sp|P60426 **Mass:** 30543 **Score:** 30 **Matches:** 1(1) **Sequences:** 1(1) **emPAI:** 0.15
50S ribosomal protein L2 OS=Listeria monocytogenes serovar 1/2a (strain ATCC BAA-679 / EGD-e) OX=169963 GN=rplB PE=3 SV=1

Query	Observed	Mr(expt)	Mr(calc)	ppm	Miss	Score	Expect	Rank	Unique	Peptide
5837	589.303	1176.591	1176.577	11.8	0	30	0.0054	1	U	R.VATIEYDPNR.S

141. sp|Q8Y7J8 **Mass:** 53193 **Score:** 29 **Matches:** 1(1) **Sequences:** 1(1) **emPAI:** 0.08
ATP-dependent protease ATPase subunit HslU OS=Listeria monocytogenes serovar 1/2a (strain ATCC BAA-679 / EGD-e) OX=169963 GN=hslU PE=3 SV=1

Query	Observed	Mr(expt)	Mr(calc)	ppm	Miss	Score	Expect	Rank	Unique	Peptide
10140	508.266	1521.777	1521.779	-1.10	0	29	0.0033	1	U	K.LIDADELAAEGIHR.A

142. tr|Q8Y7C7 **Mass:** 50297 **Score:** 29 **Matches:** 1(1) **Sequences:** 1(1) **emPAI:** 0.09
Acetyl-CoA carboxylase subunit (Biotin carboxylase subunit) OS=Listeria monocytogenes serovar 1/2a (strain ATCC BAA-679 / EGD-e) OX=169963 GN=lmo13

Query	Observed	Mr(expt)	Mr(calc)	ppm	Miss	Score	Expect	Rank	Unique	Peptide
2216	451.787	901.560	901.564	-3.98	0	29	0.0014	1	U	K.IGYPVIIK.A

143. tr|Q8Y7H4 **Mass:** 71838 **Score:** 28 **Matches:** 2(2) **Sequences:** 2(2) **emPAI:** 0.13
Transketolase OS=Listeria monocytogenes serovar 1/2a (strain ATCC BAA-679 / EGD-e) OX=169963 GN=tkt PE=3 SV=1

Query	Observed	Mr(expt)	Mr(calc)	ppm	Miss	Score	Expect	Rank	Unique	Peptide
5209	563.303	1124.592	1124.619	-23.70	0	20	0.024	1	U	R.QGLPTLPNSAK.L
9511	730.363	1458.711	1458.720	-6.36	0	25	0.0078	1	U	K.DGNDTAEILAAIEK.A

144. tr|Q8Y5T9 **Mass:** 25237 **Score:** 28 **Matches:** 1(1) **Sequences:** 1(1) **emPAI:** 0.18
Lmo1966 protein OS=Listeria monocytogenes serovar 1/2a (strain ATCC BAA-679 / EGD-e) OX=169963 GN=lmo1966 PE=4 SV=1

Query	Observed	Mr(expt)	Mr(calc)	ppm	Miss	Score	Expect	Rank	Unique	Peptide
2639	466.273	930.532	930.513	20.3	0	28	0.0061	1	U	R.TLLNDLSR.V

145. DECOY_tr|Q8Y8I5 **Mass:** 56050 **Score:** 28 **Matches:** 2(1) **Sequences:** 2(1) **emPAI:** 0.08
Lmo0917 protein OS=Listeria monocytogenes serovar 1/2a (strain ATCC BAA-679 / EGD-e) OX=169963 GN=lmo0917 PE=3 SV=1

Query	Observed	Mr(expt)	Mr(calc)	ppm	Miss	Score	Expect	Rank	Unique	Peptide
1648	432.247	862.480	862.480	0.44	0	28	0.0045	1	U	K.EFLDIVK.R
1846	439.728	877.442	877.429	14.6	0	19	0.078	2	U	R.TWDLSTR.F

146. sp|Q8YAB8 **Mass:** 57459 **Score:** 28 **Matches:** 1(1) **Sequences:** 1(1) **emPAI:** 0.08
Lysine--tRNA ligase OS=Listeria monocytogenes serovar 1/2a (strain ATCC BAA-679 / EGD-e) OX=169963 GN=lysS PE=3 SV=1

Query	Observed	Mr(expt)	Mr(calc)	ppm	Miss	Score	Expect	Rank	Unique	Peptide
5241	564.337	1126.660	1126.660	0.31	0	28	0.0024	1	U	K.LADLGDIIGIK.G

147. sp|Q8YAD4 **Mass:** 50122 **Score:** 27 **Matches:** 1(1) **Sequences:** 1(1) **emPAI:** 0.09
Bifunctional protein GlmU OS=Listeria monocytogenes serovar 1/2a (strain ATCC BAA-679 / EGD-e) OX=169963 GN=glmU PE=3 SV=1

Query	Observed	Mr(expt)	Mr(calc)	ppm	Miss	Score	Expect	Rank	Unique	Peptide
4598	540.306	1078.597	1078.602	-4.60	0	27	0.0035	1	U	R.AFIAAGSTITK.D

148. sp|P66401 **Mass:** 7370 **Score:** 27 **Matches:** 1(1) **Sequences:** 1(1) **emPAI:** 0.73
30S ribosomal protein S14 type Z OS=Listeria monocytogenes serovar 1/2a (strain ATCC BAA-679 / EGD-e) OX=169963 GN=rpsZ PE=3 SV=1

Query	Observed	Mr(expt)	Mr(calc)	ppm	Miss	Score	Expect	Rank	Unique	Peptide
3185	486.262	970.510	970.487	23.6	0	27	0.014	1	U	K.YAVQAYTR.C

149. sp|Q8Y441 **Mass:** 10909 **Score:** 27 **Matches:** 1(1) **Sequences:** 1(1) **emPAI:** 0.46
50S ribosomal protein L23 OS=Listeria monocytogenes serovar 1/2a (strain ATCC BAA-679 / EGD-e) OX=169963 GN=rplW PE=3 SV=1

Query	Observed	Mr(expt)	Mr(calc)	ppm	Miss	Score	Expect	Rank	Unique	Peptide
2343	456.229	910.443	910.444	-0.23	0	27	0.0051	1	U	K.EIQFFEV.-

150. sp|Q8Y766 **Mass:** 31446 **Score:** 27 **Matches:** 1(1) **Sequences:** 1(1) **emPAI:** 0.14
4-hydroxy-tetrahydrodipicolinate synthase OS=Listeria monocytogenes serovar 1/2a (strain ATCC BAA-679 / EGD-e) OX=169963 GN=dapA PE=3 SV=1

Query	Observed	Mr(expt)	Mr(calc)	ppm	Miss	Score	Expect	Rank	Unique	Peptide
8481	685.377	1368.740	1368.761	-15.76	0	27	0.0054	1	U	R.SVVNIEPETIIR.L

151. DECOY_tr|Q8Y8B9 **Mass:** 16251 **Score:** 26 **Matches:** 1(1) **Sequences:** 1(1) **emPAI:** 0.29
Lmo0989 protein OS=Listeria monocytogenes serovar 1/2a (strain ATCC BAA-679 / EGD-e) OX=169963 GN=lmo0989 PE=4 SV=1

Query	Observed	Mr(expt)	Mr(calc)	ppm	Miss	Score	Expect	Rank	Unique	Peptide
1459	425.738	849.462	849.474	-14.00	1	26	0.0096	1	U	K.SKIMISR.I

152. tr|Q8Y561 **Mass:** 28493 **Score:** 26 **Matches:** 1(1) **Sequences:** 1(1) **emPAI:** 0.16
Lmo2215 protein OS=Listeria monocytogenes serovar 1/2a (strain ATCC BAA-679 / EGD-e) OX=169963 GN=lmo2215 PE=4 SV=1

Query	Observed	Mr(expt)	Mr(calc)	ppm	Miss	Score	Expect	Rank	Unique	Peptide
6749	620.378	1238.741	1238.735	4.88	0	26	0.0024	1	U	K.QIVGLIGLNGAGK.S

153. DECOY_tr|Q8Y5B2 **Mass:** 87682 **Score:** 26 **Matches:** 1(1) **Sequences:** 1(1) **emPAI:** 0.05
Ribonucleoside-diphosphate reductase OS=Listeria monocytogenes serovar 1/2a (strain ATCC BAA-679 / EGD-e) OX=169963 GN=lmo2155 PE=3 SV=1

Query	Observed	Mr(expt)	Mr(calc)	ppm	Miss	Score	Expect	Rank	Unique	Peptide
1596	429.261	856.508	856.502	7.39	0	26	0.014	1	U	R.VEVEILR.E

154. sp|Q8Y557 **Mass:** 32734 **Score:** 26 **Matches:** 3(3) **Sequences:** 1(1) **emPAI:** 0.29
Foldase protein PrsA 2 OS=Listeria monocytogenes serovar 1/2a (strain ATCC BAA-679 / EGD-e) OX=169963 GN=prsA2 PE=3 SV=1

Query	Observed	Mr(expt)	Mr(calc)	ppm	Miss	Score	Expect	Rank	Unique	Peptide
1196	419.202	836.389	836.374	18.7	0	22	0.019	2	U	K.MDPAFEK.A 1197
1535	427.196	852.378	852.369	10.3	0	(20)	0.035	1	U	K.MDPAFEK.A

155. sp|Q8Y624 **Mass:** 60333 **Score:** 25 **Matches:** 1(1) **Sequences:** 1(1) **emPAI:** 0.07
Formate--tetrahydrofolate ligase OS=Listeria monocytogenes serovar 1/2a (strain ATCC BAA-679 / EGD-e) OX=169963 GN=fhs PE=3 SV=2

Query	Observed	Mr(expt)	Mr(calc)	ppm	Miss	Score	Expect	Rank	Unique	Peptide
502	382.217	762.420	762.428	-9.62	0	25	0.015	1	U	K.QIVEFK.K

156. tr|Q8Y765 **Mass:** 43169 **Score:** 25 **Matches:** 1(1) **Sequences:** 1(1) **emPAI:** 0.10
Aspartokinase OS=Listeria monocytogenes serovar 1/2a (strain ATCC BAA-679 / EGD-e) OX=169963 GN=lmo1436 PE=3 SV=1

Query	Observed	Mr(expt)	Mr(calc)	ppm	Miss	Score	Expect	Rank	Unique	Peptide
2233	452.742	903.469	903.466	2.89	0	25	0.016	1	U	K.ITEVDTAR.L

157. tr|Q8Y5V7 **Mass:** 27493 **Score:** 25 **Matches:** 3(2) **Sequences:** 1(1) **emPAI:** 0.16
ResD protein OS=Listeria monocytogenes serovar 1/2a (strain ATCC BAA-679 / EGD-e) OX=169963 GN=resD PE=4 SV=1

Query	Observed	Mr(expt)	Mr(calc)	ppm	Miss	Score	Expect	Rank	Unique	Peptide
1016	411.223	820.431	820.444	-16.49	1	23	0.026	1	U	R.ELREFK.S 1014 1017

158. tr|Q8Y486 **Mass:** 61859 **Score:** 25 **Matches:** 1(1) **Sequences:** 1(1) **emPAI:** 0.07
Lmo2569 protein OS=Listeria monocytogenes serovar 1/2a (strain ATCC BAA-679 / EGD-e) OX=169963 GN=lmo2569 PE=4 SV=1

Query	Observed	Mr(expt)	Mr(calc)	ppm	Miss	Score	Expect	Rank	Unique	Peptide
3679	504.267	1006.519	1006.533	-14.07	0	25	0.014	1	U	K.YDELVLGAK.G

159. tr|Q8Y7L4 **Mass:** 8455 **Score:** 24 **Matches:** 2(1) **Sequences:** 1(1) **emPAI:** 0.62
Lmo1263 protein OS=Listeria monocytogenes serovar 1/2a (strain ATCC BAA-679 / EGD-e) OX=169963 GN=lmo1263 PE=4 SV=1

Query	Observed	Mr(expt)	Mr(calc)	ppm	Miss	Score	Expect	Rank	Unique	Peptide
1458	425.728	849.442	849.438	4.77	1	24	0.018	1	U	-.MKTVDTR.R 1456

160. sp|Q8Y7Q1 **Mass:** 88184 **Score:** 24 **Matches:** 1(1) **Sequences:** 1(1) **emPAI:** 0.05
Phenylalanine--tRNA ligase beta subunit OS=Listeria monocytogenes serovar 1/2a (strain ATCC BAA-679 / EGD-e) OX=169963 GN=pheT PE=3 SV=1

Query	Observed	Mr(expt)	Mr(calc)	ppm	Miss	Score	Expect	Rank	Unique	Peptide
3876	513.816	1025.617	1025.623	-6.59	0	24	0.0042	1	U	R.TSIVPQLIR.S

161. tr|Q8Y725 **Mass:** 40110 **Score:** 24 **Matches:** 1(1) **Sequences:** 1(1) **emPAI:** 0.11
Endolytic murein transglycosylase OS=Listeria monocytogenes serovar 1/2a (strain ATCC BAA-679 / EGD-e) OX=169963 GN=mltG PE=1 SV=1

Query	Observed	Mr(expt)	Mr(calc)	ppm	Miss	Score	Expect	Rank	Unique	Peptide
9540	732.367	1462.720	1462.730	-7.37	0	24	0.011	1	U	K.AYPETVTNDVLNK.S

162. tr|Q8Y5P2 **Mass:** 100803 **Score:** 23 **Matches:** 1(1) **Sequences:** 1(1) **emPAI:** 0.04
Lmo2014 protein OS=Listeria monocytogenes serovar 1/2a (strain ATCC BAA-679 / EGD-e) OX=169963 GN=lmo2014 PE=4 SV=1

Query	Observed	Mr(expt)	Mr(calc)	ppm	Miss	Score	Expect	Rank	Unique	Peptide
21	351.691	701.368	701.371	-3.39	0	23	0.022	1	U	K.LADVER.F

163. DECOY_tr|Q8Y3V9 **Mass:** 28563 **Score:** 23 **Matches:** 1(1) **Sequences:** 1(1) **emPAI:** 0.16
Lmo2722 protein OS=Listeria monocytogenes serovar 1/2a (strain ATCC BAA-679 / EGD-e) OX=169963 GN=lmo2722 PE=4 SV=1

Query	Observed	Mr(expt)	Mr(calc)	ppm	Miss	Score	Expect	Rank	Unique	Peptide
846	401.738	801.461	801.475	-16.68	1	23	0.027	1	U	R.DILWKK.R

164. sp|P02668 **Mass:** 21370 **Score:** 23 **Matches:** 1(1) **Sequences:** 1(1) **emPAI:** 0.22
Kappa-casein (Laboratory-Cont) OS=Bos taurus GN=CSN3 PE=1 SV=1

Query	Observed	Mr(expt)	Mr(calc)	ppm	Miss	Score	Expect	Rank	Unique	Peptide
6918	626.356	1250.698	1250.702	-3.08	0	23	0.011	1	U	K.YIPIQYVLSR.Y

165. tr|Q8Y3T6 **Mass:** 63084 **Score:** 23 **Matches:** 1(1) **Sequences:** 1(1) **emPAI:** 0.07
Lmo2745 protein OS=Listeria monocytogenes serovar 1/2a (strain ATCC BAA-679 / EGD-e) OX=169963 GN=lmo2745 PE=4 SV=1

Query	Observed	Mr(expt)	Mr(calc)	ppm	Miss	Score	Expect	Rank	Unique	Peptide
2065	446.724	891.433	891.412	23.8	0	23	0.021	1	U	K.MATEVGER.G

166. tr|Q8YAV5 **Mass:** 56730 **Score:** 22 **Matches:** 1(1) **Sequences:** 1(1) **emPAI:** 0.08
Cardiolipin synthase OS=Listeria monocytogenes serovar 1/2a (strain ATCC BAA-679 / EGD-e) OX=169963 GN=lmo0008 PE=3 SV=1

Query	Observed	Mr(expt)	Mr(calc)	ppm	Miss	Score	Expect	Rank	Unique	Peptide
2238	452.757	903.499	903.518	-21.28	0	22	0.022	1	U	R.LLLFDQR.N

167. tr|Q8YAD0 **Mass:** 134736 **Score:** 22 **Matches:** 1(1) **Sequences:** 1(1) **emPAI:** 0.03
Transcription-repair-coupling factor OS=Listeria monocytogenes serovar 1/2a (strain ATCC BAA-679 / EGD-e) OX=169963 GN=mfd PE=3 SV=1

Query	Observed	Mr(expt)	Mr(calc)	ppm	Miss	Score	Expect	Rank	Unique	Peptide
3395	494.274	986.533	986.540	-7.04	1	22	0.031	1	U	K.ILREEAEK.R

168. sp|Q8Y653 **Mass:** 34453 **Score:** 22 **Matches:** 1(1) **Sequences:** 1(1) **emPAI:** 0.13
Manganese-binding lipoprotein MntA OS=Listeria monocytogenes serovar 1/2a (strain ATCC BAA-679 / EGD-e) OX=169963 GN=mntA PE=1 SV=1

Query	Observed	Mr(expt)	Mr(calc)	ppm	Miss	Score	Expect	Rank	Unique	Peptide
4317	531.264	1060.514	1060.519	-4.79	0	22	0.02	1	U	K.FADLPENQK.T

169. sp|Q8Y6Y2 **Mass:** 25236 **Score:** 22 **Matches:** 1(1) **Sequences:** 1(1) **emPAI:** 0.18
UPF0758 protein lmo1549 OS=Listeria monocytogenes serovar 1/2a (strain ATCC BAA-679 / EGD-e) OX=169963 GN=lmo1549 PE=3 SV=1

Query	Observed	Mr(expt)	Mr(calc)	ppm	Miss	Score	Expect	Rank	Unique	Peptide
7006	630.362	1258.710	1258.707	2.55	1	22	0.02	1	U	K.ASKIMAAIELGR.R

170. DECOY_tr|Q8YA10 **Mass:** 39588 **Score:** 22 **Matches:** 1(1) **Sequences:** 1(1) **emPAI:** 0.11
Lmo0356 protein OS=Listeria monocytogenes serovar 1/2a (strain ATCC BAA-679 / EGD-e) OX=169963 GN=lmo0356 PE=4 SV=1

Query	Observed	Mr(expt)	Mr(calc)	ppm	Miss	Score	Expect	Rank	Unique	Peptide
572	386.714	771.413	771.424	-13.56	0	22	0.017	1	U	R.GEGLLQR.V

171. tr|Q7AP65 **Mass:** 45295 **Score:** 22 **Matches:** 1(1) **Sequences:** 1(1) **emPAI:** 0.10
OpuCA protein OS=Listeria monocytogenes serovar 1/2a (strain ATCC BAA-679 / EGD-e) OX=169963 GN=opuCA PE=4 SV=1

Query	Observed	Mr(expt)	Mr(calc)	ppm	Miss	Score	Expect	Rank	Unique	Peptide
2353	456.271	910.528	910.549	-22.69	0	22	0.0071	1	U	R.ENIVLVPK.L

172. tr|Q8Y851 **Mass:** 68799 **Score:** 22 **Matches:** 1(1) **Sequences:** 1(1) **emPAI:** 0.06
Lmo1067 protein OS=Listeria monocytogenes serovar 1/2a (strain ATCC BAA-679 / EGD-e) OX=169963 GN=lmo1067 PE=4 SV=1

Query	Observed	Mr(expt)	Mr(calc)	ppm	Miss	Score	Expect	Rank	Unique	Peptide
10392	769.445	1536.876	1536.888	-7.78	0	22	0.0065	1	U	K.ALEQNLTPIVVVNK.I

173. tr|Q8Y6I9 **Mass:** 97886 **Score:** 22 **Matches:** 1(1) **Sequences:** 1(1) **emPAI:** 0.04
Phosphatidylglycerol lysyltransferase OS=Listeria monocytogenes serovar 1/2a (strain ATCC BAA-679 / EGD-e) OX=169963 GN=mprF PE=3 SV=1

Query	Observed	Mr(expt)	Mr(calc)	ppm	Miss	Score	Expect	Rank	Unique	Peptide
2878	474.262	946.510	946.512	-2.86	0	22	0.028	1	U	K.LGSPFEAVK.V

174. tr|Q8Y6J6 **Mass:** 27226 **Score:** 22 **Matches:** 1(0) **Sequences:** 1(0)
Lmo1688 protein OS=Listeria monocytogenes serovar 1/2a (strain ATCC BAA-679 / EGD-e) OX=169963 GN=lmo1688 PE=4 SV=1

Query	Observed	Mr(expt)	Mr(calc)	ppm	Miss	Score	Expect	Rank	Unique	Peptide
2025	445.256	888.497	888.503	-7.15	0	22	0.056	1	U	K.VALVTGSSR.G

175. DECOY_tr|Q8Y8S2 **Mass:** 15143 **Score:** 21 **Matches:** 1(1) **Sequences:** 1(1) **emPAI:** 0.32
Lmo0822 protein OS=Listeria monocytogenes serovar 1/2a (strain ATCC BAA-679 / EGD-e) OX=169963 GN=lmo0822 PE=4 SV=1

Query	Observed	Mr(expt)	Mr(calc)	ppm	Miss	Score	Expect	Rank	Unique	Peptide
1624	430.745	859.475	859.459	19.5	0	21	0.049	1	U	K.ALPMGTVR.L

176. DECOY_tr|Q8Y8S9 **Mass:** 17314 **Score:** 21 **Matches:** 1(1) **Sequences:** 1(1) **emPAI:** 0.27
Lmo0815 protein OS=Listeria monocytogenes serovar 1/2a (strain ATCC BAA-679 / EGD-e) OX=169963 GN=lmo0815 PE=1 SV=1

Query	Observed	Mr(expt)	Mr(calc)	ppm	Miss	Score	Expect	Rank	Unique	Peptide
2056	446.239	890.464	890.482	-19.90	1	21	0.045	1	U	R.SATTRDIK.L

177. sp|Q8Y4A2 **Mass:** 45190 **Score:** 20 **Matches:** 1(1) **Sequences:** 1(1) **emPAI:** 0.10
UDP-N-acetylglucosamine 1-carboxyvinyltransferase 2 OS=Listeria monocytogenes serovar 1/2a (strain ATCC BAA-679 / EGD-e) OX=169963 GN=murA2 PE=3 S\

Query	Observed	Mr(expt)	Mr(calc)	ppm	Miss	Score	Expect	Rank	Unique	Peptide
11089	798.404	1594.793	1594.784	5.70	0	20	0.041	1	U	R.AEGSSVITDTIYPSR.F

178. sp|P67055 **Mass:** 27484 **Score:** 20 **Matches:** 1(1) **Sequences:** 1(1) **emPAI:** 0.16
Demethylmenaquinone methyltransferase OS=Listeria monocytogenes serovar 1/2a (strain ATCC BAA-679 / EGD-e) OX=169963 GN=menG PE=3 SV=1

Query	Observed	Mr(expt)	Mr(calc)	ppm	Miss	Score	Expect	Rank	Unique	Peptide
1201	419.206	836.398	836.403	-6.20	0	20	0.027	1	U	K.ISPSYDR.M

179. tr|Q8Y544 **Mass:** 49156 **Score:** 20 **Matches:** 1(1) **Sequences:** 1(1) **emPAI:** 0.09
Lmo2232 protein OS=Listeria monocytogenes serovar 1/2a (strain ATCC BAA-679 / EGD-e) OX=169963 GN=lmo2232 PE=4 SV=1

Query	Observed	Mr(expt)	Mr(calc)	ppm	Miss	Score	Expect	Rank	Unique	Peptide
2785	471.223	940.432	940.429	2.74	0	20	0.047	1	U	K.IFDFDER.M

180. tr|Q8Y745 **Mass:** 44037 **Score:** 20 **Matches:** 1(1) **Sequences:** 1(1) **emPAI:** 0.10
Oxygen-independent coproporphyrinogen-III oxidase-like protein OS=Listeria monocytogenes serovar 1/2a (strain ATCC BAA-679 / EGD-e) OX=169963 GN=he

Query	Observed	Mr(expt)	Mr(calc)	ppm	Miss	Score	Expect	Rank	Unique	Peptide
436	378.695	1510.752	1510.716	23.9	1	20	0.039	1	U	K.EKMEEEMFLGLR.K

181. DECOY_tr|Q8Y4U7 **Mass:** 13167 **Score:** 20 **Matches:** 1(1) **Sequences:** 1(1) **emPAI:** 0.37
Lmo2334 protein OS=Listeria monocytogenes serovar 1/2a (strain ATCC BAA-679 / EGD-e) OX=169963 GN=lmo2334 PE=4 SV=1

Query	Observed	Mr(expt)	Mr(calc)	ppm	Miss	Score	Expect	Rank	Unique	Peptide
3901	515.275	1028.536	1028.561	-24.76	1	20	0.037	1	U	K.NEIADAIRK.L

182. sp|Q8Y5M0 **Mass:** 35858 **Score:** 19 **Matches:** 1(1) **Sequences:** 1(1) **emPAI:** 0.12
Phospho-N-acetylmuramoyl-pentapeptide-transferase OS=Listeria monocytogenes serovar 1/2a (strain ATCC BAA-679 / EGD-e) OX=169963 GN=mraY PE=3 SV=1

Query	Observed	Mr(expt)	Mr(calc)	ppm	Miss	Score	Expect	Rank	Unique	Peptide
148	354.197	706.380	706.376	4.99	0	19	0.039	1	U	K.FGQSIR.D

183. DECOY_tr|Q8Y859 **Mass:** 20029 **Score:** 19 **Matches:** 2(2) **Sequences:** 1(1) **emPAI:** 0.23
Lmo1059 protein OS=Listeria monocytogenes serovar 1/2a (strain ATCC BAA-679 / EGD-e) OX=169963 GN=lmo1059 PE=4 SV=1

Query	Observed	Mr(expt)	Mr(calc)	ppm	Miss	Score	Expect	Rank	Unique	Peptide
5188	562.312	1122.609	1122.603	5.11	1	18	0.038	1	U	K.SYIKNITER.T 5190

184. sp|Q8Y6M4 **Mass:** 91895 **Score:** 18 **Matches:** 1(1) **Sequences:** 1(1) **emPAI:** 0.05
Leucine--tRNA ligase OS=Listeria monocytogenes serovar 1/2a (strain ATCC BAA-679 / EGD-e) OX=169963 GN=leuS PE=3 SV=1

Query	Observed	Mr(expt)	Mr(calc)	ppm	Miss	Score	Expect	Rank	Unique	Peptide
14	351.203	700.392	700.376	24.0	0	18	0.05	1	U	K.QDTIPK.Q

185. DECOY_tr|Q8Y5I2 **Mass:** 13344 **Score:** 18 **Matches:** 1(1) **Sequences:** 1(1) **emPAI:** 0.36
Lmo2080 protein OS=Listeria monocytogenes serovar 1/2a (strain ATCC BAA-679 / EGD-e) OX=169963 GN=lmo2080 PE=4 SV=1

Query	Observed	Mr(expt)	Mr(calc)	ppm	Miss	Score	Expect	Rank	Unique	Peptide
18110	830.729	2489.164	2489.159	2.12	0	18	0.029	1	U	K.GTTANTVATNSSVTIMVESAYDNK.E

186. tr|Q8Y5A8 **Mass:** 35957 **Score:** 18 **Matches:** 1(1) **Sequences:** 1(1) **emPAI:** 0.12
Lmo2160 protein OS=Listeria monocytogenes serovar 1/2a (strain ATCC BAA-679 / EGD-e) OX=169963 GN=lmo2160 PE=4 SV=1

Query	Observed	Mr(expt)	Mr(calc)	ppm	Miss	Score	Expect	Rank	Unique	Peptide
6140	600.367	1198.720	1198.728	-6.98	0	18	0.029	1	U	R.AVTNLQSILIK.D

Mascot: http://www.matrixscience.com/
